# Merging Regiodivergent Catalysis with Atom‐Economical Radical Arylation

**DOI:** 10.1002/anie.201908860

**Published:** 2019-08-28

**Authors:** Felix Mühlhaus, Hendrik Weißbarth, Tobias Dahmen, Gregor Schnakenburg, Andreas Gansäuer

**Affiliations:** ^1^ Kekulé-Institut für Organische Chemie und Biochemie Universität Bonn Gerhard Domagk-Straße 1 53121 Bonn Germany; ^2^ Institut für Anorganische Chemie Universität Bonn Gerhard Domagk-Straße 1 53121 Bonn Germany

**Keywords:** arylation, indoline, regiodivergent synthesis, tetrahydroquinoline, titanocene

## Abstract

A titanocene‐catalyzed regiodivergent radical arylation is described that allows access to either enantiomerically pure tetrahydroquinolines or indolines from a common starting material. The regioselectivity of epoxide opening that results in the high selectivity of heterocycle formation is controlled by two factors, the absolute configuration of the enantiopure ligands of the (C_5_H_4_R)_2_TiX_2_ catalyst and the inorganic ligand X (X=Cl, OTs). The overall reaction is atom‐economical and constitutes a radical Friedel–Crafts alkylation.

## Graphical abstract

**Indolines and tetrahydroquinolines** are formed from the same substrates by titanocene catalysis. The absolute configuration of the cyclopentadienyl ligand and the inorganic ligand determine the regioselectivity of the ring opening and diastereoselectivity of tje radical arylation in the regiodivergent radical arylation of epoxides.

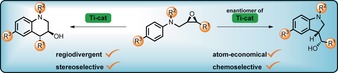

The design of catalytic methods to efficiently and highly chemo‐ and stereoselectively access small molecules with potential biological activity is a topic central to chemistry. To be attractive for potential applications, such processes have to meet the key requirements of sustainable chemistry. Essential points are that the reaction is atom‐economical and, thus, proceeds without the generation of waste, the use of readily available substrates, and mild reaction conditions. The choice of the catalyst is equally important. The use of earth‐abundant 3d transition metals^1^ that shuttle between neighboring oxidation states is particularly appealing.^2^

Herein, we show the validity of these points in a titanocene‐catalyzed^3^ regiodivergent radical arylation that allows access to enantiomerically pure tetrahydroquinolines or indolines from a common starting material through choice of the appropriate titanocene catalyst. In regiodivergent reactions, one constitutional isomer of a product is formed from an enantiomerically pure substrate by the action of one enantiomer of a catalyst and the other isomer by the action of the other enantiomer of the catalyst. In our case, two points are critical: First, the highly regioselective generation of either **R‐2** or **R‐3** from **1** by an electron transfer (ET) from titanium to the epoxide needs to be controlled by the absolute configuration of the titanocene catalyst (Scheme 1).

**Scheme 1:**
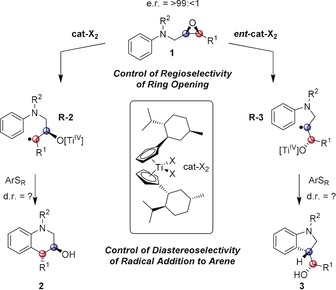
Mechanistic concept of the titanocene‐catalyzed regiodivergent radical arylation of epoxides.

Such regiodivergent processes^4^ that are the more general cases of desymmetrization reactions are highly desirable as branching points for the generation of molecular diversity (diversity‐oriented synthesis).^5^ Owing to their mild conditions and high chemoselectivity, ET‐promoted regiodivergent ring openings to radical intermediates are at least as advantageous as classical processes proceeding via an S_N_2 mechanism.^6^

Second, the radical intermediate generated through regiodivergent epoxide opening needs to add to the arene with high diastereoselectivity for the reaction to be useful.^7^

**R‐2′** may be considered a radical σ‐complex after the radical addition of **R‐2** to the arene (Scheme 2). Its rearomatization can occur via a proton coupled electron transfer (PCET) or by a stepwise ET‐proton‐transfer sequence.^8^ The overall process is an atom‐economical catalytic radical reaction and proceeds under much milder conditions than typical Friedel–Crafts alkylations^9^ that require strong electrophilic activation.

**Scheme 2:**
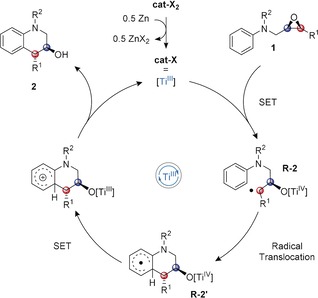
General catalytic cycle for the formation of tetrahydroquinolines **2**.

In this manner, it is possible to obtain either the desired tetrahydroquinolines **2** or indolines **3** as main products from the enantiomerically pure substrates **1**. Both classes of N‐heterocycles are common structural motifs in compounds with pertinent biological activity including natural products.^10^, ^11^

With substrate **1 a** (R^2^=Ph, R^1^=Pr, e.r.=>99:<1) the achiral Cp_2_TiCl_2_ and Cp_2_Ti(OTs)_2_ 12 display a preference for the formation of tetrahydroquinoline (THQ) **2 a** (Table 1, entry 1 and 2). Therefore, **R‐3 a** is disfavored by the inductive effect of the CH_2_NPh_2_ group.

**Table 1:** Catalyst control of regio‐ and diastereoselectivity in the regiodivergent arylation.

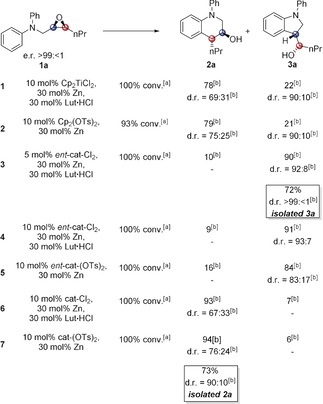

[a] Determined by ^1^H and ^13^C NMR spectroscopy. [b] Product ratio of tetrahydroquinoline to indoline and d.r.=diastereoselectivity of crude and isolated products as determined by ^13^C NMR spectroscopy.^13^

This raises the question as to whether this intrinsic selectivity can be overwhelmed by using enantiomerically pure titanocene complexes in a regiodivergent epoxide opening of **1 a**. We decided to start our investigations with enantiomerically pure Kagan's complex **cat‐X_2_** and ***ent*** **‐cat‐X_2_**.^14^ Both enantiomers of **cat‐Cl_2_** have been successfully used in the regiodivergent reduction of β‐hydroxy epoxides to 1,3‐ or 1,4‐diols with very high selectivity.6d However, in an example relevant to this study, the regiodivergent reduction of Sharpless epoxides to 1,2‐ or 1,3‐diols was not satisfactory.6c

In the matched case, the formation of **2 a** from **1 a** (Table 1, entry 7), **cat‐(OTs)_2_** (see Scheme 1 for structure) constitutes an efficient catalyst (for the synthesis and characterization of **cat‐(OTs)_2_** and structural assignments for the THQs **2**: see the Supporting Information). Regioselectivity of ring‐opening of **1 a** is high (94:6) and diastereoselectivity of the radical addition is noticeably superior to **cat‐Cl_2_**. Gratifyingly, in the mismatched case, the formation of **3 a** from **1 a**, the use of ***ent*** **‐cat‐Cl_2_** results in a high regioselectivity (90:10) of ring‐opening and even better diastereoselectivity (*trans*: *cis*=92:8) of radical arylation (Table 1, entry 4). However, ***ent*** **‐cat‐(OTs)_2_** leads to a decrease in regioselectivity as well as diastereoselectivity (for structural assignments for the indolines **3**, see the Supporting Information). Thus, with enantiomerically pure Kagan's catalysts, regioselectivity is almost completely reagent‐controlled, with **cat‐(OTs)_2_** being most suitable for THQ‐formation and ***ent*** **‐cat‐Cl_2_** for indoline formation. It should be noted that with ***ent*** **‐cat‐Cl_2_** lutidine hydrochloride (Lut⋅HCl) is crucial to reach full conversion (with the less acidic Coll⋅HCl: 54 %). NEt_3_⋅HCl leads to even slower reactions.

Employing **cat‐(OTs)_2_** does not require an additive for increasing conversion or improving catalyst stability.7c This is beneficial because decreasing the number of additives increases the atom economy of the reaction and prevents ligand scrambling. We note that strong counterion effects have been observed in titanocene catalysis with a metal‐free reducing agent.^15^ While the absolute configuration of the cyclopentadienyl ligands is the dominant factor in the control of the reaction, the effect of X should not be neglected.

The influence of X on the regioselectivity of epoxide opening can be explained by electronic effects. In the formation of **R‐3 a**, the electron deficiency at the radical center will be further increased by the more electron‐withdrawing −OTs. For the formation **R‐2 a** this inductive effect will be reduced because of the additional carbon between N and the radical center. Thus, the counterion effect on regioselectivity is noticeable for indoline formation.

The effect of X on the diastereoselectivity is more subtle (Scheme 3). The intramolecular addition of alkyl radicals to anilines is a highly exothermic reaction and will, therefore, proceed through early transition states.16a It has slightly lower rate constants than titanocene catalyzed 5‐*exo* cyclizations.16b The angle of attack of alkyl radicals to anilines has been calculated to be about 120° and the distance of the radical center to the arene has been determined to be about 2.15 Å.16a Replacing −Cl with the more electron‐deficient and larger −OTs will increase the steric demand of the [Ti^IV^] fragment.

**Scheme 3:**
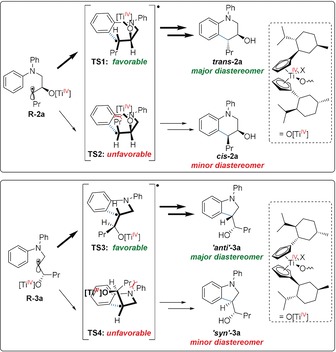
Analysis of diastereoselectivity in the radical arylation.

For THQ formation, transition state **TS2** is disfavored by an interaction of the Pr group with the O[Ti^IV^] group. In transition state **TS1**, such an interaction is absent. The larger −OTs counterion disfavors **TS2** further, yet does not affect **TS1** (Table 1).

For indoline formation the main interaction disfavoring **TS4** is the interaction of the Pr group with the CH_2_NPh_2_ group. Replacing X=Cl by X=OTs should have no substantial influence on the steric interactions in **TS4**.

In the favored **TS3**, the larger X=OTs will result in a stronger contact of X with the CH_2_NPh_2_ group. This results in a less favorable **TS3** for X=OTs, and a lower diastereoselectivity as observed for **3 a** with ***ent*** **‐cat‐(OTs)_2_** (Table 1, entry 4 and 5).

A key aspect of sustainability of any reaction is the substrate availability. Our synthesis of enantiopure epoxide **1** is short and modular (Scheme 4) and allows the preparation of a wide variety of substrates for the regiodivergent epoxide opening (REO) combined with radical arylation.

**Scheme 4:**
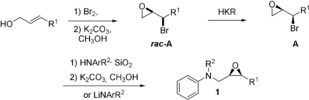
Modular synthesis of the enantiomerically pure substrates **1**.

The approach to **1** starts from readily available (*E*)‐allylic alcohols that are reacted with Br_2_ to give the corresponding dibromides that are transformed into the racemic α‐bromoepoxides ***rac*** **‐A** by stirring with K_2_CO_3_ in CH_3_OH.^17^ These compounds are resolved with Jacobsen's HKR^18^ to yield **A**. From **A**, **1** can either be obtained in one step by reaction with LiNArR^2^ or in two steps by the reaction of **A** with HNArR^2^ followed by treatment with K_2_CO_3_ in CH_3_OH.7c, 19 Substrates with potential protecting groups on N or with N−H bonds could not be easily accessed via the sequence and were therefore not investigated in the arylations.

The scope of the THQ formation is summarized in Table 2. The regiodivergent epoxide opening is efficient with R^1^ being a primary alkyl substituent.^6^ When stabilized radicals can be formed, only one product of epoxide opening is accessible. This will be the case for R^1^=Ar. Moreover, with respect to R^3^, only *p*‐substituted anilines were investigated. The reaction works well with aryl and alkyl substituents on N. For substrates with two aryl substituents on N, identical arenes were chosen to avoid issues of regioselectivity.

**Table 2:** Scope of tetrahydroquinoline synthesis via REO arylation.^[a]^

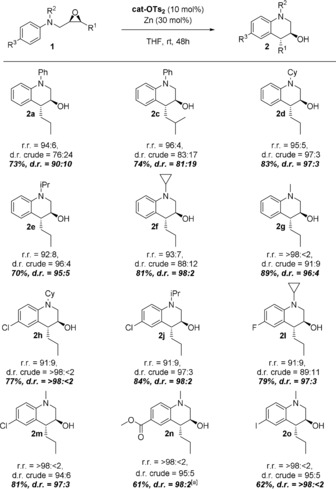

[a] r.r.=Ratio of tetrahydroquinoline to indoline, d.r. crude=diastereoselectivity of THQ formation as determined by ^13^C NMR spectroscopy. [b] 64 % conversion.^13^

The regioselectivity of epoxide openings (r.r.) is generally high (91:9–96:4) and essentially complete for R^2^=CH_3_ (>98:<2).

The diastereoselectivity of radical addition is strongly dependent on the nature of R^2^. Indeed, our initial reaction leading to **2 a** has the lowest diastereoselectivity of all examples. With R^2^=alkyl much better diastereoselectivities ranging from 88:12–97:3 (mostly 95:5 or higher) are observed.

The syntheses of **2 f**, **2 l**, **2 n**, and **2 o** deserve special comment. In many biologically active N‐heterocycles, N‐cyclopropyl substituents are important for the activity^20^ and are unaffected by the radical arylation (**2 f** and **2 l**). THQs with iodine substituents that are important intermediates for further functionalization can also be prepared with our method (**2 o**).

Thus, for N‐alkyl substituted substrates, the combination of regiodivergent epoxide opening, catalyst‐controlled diasteroselectivity of radical addition to the arene, and rearomatization of the radical σ‐complex via ET provides a highly selective entry to enantiomerically pure THQs.

The second class of N‐heterocycles that can be prepared from **1** by our method are indolines. The indoline scaffold is the key structure of numerous biologically active alkaloids.^11^ Its high relevance as pharmacophore is highlighted by its presence in about 4 % of all commercially available drugs.^21^ To investigate the practicability of the REO‐arylation for these heterocycles, we applied the catalytic system developed above (***ent*** **‐cat‐Cl_2_**+Lut⋅HCl, Table 1) to a number of substrates **1** (Table 3).

**Table 3:** Scope of indoline synthesis via REO arylation.

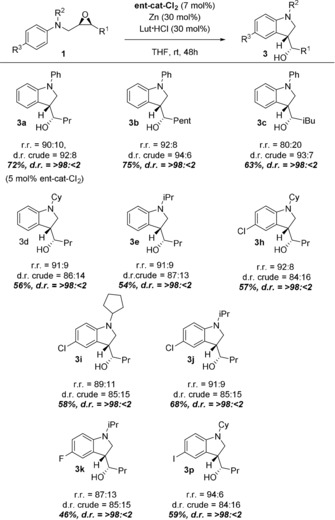

[a] r.r.=Ratio of indoline to tetrahydroquinoline, d.r. crude=diastereoselectivity of indoline formation as determined by ^13^C NMR spectroscopy.^13^

The regiodivergent epoxide opening is efficient with R^1^ being a primary alkyl substituent.^6^ The regioselectivity of epoxide opening leading to indoline formation is generally high (87:13–96:4, Table 3) and is only slightly influenced by the N‐substituent (R^2^=aryl, alkyl). The reactions of **1 c** to **2 c** and **3 c** display an interesting aspect of the regiodivergent epoxide opening. Increasing the steric bulk of one of the epoxide's substituents (R^1^=*i*Bu in **1 c**) results in a highly selective formation of **2 c**, r.r.=96:4 and a decreasing selectivity in the slightly less favorable formation of **3 c**, r.r.=80:20.6c

The diastereoselectivity is moderate to high (84:16–94:6). In contrast, for THQ formation the diastereoselectivity is highest for indolines with R^2^=aryl (90:10–94:6) and in the range of 85:15 for R^2^=alkyl. All indolines can be isolated as single diastereomers (d.r.=>98:<2).

Recently, the synthesis of indolines in racemic and enantiomerically pure form^22^ has been achieved by Co‐catalyzed metalloradical catalysis (MRC).2c However, in these complementary reactions, no radical addition to arenes is involved.

In summary, we have combined regiodivergent catalysis with titanocene(III) catalyzed radical arylation to an atom‐economical reaction that enables the synthesis of either enantiomerically pure indolines or tetrahydroquinolines from common epoxide precursors. We established that in addition to the dominating influence of the substituted cyclopentadienyl ligands, the ligand −OTs of the titanocene complexes improves both regioselectivity and diastereoselectivity of the reaction. Thus, the easy to carry out modification of the X ligand expands the scope of a given titanocene scaffold. This approach should also be of significant interest for other ET catalysts.2c

## Conflict of interest

The authors declare no conflict of interest.

## Supporting information

As a service to our authors and readers, this journal provides supporting information supplied by the authors. Such materials are peer reviewed and may be re‐organized for online delivery, but are not copy‐edited or typeset. Technical support issues arising from supporting information (other than missing files) should be addressed to the authors.

Supplementary
