# Supplementary material for: Merging Regiodivergent Catalysis with Atom‐Economical Radical Arylation
Source: Angew Chem Int Ed Engl. 2019 Aug 28;58(40):14208–12. doi: 10.1002/anie.201908860 (PMC6852184; doi:10.1002/anie.201908860)
Supplement: Supplementary file 1 — Supplementary [file ANIE-58-14208-s001.pdf]

Angewandte Chemie
Eine Zeitschrift der Gesellschaft Deutscher Chemiker

Supporting Information

Merging Regiodivergent Catalysis with Atom-Economical Radical Arylation

*Felix Mühlhaus[+], Hendrik Weißbarth[+], Tobias Dahmen, Gregor Schnakenburg, and Andreas Gansäuer**

anie_201908860_sm_miscellaneous_information.pdf

Table of Contents

1. General Information S5

1.1 Instruments S5

2. General Procedures S6

2.1 General procedure for bromination-epoxidation reaction sequence of allylic alcohols (GP1). . S6

2.2 General procedure for the hydrolytic kinetic resolution of terminal epoxides (GP2). S7

2.3.1 General procedure for the addition of anilinide derivatives to terminal epoxides (GP3a). S7

2.3.2 General procedure for the aminolysis-epoxidation reaction sequence (GP3b). S8

2.4 General procedures for the regiodivergent arylation of epoxides. S8

2.4.1 General procedure for the formation of tetrahydroquinolines GP4........ S8

2.4.2 General procedure for the formation of indolines (GP5). S9

3. Characterization of compounds S9

3.1 Synthesis of α-bromo-epoxides........ S9

3.1.1. Synthesis of *syn*-3-bromo-1,2-epoxy-hexane S9

3.1.2. Synthesis of *syn*-3-brom-1,2-epoxy-octane. S11

3.1.3. Synthesis of *syn*-3-brom-1,2-epoxy-5-methyl-hexane. S13

3.2 Kinetic resolution of α-bromo-epoxides (*rac*-A→A)........ S15

3.2.1 Kinetic resolution of *syn*-3-bromo-1,2-epoxy-hexane. S15

3.2.2 Kinetic resolution of *syn*-3-bromo-1,2-epoxy-octane. S15

3.2.3 Kinetic resolution of (*syn*)-3-bromo-1,2-epoxy-5-methyl-hexane. S16

3.3 Base mediated aminolysis of bromo-epoxides to aniline-epoxides (A→1) S17

3.3.1. Synthesis of *N*-(((*2R,3S*)-3-pentyloxiran-2-yl)methyl)-N-phenylaniline (1b). S17

3.3.2. Synthesis of *N*-(((*2R,3S*)-3-isobutyloxiran-2-yl)methyl)-N-phenylaniline (1c). S19

3.3.3. Synthesis of *N*-isopropyl-*N*-(((*2R,3S*)-3-propyloxiran-2-yl)methyl)aniline (1e). S21

3.3.4 Synthesis of *N*-Cyclopropyl-*N*-(((2R,3S)-3-propyloxirane-2-yl)methyl)aniline (1f). S23

3.3.5 Synthesis of 4-chloro-*N*-cyclohexyl-*N*-(((2R,3S)-3-propyloxirane-2-yl)methyl)aniline (1h). S25

3.3.6 Synthesis of 4-chloro-*N*-cyclopentyl-*N*-(((*2R,3S*)-3-propyloxirane-2-yl)methyl)aniline (1i). S27

3.3.7 Synthesis of 4-chloro-*N*-isopropyl-*N*-(((*2R,3S*)-3-propyloxiran-2-yl)methyl)aniline (1j). ... S29

3.3.8 Synthesis of 4-fluoro-*N*-isopropyl-*N*-(((*2R,3S*)-3-propyloxirane-2-yl)methyl)aniline (1k).. S31

3.3.9 Synthesis of *N*-cyclopropyl-4-fluoro-*N*-(((*2R,3S*)-3-propyloxiran-2-yl)methyl)aniline (1l). S34

3.3.10 Synthesis of methyl 4-(methyl(((*2R,3S*)-3-propyloxiran-2-yl)methyl)amino)benzoate (1n). S37

3.4 SiO_2-catalyzed aminolysis of bromo-epoxides to bromo-alcohols and subsequent formation of aniline-epoxides (A→1). S39

3.4.1. Synthesis of *N*-phenyl-*N*-(((*2R,3S*)-3-propyloxiran-2-yl)methyl)aniline (1a). S39

3.4.2. Synthesis of *N*-cyclohexyl-*N*-(((*2R,3S*)-3-propyloxiran-2-yl)methyl)aniline (1d). S42

3.4.3. Synthesis of *N*-methyl-*N*-(((*2R,3S*)-3-propyloxiran-2-yl)methyl)aniline (1g). S45

3.4.4. Synthesis of 4-chloro-*N*-methyl-*N*-(((*2R,3S*)-3-propyloxiran-2-yl)methyl)aniline (1m)..... S48

3.5.1. Synthesis of 4-iodo-N-methyl-N-(((2R,3S)-3-propyloxiran-2-yl)methyl)aniline (1o). S51

3.5.2 Synthesis of *N*-cyclohexyl-4-iodo-*N*-(((*2R,3S*)-3-propyloxiran-2-yl)methyl)aniline (1p). S53

4. REO-ArS$_R$ S55

4.1 Investigation of different catalysts for the REO-ArS$_R$ with epoxide 1a. S55

4.2 Structural assignment of the tetrahydroquinoline-scaffold and configurational analysis with tetrahydroquinolines 2a and 2g. S56

4.3 Synthesis of tetrahydroquinolines by REO-ArS$_R$ S59

4.3.1 Synthesis of (3*S*,4*R*)-1-Phenyl-4-propyl)-1,2,3,4-tetrahydroquinoline-3-ol (2a). S59

4.3.2 Synthesis of (3*S*,4*R*)-4-isobutyl-1-phenyl-4-propyl)-1,2,3,4-tetrahydroquinoline-3-ol (2c). S61

4.3.3 Synthesis of (3*S*,4*R*)-1-cyclohexyl-4-propyl)-1,2,3,4-tetrahydroquinoline-3-ol (2d). S63

4.3.4 Synthesis of (3*S*,4*R*)-1-*iso*propyl-4-propyl)-1,2,3,4-tetrahydroquinoline-3-ol (2e). S65

4.3.5 Synthesis of (3*S,4R*)-1-cyclopropyl-4-propyl-1,2,3,4-tetrahydroquinolin-3-ol (2f). S67

4.3.6 Synthesis of (3*S*,4*R*)-1-methyl-4-propyl)-1,2,3,4-tetrahydroquinoline-3-ol (2g). S69

4.3.7 Synthesis of (3*S*,4*R*)-6-chloro-1-cyclohexyl-4-propyl-1,2,3,4-tetrahydroquinolin-3-ol (2h). S71

4.3.8 Synthesis of (3*S*,4*R*)-6-chloro-1-isopropyl-4-propyl-1,2,3,4-tetrahydroquinolin-3-ol (2j). S73

4.3.9 (3*S*,4*R*)-1-cyclopropyl-6-fluoro-4-propyl-1,2,3,4-tetrahydroquinolin-3-ol (2l). S75

4.3.10 Synthesis of (3*S*,4*R*)-6-chloro-1-methyl-4-propyl)-1,2,3,4-tetrahydroquinoline-3-ol (2m). S78

4.3.11 Synthesis of Methyl-*(*3*S*,4*R)*-3-hydroxy-1-methyl-4-propyl-1,2,3,4-tetrahydroquinoline-6-carboxylate (2n).. S80

4.3.12 Synthesis of (3*S*,4*R*)-6-iodo-1-methyl-4-propyl)-1,2,3,4-tetrahydroquinoline-3-ol (2o). S82

4.4. Synthesis of indolines 3 by REO-ArS_R S84

4.4.1 (*S*)-1-((*R*)-1-phenylindolin-3-yl)-butan-1-ol (3a). S84

4.4.2 Synthesis of (*S*)-1-((*R*)-1-phenylindolin-3-yl)-hexan-1-ol (3b). S86

4.4.3 Synthesis of (*S*)-3-methyl-1-((*R*)-1-phenylindolin-3-yl)-butan-1-ol (3c). S88

4.4.4 Synthesis of (*S*)-1-((*R*)-1-cyclohexylindolin-3-yl)-butan-1-ol (3d)..................................... S90

4.4.5 Synthesis of (*S*)-1-((*R*)-1-isopropylindolin-3-yl)-butan-1-ol (3e)....................................... S92

4.4.6 Synthesis of (*S*)-1-((*R*)-5-chloro-1-cyclohexylindolin-3-yl)butan-1-ol (3h). S94

4.4.7 Synthesis of (*S*)-1-((*R*)-5-chloro-1-cyclopentylindolin-3-yl)butan-1-ol (3i)......................... S96

4.4.8 Synthesis of (*S*)-1-((*R*)-5-chloro-1-isopropylindolin-3-yl)butan-1-ol (3j). S98

4.4.9 Synthesis of (*S*)-1-((*R*)-5-fluoro-1-isopropylindolin-3-yl)butan-1-ol (3k)......................... S100

4.4.10 Synthesis of (*S*)-1-((*R*)-1-cyclohexyl-5-iodoindolin-3-yl)butan-1-ol (3p). S103

5. Synthesis of catalysts for the REO-ArS_R.. S105

5.1 Synthesis of cat-$(OTs)_2$.. S105

6. References.. S107

1. General Information

All Reactions involving air- and moisture sensitive substances were carried out in flame dried glassware under argon atmosphere using standard Schlenk technique. The THF used in the reactions was freshly distilled over Na before use. All reactions were monitored by thin-layer chromatography (TLC) on *Merck* silica gel F_{254} plates using UV light as visualizing agent (if applicable) and a solution of ammoniummolybdate tetrahydrate (25g/L) and $Ce(SO_4)_2{\bullet}4H_2O$ (10g/L) in 10% aqueous H_2SO_4 followed by heating as developing agents. The crude products were purified by Flash column chromatography on *Merck* silica gel 50 if not stated otherwise.

1.1 Instruments

^{1}H NMR and ^{13}C NMR spectra were measured on *Bruker* AMX 300 MHz, 400 MHz or 500 MHz spectrometers. ^{1}H NMR chemical shifts (δ_H) are given in ppm and calibrated by using the residual peak of the undeuterated solvent ($CHCl_3$ 7.26 ppm or C_6H_5D 7.16 ppm) as internal reference. ^{13}C NMR shifts are noted in ppm (δ_C) using the solvent peak as internal reference ($CDCl_3$ 77.0 ppm or C_6D_6 128.0 ppm). Coupling constants are reported in Hz and represent $J_{H,H}$ couplings, unless explicitly stated otherwise. The diastereomeric and regioisomeric ratios of the products were determined by ^{13}C NMR spectroscopy of the crude mixtures. It has been demonstrated that the NMR techniques used here are accurate for the determination of diastereomeric and regioisomeric ratios.[1] Compared to ^{1}H NMR spectroscopy the errors of the ratios in ^{13}C NMR spectroscopy are typically less than 2% and therefore within experimental error. IR spectra were recorded on *Nicolet* ATR-IR-Spectrometer TM 380 as neat films on KBr plates. High resoltution mass spectra were measured using a *Thermo Fisher Scientific* Orbitrap XL mass spectrometer by ESI (+) measurement. Enantiomeric ratios were determined by chiral HPLC on a *Daicel* Chiralpak IC-U column. The α_D^{20} values were measured in chloroform (10 g/L) on the MCP 150 polarimeter by *Anton Paar*.

The data collection for the single crystal x-Ray analysis was performed on a Bruker D8-Venture diffractometer using multi-layer optics monochromated Cu-$K\alpha$ irradiation (λ = 1.54178 Å). The diffractometer was equipped with a low-temperature device (Oxford Cryostream 800er series, Oxford Cryosystems, 100(2) K). Intensities were measured by fine-slicing ω and φ-scans and

corrected for background, polarization and Lorentz effects. For all data sets an empirical absorption correction was applied. The structures were solved by intrinsic phasing methods and refined anisotropically by the least-square procedure implemented in the SHELX program system.[2] All hydrogen atoms were included using the riding model on the bound carbon atoms.

CCDC numbers 1910522 (**2g**) and 1910523 (**3a**) contain the supplementary crystallographic data for this paper, which can be obtained free of charge from the Cambridge Crystallographic Data Centre via www.ccdc.cam.ac.uk/data_request/cif.

2. General Procedures

2.1 General procedure for bromination-epoxidation reaction sequence of allylic alcohols (GP1).

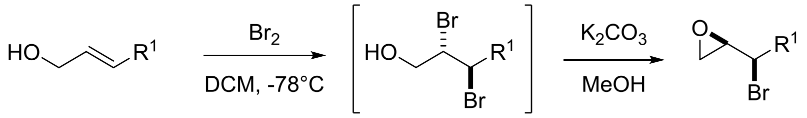

A solution of allylic alcohol (1.0 eq.) in CH_2Cl_2 (0.5 mmol/mL) is cooled to -78°C. Over a period of 1h bromine (1.0 eq.) is added via dropping funnel and the reaction is stirred for 1h at -78°C. The reaction is quenched by addition of saturated $NaHSO_3$ solution (2 mL/mmol substrate). After warming up to room temperature ¾ of the solvent is removed, phases are separated, and the aqueous phase is extracted twice with CH_2Cl_2. The combined organic extracts are dried over $MgSO_4$ and the solvent is removed under reduced pressure. The product was used without further purification.[3] α-bromo alcohol (1.0 eq.) is dissolved in methanol (2 mL/mmol) and finely ground K_2CO_3 (2.0 eq.) is added. The mixture is stirred for 3 h and the reaction progress is monitored by TLC. After complete conversion ¾ of the methanol is removed *in vacuo* and the residue is mixed with ethyl acetate (2 mL/mmol). K_2CO_3 is removed via vacuum filtration and the solvent is evaporated. The crude product is purified via flash column chromatography (silica, Eluent: CH : MTBE 98:2) or distillation (60°C, 10 mbar) depending on reaction scale.[4]

2.2 General procedure for the hydrolytic kinetic resolution of terminal epoxides (GP2).

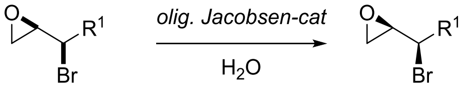

(S,S)- or (R,R)-oligomeric *Jacobsen* catalyst (0.05 mg/mmol substrate) is added to the racemic terminal epoxide (1.0 eq.). H_2O (0.53 eq.) is added dropwise and after completed addition the reaction mixture is stirred for 16h. The crude product is purified via flash column chromatography (silica, Eluent: CH : MTBE 98:2).[5] The er of the product is determined via chiral HPLC.

2.3.1 General procedure for the addition of anilinide derivatives to terminal epoxides (GP3a).

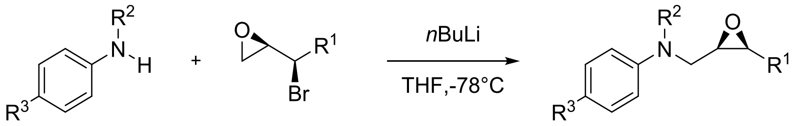

A solution of aniline derivative (1.0 eq.) in THF (0.5 mmol/mL) is cooled down to -78°C and *n*BuLi solution (2.5M in hexane, 1.2 eq.) is added dropwise. The mixture is stirred for 30 min followed by addition of the terminal *syn*-bromo-epoxide (1.2 eq.). The reaction is allowed to warm to room temperature over 16h. The reaction is quenched by addition of saturated NH_4Cl solution (2 mL/mmol aniline). The phases are separated, and the aqueous layer is extracted with diethylether three times. The combined organic extracts are washed with water and brine, then dried over Na_2SO_4 and the solvent is removed. The crude product is purified via flash column chromatography (silica, Eluent: CH : MTBE, 98:2). The resulting compounds can be air sensitive.[6]

2.3.2 General procedure for the aminolysis-epoxidation reaction sequence (GP3b).

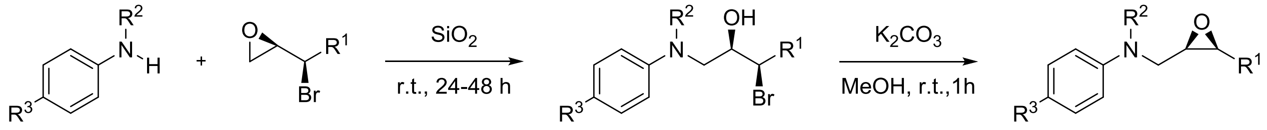

A round bottom flask is charged with Aniline derivative (1.0 eq.), terminal *syn*-bromo-epoxide (1.0 eq.) and SiO_2 (20% of the weight of both reactants). If not stated otherwise the reaction mixture is stirred at room temperature for 24-48h and progress is monitored via TLC. 5 mL of DCM are added, and the suspension is allowed to stir for another 30 min. The mixture is filtered, and all volatiles are removed *in vacuo*. The crude is purified via flash column chromatography (SiO_2, Eluent: CH : MTBE, 97:3). The obtained *syn*-bromo-alcohol (1.0 eq.) is dissolved in 5 mL MeOH and freshly ground K_2CO_3 (2.0 eq.) is added. The mixture is heated to 40°C and is stirred for 1h. The reaction progress is monitored via TLC. After cooling to room temperature 50 mL of Et_2O are added, K_2CO_3 is removed via filtration and the solution is concentrated *in vacuo*. The crude product is purified via flash column chromatography (SiO_2, eluent: CH : MTBE 99:1).[7]

2.4 General procedures for the regiodivergent arylation of epoxides.

2.4.1 General procedure for the formation of tetrahydroquinolines GP4.

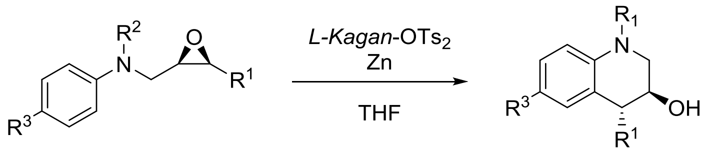

A Schlenk tube is charged with **cat-(OTs)₂** (= *L-Kagan*-(OTs)₂) (*L*-cat-OTs2 40 mg, 0.05 mmol, 0.1 eq.) and zinc powder (10 mg, 0.15 mmol, 0.3 eq.). The tube is evacuated for 15 min and then flushed with argon. Afterwards 1 mL of dry THF is added, which results in a red solution. The solution is stirred for at least five minutes. Once the color of the solution has changed from red to turquoise the substrate (0.5 mmol, 1.0 eq.) is added via syringe. The syringe is flushed with 1.5 mL of THF and the reaction mixture is allowed to stir for 48 h at room temperature. Afterwards the reaction mixture is filtered through a silica plug and flushed with Diethylether. The solvent is removed under reduce pressure and the crude product is purified via flash column chromatography (silica, Eluent: CH : MTBE 9:1). The diastereoselectivity and the regioselectivity is determined by ^{13}C NMR analysis of the crude product.

2.4.2 General procedure for the formation of indolines (GP5).

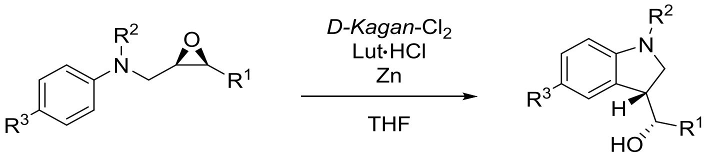

A Schlenk tube is charged with lutidine hydrochloride (Lut•HCl 22 mg, 0.15 mmol, 0.3 eq.), which is resublimed *in vacuo*. Zinc powder (10 mg, 0.15 mmol, 0.3 eq.) and ***ent*-cat-Cl_2** (= D-Kagans-complex) 18.4 mg, 0.035 mmol, 0.07 eq.) are added and the Schlenk tube is evacuated for 15 min. Afterwards 1 mL of dry THF is added, which results in a red solution. The solution is stirred until the color of the solution has changed from red to green. Then the substrate (0.5 mmol, 1.0 eq.) is added via syringe. The syringe is flushed with 1.5 mL of THF and the reaction mixture is allowed to stir for 48 h at room temperature. Afterwards the reaction mixture is filtered through a silica plug and flushed with Diethylether. The solvent is removed under reduce pressure and the crude product is purified via flash column chromatography (silica, Eluent: CH : MTBE 9:1). The diastereoselectivity and the regioselectivity is determined by ^{13}C NMR analysis of the crude product.

3. Characterization of compounds

3.1 Synthesis of α-bromo-epoxides

3.1.1. Synthesis of *syn*-3-bromo-1,2-epoxy-hexane

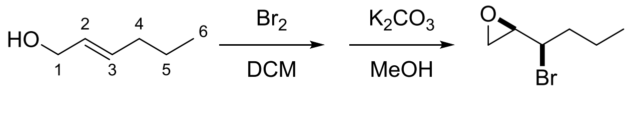

20.0 g of *(E)*-hex-2-en-1-ol (200 mmol, 1.0 eq.) are reacted with 32.0 g of bromine (10.3 mL, 200 mmol, 1.0 eq.) following GP1. After workup, the crude product is reacted with 55.3 g K_2CO_3 (400 mmol, 2.0 eq.) in MeOH. After distillation (60°C, 10 mbar), 25.8 g (144 mmol, 72%) of *syn*-3-bromo-1,2-epoxyhexane are obtained as a colorless liquid.

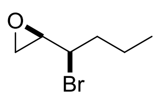

$C_6H_{11}BrO$
179.05 g/mol

R_f = 0.40 (10% Et_2O in CH),**^{1}H-NMR (500 MHz, $CDCl_3$, RT): δ [ppm]** = 0.95 (t, 3H, *J* = 7.4 Hz), 1.36-1.71 (m, 1H), 1.52-1.65 (m, 1H), 1.86 (q, 2H, *J* = 7.5 Hz), 2.74 (dd, 1H, *J* = 4.8, *J* = 2.5 Hz), 2.97 (dd, 1H, *J* = 4.8 Hz, *J* = 3.8), 3.21 (ddd, 1H, *J* = 7.6 Hz, *J* = 3.8 Hz, *J* = 2.4 Hz), 3.65-3.71 (m, 1H). **^{13}C-NMR (125 MHz, $CDCl_3$, RT): δ [ppm]** = 13.6, 20.8, 37.1, 49.0, 55.8, 55.9. **IR ṽ [cm^{-1}]** = 517, 525, 611, 693, 749, 761, 798, 854, 873, 928, 1193, 1252, 1465, 2933, 2961.

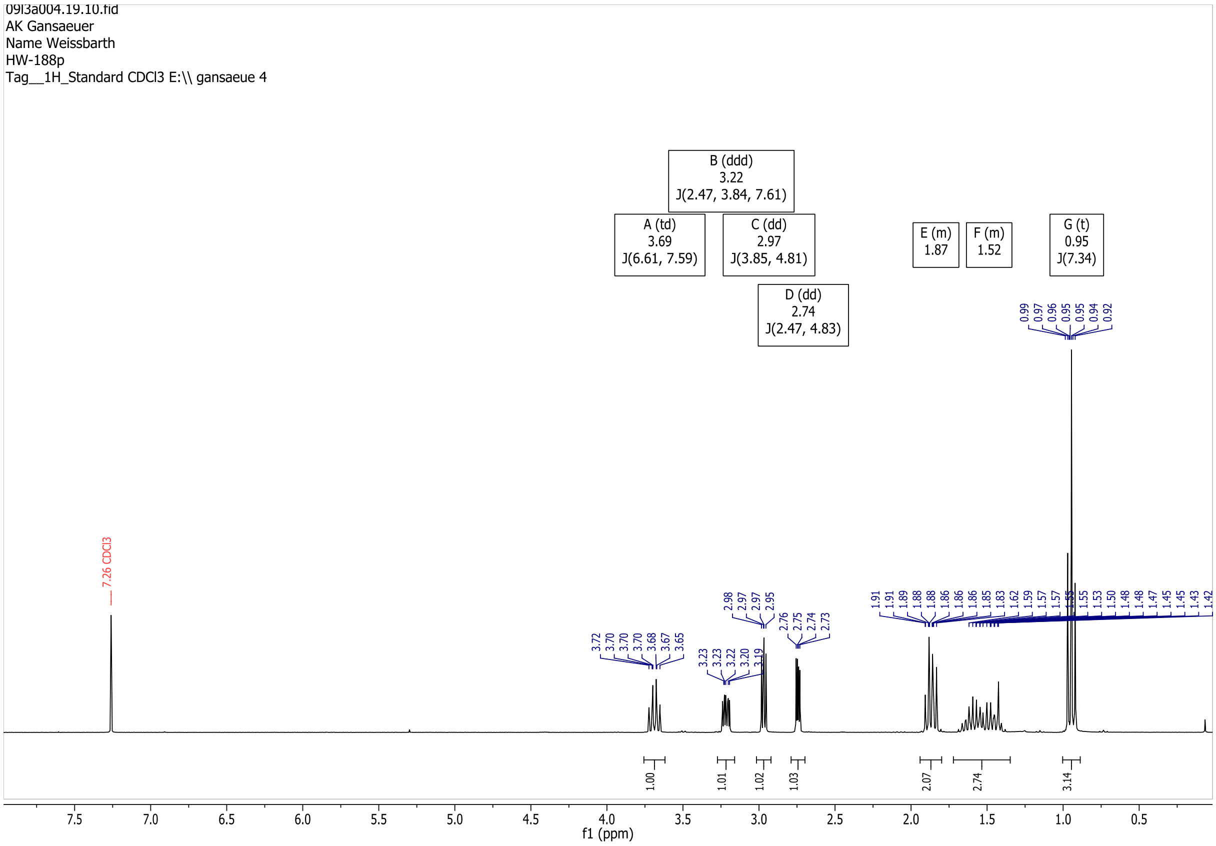
09l3a004.19.10.fid
AK Gansaeuer
Name Weissbarth
HW-188p
Tag__1H_Standard CDCl3 E:\\ gansaeue 4
A (td) 3.69 J(6.61, 7.59)
B (ddd) 3.22 J(2.47, 3.84, 7.61)
C (dd) 2.97 J(3.85, 4.81)
D (dd) 2.74 J(2.47, 4.83)
E (m) 1.87
F (m) 1.52
G (t) 0.95 J(7.34)
7.26 CDCl3
f1 (ppm)

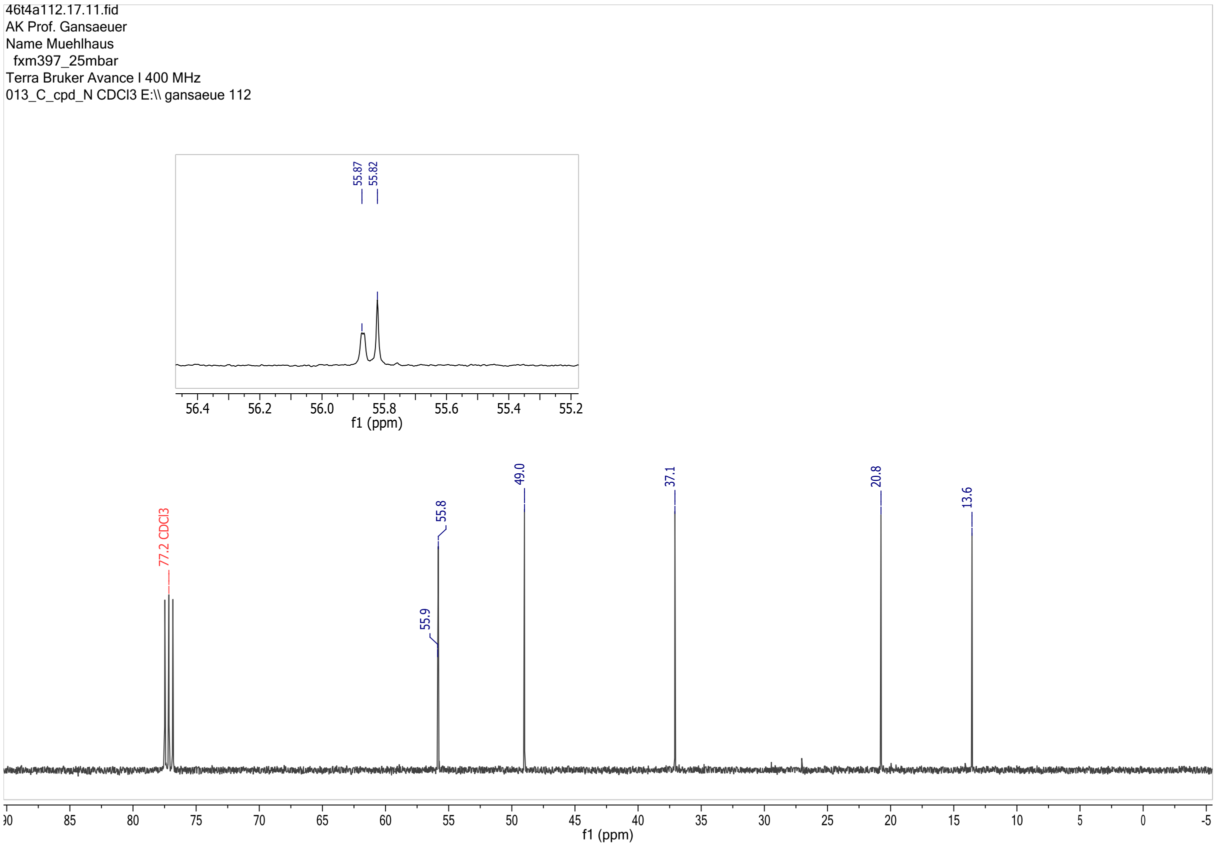
46t4a112.17.11.fid
AK Prof. Gansaeuer
Name Muehlhaus
fxm397_25mbar
Terra Bruker Avance I 400 MHz
013_C_cpd_N CDCl3 E:\\ gansaeue 112
55.87
55.82
77.2 CDCl3
55.9
55.8
49.0
37.1
20.8
13.6
f1 (ppm)

3.1.2. Synthesis of *syn*-3-brom-1,2-epoxy-octane.

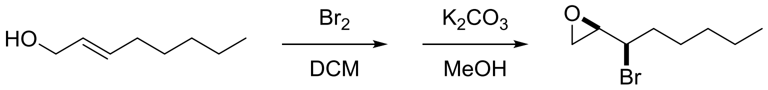

According to GP1: 12.1 g *(E)*-oct-2-en-1-ol (1.00 eq., 120.0 mmol), 19.2 g bromine (1.00 eq., 120.0 mmol, 6.15 mL) are reacted for 30 minutes at -78 °C in DCM. After workup, the crude product is reacted with 33.2 g K_2CO_3 (2.00 eq., 240 mmol) in MeOH. Flash chromatography (SiO_2, Eluent: Ch : Et_2O = 99 : 1) afforded 13.8 g (*syn*)-3-bromo-1,2-epoxyoctane 56 % as a light yellow oil.

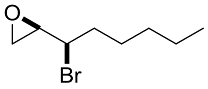

$C_8H_{15}BrO$
207.11 g/mol

R_f = 0.50 (10% Et_2O in CH), **^{1}H-NMR (500 MHz, $CDCl_3$, RT): δ [ppm] =** 0.83-0.92 (m, 3H), 1.22-1-35 (m, 4H), 1.36-1.46 (m, 1H), 1.54 (dddd, 1H, , *J* = 15.4, *J* = 12.3 Hz, *J* = 9.3 Hz, *J* = 4.5 Hz), 1.80-1.92 (m, 2H), 2.72 (dt, 1H, *J* = 4.2, *J* = 2.0 Hz), 2.92-2.97 (m, 1H), 3.19 (ddt, 1H, *J* = 7.8 Hz, *J* = 4.0 Hz, *J* = 1.9j Hz), 3.62-3.68 (m, 1H). **^{13}C-NMR (125 MHz, $CDCl_3$, RT): δ [ppm] =** 14.0, 22.5, 27.1, 31.2, 35.1, 49.0, 55.8, 62.2. **IR ṽ [cm^{-1}] =** 613, 807, 856, 924, 1227, 1251, 1466, 1740, 2860, 2929, 2957.

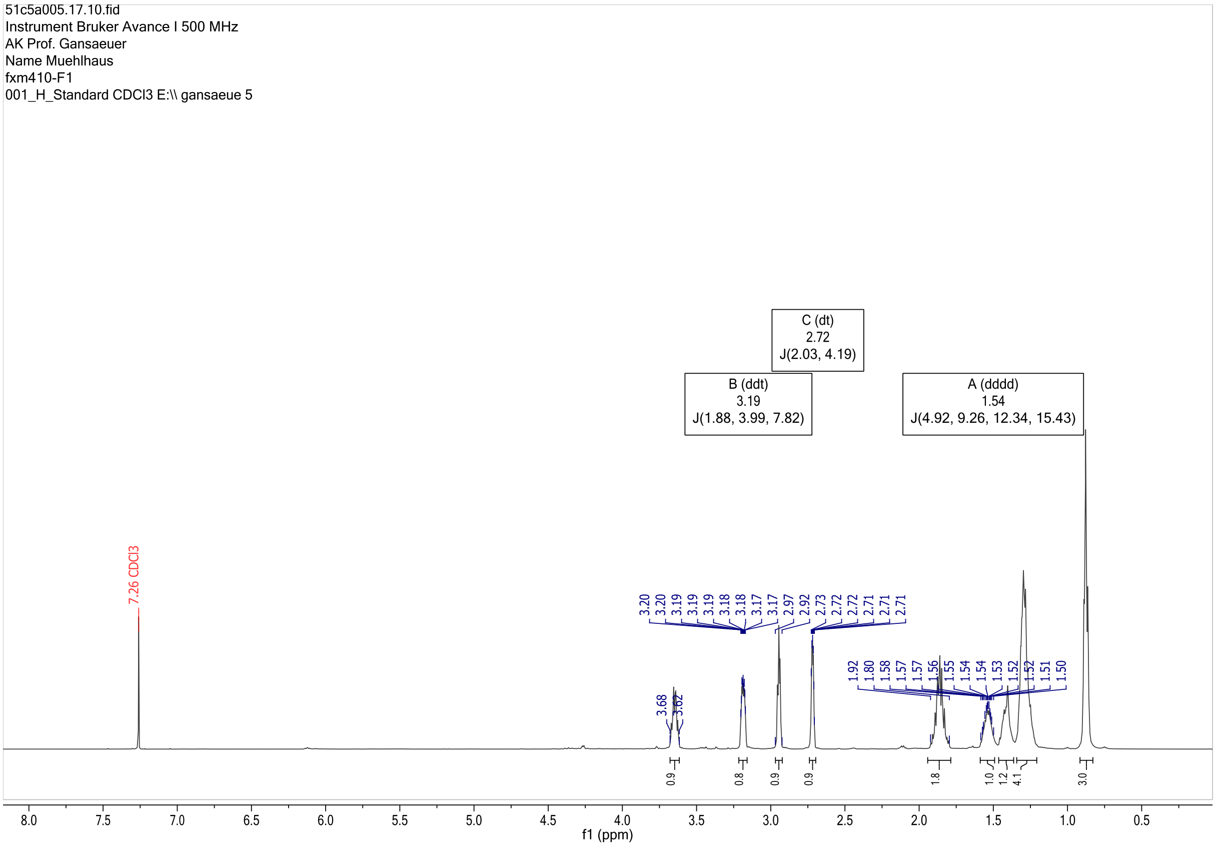
51c5a005.17.10.fid
Instrument Bruker Avance I 500 MHz
AK Prof. Gansaeuer
Name Muehlhaus
fxm410-F1
001_H_Standard CDCl3 E:\\ gansaeue 5
C (dt)
2.72
J(2.03, 4.19)
B (ddt)
3.19
J(1.88, 3.99, 7.82)
A (dddd)
1.54
J(4.92, 9.26, 12.34, 15.43)
7.26 CDCl3
f1 (ppm)

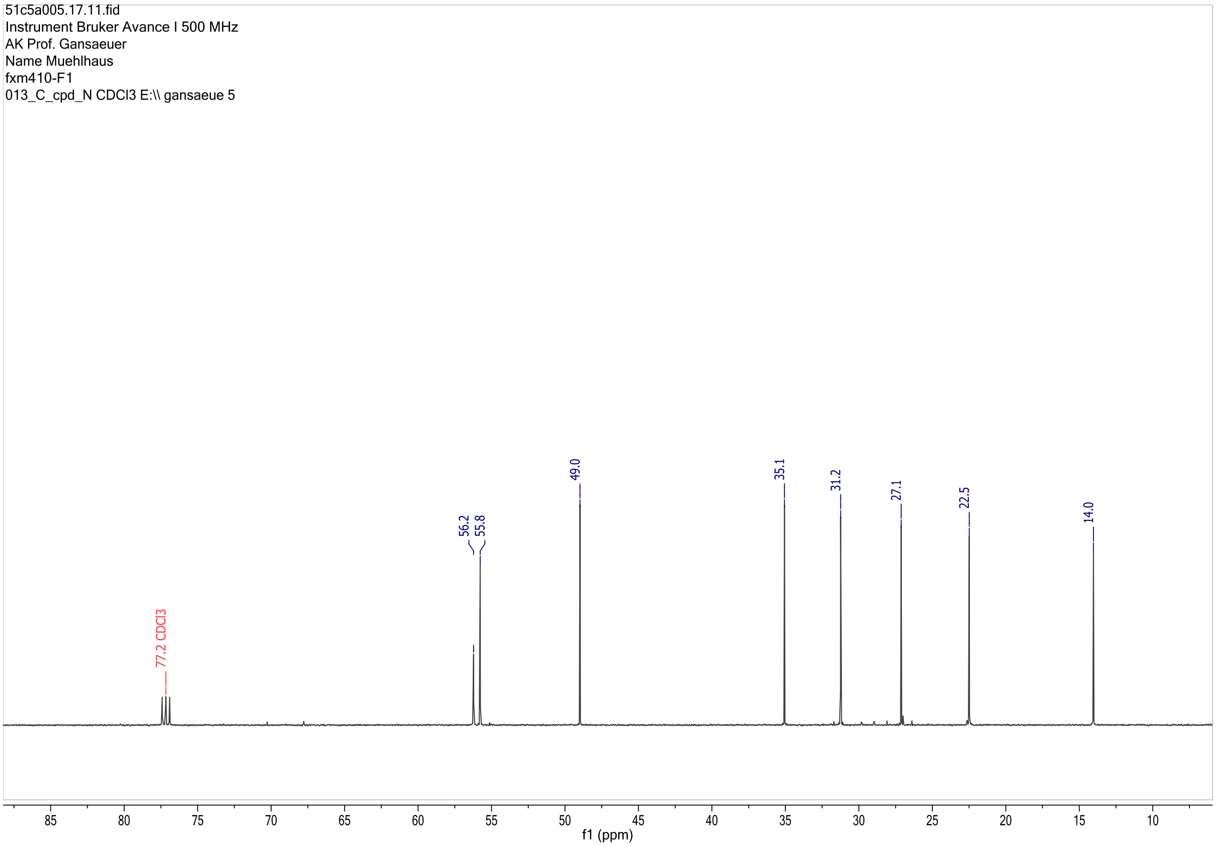
51c5a005.17.11.fid
Instrument Bruker Avance I 500 MHz
AK Prof. Gansaeuer
Name Muehlhaus
fxm410-F1
013_C_cpd_N CDCl3 E:\\ gansaeue 5
77.2 CDCl3
56.2
55.8
49.0
35.1
31.2
27.1
22.5
14.0
f1 (ppm)

3.1.3. Synthesis of *syn*-3-brom-1,2-epoxy-5-methyl-hexane.

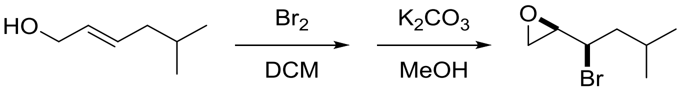

According to GP1: 3.19 g *(E)*-5-methyl-hex-2-en-1-ol (1.00 eq., 31.9 mmol), 5.10 g bromine (1.00 eq., 31.9 mmol, 1.63 mL) are reacted for 30 minutes at -78 °C in DCM. After workup, the crude product is reacted with 8.81 g K_2CO_3 (2.00 eq., 63.8 mmol) in MeOH. Flash chromatography (SiO_2, Eluent: Ch : Et_2O = 99 : 1) afforded 4.45 g (*syn*)-3-bromo-1,2-epoxy-5-methyl-hexane 78 % as a light colorless oil.

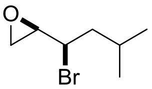

$C_7H_{13}BrO$
193.08 g/mol

R_f = 0.40 (10% Et_2O in CH),**^{1}H-NMR (500 MHz, $CDCl_3$, RT): δ [ppm] =** 1.09 (ddd, 6H, *J* = 11.4 Hz, *J* = 6.7 Hz, *J* = 1.9 Hz,), 2.05 (hept, 1H, *J* = 6.7 Hz), 2.73 (dt, 1H, *J* = 4.5, *J* = 2.1 Hz), 3.01 (ddd, 1H *J* = 6.0 Hz, *J* = 4.1 Hz, *J* = 1.9 Hz,), 3.20 (ddt, 1H, *J* = 8.6 Hz, *J* = 3.7 Hz, *J* = 2.3 Hz), 3.47 (ddd, 1H, *J* = 8.5 Hz, *J* = 5.4 Hz, *J* = 1.7 Hz). **^{13}C-NMR (125 MHz, $CDCl_3$, RT): δ [ppm] =** 19.9, 20.6, 33.5, 50.1, 54.8, 64.8. **IR ṽ [cm^{-1}] =** 407, 471, 678, 692, 808, 819, 838, 857, 921, 1198, 1255, 1369, 1388, 1465, 2967. **HRMS (ESI):** *m/z* berechnet für $C_6H_{11}BrONa^+$: 200.9885 u; found: 200.9877 u.

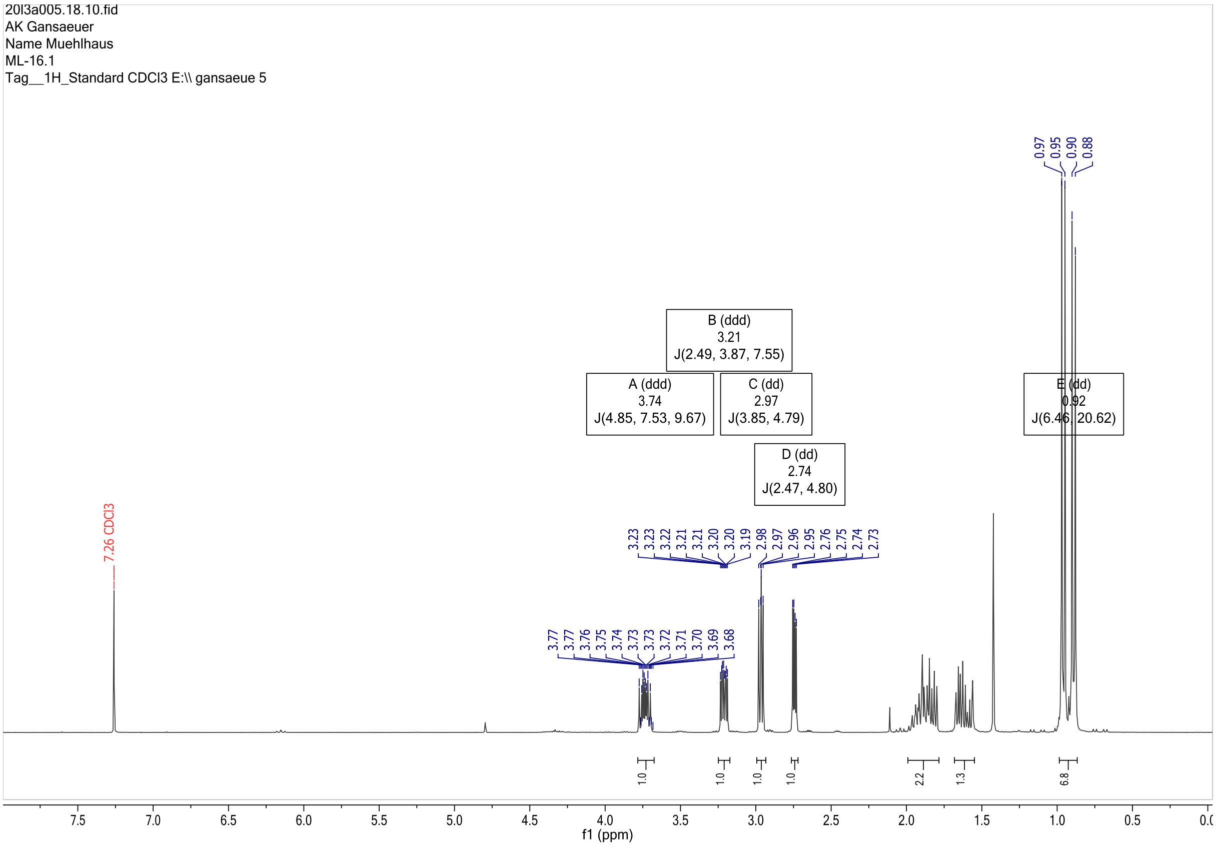
20l3a005.18.10.fid
AK Gansaeuer
Name Muehlhaus
ML-16.1
Tag__1H_Standard CDCl3 E:\\ gansaeue 5
7.26 CDCl3
A (ddd)
3.74
J(4.85, 7.53, 9.67)
B (ddd)
3.21
J(2.49, 3.87, 7.55)
C (dd)
2.97
J(3.85, 4.79)
D (dd)
2.74
J(2.47, 4.80)
E (dd)
0.92
J(6.46, 20.62)
f1 (ppm)

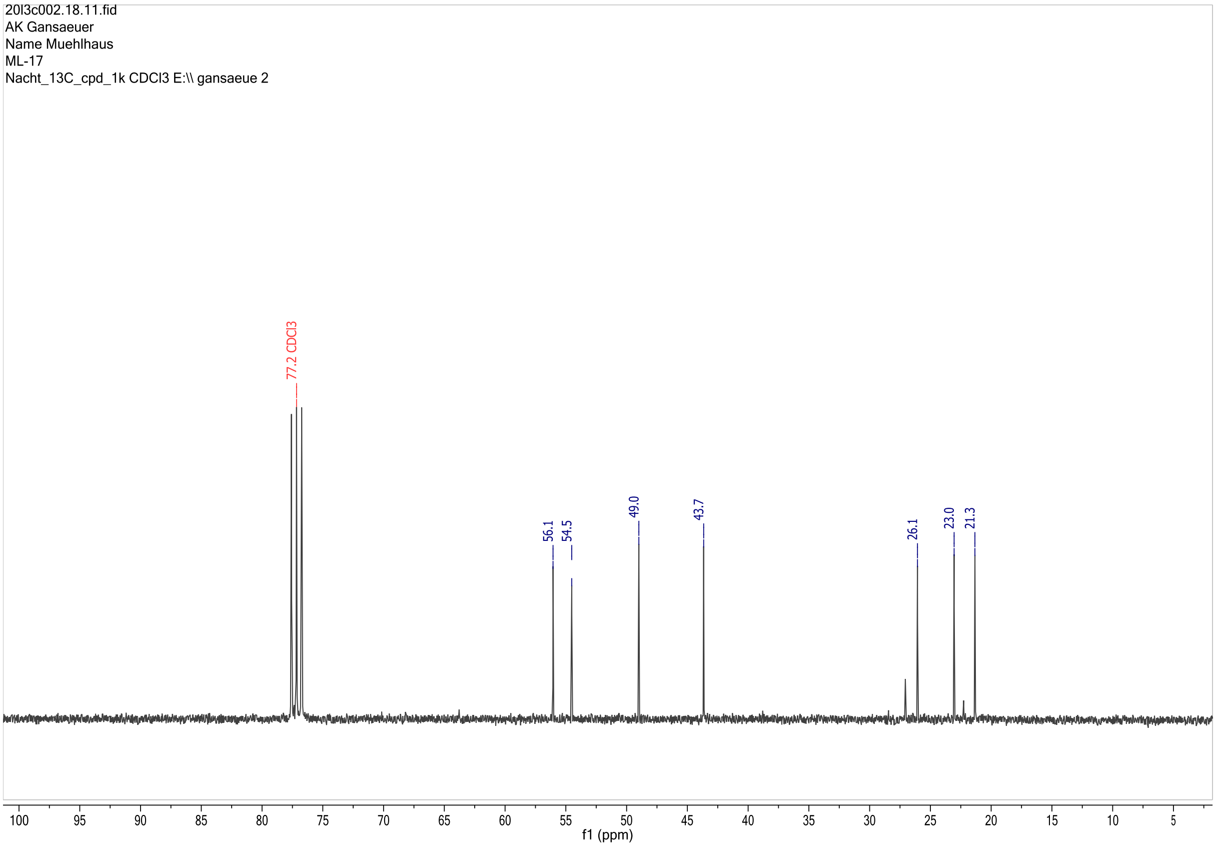
20l3c002.18.11.fid
AK Gansaeuer
Name Muehlhaus
ML-17
Nacht_13C_cpd_1k CDCl3 E:\\ gansaeue 2
77.2 CDCl3
56.1
54.5
49.0
43.7
26.1
23.0
21.3
f1 (ppm)

3.2 Kinetic resolution of α-bromo-epoxides (*rac*-A→A)

3.2.1 Kinetic resolution of *syn*-3-bromo-1,2-epoxy-hexane.

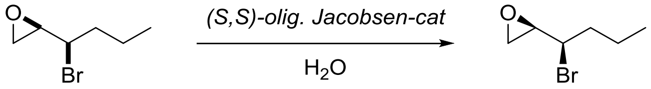

According to GP2 25.8 g of *syn*-3-bromo-1,2-epoxy-hexane (144 mmol, 1.0 eq) are mixed with 10 mg (0.01 mmol, 0.00007 eq.) *(S,S)*-oligomeric Jacobsen catalyst. 1.37 g H_2O (76.3 mmol, 0.53 eq.) is added dropwise and the mixture is stirred for 16h at room temperature. The crude mixture is purified without further workup by flash column chromatography (SiO_2, Eluent: CH : MTBE 98:2). 11.0 g (*2R,3R*)-3-bromo-1,2-epoxy-hexane (61.2 mmol, 43% yield) are isolated.

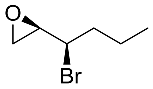

$C_6H_{11}BrO$
179.05 g/mol

R_f = 0.40 (10% Et_2O in CH),**^{1}H-NMR (500 MHz, $CDCl_3$, RT): δ [ppm] =** 0.95 (t, 3H, *J* = 7.4 Hz), 1.36-1.71 (m, 1H), 1.52-1.65 (m, 1H), 1.86 (q, 2H, *J* = 7.5 Hz), 2.74 (dd, 1H, *J* = 4.8, *J* = 2.5 Hz), 2.97 (dd, 1H, *J* = 4.8 Hz, *J* = 3.8), 3.21 (ddd, 1H, *J* = 7.6 Hz, *J* = 3.8 Hz, *J* = 2.4 Hz), 3.65-3.71 (m, 1H). **^{13}C-NMR (125 MHz, $CDCl_3$, RT): δ [ppm] =** 13.6, 20.8, 37.1, 49.0, 58.8, 55.9. **IR ṽ [cm^{-1}] =** 517, 525, 611, 693, 749, 761, 798, 854, 873, 928, 1193, 1252, 1465, 2933, 2961; **$[\alpha]_D^{20}$=** –7.9° (*c* 1.0, $CHCl_3$); **HPLC**: *DAICEL Chiralpak AS-3*; *n*-Heptane/2-Propanol (98:2); flowrate 1.0 mL/min; t_R= 6.1 min (minor, *2S,3S*), t_R= 7.1 min (major, *2R, 3R*); ***er***= >99:<1.

3.2.2 Kinetic resolution of *syn*-3-bromo-1,2-epoxy-octane.

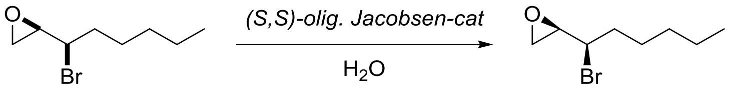

According to GP2 13.6 g of *syn*-3-bromo-1,2-epoxy-octane (65.6 mmol, 1.0 eq) are mixed with 10 mg (0.01 mmol, 0.0002 eq.) *(S,S)*-oligomeric Jacobsen catalyst. 0.65 g H_2O (36.1 mmol, 0.55 eq.) is added dropwise and the mixture is stirred for 16h at room temperature. The crude mixture is purified without further workup by flash column chromatography (SiO_2, Eluent: CH:MTBE 98:2). 4.65 g (*2R,3R*)-3-bromo-1,2-epoxy-octane (36.1 mmol, 35% yield) are isolated.

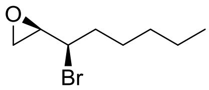

$C_8H_{15}BrO$
207.11 g/mol

R_f = 0.50 (10% Et_2O in CH), **^{1}H-NMR (500 MHz, $CDCl_3$, RT): δ [ppm] =** 0.83-0.92 (m, 3H), 1.22-1-35 (m, 4H), 1.36-1.46 (m, 1H), 1.54 (dddd, 1H, , *J* = 15.4, *J* = 12.3 Hz, *J* = 9.3 Hz, *J* = 4.5 Hz), 1.80-1.92 (m, 2H), 2.72 (dt, 1H, *J* = 4.2, *J* = 2.0 Hz), 2.92-2.97

(m, 1H), 3.19 (ddt, 1H, *J* = 7.8 Hz, *J* = 4.0 Hz, *J* = 1.9j Hz), 3.62-3.68 (m, 1H). **^{13}C-NMR (125 MHz, $CDCl_3$, RT): δ [ppm] =** 14.0, 22.5, 27.1, 31.2, 35.1, 49.0, 55.8, 62.2. **IR ṽ [cm^{-1}] =** 613, 807, 856, 924, 1227, 1251, 1466, 1740, 2860, 2929, 2957; **$[\alpha]_D^{20}$=** −7.6° (*c* 1.0, $CHCl_3$); **HPLC**: *DAICEL Chiralpak AS-3*; *n*-Heptane/2-Propanol (98:2); flowrate 1.0 mL/min; t_R= 4.9 min (minor, *2S,3S*), t_R= 5.5 min (major, *2R, 3R*); ***er***= >99:<1.

3.2.3 Kinetic resolution of (*syn*)-3-bromo-1,2-epoxy-5-methyl-hexane.

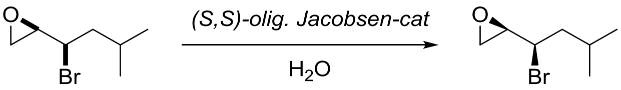

According to GP2 7.54 g of (*syn*)-3-bromo-1,2-epoxy-5-methyl-hexane (39.1 mmol, 1.0 eq) are mixed with 20 mg (0.02 mmol, 0.0004 eq.) *(S,S)*-oligomeric Jacobsen catalyst. 0.373 g H_2O (20.7 mmol, 0.53 eq.) is added dropwise and the mixture is stirred for 16h at room temperature. The crude mixture is purified without further workup by flash column chromatography (SiO_2, Eluent: CH : Et_2O 98:2). 2.62 g (*2R,3R*)-3-bromo-1,2-epoxy-hexane (13.6 mmol, 35% yield) are isolated.

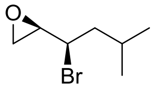

$C_7H_{13}BrO$
193.08 g/mol

R_f = 0.40 (10% Et_2O in CH),**^{1}H-NMR (500 MHz, $CDCl_3$, RT): δ [ppm] =** 1.09 (ddd, 6H, *J* = 11.4 Hz, *J* = 6.7 Hz, *J* = 1.9 Hz,), 2.05 (hept, 1H, *J* = 6.7 Hz), 2.73 (dt, 1H, *J* = 4.5, *J* = 2.1 Hz), 3.01 (ddd, 1H *J* = 6.0 Hz, *J* = 4.1 Hz, *J* = 1.9 Hz,), 3.20 (ddt, 1H, *J* = 8.6 Hz, *J* = 3.7 Hz, *J* = 2.3 Hz), 3.47 (ddd, 1H, *J* = 8.5 Hz, *J* = 5.4 Hz, *J* = 1.7 Hz). **^{13}C-NMR (125 MHz, $CDCl_3$, RT): δ [ppm] =** 19.9, 20.6, 33.5, 50.1, 54.8, 64.8. **IR ṽ [cm^{-1}] =** 407, 471, 678, 692, 808, 819, 838, 857, 921, 1198, 1255, 1369, 1388, 1465, 2967. **HRMS (ESI):** *m/z* calculated for $C_7H_{14}BrO^+$: 193.0223 u; found: 193.0227 u. **$[\alpha]_D^{20}$=** -8.0° (*c* 1.0, $CHCl_3$); **HPLC**: *DAICEL Chiralpak IC-U*; *n*-Heptane/2-Propanol (98:2); flowrate 0.85 mL/min; t_R= 1.4 min (minor, *2S,3S*), t_R= 1.7 min (major, *2R, 3R*); ***er***= >99:<1.

3.3 Base mediated aminolysis of bromo-epoxides to aniline-epoxides (A→1)

3.3.1. Synthesis of *N*-(((*2R,3S*)-3-pentyloxiran-2-yl)methyl)-N-phenylaniline (1b).

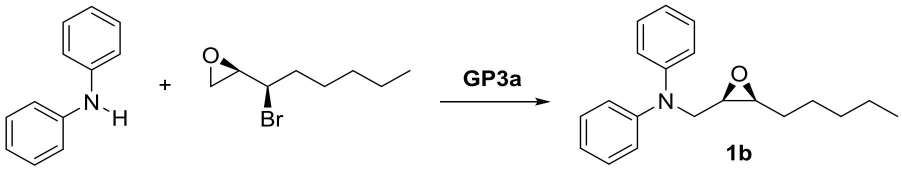

According to GP3a: 1.53 g diphenylamine (1.00 eq., 8.0 mmol), 2.49 g (2*R*,3*R*)-3-bromo-1,2-epoxyoctane (1.50 eq., 12.0 mmol) and 3.52 mL *n*Butylithium (1.1 eq., 8.8 mmol, 2.5 M in hexane) are reacted for 20 hours (-78 °C to RT) in dry THF. Remaining (2*R*,3*R*)-3-bromo-1,2-epoxyoctane was removed under reduced pressure (5 mbar, 80 °C). Flash chromatography (SiO_2, Eluent: Ch : Et_2O = 98 : 2) yielded 1.89 g **1b** 80 % as a colorless liquid.

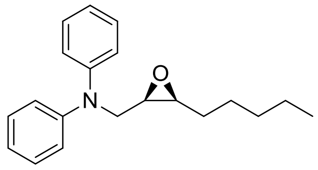

1b
$C_{20}H_{25}NO$
295.42 g/mol

R_f = 0.8 (20% Et_2O in CH), **^{1}H-NMR (500 MHz, C_6D_6, RT): δ [ppm] =** 0.84 (t, 3H, *J* = 7.1 Hz), 1.08-1.33 (m, 8H), 2.61 (dt, 1H, *J* = 6.8 Hz, *J* = 4.3 Hz), 3.06 (ddd, 1H, *J* = 6.8 Hz, *J* = 4.3 Hz, *J* = 4.3 Hz), 3.64 (dd, 1H, *J* = 15.7 Hz, *J* = 5.5 Hz), 3.76 (dd, 1H, *J* = 15.8 Hz, *J* = 4.3 Hz), 6.85 (tt, 2H, *J* = 7.3 Hz, *J* = 1.3 Hz), 7.06 (dd, 4H, *J* = 8.4 Hz, *J* = 1.4 Hz), 7.12 (tt, 4H, *J* = 8.6 Hz, *J* = 7.1 Hz). **^{13}C-NMR (125 MHz, C_6D_6, RT): δ [ppm] =** 14.2, 22.9, 26.7, 28.4, 31.9, 51.5, 54.6, 56.8, 121.7, 122.0, 129.7, 148.5. **IR ṽ [cm^{-1}] =** 513, 593, 607, 692, 730, 746, 1222, 1249, 1362, 1460, 1494, 1589, 2926, 2955. **HRMS (ESI):** *m/z* calculated for $C_{20}H_{26}NO^+$: 296.2009 u, found: 296.2014 u; **$[\alpha]_D^{20}$=** –80.4° (*c* 1.0, $CHCl_3$)

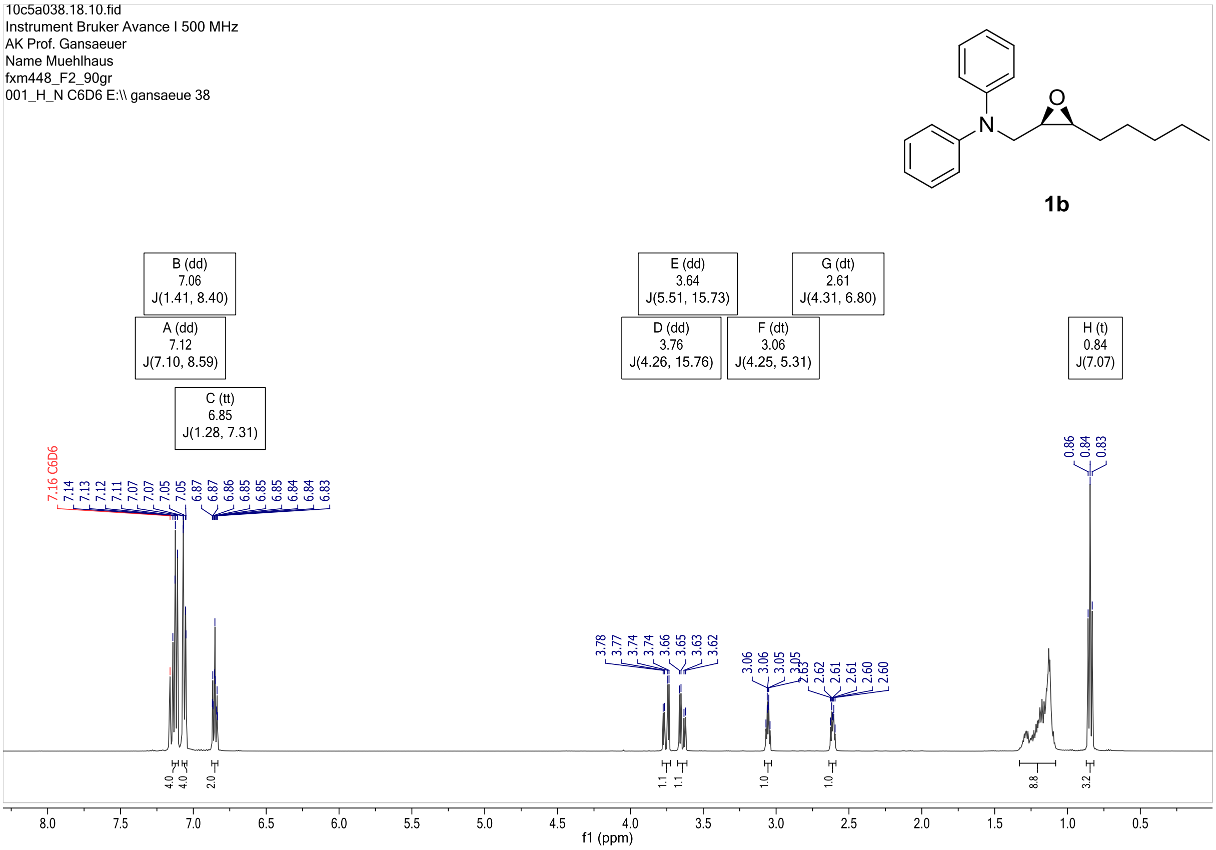
10c5a038.18.10.fid
Instrument Bruker Avance I 500 MHz
AK Prof. Gansaeuer
Name Muehlhaus
fxm448_F2_90gr
001_H_N C6D6 E:\\ gansaeue 38
N
O
1b
B (dd) 7.06 J(1.41, 8.40)
A (dd) 7.12 J(7.10, 8.59)
C (tt) 6.85 J(1.28, 7.31)
E (dd) 3.64 J(5.51, 15.73)
D (dd) 3.76 J(4.26, 15.76)
F (dt) 3.06 J(4.25, 5.31)
G (dt) 2.61 J(4.31, 6.80)
H (t) 0.84 J(7.07)
7.16 C6D6
7.14 7.13 7.12 7.11 7.07 7.07 7.05 7.05 6.87 6.87 6.86 6.85 6.85 6.85 6.84 6.84 6.83
3.78 3.77 3.74 3.74 3.66 3.65 3.63 3.62
3.06 3.06 3.05 3.05 2.63 2.62 2.61 2.61 2.60 2.60
0.86 0.84 0.83
4.0 4.0 2.0 1.1 1.1 1.0 1.0 8.8 3.2
f1 (ppm)

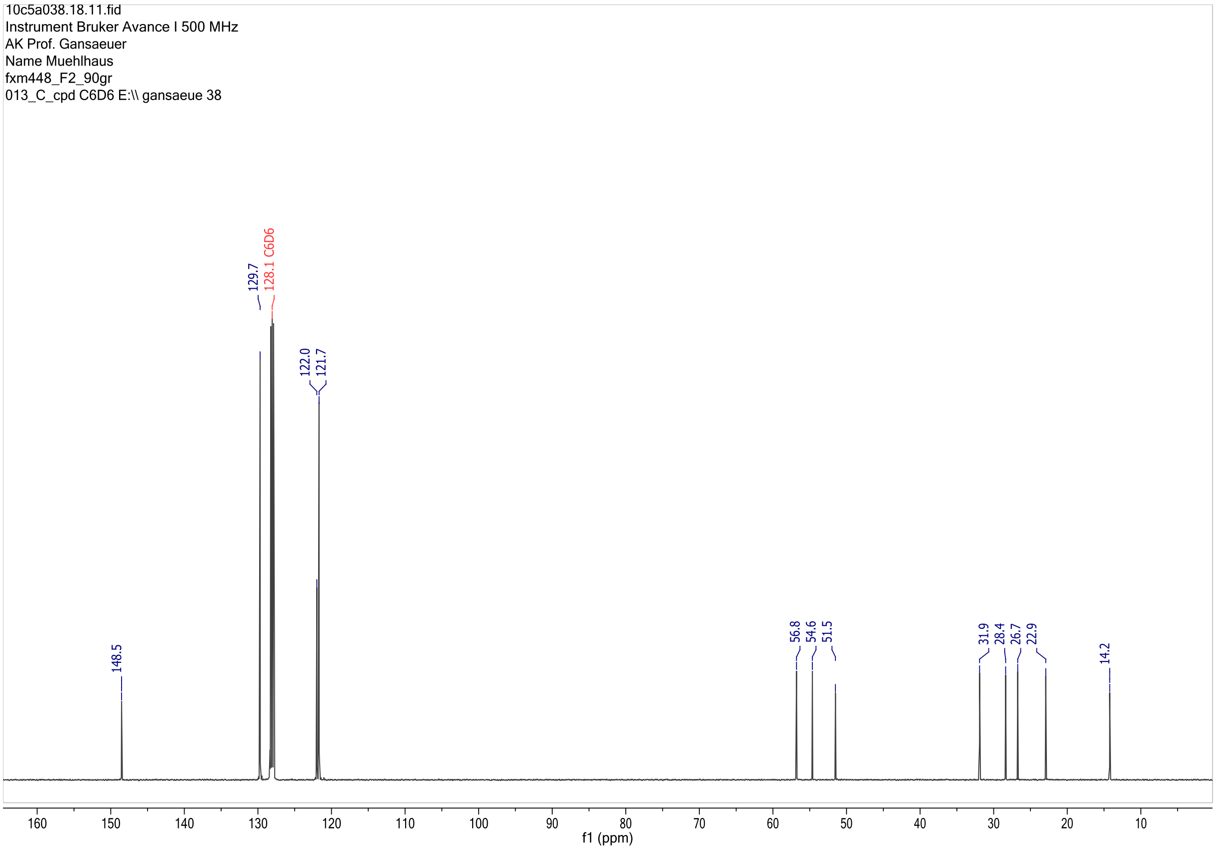
10c5a038.18.11.fid
Instrument Bruker Avance I 500 MHz
AK Prof. Gansaeuer
Name Muehlhaus
fxm448_F2_90gr
013_C_cpd C6D6 E:\\ gansaeue 38
129.7
128.1 C6D6
122.0 121.7
148.5
56.8 54.6 51.5
31.9 28.4 26.7 22.9
14.2
f1 (ppm)

3.3.2. Synthesis of *N*-(((*2R,3S*)-3-isobutyloxiran-2-yl)methyl)-N-phenylaniline (1c).

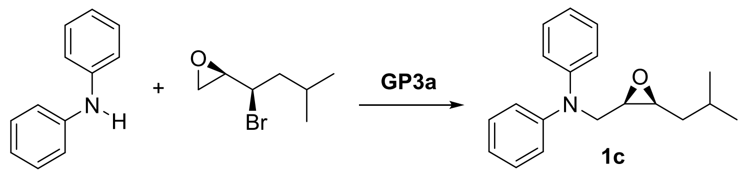

According to GP3a: 846 mg diphenylamine (1.00 eq., 6.0 mmol), 1.16 g (2*R*,3*R*)-3-bromo-1,2-epoxy-5-methyl-hexane (1.20 eq., 6.0 mmol) and 2.2 mL *n*Butylithium (1.1 eq., 5.5 mmol, 2.5 M in hexane) are reacted for 20 hours (-78 °C to RT) in dry THF. Flash chromatography (SiO_2, Eluent: Ch : Et_2O = 99 : 1) yielded 693 mg (*N*,*N*-Diphenyl)(2*R*,3*R*)-3-bromo-5-methyl-hexanmine- **1c** 49 % as a colorless, viscous liquid.

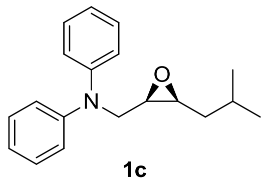

1c
$C_{19}H_{23}NO$
281.39 g/mol

R_f = 0.8 (20% Et_2O in CH), **^{1}H-NMR (500 MHz, C_6D_6, RT): δ [ppm] =** 0.78 (d, 3H, *J* = 6.7 Hz), 0.86 (d, 3H, *J* = 6.7 Hz), 0.98-1.15 (m, 2H), 1.55 (hept, 1H, *J* = 6.7 Hz), 2.63 (dt, 1H, *J* = 7.1 Hz, *J* = 4.6 Hz), 3.03 (ddd, 1H, *J* = 5.8 Hz, *J* = 4.0 Hz, *J* = 4.0 Hz), 3.59 (dd, 1H, *J* = 15.7 Hz, *J* = 5.8 Hz), 3.77 (dd, 1H, *J* = 15.7 Hz, *J* = 3.9 Hz), 6.85 (tt, 2H, *J* = 7.3 Hz, *J* = 1.3 Hz), 7.05-7.14 (m, 8H). **^{13}C-NMR (125 MHz, C_6D_6, RT): δ [ppm] =** 22.6, 22.8, 27.1, 37.1, 51.8, 54.3, 55.6, 121.7, 122.0, 129.7, 148.6. **IR ṽ [cm^{-1}] =** 513, 608, 692, 729, 746, 1248, 1362, 1463, 1493, 1589, 2955. **HRMS (ESI):** *m/z* calculated for $C_{19}H_{24}NO^+$: 282.1852 u, found: 282.1857 u. **$[\alpha]_D^{20}$=** – 68.0 (*c* 1.0, $CHCl_3$).

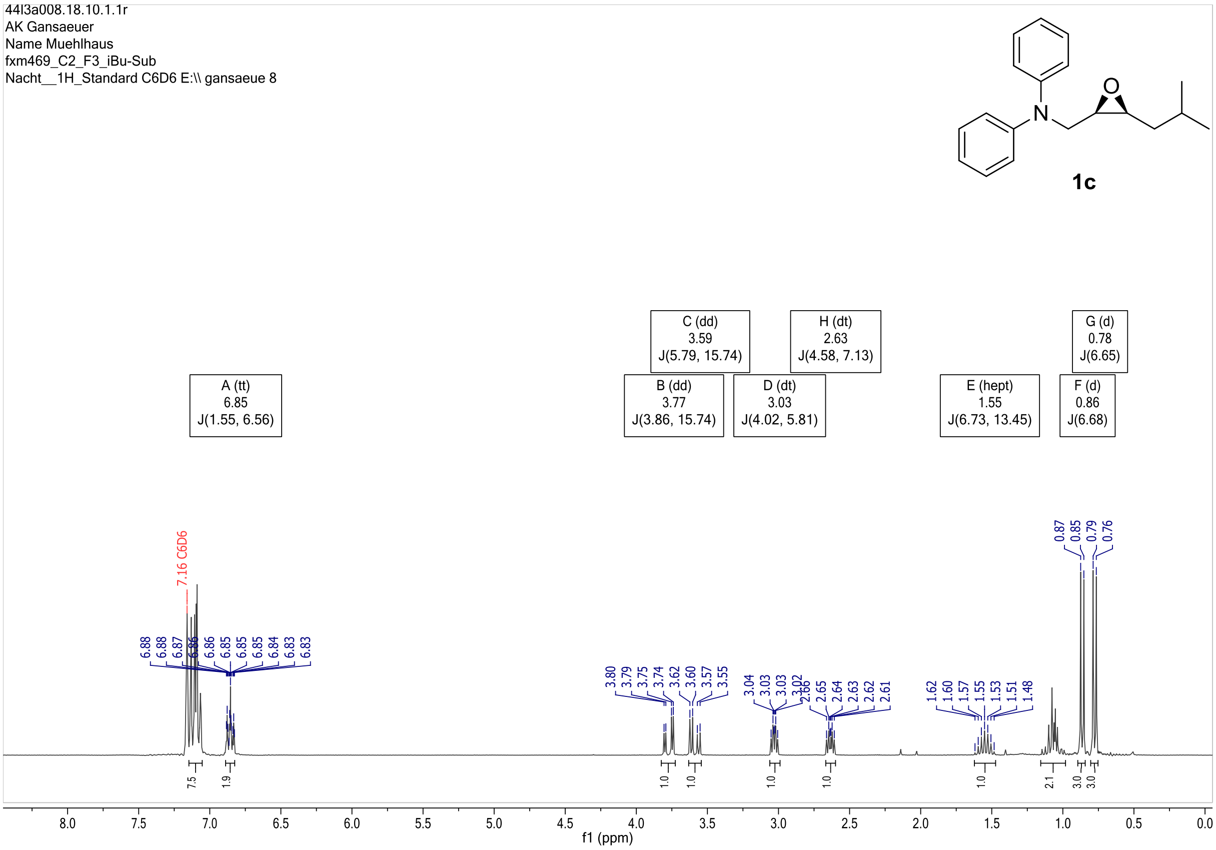
44l3a008.18.10.1.1r
AK Gansaeuer
Name Muehlhaus
fxm469_C2_F3_iBu-Sub
Nacht__1H_Standard C6D6 E:\\ gansaeue 8
N
O
1c
A (tt) 6.85 J(1.55, 6.56)
B (dd) 3.77 J(3.86, 15.74)
C (dd) 3.59 J(5.79, 15.74)
D (dt) 3.03 J(4.02, 5.81)
H (dt) 2.63 J(4.58, 7.13)
E (hept) 1.55 J(6.73, 13.45)
F (d) 0.86 J(6.68)
G (d) 0.78 J(6.65)
7.16 C6D6
f1 (ppm)

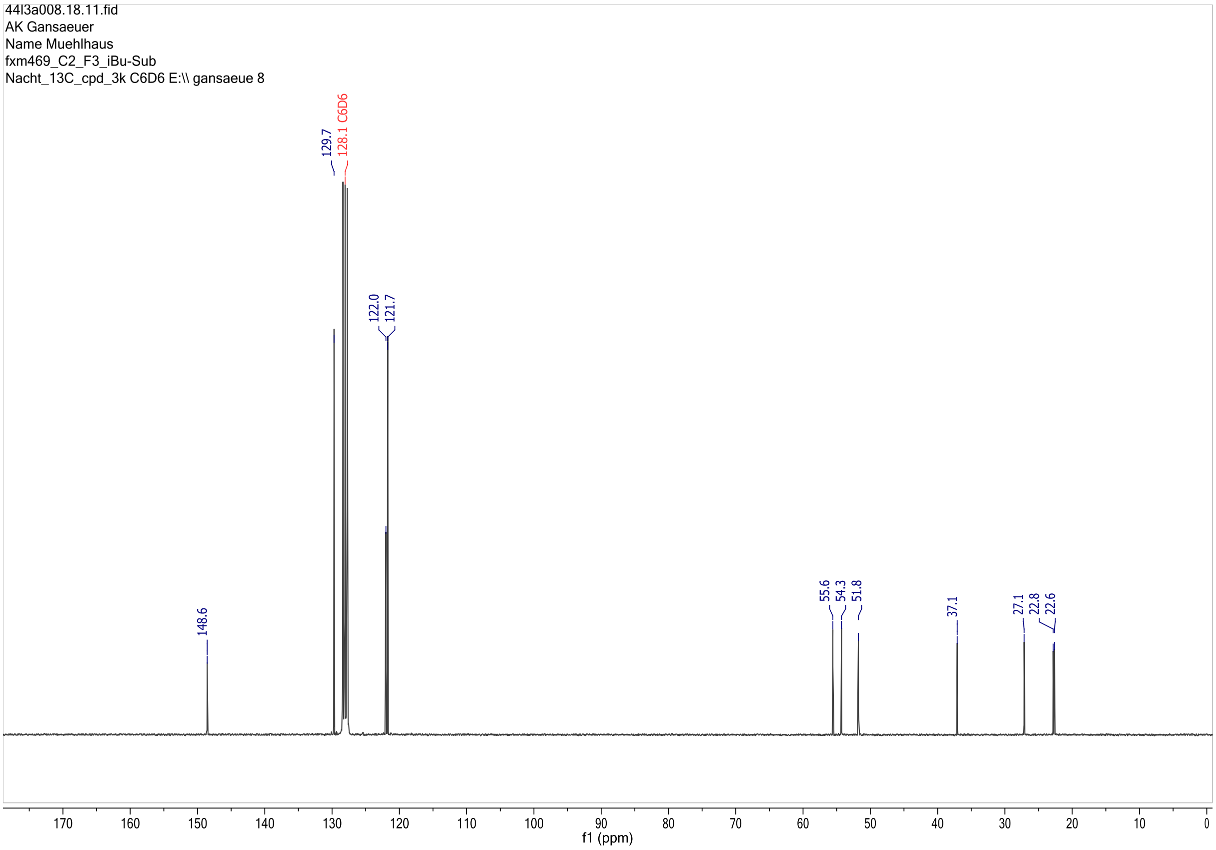
44l3a008.18.11.fid
AK Gansaeuer
Name Muehlhaus
fxm469_C2_F3_iBu-Sub
Nacht_13C_cpd_3k C6D6 E:\\ gansaeue 8
148.6
129.7
128.1 C6D6
122.0
121.7
55.6
54.3
51.8
37.1
27.1
22.8
22.6
f1 (ppm)

3.3.3. Synthesis of *N*-isopropyl-*N*-(((*2R,3S*)-3-propyloxiran-2-yl)methyl)aniline (1e).

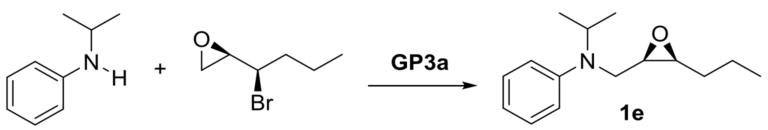

According to GP3a: 1.53 g *N*-isopropylaniline (1.00 eq., 5.0 mmol), 2.49 g (2*R*,3*R*)-3-bromo-1,2-epoxyhexane (1.20 eq., 6.0 mmol) and 2.2 mL *n*Butylithium (1.1 eq., 5.5 mmol, 2.5 M in hexane) are reacted for 20 hours (-78 °C to RT) in dry THF. Flash chromatography (Al_2O_3, Eluent: Ch : Et_2O = 95 : 5) yielded 540 mg **1e** 46% as a colorless liquid.

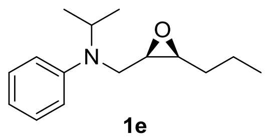

1e
$C_{15}H_{23}NO$
233.35 g/mol

R_f = 0.35 (10% Et_2O in CH), **^{1}H-NMR (500 MHz, C_6D_6, RT): δ [ppm] =** 0.86 (t, 3H, *J* = 7.2 Hz), 0.94 (d, 3H, *J* = 6.7 Hz), 1.04 (d, 3H, *J* = 6.6 Hz), 1.25-1.45 (m, 4H), 2.65 (ddd, 1H, *J* = 6.5 Hz, *J* = 5.4 Hz, *J* = 4.1 Hz), 2.86 (ddd, 1H, *J* = 5.0 Hz, *J* = 4.1 Hz, *J* = 3.1 Hz), 3.07 (dd, 1H, *J* = 16.1 Hz, *J*= 5.1 Hz), 3.33 (dd, 1H, *J* = 16.1 Hz, *J*= 3.1 Hz), 3.84 (hept, 1H, *J* = 6.6 Hz), 6.80 (tt, 1H, *J* = 7.3 Hz, *J* = 1.0 Hz), 6.88 (dd, 2H, *J* = 8.9 Hz, *J* = 1.0 Hz), 7.26 (dd, 2H, *J* = 8.9 Hz, *J* = 7.2 Hz. **^{13}C-NMR (125 MHz, C_6D_6, RT): δ [ppm] =** 14.1, 19.9, 20.4, 20.6, 30.7, 43.5, 48.5, 56.6, 56.7, 114.3, 117.5, 129.7, 149.5. **IR ṽ [cm^{-1}] =** 475, 491, 745, 827, 988, 1041, 1104, 1122, 1159, 1159, 1248, 1297, 1350, 1364, 1392, 1464, 1504, 1598, 2963. **HRMS (ESI):** *m/z* calculated for $C_{15}H_{24}NO^+$: 234.1852 u, found: 234.1852 u; **$[\alpha]_D^{20}$=** –18.3° (*c* 1.0, $CHCl_3$)

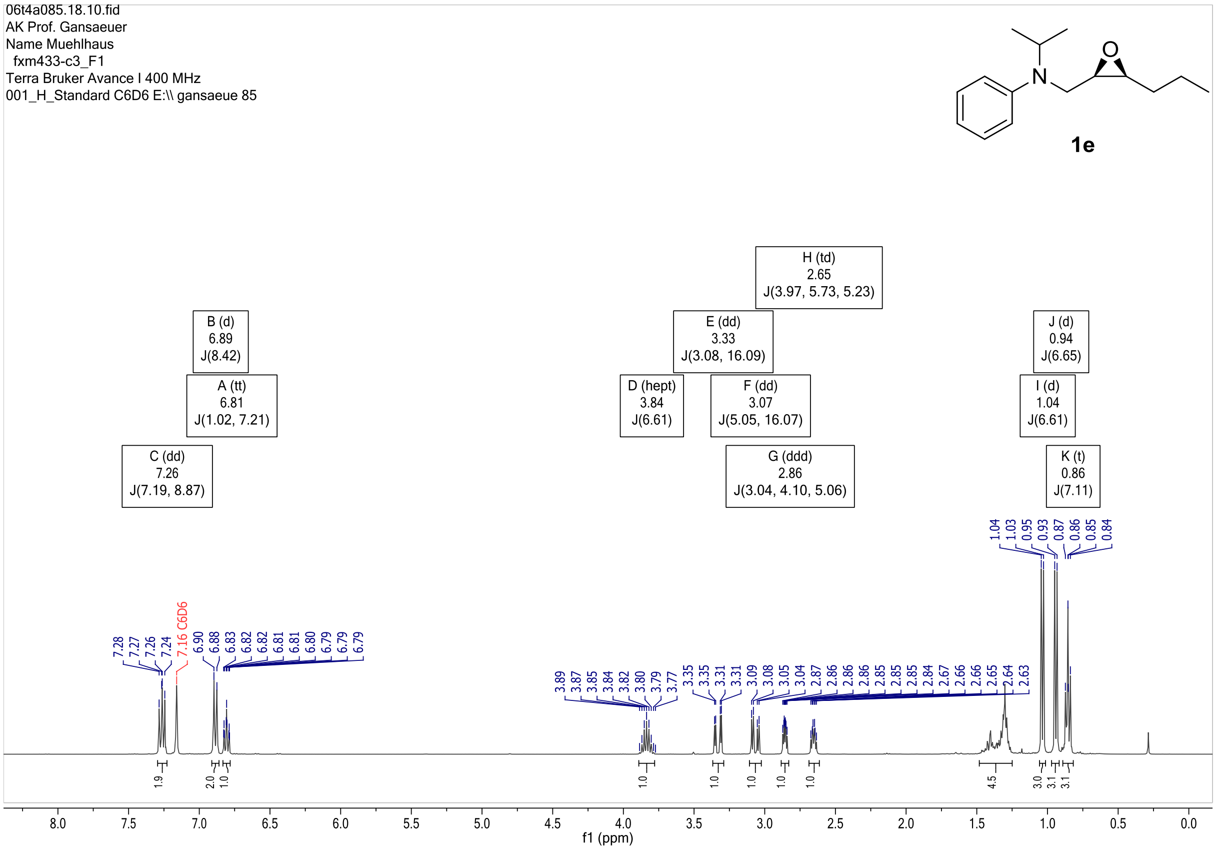
06t4a085.18.10.fid
AK Prof. Gansaeuer
Name Muehlhaus
fxm433-c3_F1
Terra Bruker Avance I 400 MHz
001_H_Standard C6D6 E:\\ gansaeue 85
1e
H (td) 2.65 J(3.97, 5.73, 5.23)
B (d) 6.89 J(8.42)
E (dd) 3.33 J(3.08, 16.09)
J (d) 0.94 J(6.65)
A (tt) 6.81 J(1.02, 7.21)
D (hept) 3.84 J(6.61)
F (dd) 3.07 J(5.05, 16.07)
I (d) 1.04 J(6.61)
C (dd) 7.26 J(7.19, 8.87)
G (ddd) 2.86 J(3.04, 4.10, 5.06)
K (t) 0.86 J(7.11)
7.16 C6D6
f1 (ppm)

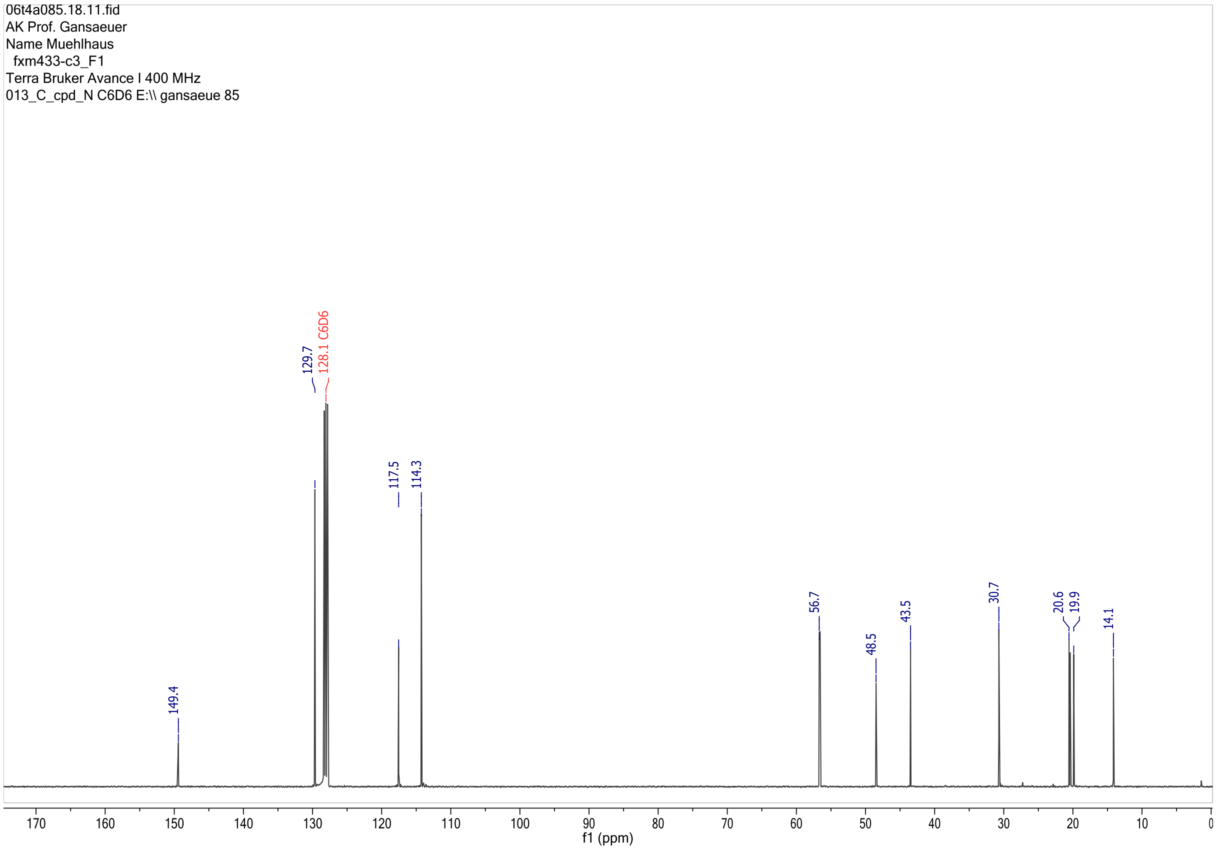
06t4a085.18.11.fid
AK Prof. Gansaeuer
Name Muehlhaus
fxm433-c3_F1
Terra Bruker Avance I 400 MHz
013_C_cpd_N C6D6 E:\\ gansaeue 85
149.4
129.7
128.1 C6D6
117.5
114.3
56.7
48.5
43.5
30.7
20.6
19.9
14.1
f1 (ppm)

3.3.4 Synthesis of *N*-Cyclopropyl-*N*-(((2R,3S)-3-propyloxirane-2-yl)methyl)aniline (1f).

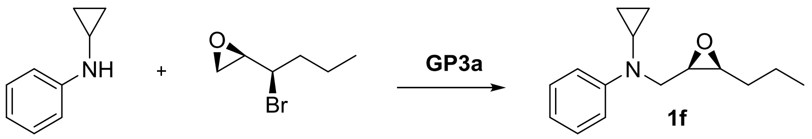

1.04 g (7.8 mmol, 1.0 eq.) of *N*-cyclopropylaniline are reacted with 3.4 mL of 2.5M *n*BuLi-solution in Hexane (8.6 mmol, 1.1 eq.) and 1.95 g (2*R*,3*R*)-3-bromo-1,2-epoxyhexane (10.9 mmol, 1.2 eq.) following GP3a. After flash column chromatography (SiO_2, CH : MTBE 98:2) the product is obtained as a colorless oil in 63% yield (1.14 g, 5.5 mmol) and is stored under argon atmosphere.

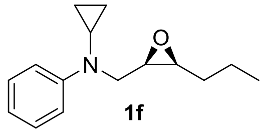

1f

$C_{15}H_{21}NO$

231,34 g/mol

R_f= 0.7 (20% EE in CH)**; ^{1}H NMR (300.1 MHz, C_6D_6, RT) δ [ppm**0.42-0.60 (m, 4H), 0.81-0,89 (m, 3H), 1.22-1.48 (m, 4H), 2.28 (tt, *J*= 6.2 Hz, *J*= 4.0 Hz, 1H), 2.56-2.65 (m, 1H), 2.89 (dt, *J*= 5.8 Hz, *J*= 4.2 Hz, 1H), 3.30 (dd, *J*= 15.5 Hz, *J*= 5.6 Hz, 1H), 3.43 (dd, *J*= 15.5 Hz, *J*= 4.2 Hz, 1H), 6.86 (tt, *J*= 7.2 Hz, *J*= 1.1 Hz, 1H), 7.06-7.13 (m, 2H), 7.23-7.33 (m, 2H); **^{13}C NMR (75 MHz, C_6D_6, RT) δ [ppm]**= 9.6, 9.6, 14.1, 20.5, 30.5, 32.6, 51.3, 54.8, 56.3, 115.1, 118.5, 129.3, 150.2; **IR ν_{max} (neat)[cm^{-1}]**= 691, 719, 748, 824, 1024, 1231, 1300, 1338, 1366, 1452, 1498, 1598, 2958; **HRMS (ESI) m/z** calculated for $[M+H]^+$ 232.1696 u found 232.1697 u; **α_D^{20}($CHCl_3$)=** -18.2°.

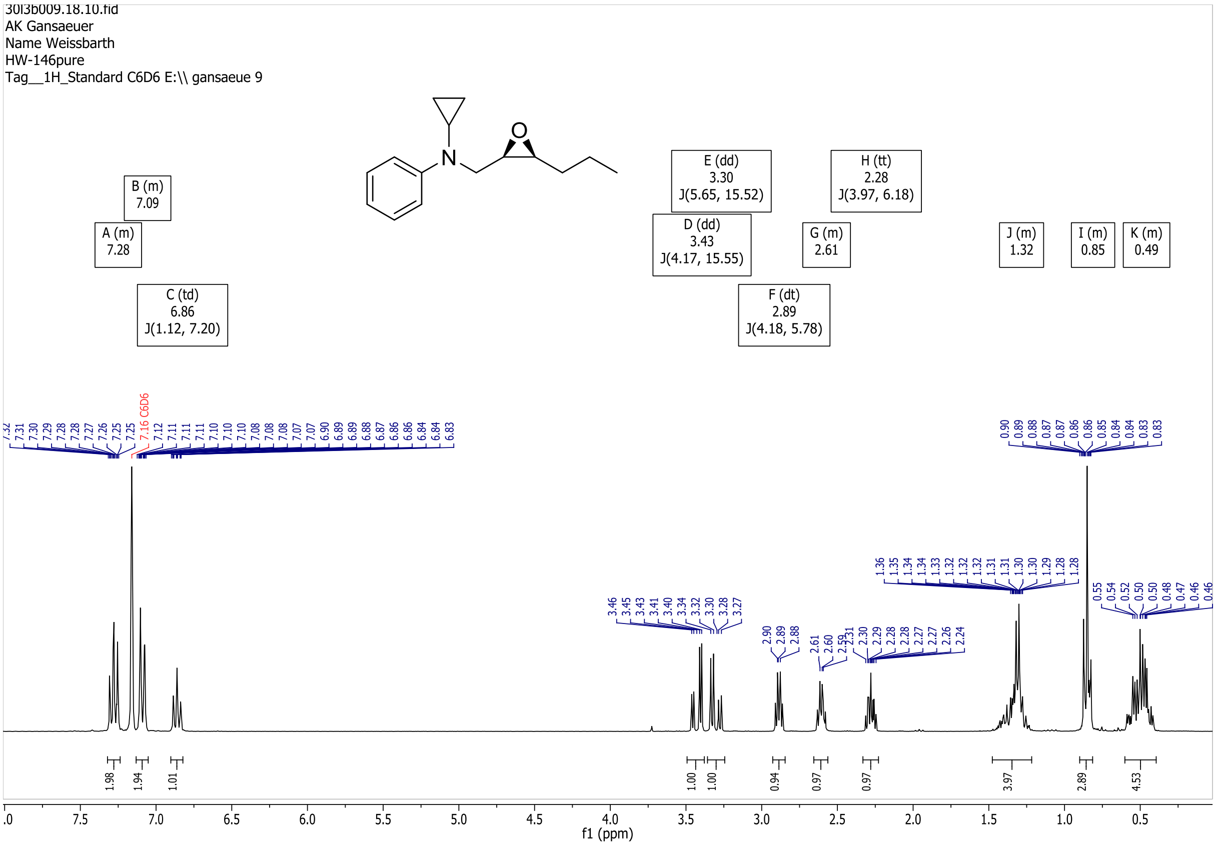
30l3b009.18.10.fid
AK Gansaeuer
Name Weissbarth
HW-146pure
Tag__1H_Standard C6D6 E:\\ gansaeue 9
A (m) 7.28
B (m) 7.09
C (td) 6.86 J(1.12, 7.20)
D (dd) 3.43 J(4.17, 15.55)
E (dd) 3.30 J(5.65, 15.52)
F (dt) 2.89 J(4.18, 5.78)
G (m) 2.61
H (tt) 2.28 J(3.97, 6.18)
J (m) 1.32
I (m) 0.85
K (m) 0.49
7.16 C6D6
f1 (ppm)

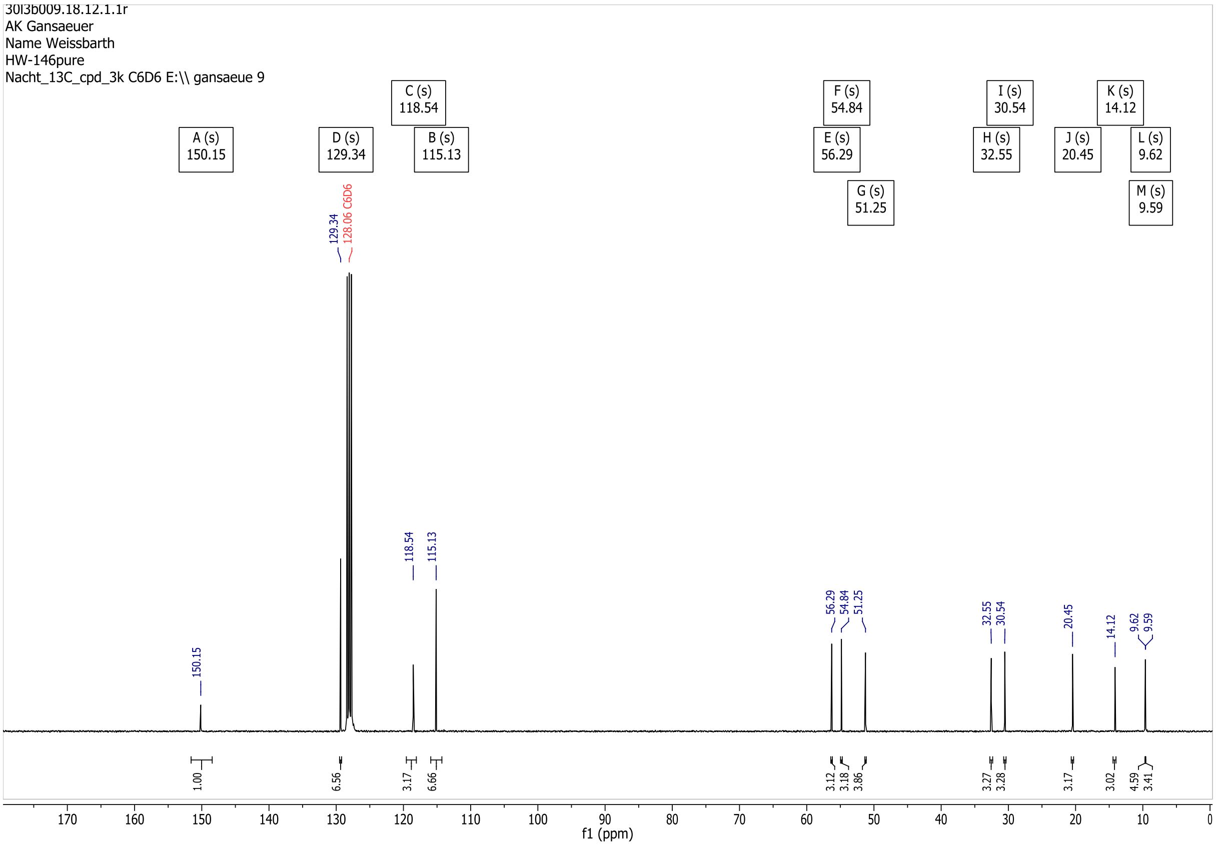
30l3b009.18.12.1.1r
AK Gansaeuer
Name Weissbarth
HW-146pure
Nacht_13C_cpd_3k C6D6 E:\\ gansaeue 9
A (s) 150.15
D (s) 129.34
C (s) 118.54
B (s) 115.13
F (s) 54.84
E (s) 56.29
G (s) 51.25
I (s) 30.54
H (s) 32.55
J (s) 20.45
K (s) 14.12
L (s) 9.62
M (s) 9.59
128.06 C6D6
f1 (ppm)

3.3.5 Synthesis of 4-chloro-*N*-cyclohexyl-*N*-(((2R,3S)-3-propyloxirane-2-yl)methyl)aniline (1h).

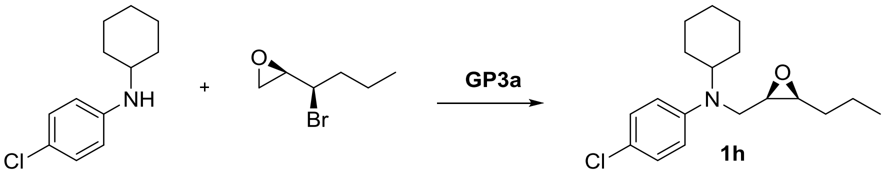

1.01 g (4.8 mmol, 1.0 eq.) of 4-Chloro-*N*-cyclohexylaniline are reacted with 3.6 mL of 1.6M *n*BuLi-solution in Hexane (5.8 mmol, 1.2 eq.) and 1.04 g (2*R*,3*R*)-3-bromo-1,2-epoxyhexane (5.8 mmol, 1.2 eq.) following GP3a. After flash column chromatography (Al_2O_3, CH : MTBE 98:2) the product is obtained as a colorless oil in 53% yield (786 mg, 2.55 mmol) and is stored under argon atmosphere.

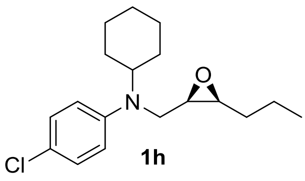

R_f= 0.7 (20% EE in CH); **^{1}H NMR (500.1 MHz, C_6D_6, RT) δ [ppm]**= 0.84-0.97 (m, 4H), 1.04-1.51 (m, 9H), 1.56-1.64 (m, 3H), 1.70-1.77 (m, 1H), 2.66 (ddd, *J*= 6.7 Hz, *J*= 5.1 Hz, *J*= 4.1 Hz, 1H), 2.75 (ddd, *J*= 5.3 Hz, *J*= 4.1 Hz, *J*= 2.9 Hz, 1H), 3.01 (dd, *J*= 16.2 Hz, *J*= 5.3 Hz, 1H), 3.26-3.33 (m, 2H), 6.66-6.71 (m, 2H), 7.20-7.25 (m, 2H); **^{13}C NMR (125.5 MHz, C_6D_6, RT) δ [ppm]**=14.1, 20.6, 26.1, 26.2, 26.4, 30.6, 30.7, 31.4, 44.5, 56.5, 56.6, 57.8, 115.2, 122.1, 129.5, 147.9; **IR ν_{max} (neat)[cm^{-1}]**= 511, 669, 770, 806, 1006, 1100, 1147, 1172, 1234, 1283, 1450, 1496, 1593, 2855, 2930; **HRMS (ESI) m/z** calculated for $[M+H]^+$ 308.1776 u found 308.1773 u; **α_D^{20}($CHCl_3$)=** -23.8°

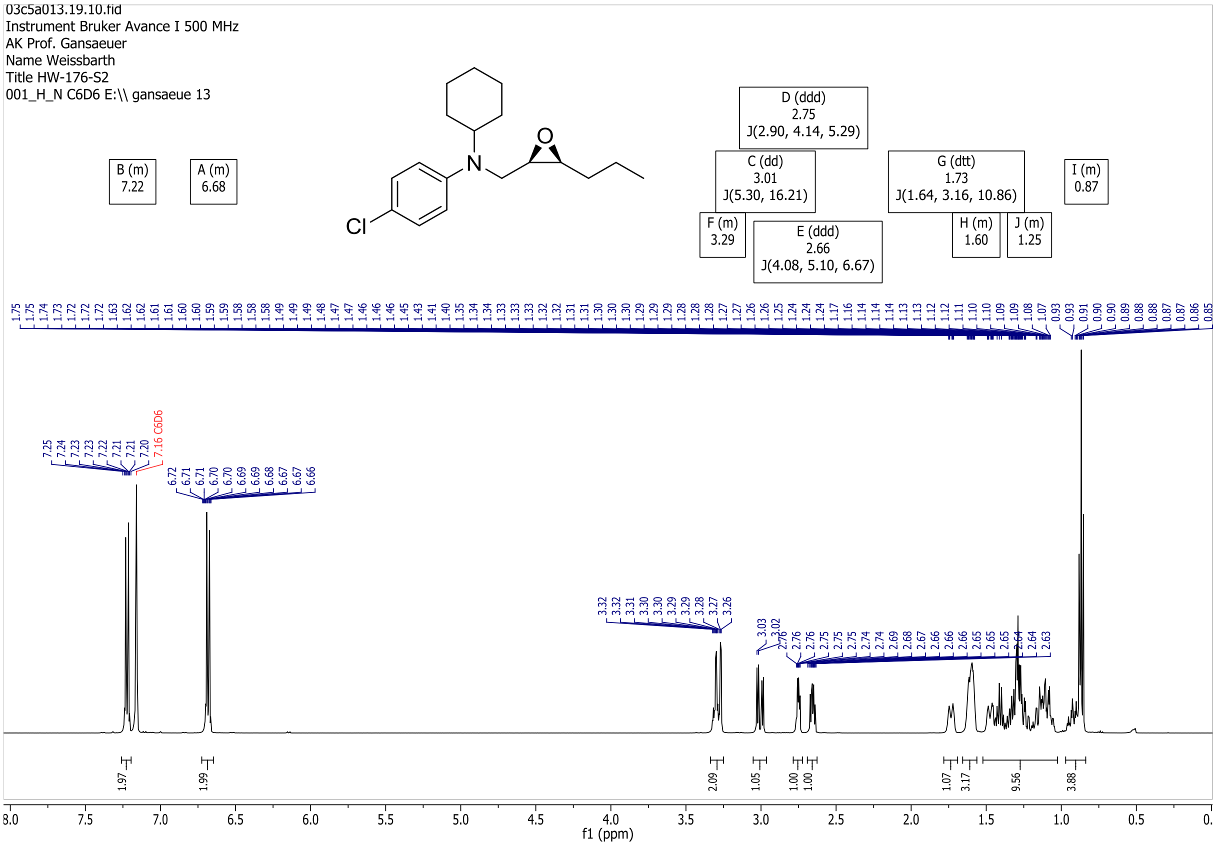
03c5a013.19.10.fid
Instrument Bruker Avance I 500 MHz
AK Prof. Gansaeuer
Name Weissbarth
Title HW-176-S2
001_H_N C6D6 E:\\ gansaeue 13
Cl
N
O
B (m) 7.22
A (m) 6.68
D (ddd) 2.75 J(2.90, 4.14, 5.29)
C (dd) 3.01 J(5.30, 16.21)
F (m) 3.29
E (ddd) 2.66 J(4.08, 5.10, 6.67)
G (dtt) 1.73 J(1.64, 3.16, 10.86)
H (m) 1.60
J (m) 1.25
I (m) 0.87
7.16 C6D6
f1 (ppm)

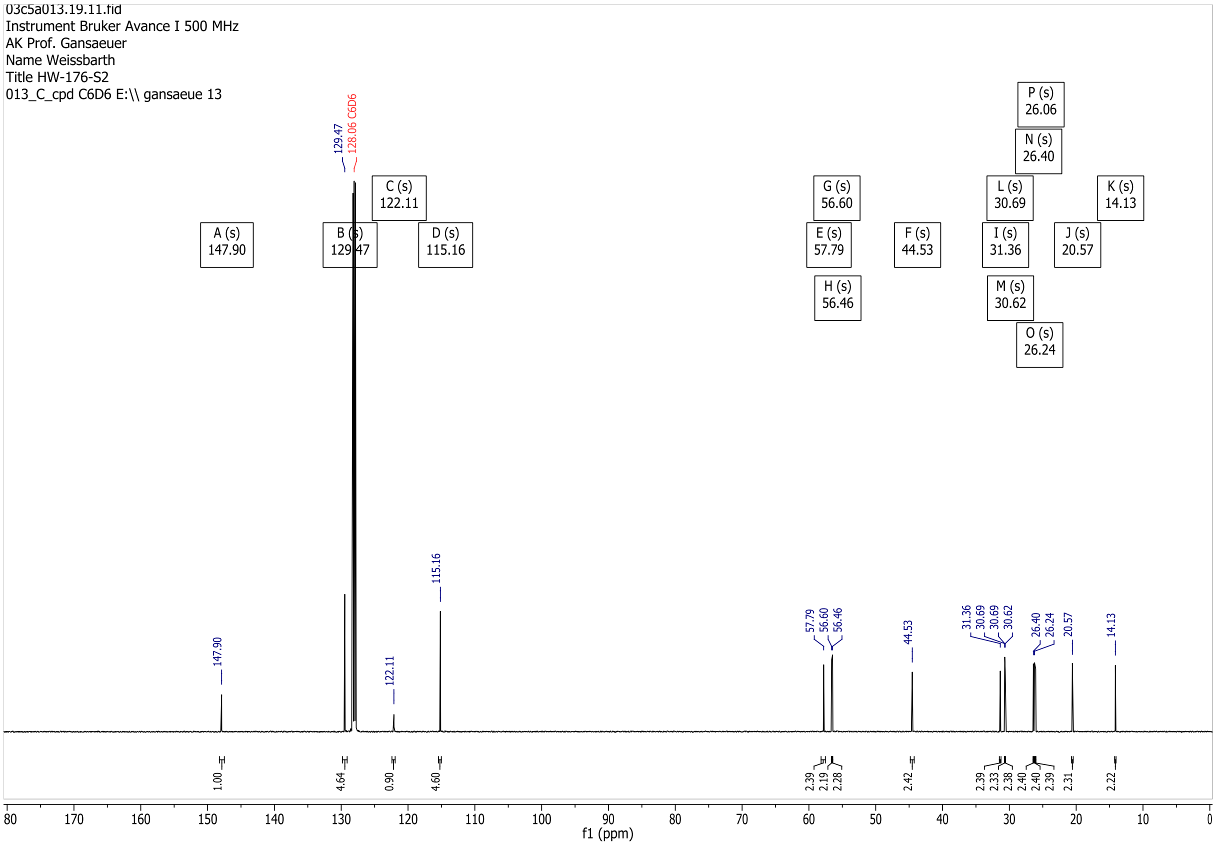
03c5a013.19.11.fid
Instrument Bruker Avance I 500 MHz
AK Prof. Gansaeuer
Name Weissbarth
Title HW-176-S2
013_C_cpd C6D6 E:\\ gansaeue 13
A (s) 147.90
B (s) 129.47
C (s) 122.11
D (s) 115.16
E (s) 57.79
G (s) 56.60
H (s) 56.46
F (s) 44.53
I (s) 31.36
L (s) 30.69
M (s) 30.62
N (s) 26.40
O (s) 26.24
P (s) 26.06
J (s) 20.57
K (s) 14.13
128.06 C6D6
f1 (ppm)

3.3.6 Synthesis of 4-chloro-*N*-cyclopentyl-*N*-(((*2R,3S*)-3-propyloxirane-2-yl)methyl)aniline (1i).

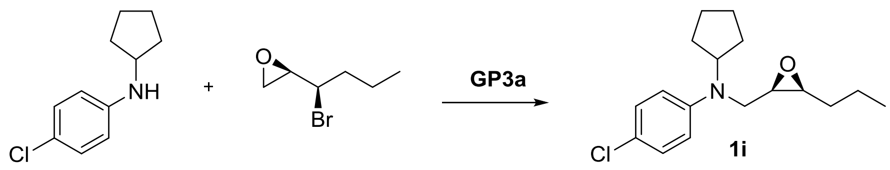

913 mg (4.7 mmol, 1.0 eq.) of 4-Chloro-*N*-cyclopentylaniline are reacted with 3.5 mL of 1.6M *n*BuLi-solution in Hexane (5.6 mmol, 1.2 eq.) and 1.00 g (2*R*,3*R*)-3-bromo-1,2-epoxyhexane (5.6 mmol, 1.2 eq.) following GP3a. After flash column chromatography (Al_2O_3, CH : MTBE 98:2) the product is obtained as a colorless oil in 40% yield (549 mg, 1.9 mmol) and is stored under argon atmosphere.

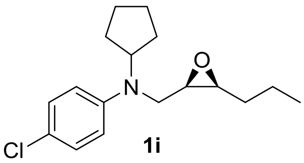

1i
$C_{17}H_{24}ClNO$
293,84 g/mol

R_f= 0.7 (20% EE in CH); **^{1}H NMR (400.1 MHz, C_6D_6, RT) δ [ppm]**= 0.85 (t, *J*= 7.2 Hz, 3H), 1.20-1.54 (m, 10H), 1.57-1.67 (m, 1H), 1.68-1.78 (m, 1H), 2.64 (td, *J*= 5.9 Hz, *J*= 4.1 Hz, 1H), 2.77 (ddd, *J*= 5.5 Hz, *J*= 4.1 Hz, *J*= 2.9 Hz, 1H), 2.96 (dd, *J*= 16.0 Hz, *J*= 5.5 Hz, 1H), 3.23 (dd, *J*= 16.0 Hz, *J*= 2.9 Hz, 1H), 3.66-3.76 (m, 1H), 6.64-6.70 (m, 2H), 7.17-7.22 (m, 2H); **^{13}C NMR (101 MHz, C_6D_6, RT) δ [ppm]**= 14.1, 20.5, 23.9, 24.0, 29.6, 30.1, 30.6, 46.4, 56.2, 56.5, 60.3, 116.6, 122.8, 129.3, 149.0; **IR ν_{max} (neat)[cm^{-1}]**= 511, 807, 976, 1098, 1188, 1244, 1277, 1350, 1397, 1455, 1495, 1594, 2870, 2957; **HRMS (ESI) m/z** calculated for $[M+H]^+$ 294.1619 u found 294.1616 u; **α_D^{20}($CHCl_3$)=** -38.9°.

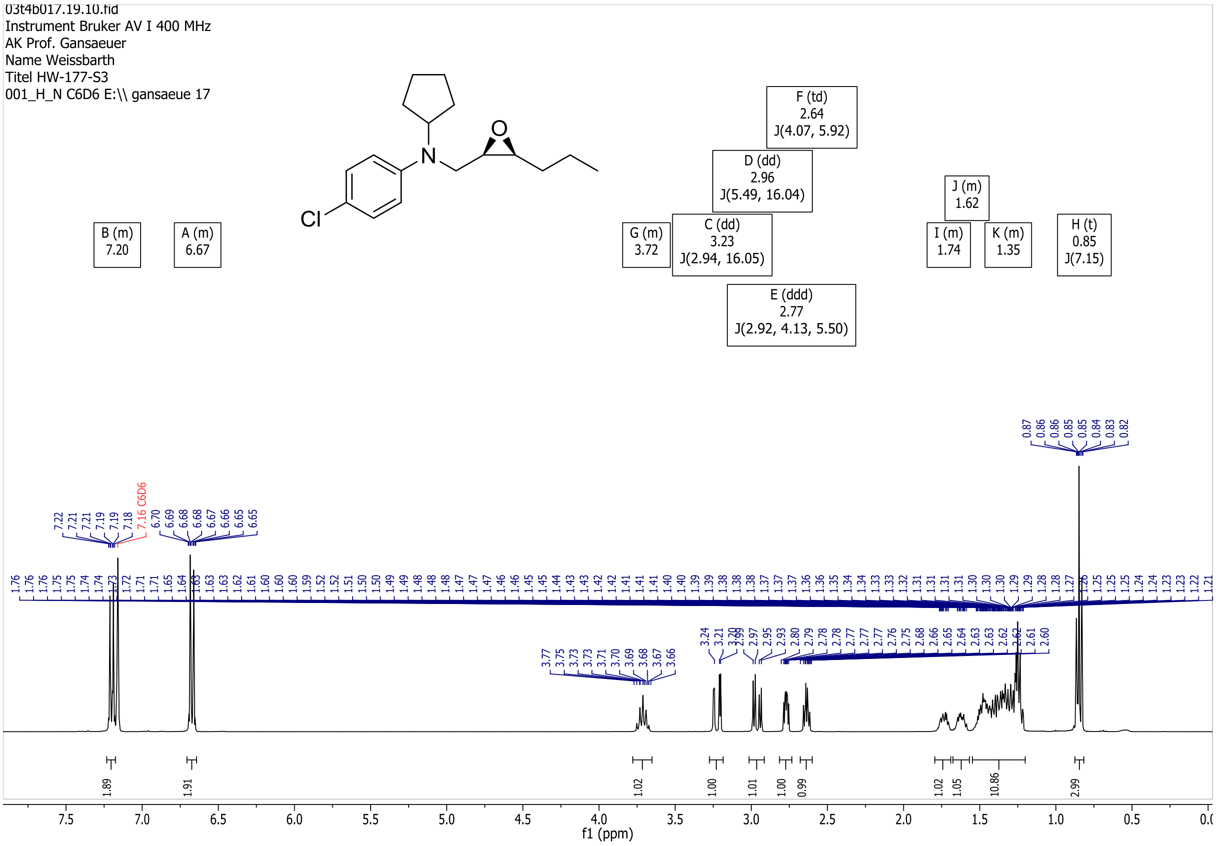
03t4b017.19.10.fid
Instrument Bruker AV I 400 MHz
AK Prof. Gansaeuer
Name Weissbarth
Titel HW-177-S3
001_H_N C6D6 E:\\ gansaeue 17
Cl
N
O
B (m) 7.20
A (m) 6.67
G (m) 3.72
C (dd) 3.23 J(2.94, 16.05)
D (dd) 2.96 J(5.49, 16.04)
F (td) 2.64 J(4.07, 5.92)
E (ddd) 2.77 J(2.92, 4.13, 5.50)
J (m) 1.62
I (m) 1.74
K (m) 1.35
H (t) 0.85 J(7.15)
7.16 C6D6
1.89
1.91
1.02
1.00
1.01
1.00
0.99
1.02
1.05
10.86
2.99
f1 (ppm)

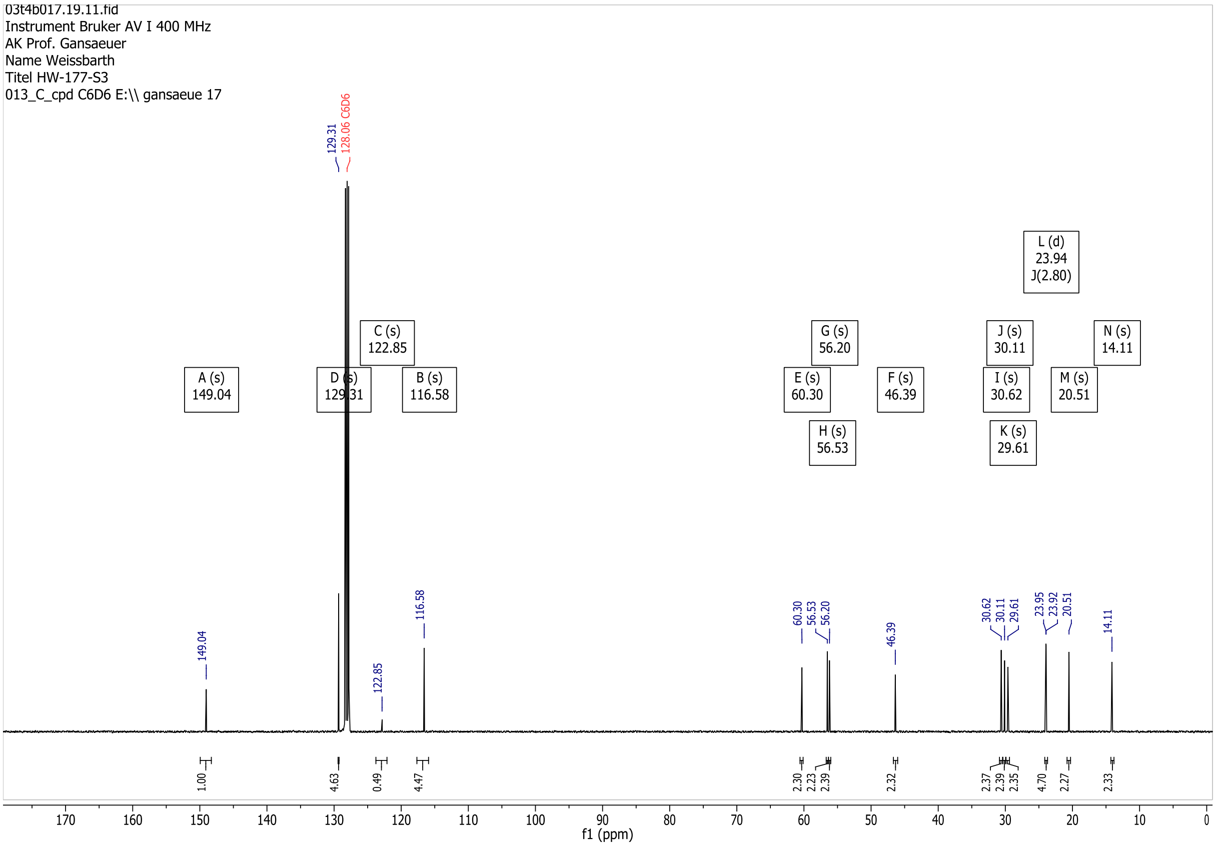
03t4b017.19.11.fid
Instrument Bruker AV I 400 MHz
AK Prof. Gansaeuer
Name Weissbarth
Titel HW-177-S3
013_C_cpd C6D6 E:\\ gansaeue 17
129.31
128.06 C6D6
A (s) 149.04
D (s) 129.31
C (s) 122.85
B (s) 116.58
E (s) 60.30
G (s) 56.20
H (s) 56.53
F (s) 46.39
J (s) 30.11
I (s) 30.62
K (s) 29.61
L (d) 23.94 J(2.80)
M (s) 20.51
N (s) 14.11
f1 (ppm)

3.3.7 Synthesis of 4-chloro-*N*-isopropyl-*N*-(((*2R,3S*)-3-propyloxiran-2-yl)methyl)aniline (1j).

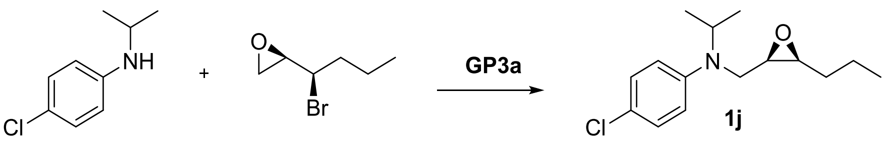

848.3 mg (5 mmol, 1.0 eq.) of 4-chloro-*N*-isopropylaniline are reacted with 2.4 mL of 2.5M *n*BuLi-solution in Hexane (6 mmol, 1.2 eq.) and 1.07 g (2*R*,3*R*)-3-bromo-1,2-epoxyhexane (6 mmol, 1.2 eq.) following GP3a. After flash column chromatography (Al_2O_3, CH : MTBE 98:2) the product is obtained as a colorless oil in 30% yield (386 mg, 1.55 mmol) and is stored under argon atmosphere.

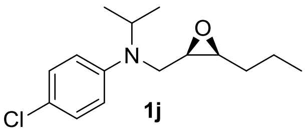

1j

$C_{15}H_{22}ClNO$
267,80 g/mol

Rf= 0.7 (20% EE in CH); **^{1}H NMR (400.1 MHz, C_6D_6, RT) δ [ppm]**= 0.84-0.88 (m, 6H), 0.94 (d, *J*= 6.6 Hz, 3H), 1.22-1.46 (m, 4H), 2.63 (ddd, *J*= 6.6 Hz, *J*= 5.4 Hz, *J*= 4.1 Hz, 1H), 2.72 (ddd, *J*= 5.4 Hz, *J*= 4.1 Hz, *J*= 2.9 Hz, 1H), 2.91 (dd, *J*= 16.2 Hz, *J*= 5.4 Hz, 1H), 3.20 (dd, *J*= 16.2 Hz, *J*= 2.9 Hz, 1H), 3.61 (hept, *J*= 6.6 Hz, 1H), 6.58-6.22 (m, 2H) 7.18-7.22 (m, 2H); **^{13}C NMR (125.5 MHz, C_6D_6, RT) δ [ppm]**= 14.1, 19.6, 20.3, 20.5, 30.6, 43.5, 48.6, 56.2, 56.6, 115.2, 122.2, 129.5, 147.9; **IR ν_{max} (neat)[cm^{-1}]**= 510, 753, 807, 1101, 1161, 1188, 1246, 1283, 1393, 1465, 1496, 1596, 2963; **HRMS (ESI) m/z** calculated for $[M+H]^+$ 268.1463 u found 268.1462 u; **α_D^{20}($CHCl_3$)=** -27.0°.

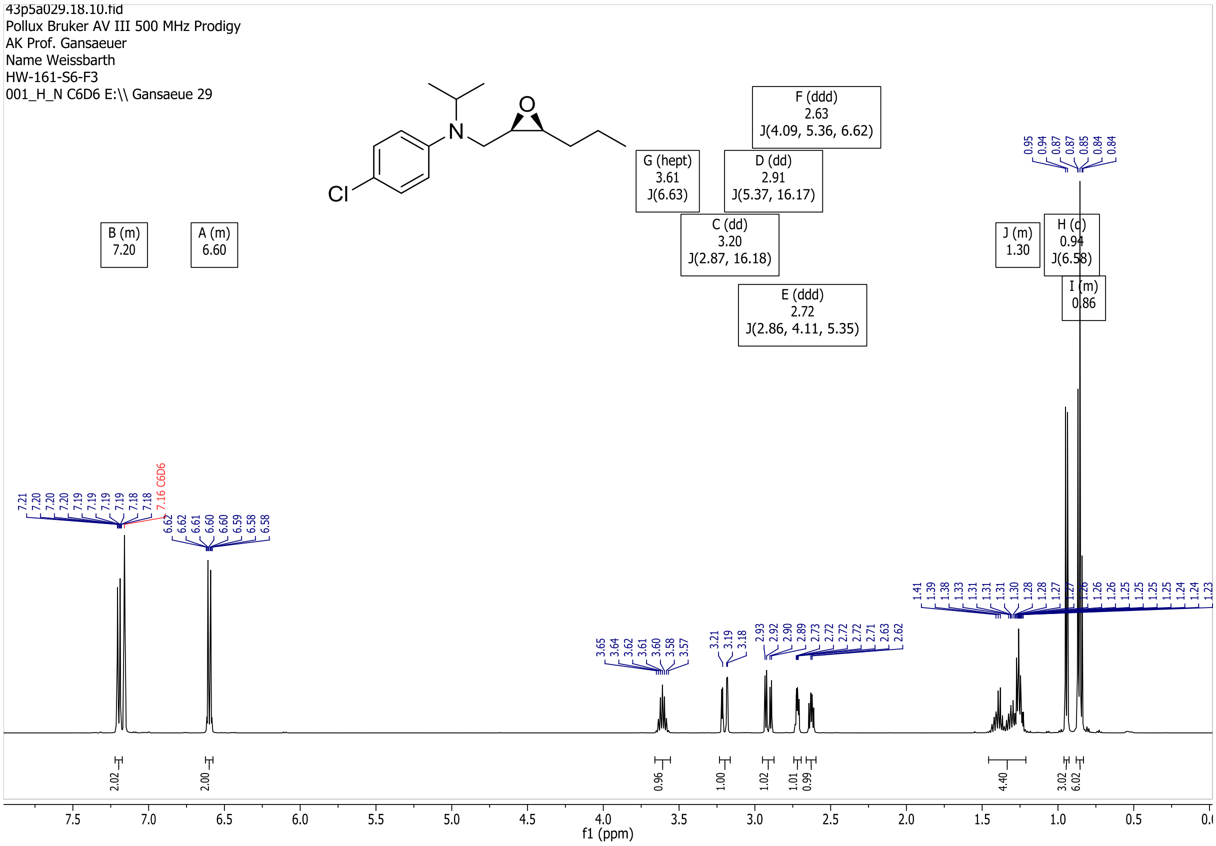
43p5a029.18.10.fid
Pollux Bruker AV III 500 MHz Prodigy
AK Prof. Gansaeuer
Name Weissbarth
HW-161-S6-F3
001_H_N C6D6 E:\\ Gansaeue 29
Cl
N
O
B (m) 7.20
A (m) 6.60
G (hept) 3.61 J(6.63)
C (dd) 3.20 J(2.87, 16.18)
D (dd) 2.91 J(5.37, 16.17)
E (ddd) 2.72 J(2.86, 4.11, 5.35)
F (ddd) 2.63 J(4.09, 5.36, 6.62)
J (m) 1.30
H (d) 0.94 J(6.58)
I (m) 0.86
7.21 7.20 7.20 7.20 7.20 7.19 7.19 7.19 7.19 7.18 7.18
7.16 C6D6
6.62 6.62 6.61 6.60 6.60 6.59 6.58 6.58
3.65 3.64 3.62 3.61 3.60 3.58 3.57
3.21 3.19 3.18
2.93 2.92 2.90 2.89 2.73 2.72 2.72 2.72 2.71 2.63 2.62
1.41 1.39 1.38 1.33 1.31 1.31 1.31 1.30 1.28 1.28 1.27 1.27 1.26 1.26 1.25 1.25 1.25 1.25 1.24 1.24 1.23
0.95 0.94 0.87 0.87 0.85 0.85 0.84 0.84
2.02 2.00 0.96 1.00 1.02 1.01 0.99 4.40 3.02 6.02
7.5 7.0 6.5 6.0 5.5 5.0 4.5 4.0 3.5 3.0 2.5 2.0 1.5 1.0 0.5 0.0
f1 (ppm)

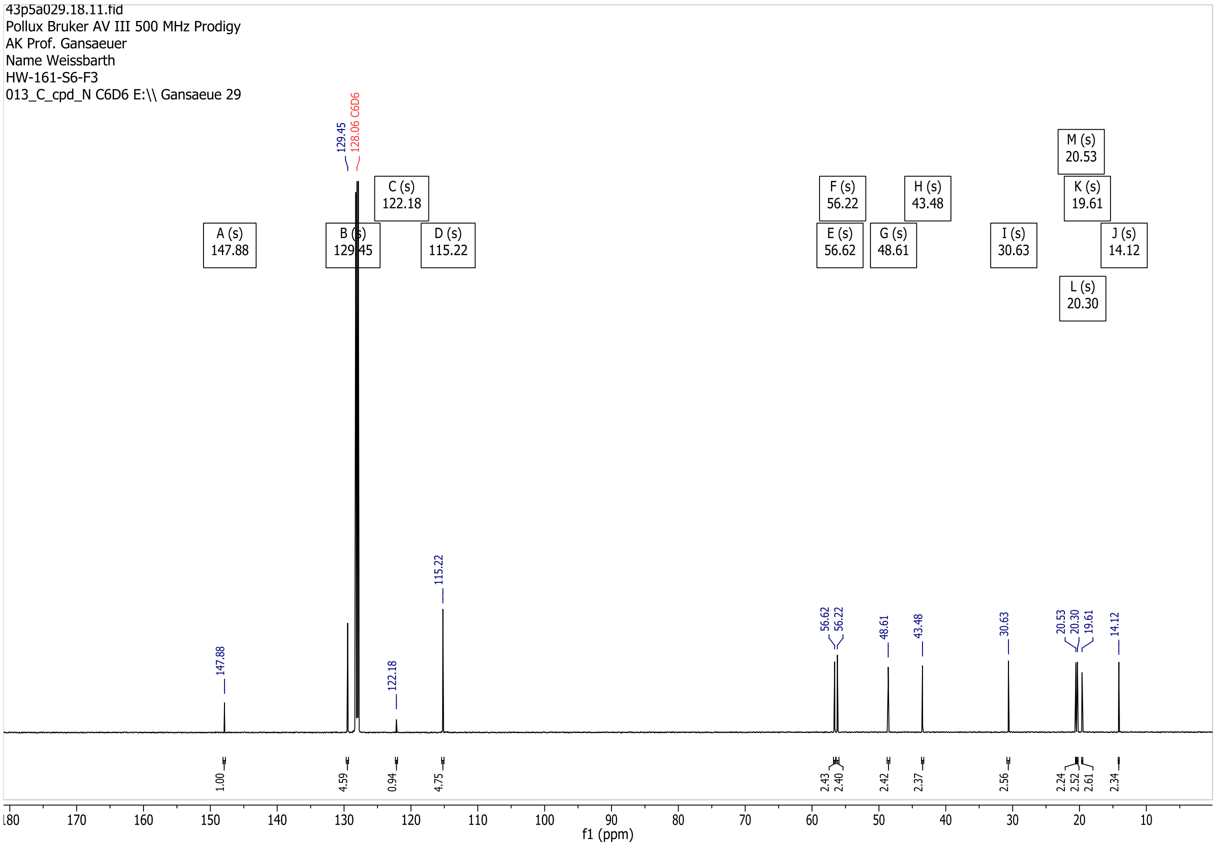
43p5a029.18.11.fid
Pollux Bruker AV III 500 MHz Prodigy
AK Prof. Gansaeuer
Name Weissbarth
HW-161-S6-F3
013_C_cpd_N C6D6 E:\\ Gansaeue 29
A (s) 147.88
B (s) 129.45
C (s) 122.18
D (s) 115.22
E (s) 56.62
F (s) 56.22
G (s) 48.61
H (s) 43.48
I (s) 30.63
J (s) 14.12
K (s) 19.61
L (s) 20.30
M (s) 20.53
129.45
128.06 C6D6
147.88 122.18 115.22 56.62 56.22 48.61 43.48 30.63 20.53 20.30 19.61 14.12
1.00 4.59 0.94 4.75 2.43 2.40 2.42 2.37 2.56 2.24 2.52 2.61 2.34
180 170 160 150 140 130 120 110 100 90 80 70 60 50 40 30 20 10
f1 (ppm)

3.3.8 Synthesis of 4-fluoro-*N*-isopropyl-*N*-(((*2R,3S*)-3-propyloxirane-2-yl)methyl)aniline (1k).

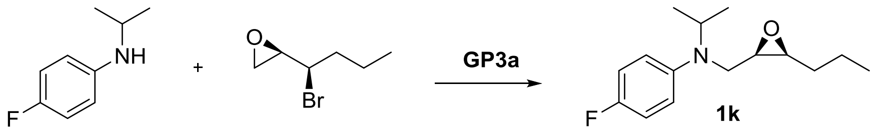

2.45 g (16 mmol, 1.0 eq.) of 4-Fluoro-*N*-cyclopentylaniline are reacted with 7.0 mL of 2.5M *n*BuLi-solution in Hexane (17.6 mmol, 1.1 eq.) and 4.33 g (2*R*,3*R*)-3-bromo-1,2-epoxyhexane (24.2 mmol, 1.5 eq.) following GP3a. After flash column chromatography (Al_2O_3, CH : MTBE 98:2) the product is obtained as a colorless oil in 31% yield (1.23 g, 4.9 mmol) and is stored under argon atmosphere.

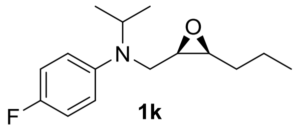

1k
$C_{15}H_{22}FNO$
251,35 g/mol

R_f= 0.4 (10% MTBE in CH); **^{1}H NMR (400.1 MHz, C_6D_6, RT) δ [ppm]**= 0.83-0.88 (m, 3H), 0.90 (d, *J*= 6.6 Hz, 3H), 0.95 (d, *J*= 6.6 Hz, 3H), 1.23-1.46 (m, 4H), 2.64 (td, *J*= 5.9 Hz, *J*= 4.1 Hz, 1H), 2.79 (ddd, *J*= 5.2 Hz, *J*= 4.1 Hz, *J*= 3.2 Hz, 1H), 2.96 (dd, *J*= 15.8 Hz, *J*= 5.3 Hz, 1H), 3.20 (dd, *J*= 15.8 Hz, *J*= 3.2 Hz, 1H), 3.60 (hept, *J*= 6.6 Hz, 1H), 6.64-6.70 (m, 2H), 6.88-6.95 (m, 2H); **^{13}C NMR (101 MHz, C_6D_6, RT) δ [ppm]**= 14.1, 19.5, 20.4, 20.5, 30.6, 44.3, 49.7, 56.4, 56.6, 115.9 (d, $J_{C,F}$= 21.9 Hz), 116.5 (d, $J_{C,F}$= 7.2 Hz), 146.0 (d, $J_{C,F}$= 2.0 Hz), 156.5 (d, $J_{C,F}$= 235.9 Hz); **^{19}F NMR (470 MHz, C_6D_6, RT) δ [ppm]**= -127.6; **IR ν_{max} (neat)[cm^{-1}]**= 515, 557, 809, 1169, 1189, 1230, 1464, 1508, 2965; **HRMS (ESI) m/z** calculated for $[M+H]^+$ 252.1758 u found 252.1755 u; **α_D^{20}($CHCl_3$)=** -27.1°.

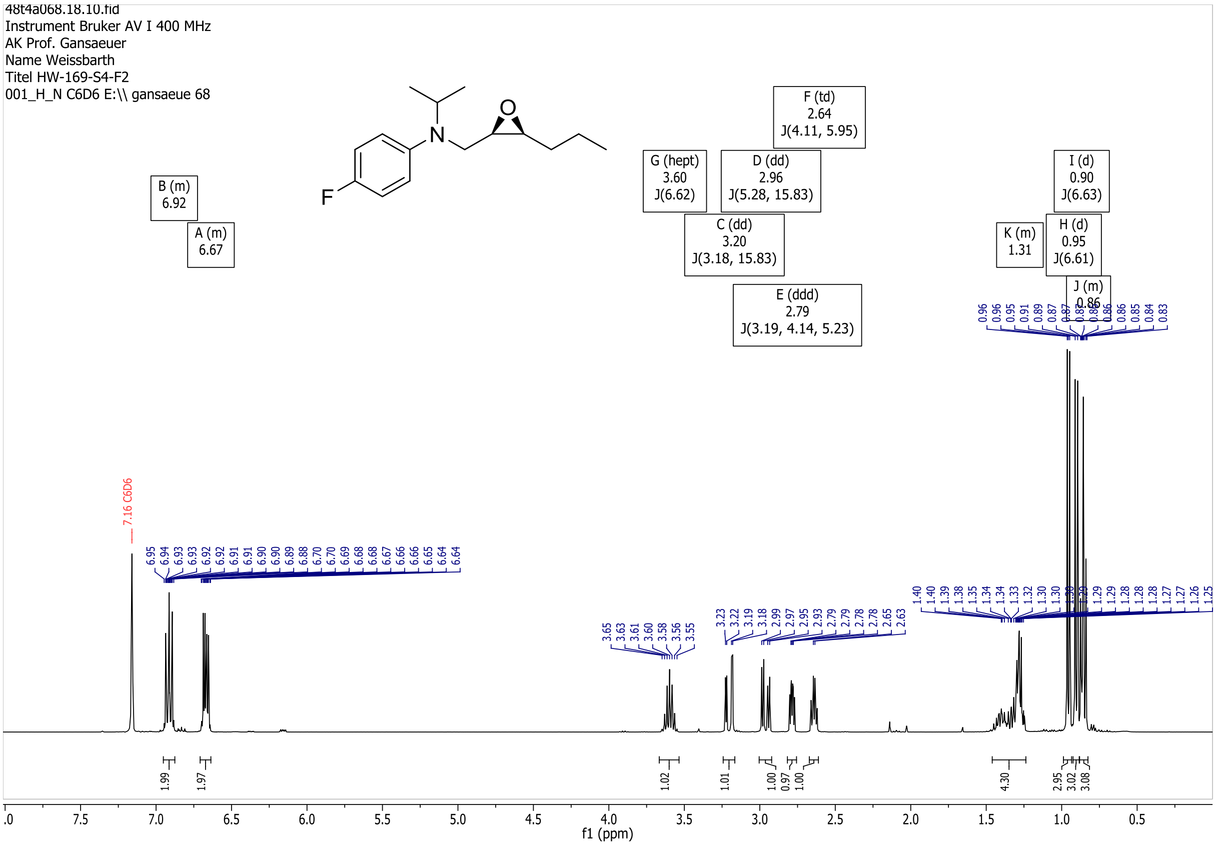
48t4a068.18.10.fid
Instrument Bruker AV I 400 MHz
AK Prof. Gansaeuer
Name Weissbarth
Titel HW-169-S4-F2
001_H_N C6D6 E:\\ gansaeue 68
F
N
O
B (m) 6.92
A (m) 6.67
G (hept) 3.60 J(6.62)
F (td) 2.64 J(4.11, 5.95)
D (dd) 2.96 J(5.28, 15.83)
C (dd) 3.20 J(3.18, 15.83)
E (ddd) 2.79 J(3.19, 4.14, 5.23)
K (m) 1.31
I (d) 0.90 J(6.63)
H (d) 0.95 J(6.61)
J (m) 0.86
7.16 C6D6
f1 (ppm)

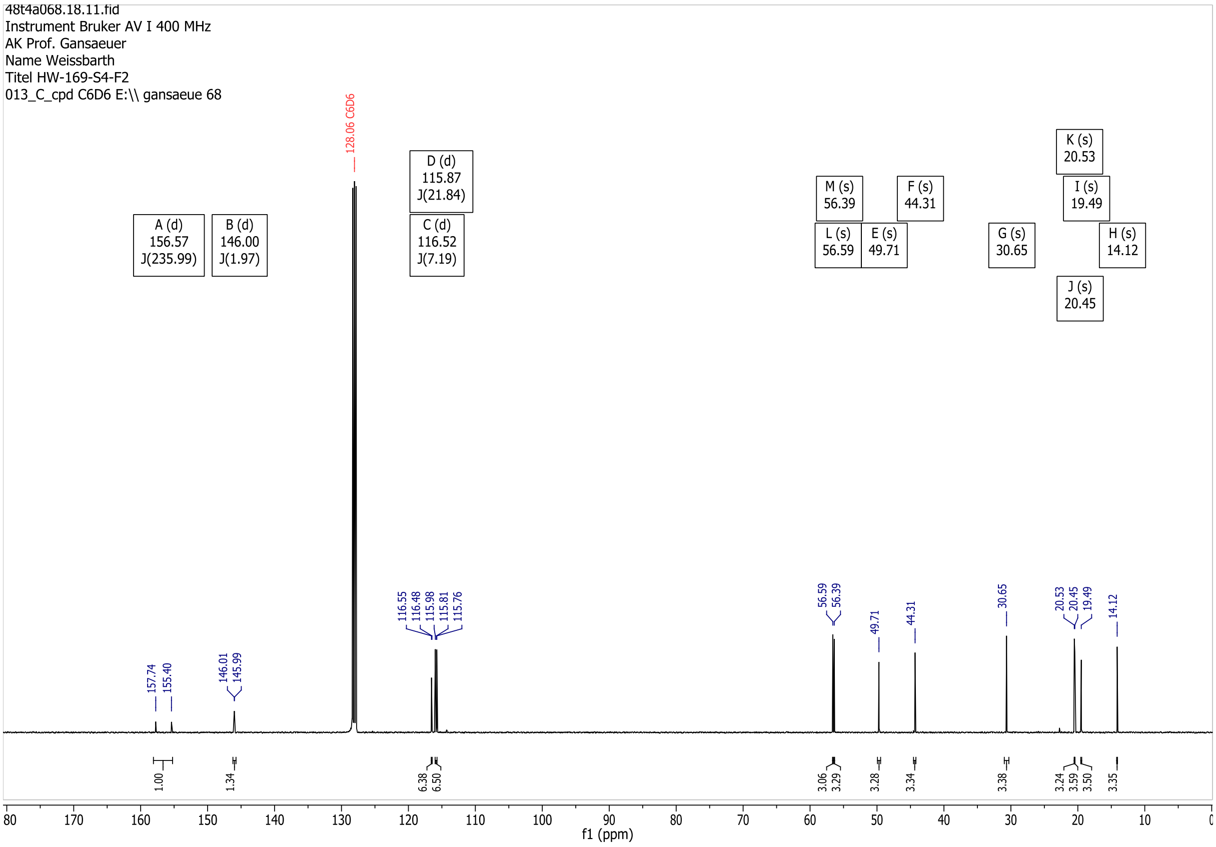
48t4a068.18.11.fid
Instrument Bruker AV I 400 MHz
AK Prof. Gansaeuer
Name Weissbarth
Titel HW-169-S4-F2
013_C_cpd C6D6 E:\\ gansaeue 68
128.06 C6D6
A (d) 156.57 J(235.99)
B (d) 146.00 J(1.97)
D (d) 115.87 J(21.84)
C (d) 116.52 J(7.19)
M (s) 56.39
L (s) 56.59
F (s) 44.31
E (s) 49.71
G (s) 30.65
K (s) 20.53
I (s) 19.49
H (s) 14.12
J (s) 20.45
f1 (ppm)

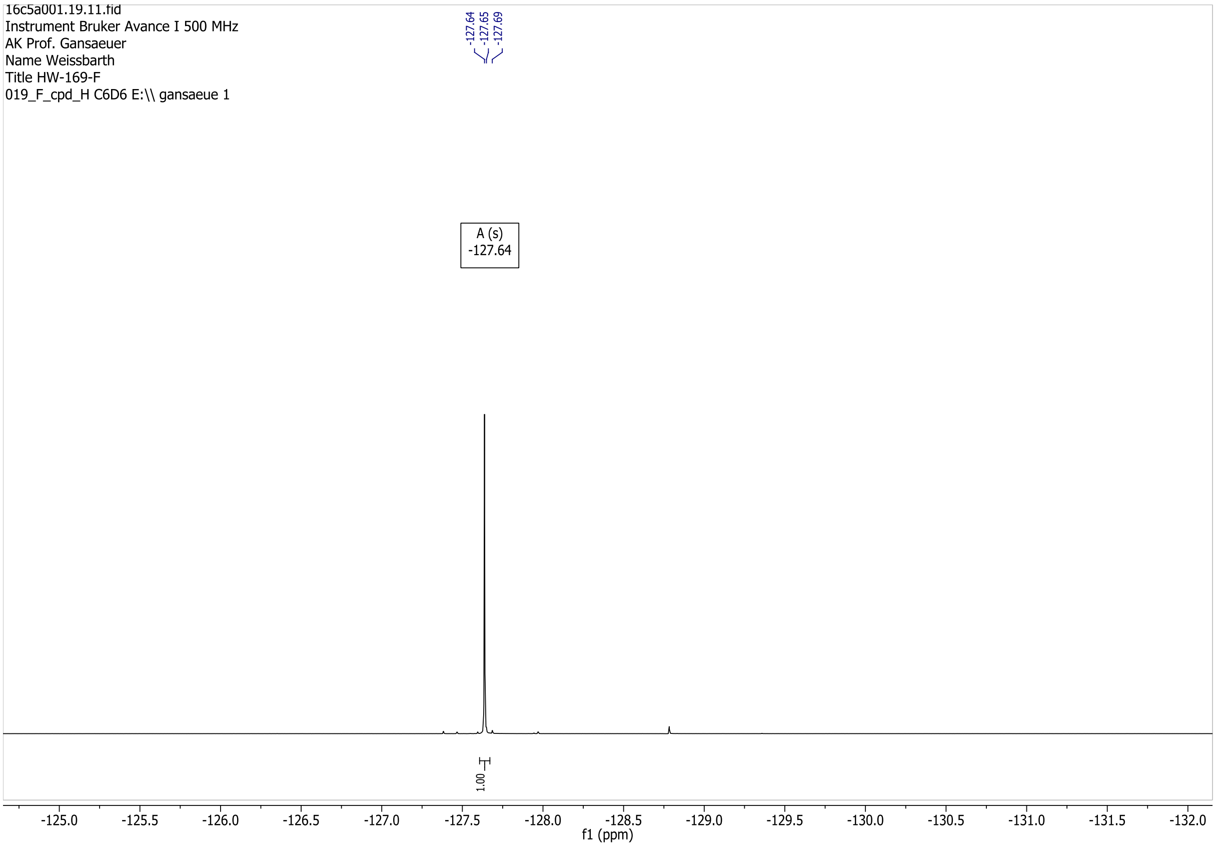
16c5a001.19.11.fid
Instrument Bruker Avance I 500 MHz
AK Prof. Gansaeuer
Name Weissbarth
Title HW-169-F
019_F_cpd_H C6D6 E:\\ gansaeue 1
-127.64
-127.65
-127.69
A (s)
-127.64
1.00
-125.0
-125.5
-126.0
-126.5
-127.0
-127.5
-128.0
-128.5
-129.0
-129.5
-130.0
-130.5
-131.0
-131.5
-132.0
f1 (ppm)

3.3.9 Synthesis of *N*-cyclopropyl-4-fluoro-*N*-(((*2R,3S*)-3-propyloxiran-2-yl)methyl)aniline (1l).

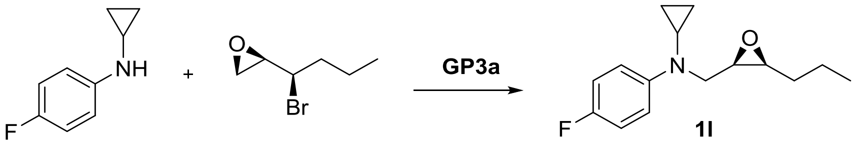

357 mg (2.36 mmol, 1.0 eq.) of 4-fluoro-N-cyclopropylaniline are reacted with 1.05 mL 2.5M *n*BuLi-solution in Hexane (2.6 mmol, 1.1 eq.) and 507 mg (2*R*,3*R*)-3-bromo-1,2-epoxyhexane (1.2 eq., 2.83 mmol) following GP3a. After flash column chromatography (SiO_2, CH : MTBE 98:2) the product is obtained as a light yellow oil in 66% yield (386 mg, 1.55 mmol) and is stored under argon atmosphere.

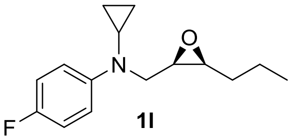

1l

$C_{15}H_{20}FNO$

249,33 g/mol

R_f= 0.7 (20% EE in CH)**; ^{1}H NMR (300.1 MHz, C_6D_6, RT) δ [ppm]=** 0.34-0.51 (m, 4H), 0.82-0.89 (m, 3H), 1.21-1.46 (m, 4H), 2.13-2.22 (m, 1H), 2.61 (ddd, *J*= 6.4 Hz, *J*= 5.3 Hz, *J*= 4.1 Hz, 1H), 2.81 (dt, *J*= 5.9 Hz, *J*= 4.1 Hz, 1H), 3.19 (dd, *J*= 15.5 Hz, *J*= 5.9 Hz, 1H), 3.32 (dd, *J*= 15.5 Hz, *J*= 4.1 Hz, 1H), 6.18-6.81 (m, 2H), 6.89-6.98 (m, 2H); **^{13}C NMR (75.5 MHz, C_6D_6, RT) δ [ppm]**= 9.6, 9.6, 14.1, 20.5, 30.5, 32.9, 51.9, 54.7, 56.3, 115.6 (d, $J_{C,F}$= 21.9 Hz), 116.3 (d, $J_{C,F}$= 7.2 Hz), 146.7 (d, $J_{C,F}$ = 2.1 Hz), 156.9 (d, $J_{C,F}$= 236.2 Hz); **^{19}F NMR (470 MHz, C_6D_6, RT) δ [ppm]**= -127.3; **IR ν_{max} (neat)[cm^{-1}]**= 771, 817, 1223, 1364, 1455, 1507, 2962; **HRMS (ESI) m/z** calculated for $[M+H]^+$ 250.1602 u found 250.1602 u; **α_D^{20}($CHCl_3$)=** -10.0°.

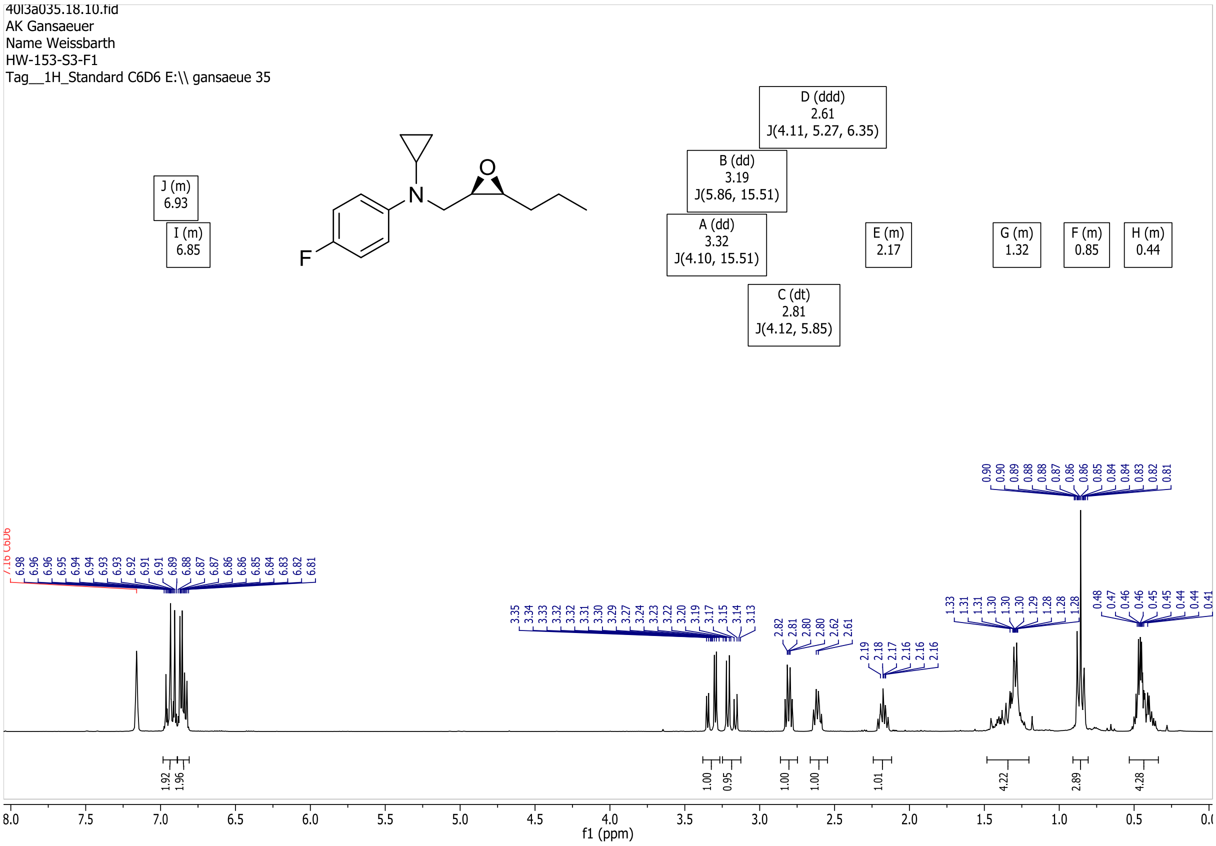
40l3a035.18.10.fid
AK Gansaeuer
Name Weissbarth
HW-153-S3-F1
Tag__1H_Standard C6D6 E:\\ gansaeue 35
F
N
O
D (ddd)
2.61
J(4.11, 5.27, 6.35)
B (dd)
3.19
J(5.86, 15.51)
A (dd)
3.32
J(4.10, 15.51)
C (dt)
2.81
J(4.12, 5.85)
J (m)
6.93
I (m)
6.85
E (m)
2.17
G (m)
1.32
F (m)
0.85
H (m)
0.44
7.16 C6D6
f1 (ppm)

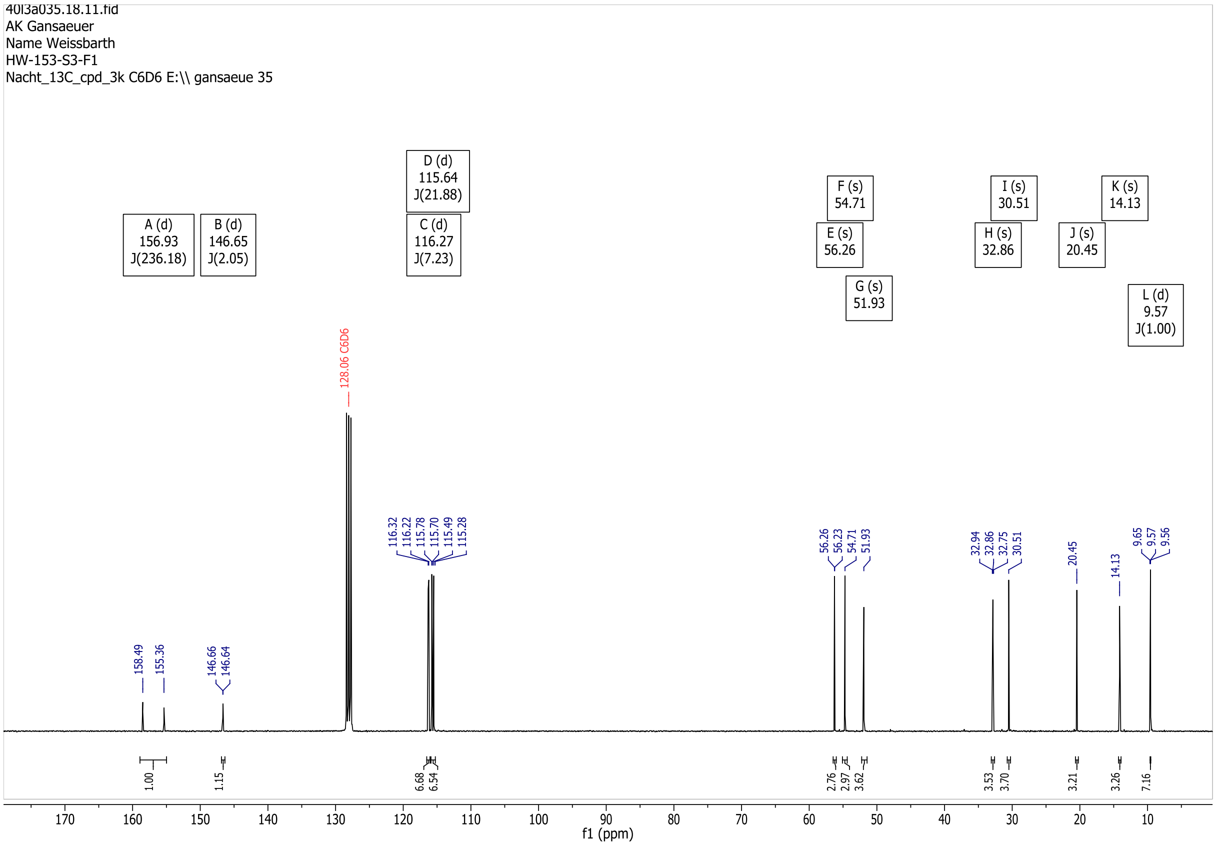
40l3a035.18.11.fid
AK Gansaeuer
Name Weissbarth
HW-153-S3-F1
Nacht_13C_cpd_3k C6D6 E:\\ gansaeue 35
A (d)
156.93
J(236.18)
B (d)
146.65
J(2.05)
D (d)
115.64
J(21.88)
C (d)
116.27
J(7.23)
F (s)
54.71
E (s)
56.26
G (s)
51.93
I (s)
30.51
H (s)
32.86
K (s)
14.13
J (s)
20.45
L (d)
9.57
J(1.00)
128.06 C6D6
f1 (ppm)

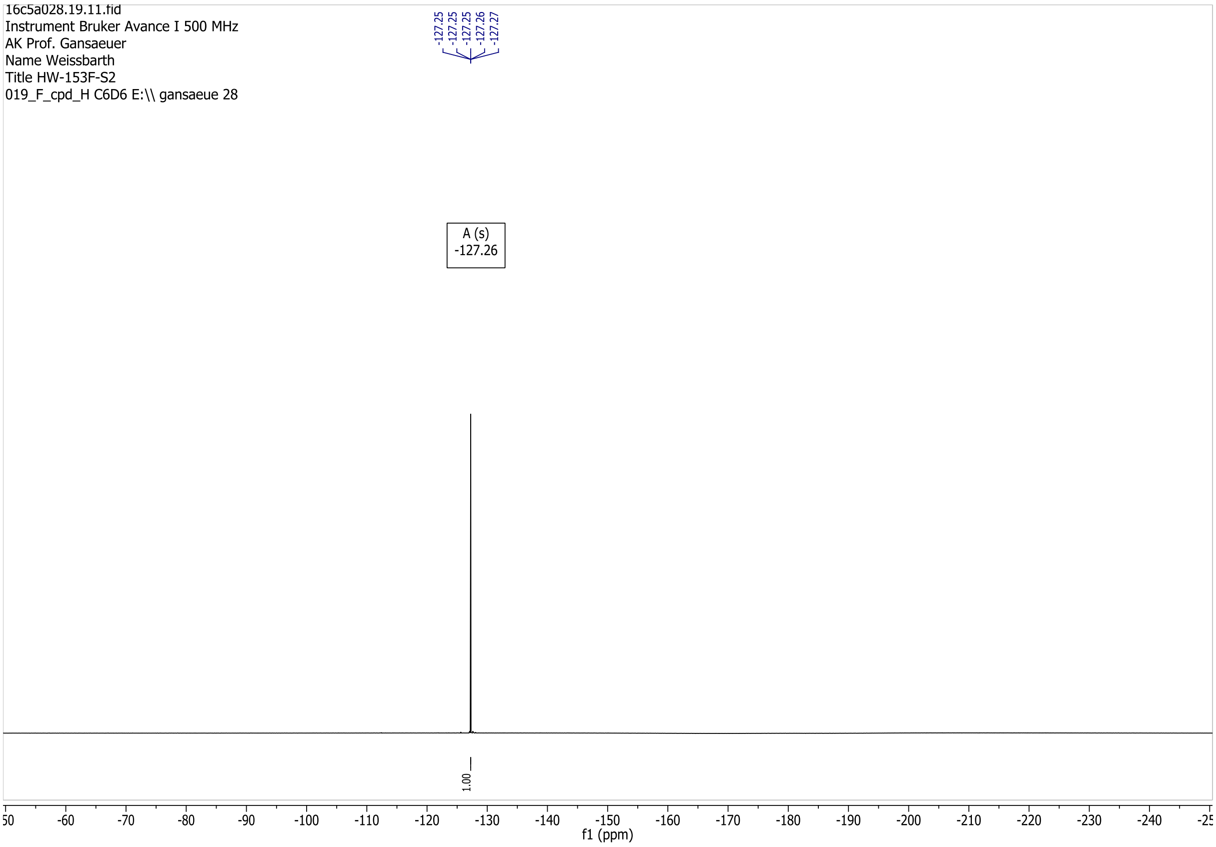
16c5a028.19.11.fid
Instrument Bruker Avance I 500 MHz
AK Prof. Gansaeuer
Name Weissbarth
Title HW-153F-S2
019_F_cpd_H C6D6 E:\\ gansaeue 28
-127.25
-127.25
-127.25
-127.26
-127.27
A (s)
-127.26
1.00
-60
-70
-80
-90
-100
-110
-120
-130
-140
-150
-160
-170
-180
-190
-200
-210
-220
-230
-240
f1 (ppm)

3.3.10 Synthesis of methyl 4-(methyl(((*2R,3S*)-3-propyloxiran-2-yl)methyl)amino)benzoate (1n).

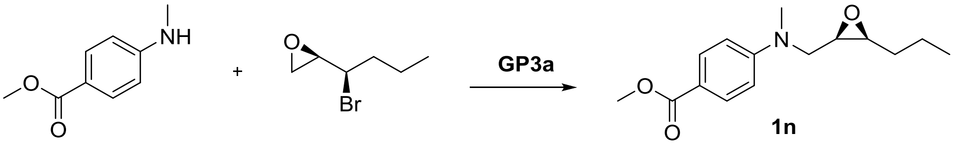

1.34 g (8.1 mmol, 1.0 eq.) of Methyl-4-(methylamino)benzoate are reacted with 3.6 mL of 2.5M *n*BuLi-solution in Hexane (8.9 mmol, 1.1 eq.) and 1.74 g (2*R*,3*R*)-3-bromo-1,2-epoxyhexane (9.7 mmol, 1.2 eq.) following GP3a. After flash column chromatography (SiO_2, CH : MTBE 98:2) the product is obtained as a colorless oil in 29% yield (613 mg, 2.8 mmol) and is stored under argon atmosphere.

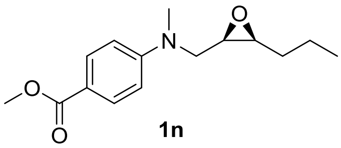

1n

$C_{15}H_{21}NO_3$
263,34 g/mol

R_f= 0.4 (30% Et_2O in CH);**^{1}H NMR (300.1 MHz, C_6D_6, RT) δ [ppm]**= 0.83 (t, *J*= 7.1 Hz, 3H), 1.13-1.40 (m, 4H)z, 2.53 (s, 3H), 2.65 (dt, *J*= 6.5 Hz, *J*= 3.9 Hz, 1H), 2.87 (ddd, *J*= 15.7 Hz, *J*= 6.5 Hz, *J*= 1.0 Hz, 1H), 3.16 (dd, *J*= 15.5 Hz, *J*= 3.7 Hz, 1H), 3.62 (s, 3H), 6.47-6.54 (m, 2H), 8.21-8.29 (m, 2H); **^{13}C NMR (75 MHz, C_6D_6, RT) δ [ppm]**= 14.1, 20.4, 30.3, 38.2, 51.0, 51.2, 54.2, 55.6, 111.6, 131.9, 152.7, 167.1; **IR ν_{max} (neat)[cm^{-1}]**= 700, 752, 767, 839, 946, 1097, 1145, 1175, 1188, 1252, 1261, 1279, 1378, 1523, 1599, 1702, 2981; **HRMS (ESI) m/z** calculated for $[M+H]^+$ 264.1594 u found 264.1591 u; **α_D^{20}(CHCl$_3$)=** -1.9°.

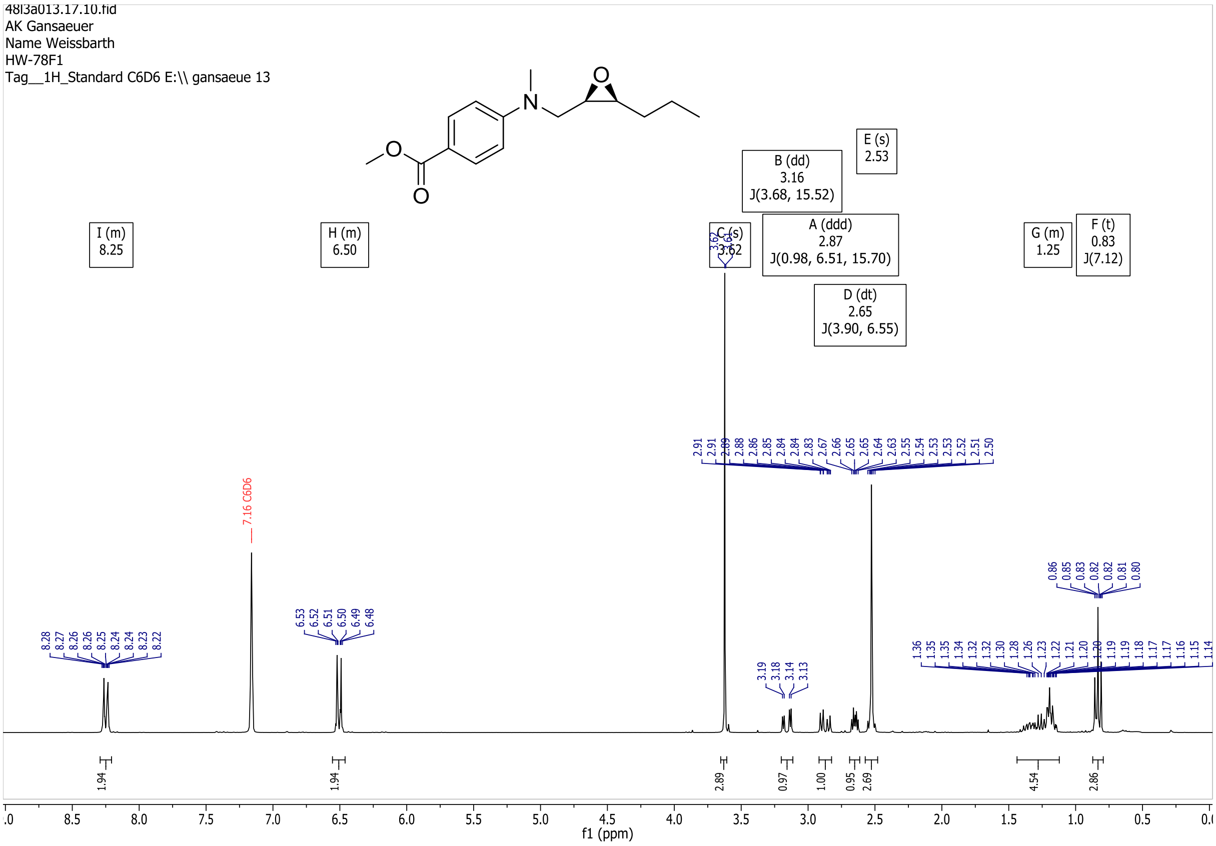
48l3a013.17.10.fid
AK Gansaeuer
Name Weissbarth
HW-78F1
Tag__1H_Standard C6D6 E:\\ gansaeue 13
I (m) 8.25
H (m) 6.50
C (s) 3.62
B (dd) 3.16 J(3.68, 15.52)
A (ddd) 2.87 J(0.98, 6.51, 15.70)
D (dt) 2.65 J(3.90, 6.55)
E (s) 2.53
G (m) 1.25
F (t) 0.83 J(7.12)
7.16 C6D6
f1 (ppm)

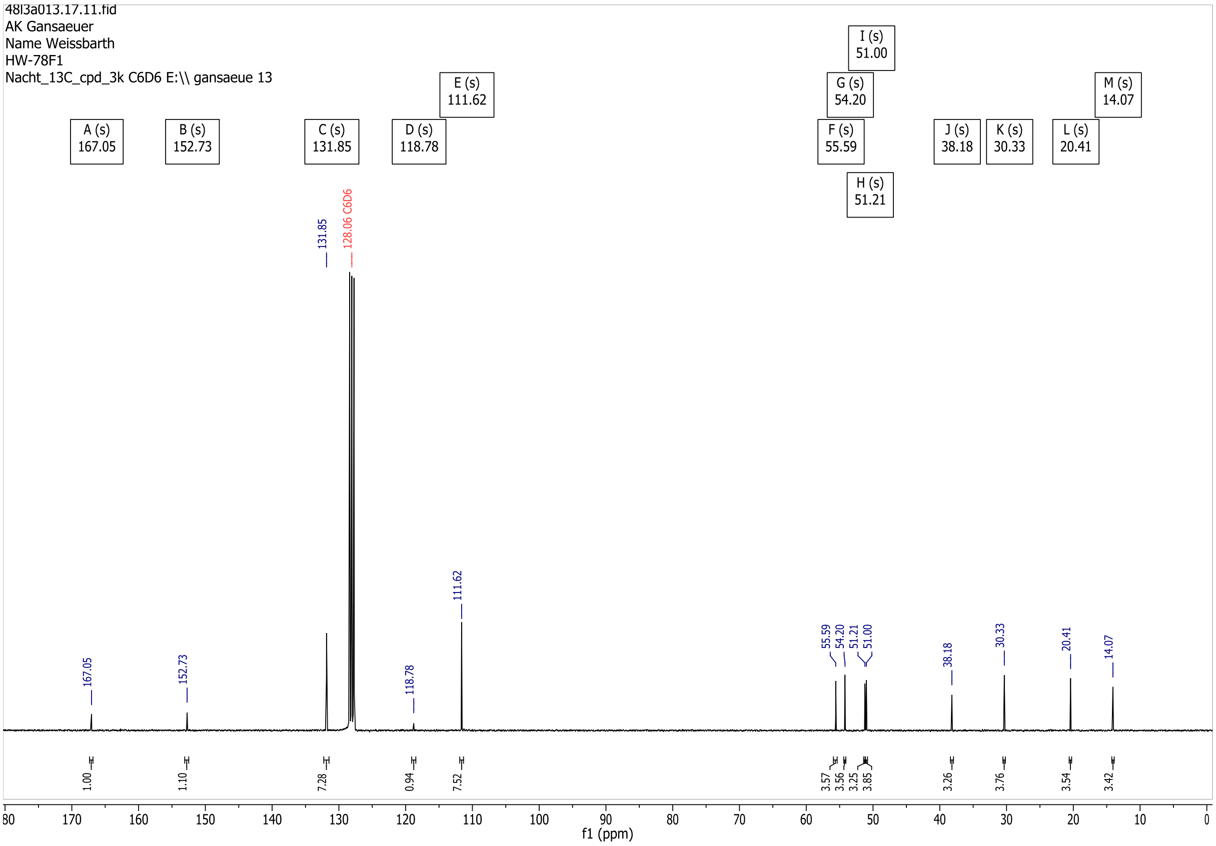
48l3a013.17.11.fid
AK Gansaeuer
Name Weissbarth
HW-78F1
Nacht_13C_cpd_3k C6D6 E:\\ gansaeue 13
A (s) 167.05
B (s) 152.73
C (s) 131.85
D (s) 118.78
E (s) 111.62
F (s) 55.59
G (s) 54.20
H (s) 51.21
I (s) 51.00
J (s) 38.18
K (s) 30.33
L (s) 20.41
M (s) 14.07
128.06 C6D6
f1 (ppm)

3.4 SiO_2-catalyzed aminolysis of bromo-epoxides to bromo-alcohols and subsequent formation of aniline-epoxides (A→1).

3.4.1. Synthesis of *N*-phenyl-*N*-(((*2R,3S*)-3-propyloxiran-2-yl)methyl)aniline (1a).

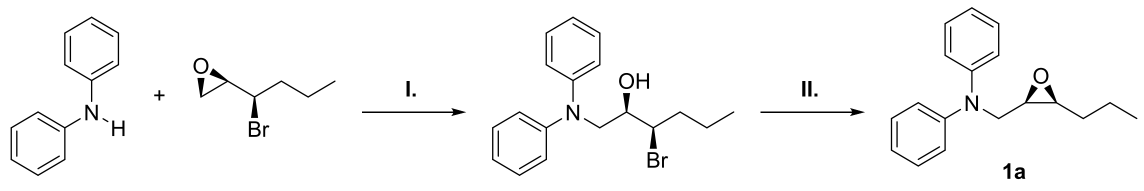

According to GP3: **I.** aminolysis: 1.69 g of diphenylamine (1.00 eq., 10.0 mmol), 1.79 g (2*R*,3*R*)-3-bromo-1,2-epoxyhexane (1.00 eq., 10.0 mmol) and 696 mg SiO_2 are reacted for 72 hours at 80 °C (without solvent). Flash chromatography (SiO_2, Eluent: Ch : Et_2O = 95 : 5) yielded 1.04 g (2*R*,3*R*)-3-bromo-1-(diphenylamino)-hexan-2-ol 30 % as a colorless liquid. **II.** epoxide formation: 880 mg of (2*R*,3*R*)-3-bromo-1-(diphenylamino)-hexan-2-ol (1.00 eq., 2.53 mmol) and 698 mg K_2CO_3 (2.00 eq., 5.05 mmol) are reacted for 1 hour at 40 °C in MeOH. Flash chromatography (SiO_2, Eluent: Ch : Et_2O = 98 : 2) yielded 613 g **1a** (84%) as colorless liquid.

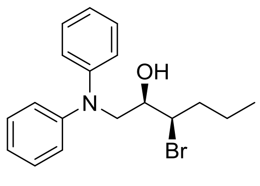

$C_{18}H_{22}BrNO$
348,2840 g/mol

R_f = 0.35 (20% Et_2O in CH), **^{1}H-NMR (300 MHz, C_6D_6, RT): δ [ppm] =** 0.67 (t, *J* = 7.3 Hz, 3H), 1.03-1.21 (m, 1H), 1.31-1.43 (m, 1H), 1.44-1.58 (m, 1H), 1.77 (dtd, *J* = 14.1 Hz, *J* = 9.8 Hz, *J* = 4.5 Hz, 1H), 1.89-1.95 (m,1H), 3.65-3.73 (m, 1H), 3.77-3.81 (m, 2H), 3.92 (ddd, *J* = 9.7 Hz, *J* = 4.5 Hz, *J* = 2.2 Hz, 1H), 6.85 (tt, *J* = 7.4 Hz, *J* = 1.4 Hz, 1H), 7.03-7.15 (m, 8H); **^{13}C-NMR (75 MHz, C_6D_6, RT): δ [ppm] =** 13.4, 21.4, 37.7, 57.4, 61.4, 70.7, 121.9, 122.2, 129.7, 148.8. **IR ṽ [cm^{-1}] =** 408, 419, 498, 543, 594, 694, 747, 1031, 1072, 1204, 1247, 1355, 1493, 1587. **HRMS (ESI):** *m/z* calculated for $C_{18}H_{23}NBrO^+$: 348.0958 u; found: 348.0948 u. **$[\alpha]_D^{20}$**= -14.4° (*c* 0.5, $CHCl_3$).

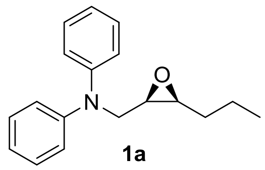

1a
$C_{18}H_{21}NO$
267,3720 g/mol

R_f = 0.8 (20% Et_2O in CH), **^{1}H-NMR (300 MHz, C_6D_6, RT): δ [ppm] =** 0.77 (t, *J* = 7.2 Hz 3H), 1.03-1.33 (m, 4H), 2.58 (ddd, *J* = 6.9 Hz, *J* = 4.9 Hz, *J* = 4.0 Hz, 1H), 3.04 (dt, *J* = 5.6 Hz, *J* = 4.2 Hz, 1H), 3.61 (dd, *J* = 15.7 Hz, *J* = 5.5 Hz, 1H), 3.74 (dd, *J* = 15.7 Hz, *J* = 4.2 Hz, 1H), 6.84-6.87 (m, 2H), 7.04-7.12 (m, 8H); **^{13}C-NMR (75 MHz, C_6D_6, RT): δ [ppm] =** 14.1, 20.3, 30.3, 51.5, 54.6, 56.6, 121.7, 122.0, 129.7, 148.5. **IR ṽ [cm^{-1}] =** 692, 747, 1223, 1249, 1362, 1493, 1589. **HRMS (ESI):** *m/z* calculated for $C_{18}H_{22}NO^+$: 268.1696 u, found: 268.1690 u. **$[\alpha]_D^{20}$**= -88.0 (*c* 0.5, $CHCl_3$).

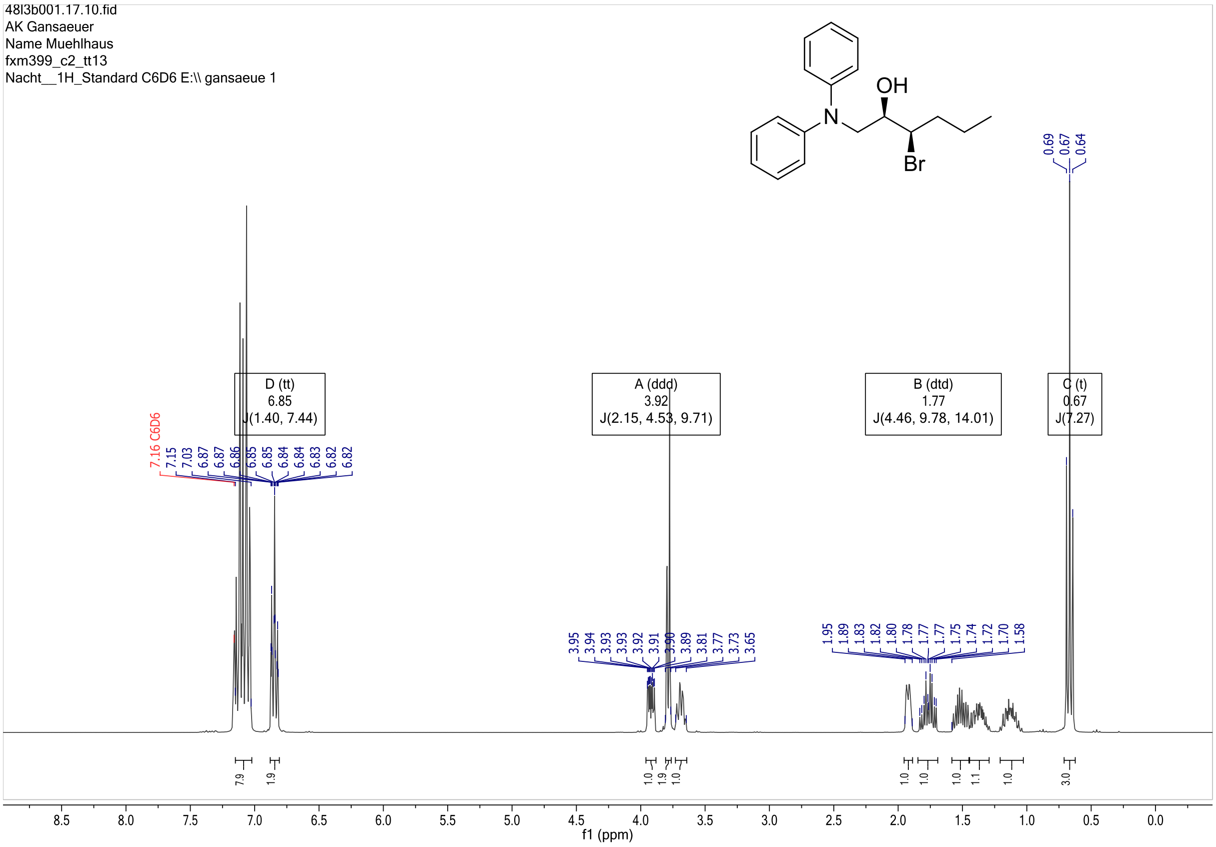

48l3b001.17.10.fid
AK Gansaeuer
Name Muehlhaus
fxm399_c2_tt13
Nacht__1H_Standard C6D6 E:\\ gansaeue 1
OH
N
Br
D (tt)
6.85
J(1.40, 7.44)
A (ddd)
3.92
J(2.15, 4.53, 9.71)
B (dtd)
1.77
J(4.46, 9.78, 14.01)
C (t)
0.67
J(7.27)
7.16 C6D6
f1 (ppm)

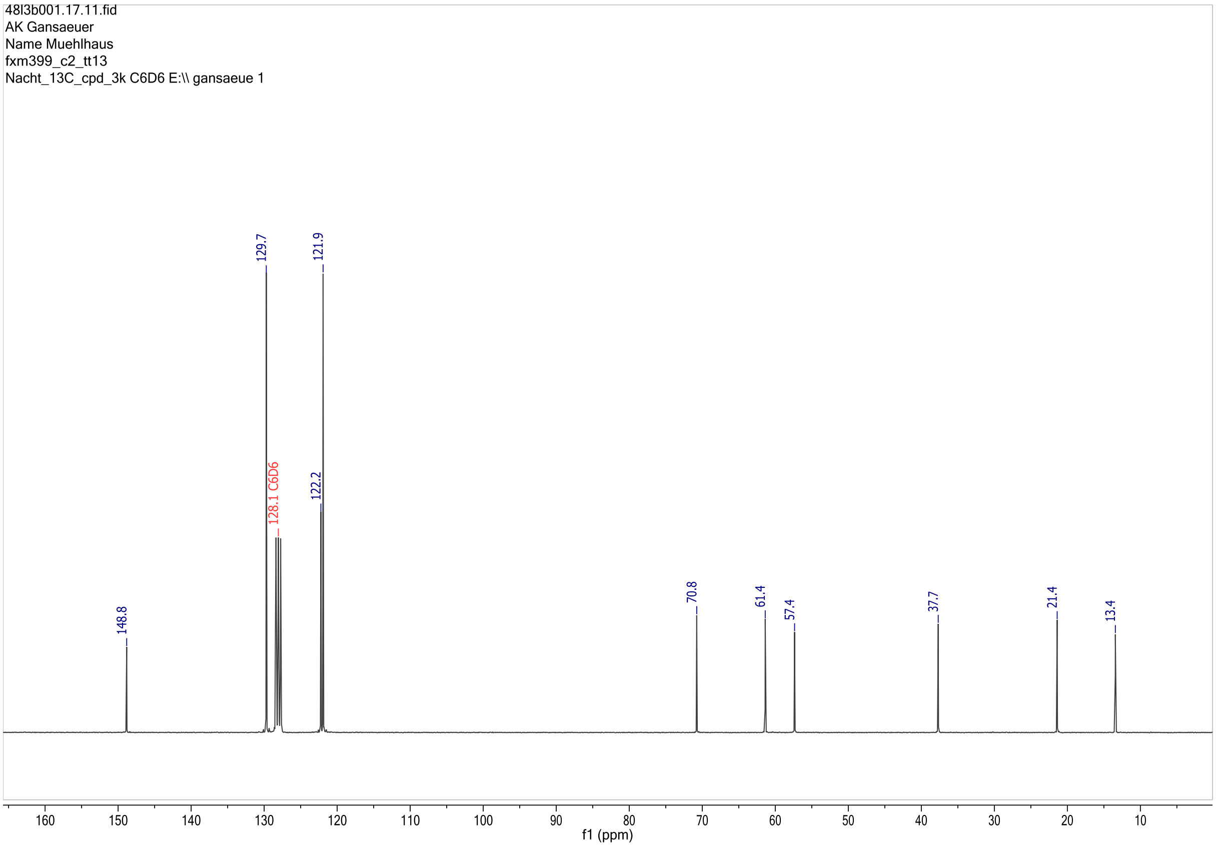

48l3b001.17.11.fid
AK Gansaeuer
Name Muehlhaus
fxm399_c2_tt13
Nacht_13C_cpd_3k C6D6 E:\\ gansaeue 1
148.8
129.7
128.1 C6D6
122.2
121.9
70.8
61.4
57.4
37.7
21.4
13.4
f1 (ppm)

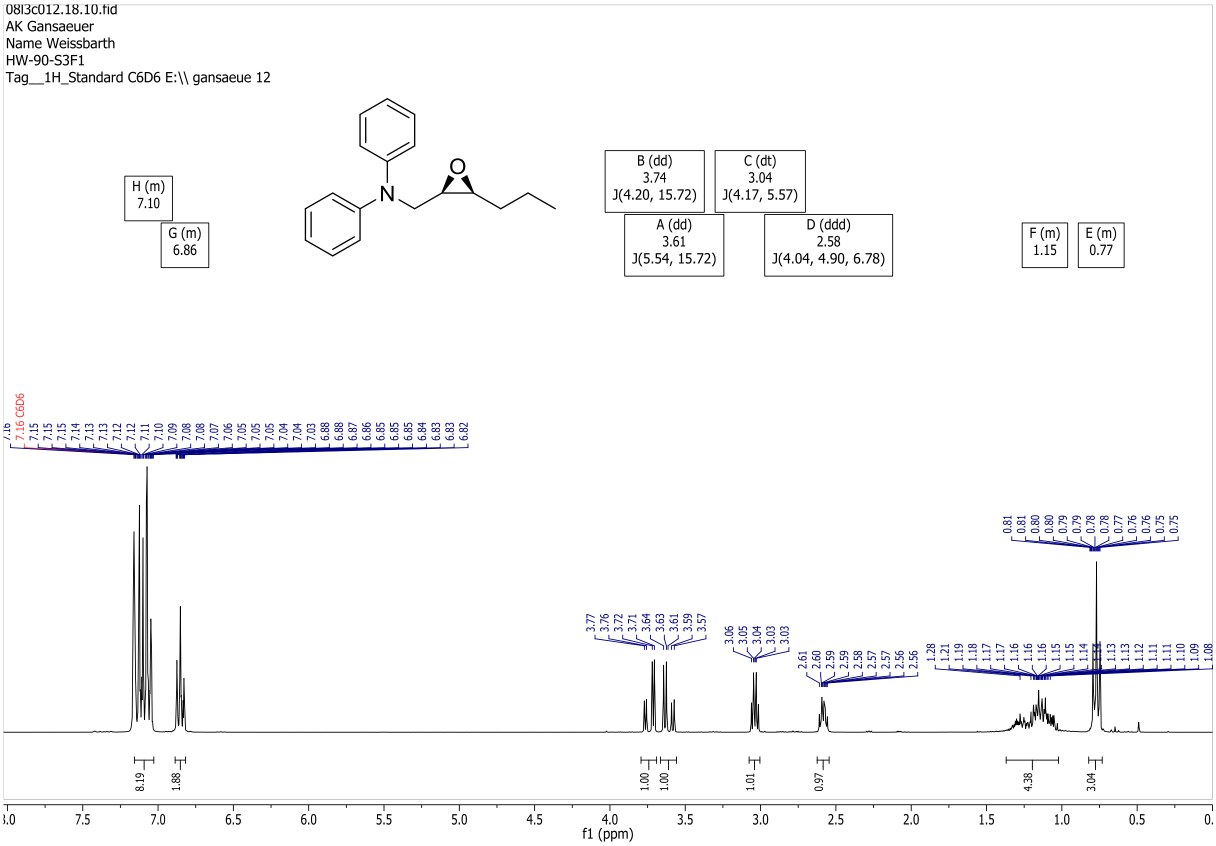
08l3c012.18.10.fid
AK Gansaeuer
Name Weissbarth
HW-90-S3F1
Tag__1H_Standard C6D6 E:\\ gansaeue 12
N
O
H (m) 7.10
G (m) 6.86
B (dd) 3.74 J(4.20, 15.72)
A (dd) 3.61 J(5.54, 15.72)
C (dt) 3.04 J(4.17, 5.57)
D (ddd) 2.58 J(4.04, 4.90, 6.78)
F (m) 1.15
E (m) 0.77
7.16 C6D6
8.19
1.88
1.00
1.00
1.01
0.97
4.38
3.04
f1 (ppm)

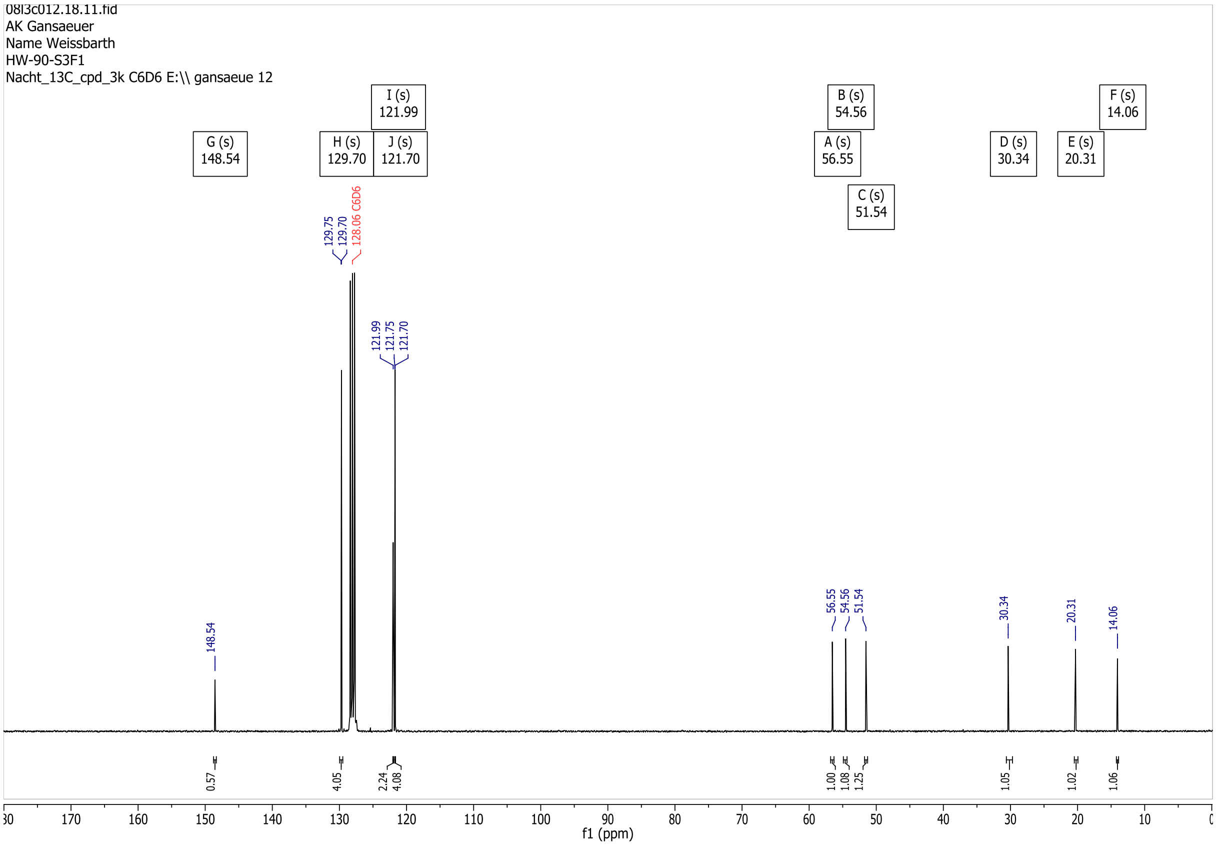
08l3c012.18.11.fid
AK Gansaeuer
Name Weissbarth
HW-90-S3F1
Nacht_13C_cpd_3k C6D6 E:\\ gansaeue 12
G (s) 148.54
H (s) 129.70
I (s) 121.99
J (s) 121.70
B (s) 54.56
A (s) 56.55
C (s) 51.54
D (s) 30.34
E (s) 20.31
F (s) 14.06
128.06 C6D6
f1 (ppm)

3.4.2. Synthesis of *N*-cyclohexyl-*N*-(((*2R,3S*)-3-propyloxiran-2-yl)methyl)aniline (1d).

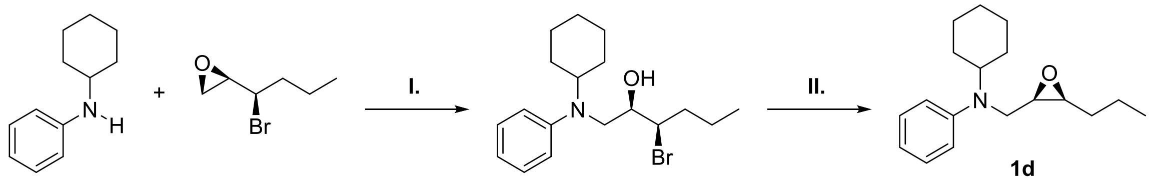

1d

According to GP3b: **I.** aminolysis: 1.75 g of *N*-cyclohexylaniline (1.00 eq., 10.0 mmol), 1.79 g (2*R*,3*R*)-3-bromo-1,2-epoxyhexane (1.00 eq., 10.0 mmol) and 696 mg SiO_2 are reacted for 7 days hours at RT (without solvent). Flash chromatography (SiO_2, Eluent: Ch : Et_2O = 95 : 5) yielded 847 mg (2*R*,3*R*)-3-bromo-1-(cyclohexyl(phenyl)amino)-hexan-2-ol (24%) as a colorless liquid. **II.** epoxide formation: 788 mg of (2*R*,3*R*)-3-bromo-1-(cyclohexyl(phenyl)amino)-hexan-2-ol (1.00 eq., 2.22 mmol) and 614 mg K_2CO_3 (2.00 eq., 4.44 mmol) are reacted for 1 hour at 40 °C in MeOH. Flash chromatography (SiO_2, Eluent: Ch : Et_2O = 98 : 2) yielded 392 g **1d** (65%) as a colorless liquid.

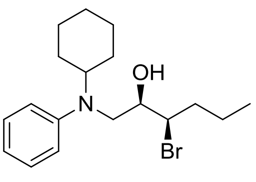

$C_{18}H_{28}BrNO$
354.33 g/mol

R_f = 0.4 (20% Et_2O in CH), **^{1}H-NMR (300 MHz, C_6D_6, RT): δ [ppm] =** 0.70 (t, 3H, *J* = 7.3 Hz), 0.79-1.35 (m, 8H), 1.34-1.48 (m, 1H), 1.45-1.76 (m, 8H), 1.94 (dtd, 1H, *J* = 14.1 Hz, *J* = 9.8 Hz, *J* = 4.7 Hz), 2.26-2.37 (m,1H), 3.18-3.32 (m, 2H)*, 3.55-3.62 (m, 1H), 4.07 (ddd, 1H, *J* = 9.7 Hz, *J* = 4.6 Hz, *J* = 2.0 Hz), 6.84 (tt, 1H, *J* = 7.2 Hz, *J* = 1.1 Hz), 6.93 (d, 1H, *J* = 6.9 Hz), 7.17-7.22 (m, 2H). *signal integrates to 3H caused by impurity. **^{13}C-NMR (75 MHz, C_6D_6, RT): δ [ppm] =** 13.5, 21.5, 26.1, 26.4, 26.5, 27.3, 31.1, 31.3, 38.1, 50.5, 61.5, 62.8, 70.2, 119.4, 120.5, 129.4, 149.6. **IR ṽ [cm^{-1}] =** 504, 691, 868, 1027, 1147, 1254, 1319, 1449, 1502, 1600, 2852, 2926. **HRMS (ESI):** *m/z* calculated for $C_{18}H_{29}NBrO^+$: 354.1427 u; found: 354.1426 u. **$[\alpha]_D^{20}$=** 6.0 (*c* 0.2, $CHCl_3$).

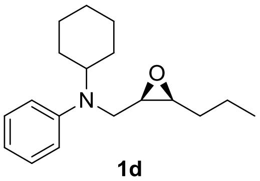

1d
$C_{18}H_{27}NO$
273.41 g/mol

R_f = 0.7 (20% Et_2O in CH), **^{1}H-NMR (500 MHz, C_6D_6, RT): δ [ppm] =** 0.84-0.90 (m, 3H), 0.96 (tt, 2H, *J* = 12.8 Hz, *J* = 3.7 Hz), 1.08-1.53 (m, 10H), 1.60-1.73 (m, 2 H), 1.84 (d, 1H, *J* = 16.7 Hz), 2.68 (dt, 1H, *J* = 6.6 Hz, *J* = 4.4 Hz), 2.85-2.90 (m, 1H), 3.15 (dd, 1H, *J* = 16.3 Hz, *J* = 5.5 Hz), 3.41 (dd, 1H, *J* = 16.2 Hz, *J* = 3.0 Hz), 3.49 (dd, 1H, *J* = 12.0 Hz, *J* = 3.4 Hz), 6.79-6.84 (m, 1H), 6.94 (d, 2H, *J* = 6.1 Hz), 7.25-7.30 (m, 2H). **^{13}C-NMR (125 MHz, C_6D_6, RT): δ [ppm] =** 14.2, 20.6, 26.2, 26.4, 26.5, 30.8, 30.9, 31.5, 44.5, 56.7, 56.8, 57.7, 114.2, 117.5, 129.7, 149.4. **IR ṽ [cm^{-1}] =** 691, 744, 1174, 1234, 1450, 1503, 1595, 2929. **HRMS (ESI):** *m/z* calculated for $C_{18}H_{28}NO^+$: 274.2165 u, found: 274.2158 u. **$[\alpha]_D^{20}$=** -20.9 (*c* 0.1, $CHCl_3$).

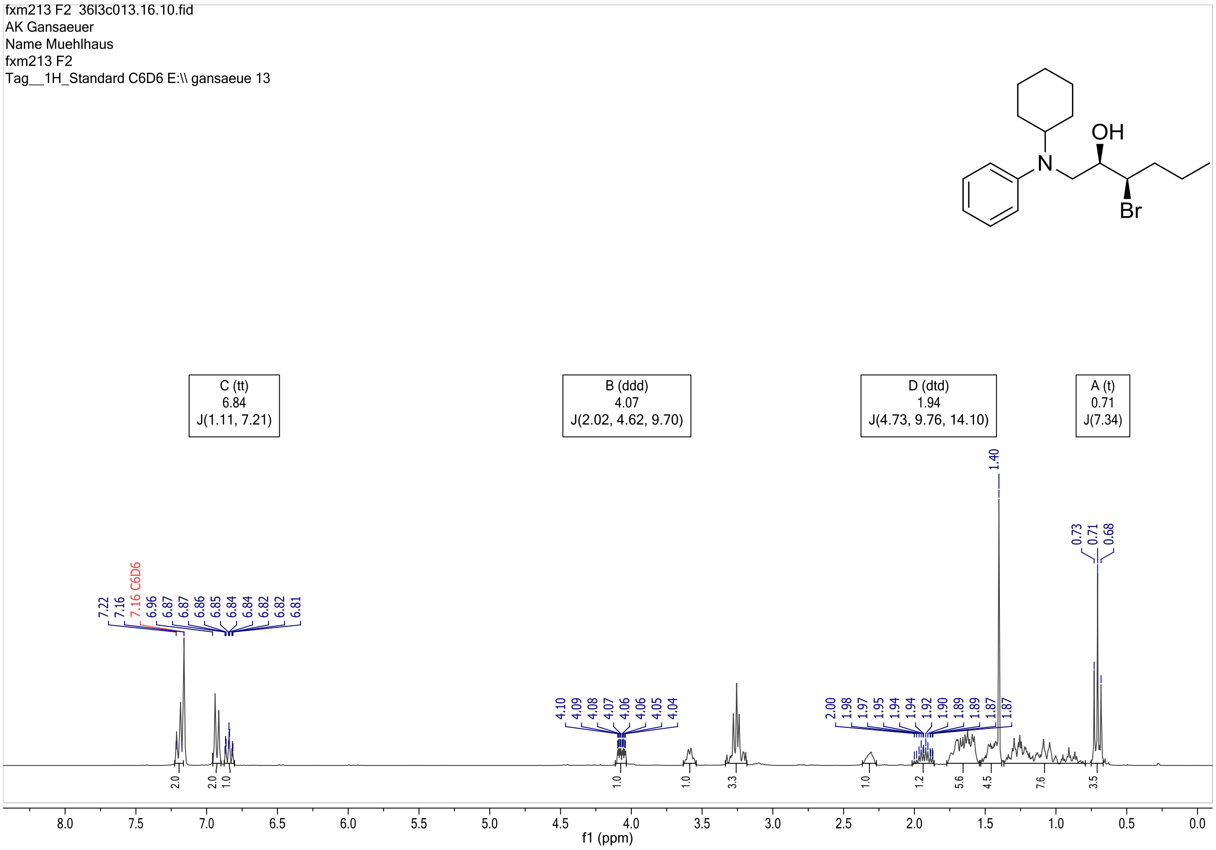
fxm213 F2 36l3c013.16.10.fid
AK Gansaeuer
Name Muehlhaus
fxm213 F2
Tag__1H_Standard C6D6 E:\\ gansaeue 13
OH
N
Br
C (tt)
6.84
J(1.11, 7.21)
B (ddd)
4.07
J(2.02, 4.62, 9.70)
D (dtd)
1.94
J(4.73, 9.76, 14.10)
A (t)
0.71
J(7.34)
7.22
7.16
7.16 C6D6
6.96
6.87
6.87
6.86
6.85
6.84
6.84
6.82
6.82
6.81
4.10
4.09
4.08
4.07
4.06
4.06
4.05
4.04
2.00
1.98
1.97
1.95
1.94
1.94
1.92
1.90
1.89
1.87
1.87
1.40
0.73
0.71
0.68
2.0
2.0
1.0
1.0
1.0
3.3
1.0
1.2
5.6
4.5
7.6
3.5
f1 (ppm)

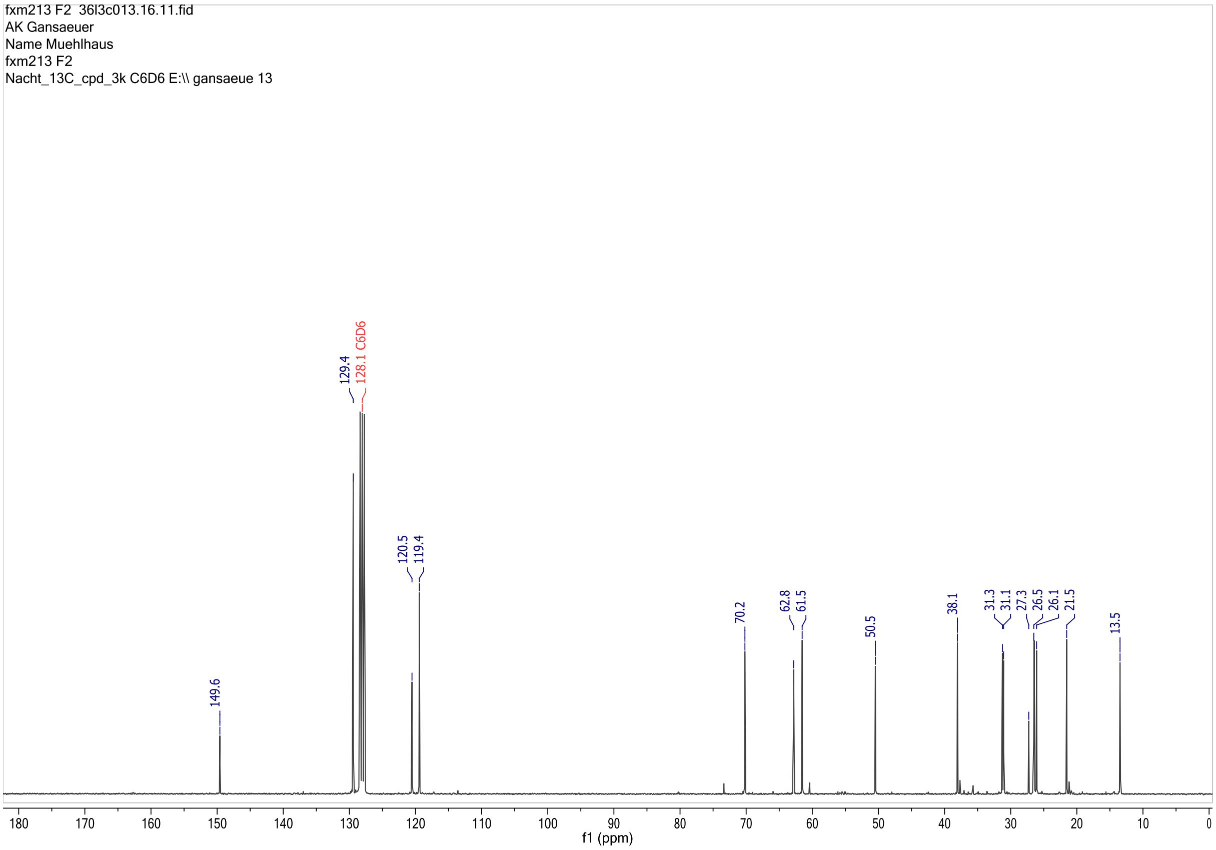
fxm213 F2 36l3c013.16.11.fid
AK Gansaeuer
Name Muehlhaus
fxm213 F2
Nacht_13C_cpd_3k C6D6 E:\\ gansaeue 13
149.6
129.4
128.1 C6D6
120.5
119.4
70.2
62.8
61.5
50.5
38.1
31.3
31.1
27.3
26.5
26.1
21.5
13.5
f1 (ppm)

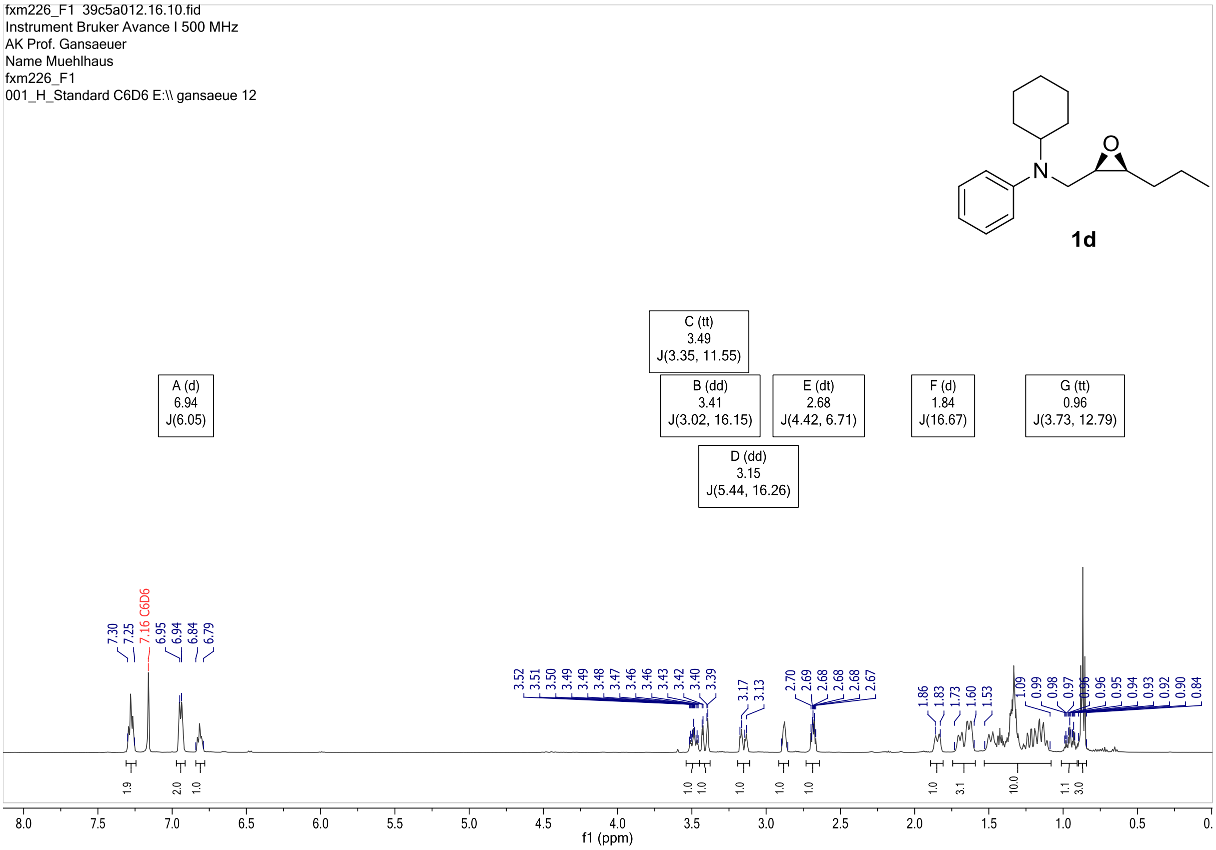
fxm226_F1 39c5a012.16.10.fid
Instrument Bruker Avance I 500 MHz
AK Prof. Gansaeuer
Name Muehlhaus
fxm226_F1
001_H_Standard C6D6 E:\\ gansaeue 12
1d
C (tt) 3.49 J(3.35, 11.55)
A (d) 6.94 J(6.05)
B (dd) 3.41 J(3.02, 16.15)
E (dt) 2.68 J(4.42, 6.71)
F (d) 1.84 J(16.67)
G (tt) 0.96 J(3.73, 12.79)
D (dd) 3.15 J(5.44, 16.26)
7.30 7.25 7.16 C6D6 6.95 6.94 6.84 6.79
3.52 3.51 3.50 3.49 3.49 3.48 3.47 3.46 3.46 3.43 3.42 3.40 3.39 3.17 3.13 2.70 2.69 2.68 2.68 2.68 2.67
1.86 1.83 1.73 1.60 1.53 1.09 0.99 0.98 0.97 0.96 0.96 0.95 0.94 0.93 0.92 0.90 0.84
f1 (ppm)

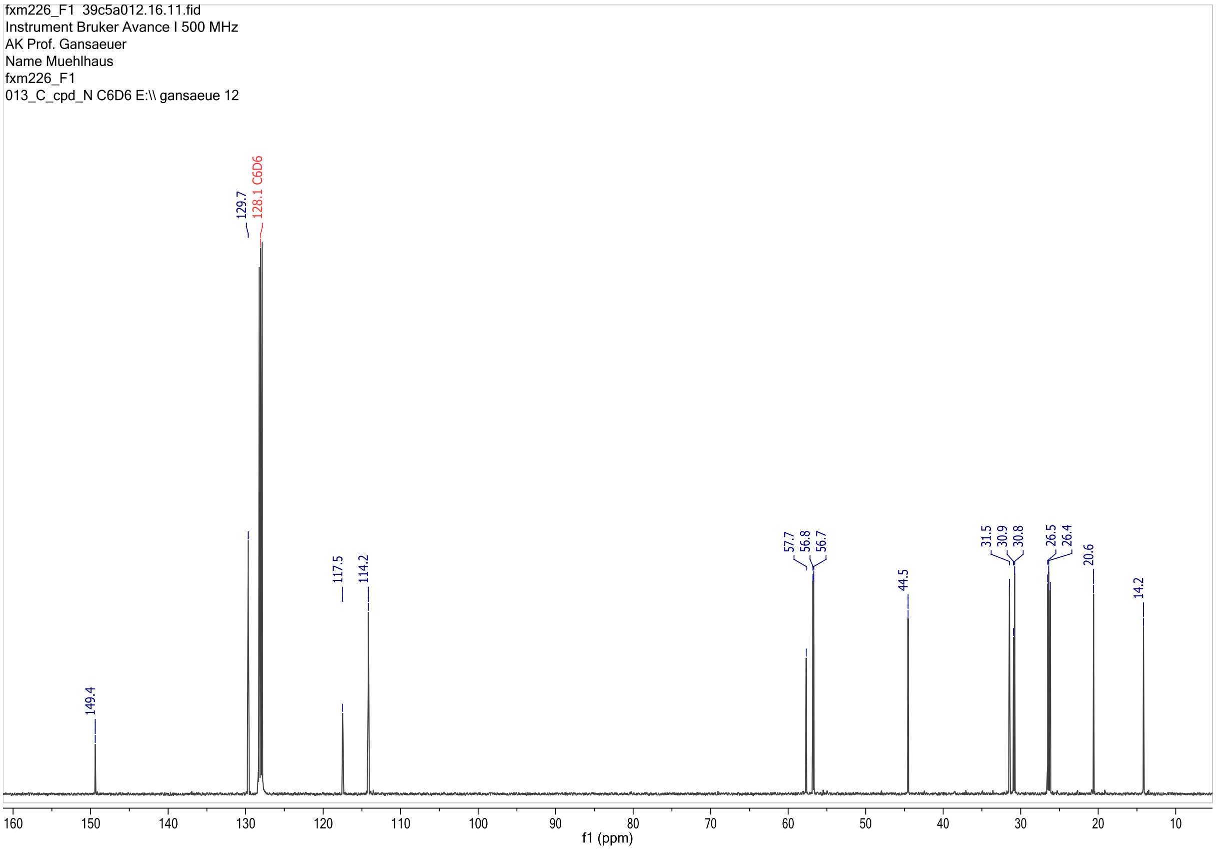
fxm226_F1 39c5a012.16.11.fid
Instrument Bruker Avance I 500 MHz
AK Prof. Gansaeuer
Name Muehlhaus
fxm226_F1
013_C_cpd_N C6D6 E:\\ gansaeue 12
149.4 129.7 128.1 C6D6 117.5 114.2 57.7 56.8 56.7 44.5 31.5 30.9 30.8 26.5 26.4 20.6 14.2
f1 (ppm)

3.4.3. Synthesis of *N*-methyl-*N*-(((*2R,3S*)-3-propyloxiran-2-yl)methyl)aniline (1g).

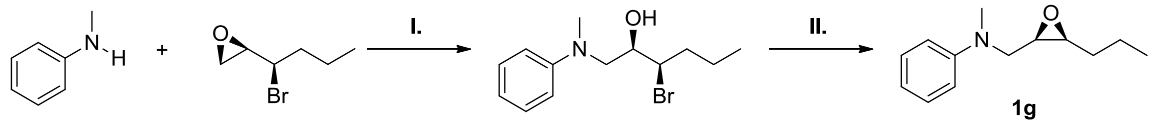

According to GP3b: **I.** aminolysis: 2.38 g of *N*-methylaniline (1.00 eq., 22.2 mmol), 3.63 g (2*R*,3*R*)-3-bromo-1,2-epoxyhexane (1.00 eq., 22.2 mmol) and 1.20 g SiO_2 are reacted for 2 days hours at RT (without solvent). Flash chromatography (SiO_2, Eluent: Ch : Et_2O = 95 : 5) yielded 4.24 g (2*R*,3*R*)-3-bromo-1-(methyl(phenyl)amino)-hexan-2-ol (67%) as a colorless liquid. **II.** epoxide formation: 4.20 g of (2*R*,3*R*)-3-bromo-1-(methyl(phenyl)amino)-hexan-2-ol (1.00 eq., 14.7 mmol) and 4.05 g K_2CO_3 (2.00 eq., 29.3 mmol) are reacted for 1 hour at 40 °C in MeOH. Flash chromatography (SiO_2, Eluent: Ch : Et_2O = 98 : 2) yielded 3.03 g **1g** (100%) as a colorless liquid.

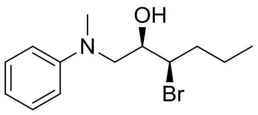

$C_{13}H_{20}BrNO$
286.21 g/mol

R_f = 0.3 (20% Et_2O in CH), **^{1}H-NMR (300 MHz, C_6D_6, RT): δ [ppm]** = 0.70 (t, 3H, *J* = 7.2 Hz), 1.07-1.22 (m, 1H), 1.34-1.58 (m, 2H), 1.68-1.86 (m, 2H), 2.64 (s, 3H), 3.16-3.32 (m, 2H), 3.50-3.59 (m, 1H), 3.83 (ddd, 1H, *J* = 9.8 Hz, *J* = 4.4 Hz, *J* = 2.3 Hz), 6.68-6.73 (m, 2H), 6.75-6.82 (m, 1H), 7.18-7.26 (m, 2H). **^{13}C-NMR (75 MHz, C_6D_6, RT): δ [ppm]** = 13.5, 21.5, 37.8, 39.5, 58.0, 61.9, 71.7, 113.2, 117.6, 129.6, 149.9. **IR (Film) ṽ [cm^{-1}]** = 412, 422, 488, 513, 692, 746, 991, 1034, 1075, 1119, 1204, 1241, 1343, 1449, 1505, 1599, 2959. **HRMS (ESI)**: *m/z* calculated for $C_{13}H_{21}BrNO^{+,}$ $[M+H]^+$: 286.0801 u; found: 286.0786 u. **$[\alpha]_D^{20}$** -23.5 ° (*c* 0.2, $CHCl_3$).

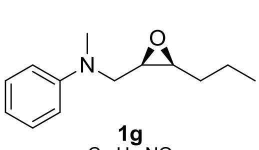

1g
$C_{13}H_{19}NO$
205.30 g/mol

R_f = 0.5 (20% Et_2O in CH); **^{1}H-NMR (300 MHz, C_6D_6, RT): δ [ppm]** = 0.84 (t, 3H, *J* = 7.0 Hz), 1.18-1.42 (m, 4H), 2.57 (ddd, 1H, *J* = 6.3 Hz, *J* = 4.9 Hz, *J* = 3.0 Hz), 2.69 (s, 3H), 2.83 (ddd, 1H, *J* = 6.2 Hz, *J* = 4.0 Hz, *J* = 4.0 Hz), 3.06 (dd, 1H, *J* = 15.4 Hz, *J* = 6.4 Hz), 3.29 (dd, 1H, *J* = 15.4 Hz, *J* = 3.9 Hz), 6.74 (d, 2H, *J* = 8.8 Hz); 6.80 (tt, 1H, *J* = 7.3 Hz, *J* = 1.1 Hz), 7.24 (ddt, 2H, *J* = 8.7 Hz, *J* = 7.1 Hz, *J* = 0.9 Hz). **^{13}C-NMR (75 MHz, C_6D_6, RT)**: δ [ppm] = 14.1, 20.4, 30.4, 38.6, 51.8, 54.6, 55.7, 112.6, 113.5, 117.5, 129.5, 150.1. **IR (Film) ṽ [cm^{-1}]** = 406, 512, 690, 746, 775, 827, 860, 957, 990, 1034, 1120, 1208, 1244, 1365, 1451, 1504, 1599, 2871, 2959; **HRMS (ESI)**: *m/z* calculated for $C_{13}H_{20}NO^+$ $[M+H]^+$: 206.1539 u; found: 206.1540 u. **$[\alpha]_D^{20}$** – 18.2 (*c* 0.5, $CHCl_3$), deterination of e.r. by HPLC: *KNAUER Eurocel* 01; *n*-hexane/2-propanol (90:10); flowrate 1.0 mL/min; t_R = 7.9 min (major, *2R3S*), t_R = 9.2 min (minor, *2S3R*); *e.r.* = > 99 : < 1.

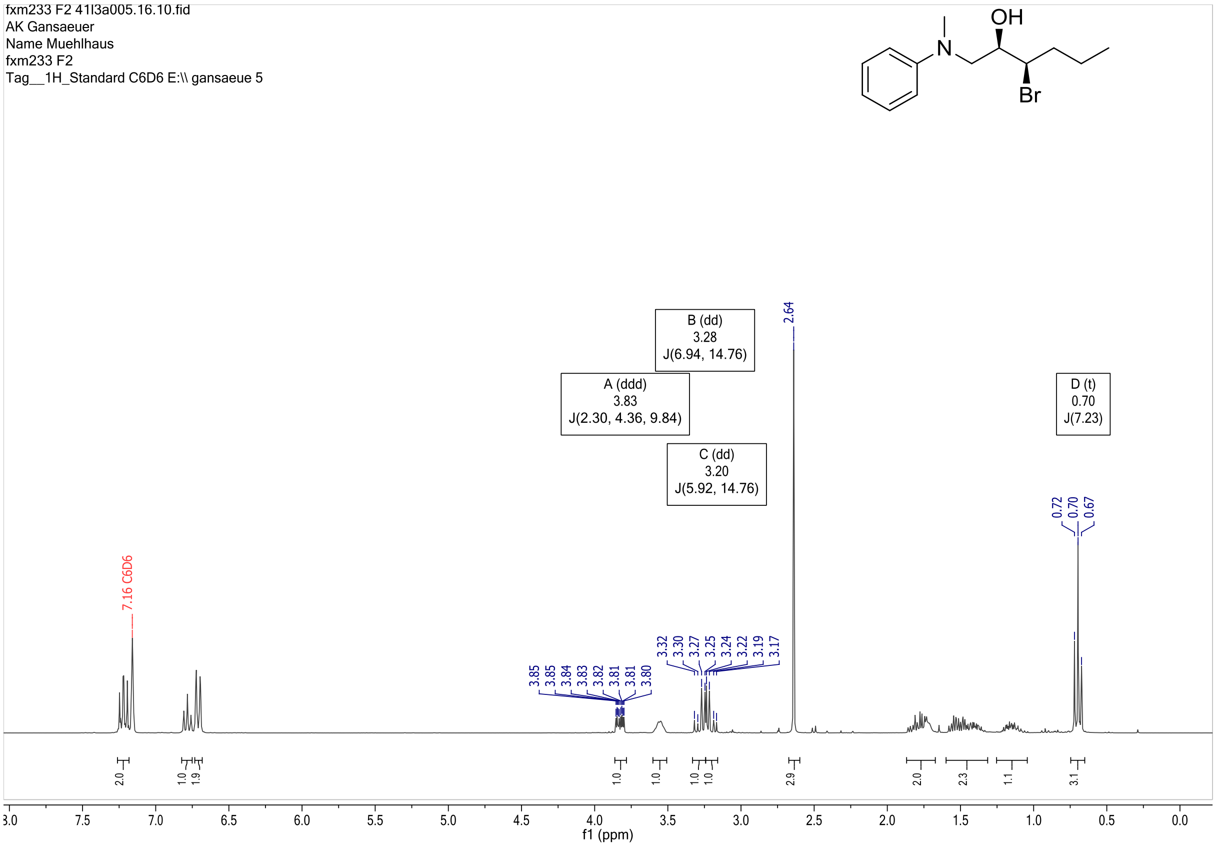
fxm233 F2 41l3a005.16.10.fid
AK Gansaeuer
Name Muehlhaus
fxm233 F2
Tag__1H_Standard C6D6 E:\\ gansaeue 5
OH
N
Br
B (dd)
3.28
J(6.94, 14.76)
A (ddd)
3.83
J(2.30, 4.36, 9.84)
C (dd)
3.20
J(5.92, 14.76)
D (t)
0.70
J(7.23)
2.64
7.16 C6D6
3.85
3.85
3.84
3.83
3.82
3.81
3.81
3.80
3.32
3.30
3.27
3.25
3.24
3.22
3.19
3.17
0.72
0.70
0.67
2.0
1.0
1.9
1.0
1.0
1.0
1.0
2.9
2.0
2.3
1.1
3.1
f1 (ppm)

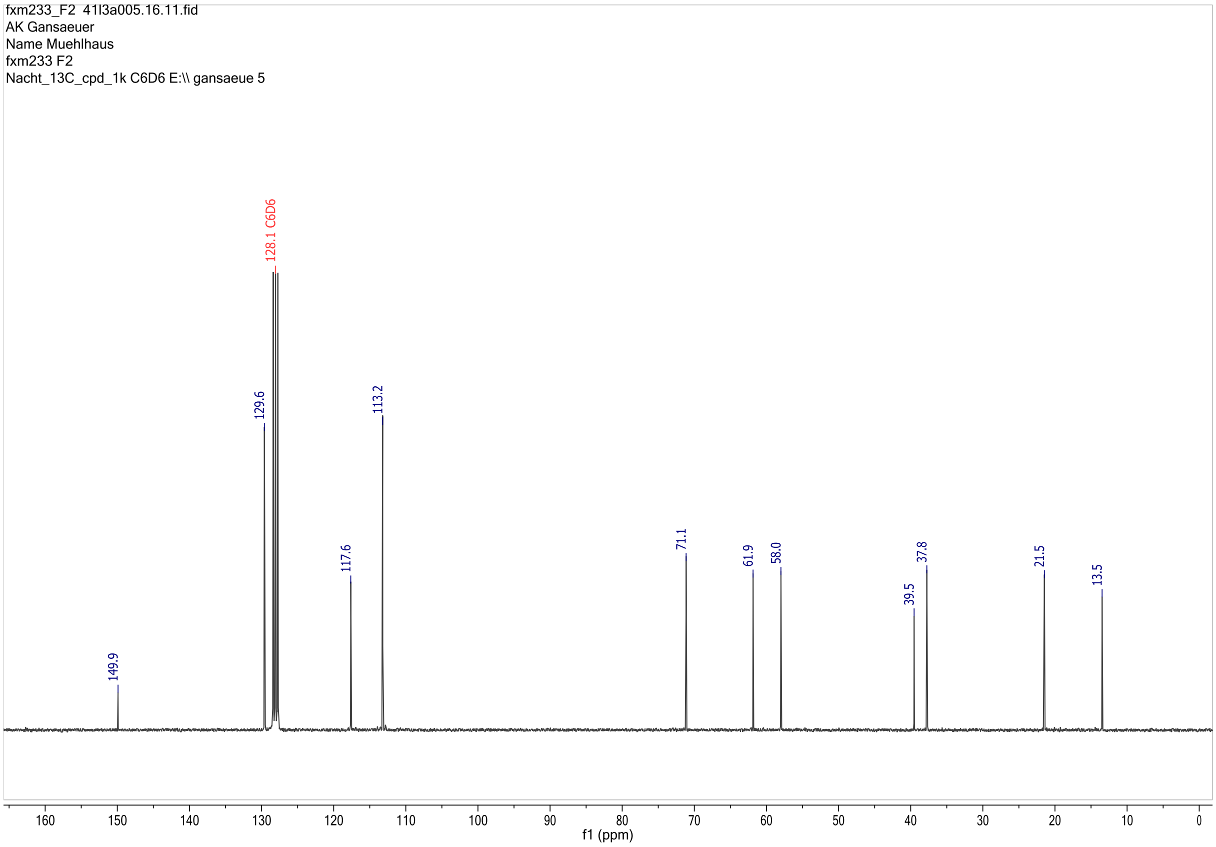
fxm233_F2 41l3a005.16.11.fid
AK Gansaeuer
Name Muehlhaus
fxm233 F2
Nacht_13C_cpd_1k C6D6 E:\\ gansaeue 5
128.1 C6D6
129.6
113.2
117.6
149.9
71.1
61.9
58.0
39.5
37.8
21.5
13.5
f1 (ppm)

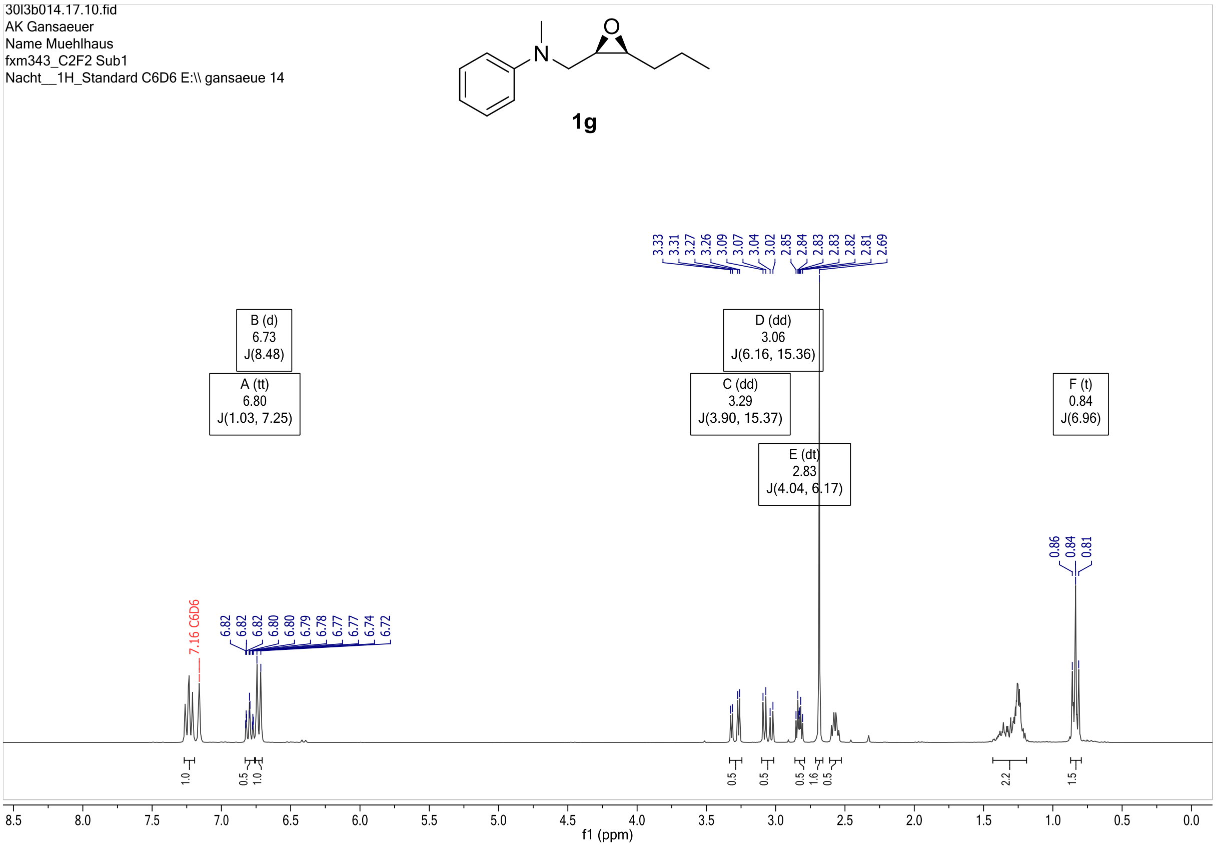
30l3b014.17.10.fid
AK Gansaeuer
Name Muehlhaus
fxm343_C2F2 Sub1
Nacht__1H_Standard C6D6 E:\\ gansaeue 14
1g
3.33 3.31 3.27 3.26 3.09 3.07 3.04 3.02 2.85 2.84 2.83 2.83 2.82 2.81 2.69
B (d) 6.73 J(8.48)
A (tt) 6.80 J(1.03, 7.25)
D (dd) 3.06 J(6.16, 15.36)
C (dd) 3.29 J(3.90, 15.37)
E (dt) 2.83 J(4.04, 6.17)
F (t) 0.84 J(6.96)
0.86 0.84 0.81
7.16 C6D6
6.82 6.82 6.82 6.80 6.80 6.79 6.78 6.77 6.77 6.74 6.72
1.0 0.5 1.0 0.5 0.5 0.5 1.6 0.5 2.2 1.5
f1 (ppm)

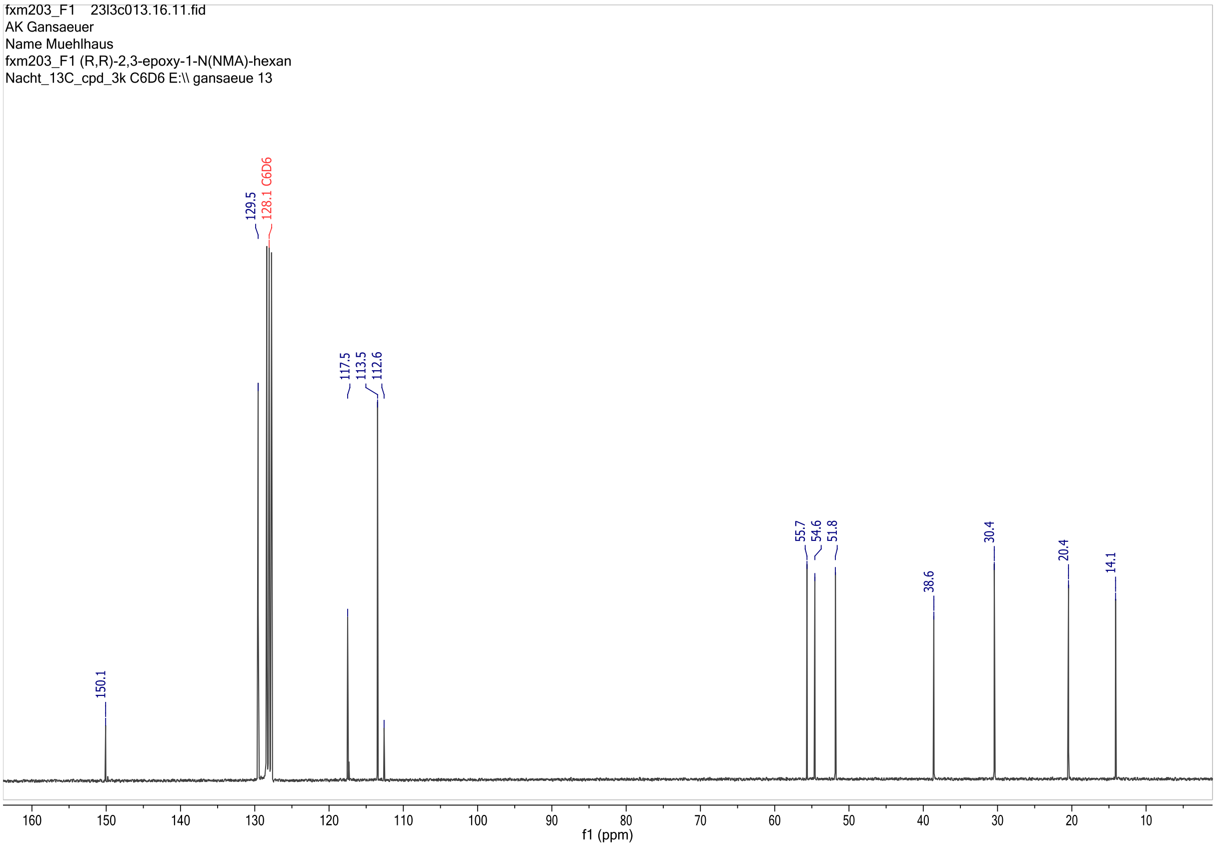
fxm203_F1 23l3c013.16.11.fid
AK Gansaeuer
Name Muehlhaus
fxm203_F1 (R,R)-2,3-epoxy-1-N(NMA)-hexan
Nacht_13C_cpd_3k C6D6 E:\\ gansaeue 13
129.5
128.1 C6D6
117.5 113.5 112.6
150.1
55.7 54.6 51.8
38.6
30.4
20.4
14.1
f1 (ppm)

3.4.4. Synthesis of 4-chloro-*N*-methyl-*N*-(((*2R,3S*)-3-propyloxiran-2-yl)methyl)aniline (1m).

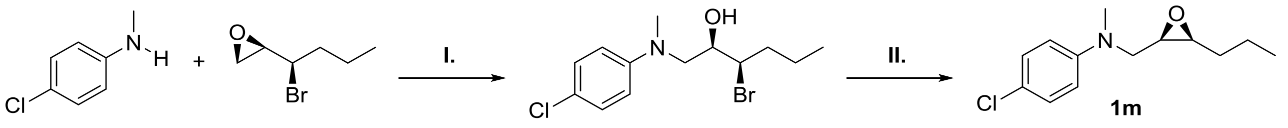

According to GP3b: **I.** aminolysis: 389 mg of 4-chloro-*N*-methylaniline (1.10 eq., 2.75 mmol), 448 mg (2*R*,3*R*)-3-bromo-1,2-epoxyhexane (1.00 eq., 2.50 mmol) and 250 mg SiO_2 are reacted for 2 days hours at RT (without solvent). Flash chromatography (SiO_2, Eluent: Ch : Et_2O = 95 : 5) yielded 360 mg (2*R*,3*R*)-3-bromo-1-(cyclohexyl(4-chloro-phenyl)amino)-hexan-2-ol (45%) as a colorless liquid. **II.** epoxide formation: 1.64 g of (2*R*,3*R*)-3-bromo-1-(cyclohexyl(4-chloro-phenyl)amino)-hexan-2-ol (1.00 eq., 5.13 mmol) and 1.42 g K_2CO_3 (2.00 eq., 10.3 mmol) are reacted for 1 hour at 40 °C in MeOH. Flash chromatography (SiO_2, Eluent: Ch : Et_2O = 98 : 2) yielded 943 mg **1m** (77%) as a colorless liquid.

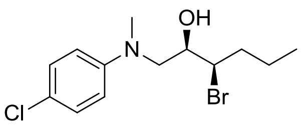

$C_{13}H_{19}BrClNO$
354.33 g/mol

R_f = 0.3 (20% Et_2O in CH), **^{1}H-NMR (500 MHz, C_6D_6, RT): δ [ppm] =** 0.71 (ddt, 3H, *J* = 7.3 Hz, *J* = 4.1 Hz, *J* = 1.9 Hz), 1.15 (ddddd, 1H, *J* = 13.1 Hz, *J* = 9.9 Hz, *J* = 7.1 Hz, *J* = 5.3 Hz, *J* = 2.7 Hz), 1.41 (dtdd, 1H, *J* = 17.3 Hz, *J* = 7.1 Hz, *J* = 5.0 Hz, *J* = 2.3 Hz), 1.50 (dddd, 1H, *J* = 13.8 Hz, *J* = 6.5 Hz, *J* = 5.2 Hz *J* = 3.9 Hz), 1.71-1.80 (m, 1H), 2.50 (s, 3H), 3.05-3.16 (m, 2H), 3.40-3.47 (m, 1H), 3.74 (dtd, 1H, *J* = 9.8 Hz, *J* = 4.3 Hz, *J* = 2.1 Hz), 6.38-6.43 (m, 2H), 7.16-7.19* (m, 2H). **^{13}C-NMR (75 MHz, C_6D_6, RT): δ [ppm] =** 13.5, 21.4, 37.6, 39.5, 57.8, 61.6, 71.0, 114.1, 122.6, 129.4, 148.3. **IR ṽ [cm^{-1}] =** 505, 627, 807, 1076, 1100, 1190, 1239, 1364, 1498, 1596. **HRMS (ESI):** *m/z* calculated for $C_{13}H_{20}BrClNO^+$: 320.0419 u; found: 320.0411 u. **$[\alpha]_D^{20}$**= – 28.4 (*c* 1.0, $CHCl_3$).

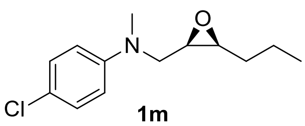

1m
$C_{13}H_{18}ClNO$
239.74 g/mol

R_f = 0.8 (40% EE in CH), **^{1}H-NMR (500 MHz, C_6D_6, RT): δ [ppm] =** 0.84 (t, 3H, *J* = 7.1 Hz), 1.14-1.43 (m, 4H), 2.53 (s, 3H), 2.54-2.59 (m, 1H), 2.70 (dt, 1H, *J* = 6.4 Hz, *J* = 4.0 Hz, *J* = 1.7 Hz), 2.89 (dd, 1H, *J* = 15.4 Hz, *J* = 6.4 Hz), 3.12 (dd, 1H, *J* = 15.4 Hz, *J* = 3.8 Hz), 6.38-6.44 (m, 2H), 7.14-7.20 (m, 2H). **^{13}C-NMR (125 MHz, C_6D_6, RT): δ [ppm] =** 14.1, 20.4, 30.8, 38.5, 51.8, 54.3, 55.6, 114.4, 122.2, 129.3, 148.5. **IR ṽ [cm^{-1}] =** 507, 629, 764, 808, 957, 1097, 1120, 1190, 1206, 1243, 1368, 1455, 1498, 1596, 2960. **HRMS (ESI):** *m/z* calculated for $C_{13}H_{19}ClNO^+$: 240.1150 u, found: 240.1147 u. **$[\alpha]_D^{20}$**= -12.6 (*c* 0.5, $CHCl_3$).

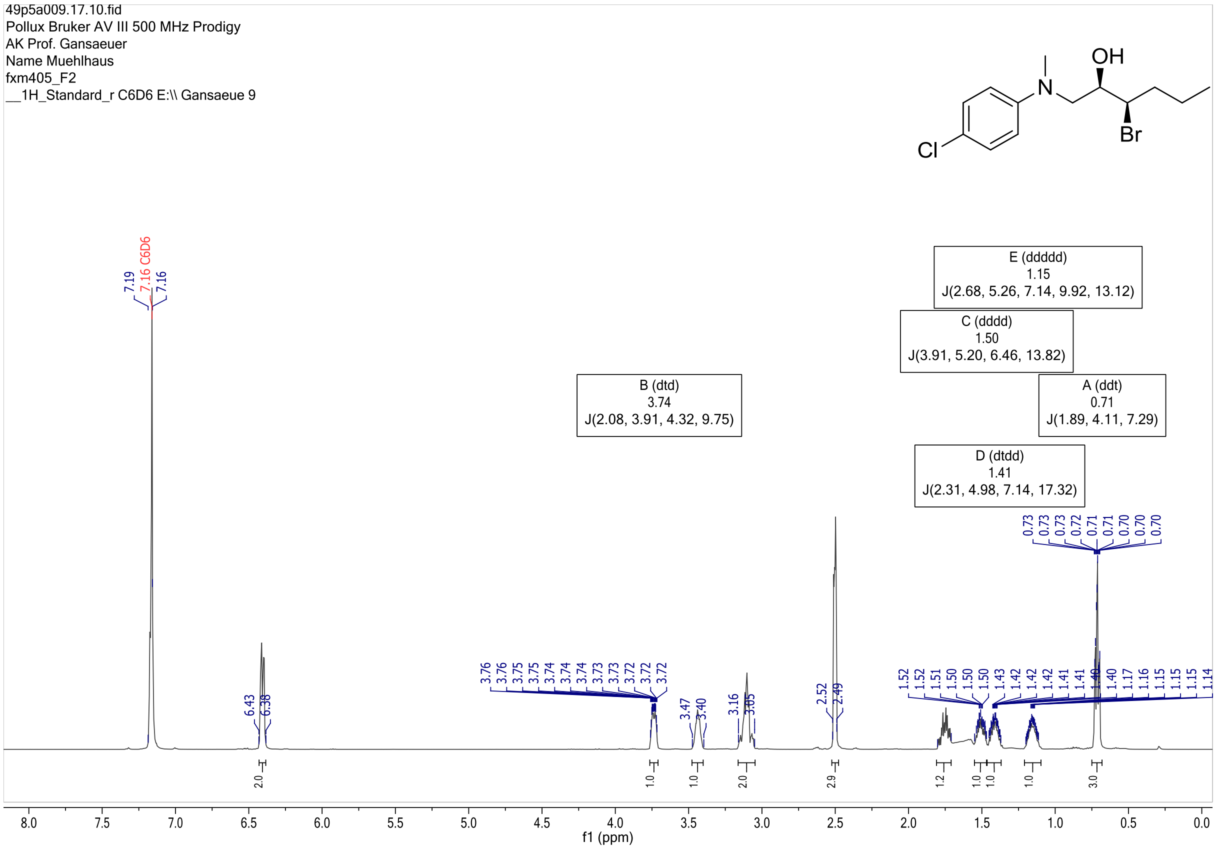
49p5a009.17.10.fid
Pollux Bruker AV III 500 MHz Prodigy
AK Prof. Gansaeuer
Name Muehlhaus
fxm405_F2
__1H_Standard_r C6D6 E:\\ Gansaeue 9
OH
N
Br
Cl
7.19
7.16 C6D6
7.16
E (ddddd)
1.15
J(2.68, 5.26, 7.14, 9.92, 13.12)
C (dddd)
1.50
J(3.91, 5.20, 6.46, 13.82)
B (dtd)
3.74
J(2.08, 3.91, 4.32, 9.75)
A (ddt)
0.71
J(1.89, 4.11, 7.29)
D (dtdd)
1.41
J(2.31, 4.98, 7.14, 17.32)
f1 (ppm)

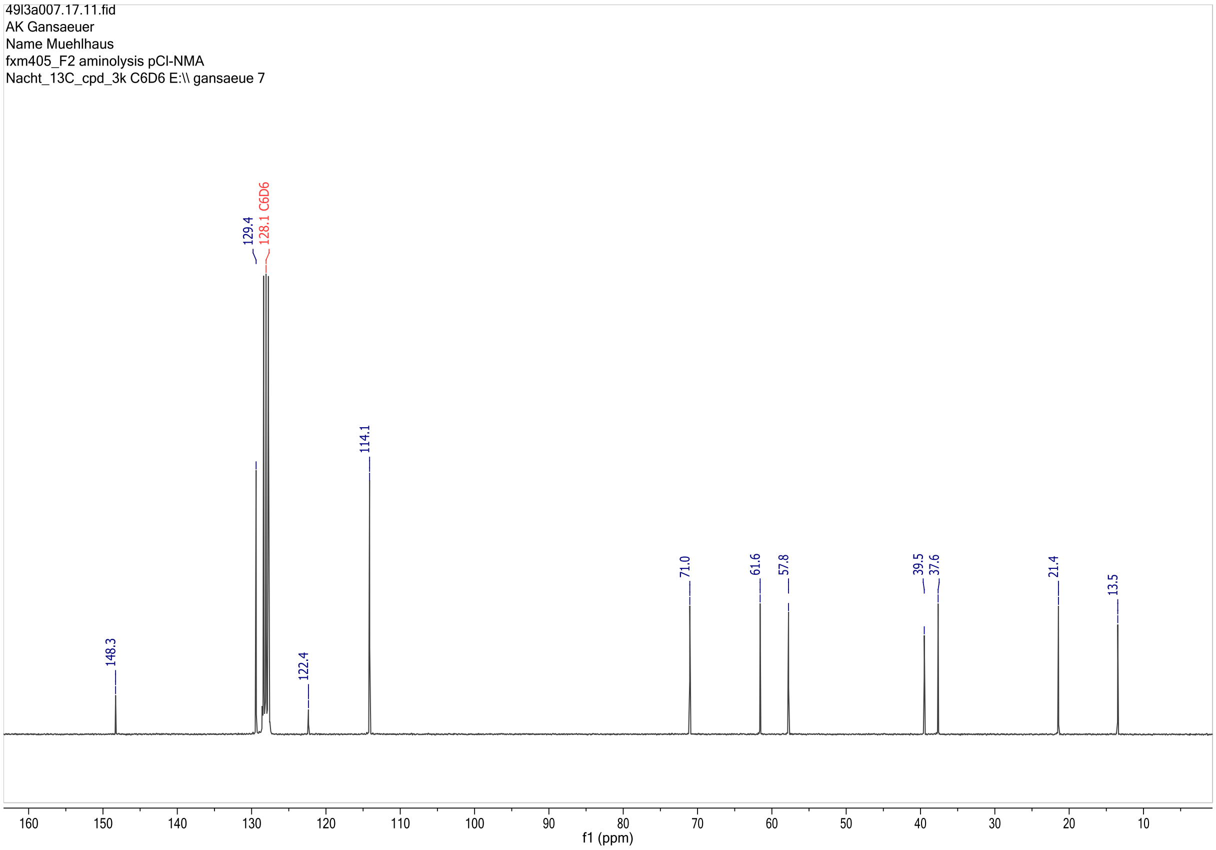
49l3a007.17.11.fid
AK Gansaeuer
Name Muehlhaus
fxm405_F2 aminolysis pCl-NMA
Nacht_13C_cpd_3k C6D6 E:\\ gansaeue 7
129.4
128.1 C6D6
114.1
148.3
122.4
71.0
61.6
57.8
39.5
37.6
21.4
13.5
f1 (ppm)

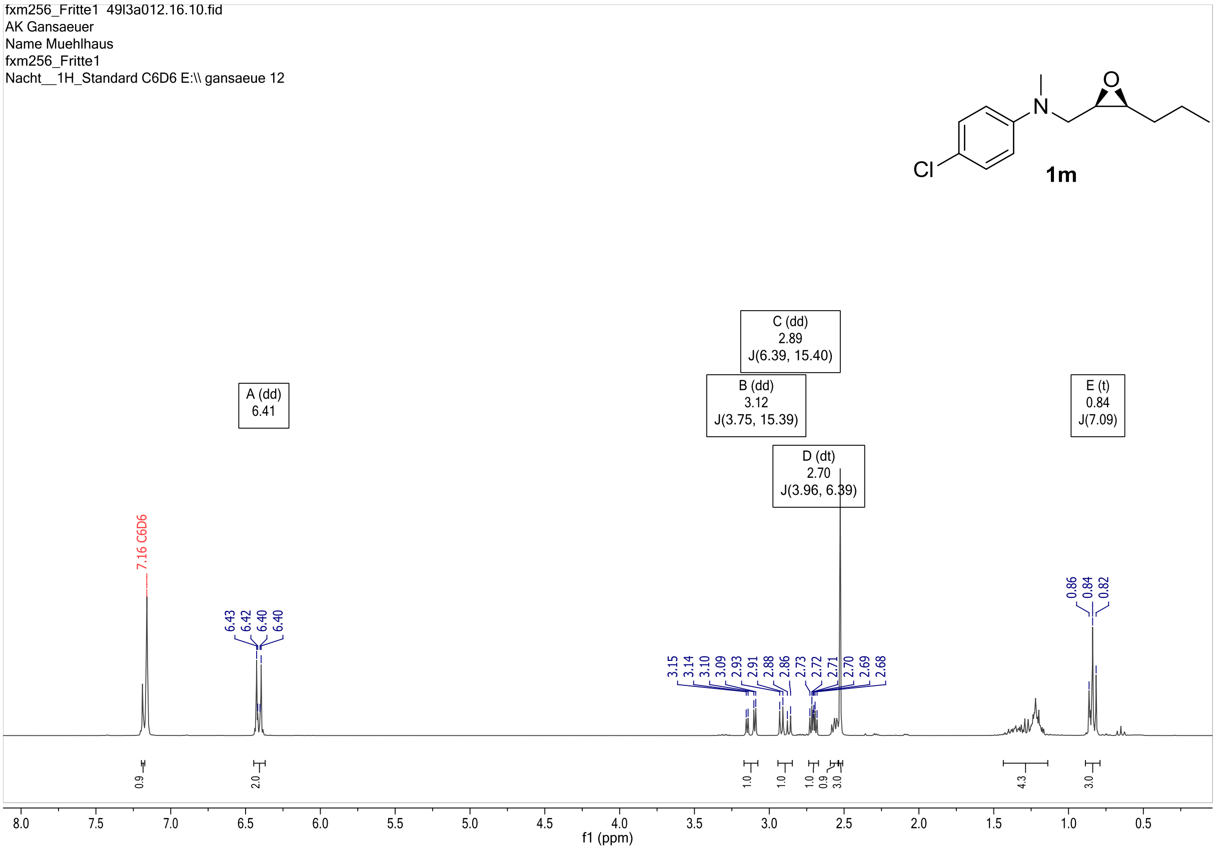

fxm256_Fritte1 49l3a012.16.10.fid
AK Gansaeuer
Name Muehlhaus
fxm256_Fritte1
Nacht__1H_Standard C6D6 E:\\ gansaeue 12
Cl
N
O
1m
C (dd)
2.89
J(6.39, 15.40)
A (dd)
6.41
B (dd)
3.12
J(3.75, 15.39)
E (t)
0.84
J(7.09)
D (dt)
2.70
J(3.96, 6.39)
7.16 C6D6
6.43
6.42
6.40
6.40
3.15
3.14
3.10
3.09
2.93
2.91
2.88
2.86
2.73
2.72
2.71
2.70
2.69
2.68
0.86
0.84
0.82
0.9
2.0
1.0
1.0
1.0
0.9
3.0
4.3
3.0
8.0
7.5
7.0
6.5
6.0
5.5
5.0
4.5
4.0
3.5
3.0
2.5
2.0
1.5
1.0
0.5
f1 (ppm)

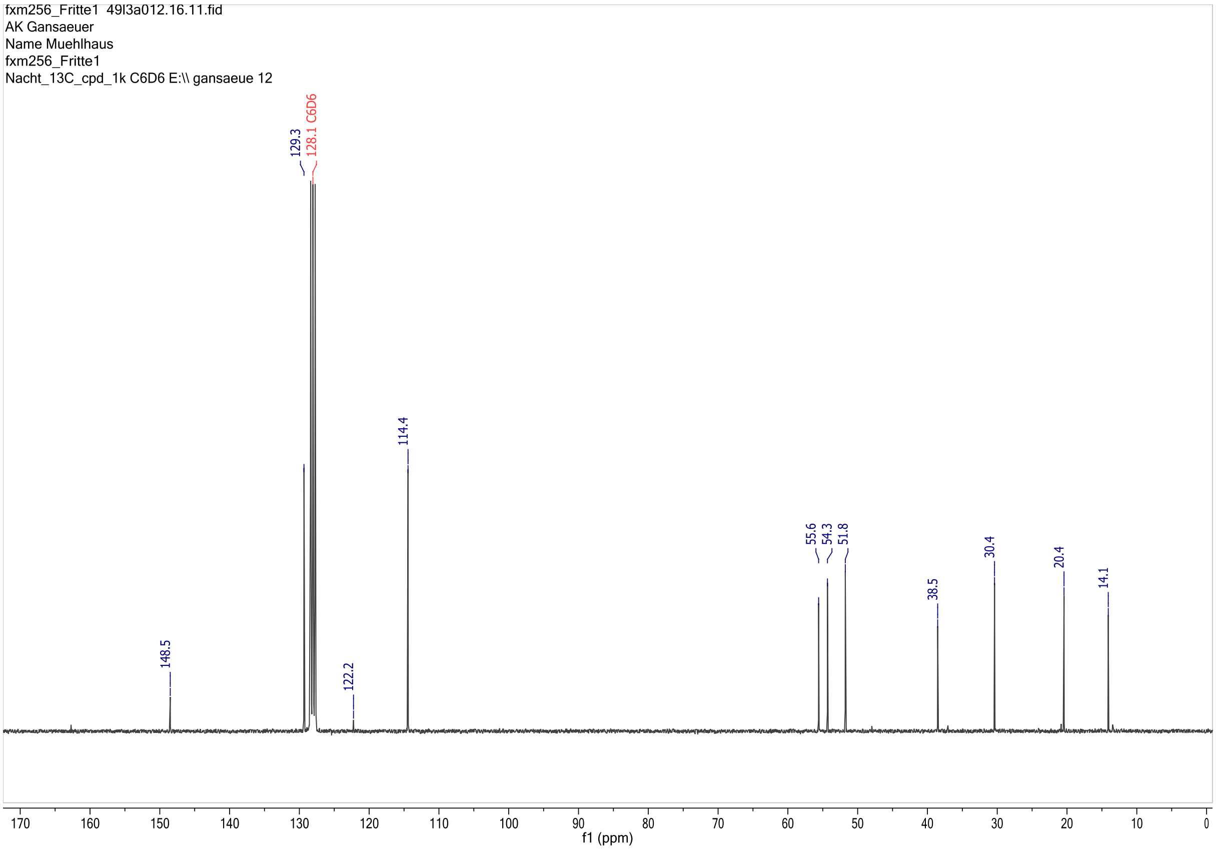

fxm256_Fritte1 49l3a012.16.11.fid
AK Gansaeuer
Name Muehlhaus
fxm256_Fritte1
Nacht_13C_cpd_1k C6D6 E:\\ gansaeue 12
129.3
128.1 C6D6
114.4
148.5
122.2
55.6
54.3
51.8
38.5
30.4
20.4
14.1
170
160
150
140
130
120
110
100
90
80
70
60
50
40
30
20
10
0
f1 (ppm)

3.5.1. Synthesis of 4-iodo-N-methyl-N-(((2R,3S)-3-propyloxiran-2-yl)methyl)aniline (1o).

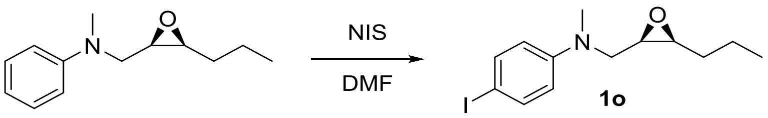

513 mg of **1g** (1.00 eq., 2.50 mmol) are dissolved in 3.0 mL DMF and cooled to 0 °C. Under vigorous stirring 563 mg *N*-iodosuccinimide NIS (1.00 eq., 2.50 mmol) is added in three portions after 0, 15 and 30 minutes. The mixture is stirred for 1 h at 0 °C and for 1 h at rt. The reaction is quenched by addition of 5 mL saturated $Na_2S_2O_3$-solution and 10 mL cyclohexane are added. After phase separation the aqueous-solution is extracted with cyclohexane (4 * 15 mL cyclohexane). The combined organic solutions are washed with H_2O (2 * 5 mL) and brine (1 * 5 mL) and dried under Na_2SO_4. After removal of solvents under reduced pressure, the crude product is purified by flash chromatography (SiO_2, Eluent: CH : MTBE = 95 : 5) to yield 643 mg **1o** (78%) as a colorless solution.

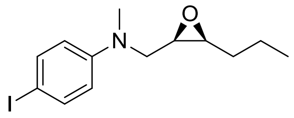

1o
$C_{13}H_{18}INO$
331.19 g/mol

R_f = 0.8 (40% EE in CH), **^{1}H-NMR (300 MHz, C_6D_6, RT): δ [ppm] =** 0.83 (t, 3H, *J* = 7.1 Hz), 1.12-1.45 (m, 4H), 2.49 (s, 3H), 2.68 (dt, 1H, *J* = 6.4 Hz, *J* = 4.0 Hz), 2.86 (dd, 1H, *J* = 15.4 Hz, *J* = 6.4 Hz), 3.10 (dd, 1H, *J* = 15.4 Hz, *J* = 3.7 Hz), 6.22-6.29 (m, 2H), 7.44-7.51 (m, 2H). **^{13}C-NMR (75 MHz, C_6D_6, RT): δ [ppm] =** 14.1, 20.4, 30.4, 38.3, 51.5, 54.3, 55.6, 78.3, 115.5, 138.1, 149.3. **IR ṽ [cm^{-1}] =** 505, 754, 803, 957, 1119, 1203, 1243, 1314, 1368, 1456, 1496, 1587, 2959. **HRMS (ESI):** *m/z* calculated for $C_{13}H_{19}INO^+$: 332.0506 u, found: 332.0503 u. **$[\alpha]_D^{20}$=** -10.8 (*c* 0.5, $CHCl_3$).

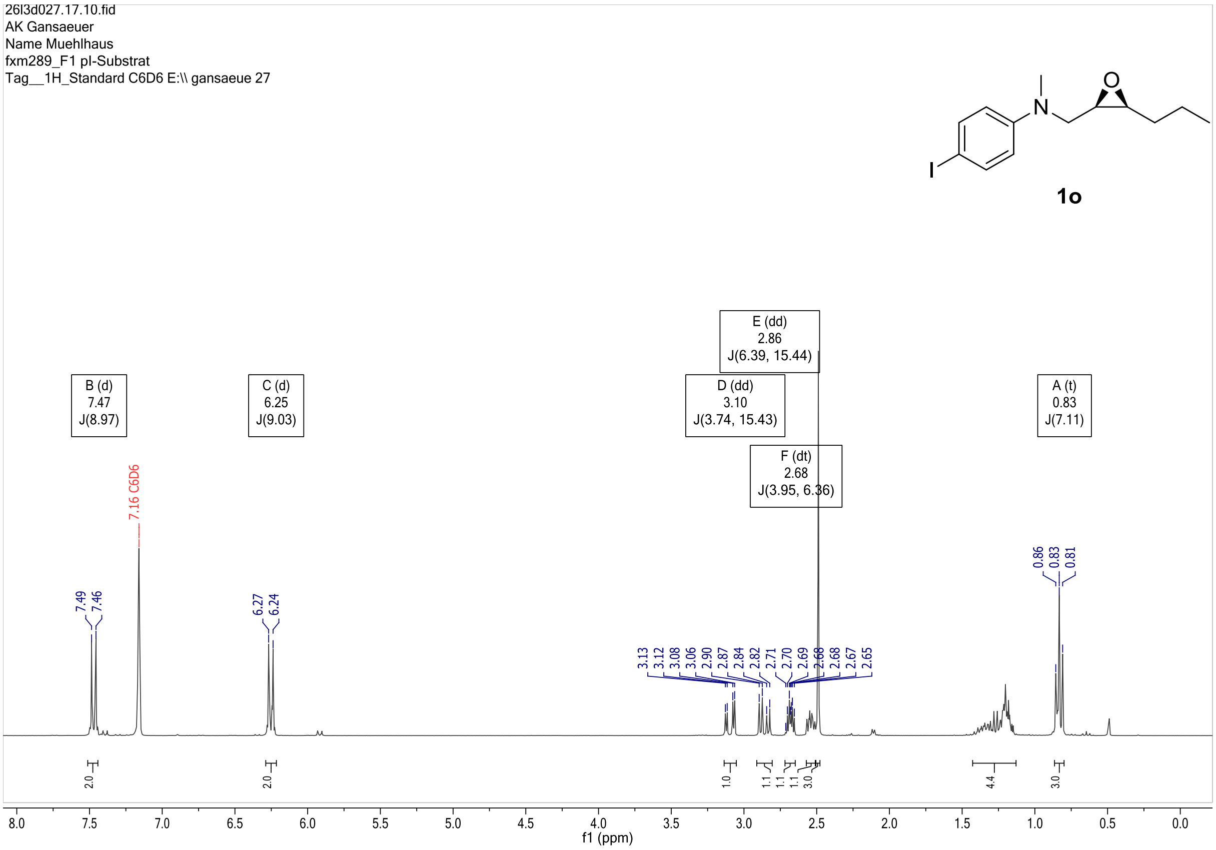
26l3d027.17.10.fid
AK Gansaeuer
Name Muehlhaus
fxm289_F1 pI-Substrat
Tag__1H_Standard C6D6 E:\\ gansaeue 27
1o
B (d)
7.47
J(8.97)
C (d)
6.25
J(9.03)
D (dd)
3.10
J(3.74, 15.43)
E (dd)
2.86
J(6.39, 15.44)
F (dt)
2.68
J(3.95, 6.36)
A (t)
0.83
J(7.11)
7.16 C6D6
7.49
7.46
6.27
6.24
3.13
3.12
3.08
3.06
2.90
2.87
2.84
2.82
2.71
2.70
2.69
2.68
2.68
2.67
2.65
0.86
0.83
0.81
2.0
2.0
1.0
1.1
1.1
1.1
3.0
4.4
3.0
f1 (ppm)

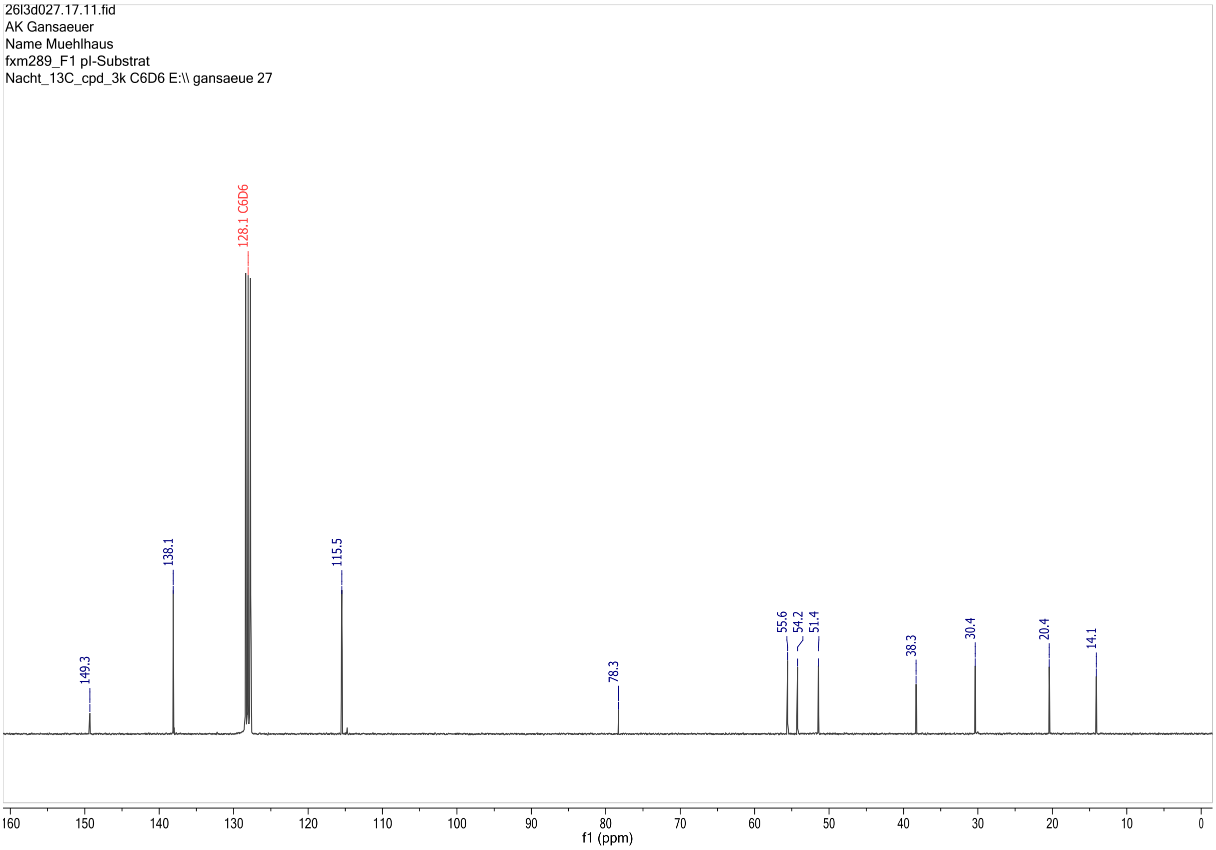
26l3d027.17.11.fid
AK Gansaeuer
Name Muehlhaus
fxm289_F1 pI-Substrat
Nacht_13C_cpd_3k C6D6 E:\\ gansaeue 27
128.1 C6D6
149.3
138.1
115.5
78.3
55.6
54.2
51.4
38.3
30.4
20.4
14.1
f1 (ppm)

3.5.2 Synthesis of *N*-cyclohexyl-4-iodo-*N*-(((*2R,3S*)-3-propyloxiran-2-yl)methyl)aniline (1p).

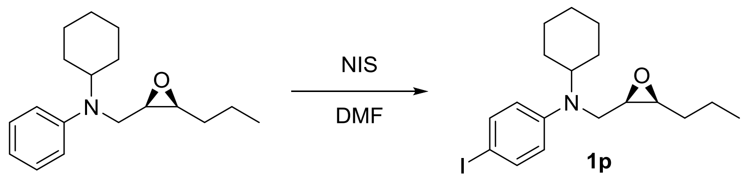

273 mg of **1d** (1.00 eq., 1.0 mmol) are dissolved in 1.5 mL DMF and cooled to 0 °C. Under vigorous stirring 225 mg *N*-iodosuccinimide NIS (1.00 eq., 1.0 mmol) is added in three portions after 0, 15 and 30 minutes. The mixture is stirred for 1 h at 0 °C and for 1 h at rt. The reaction is quenched by addition of 2 mL saturated $NaHSO_3$-solution and 5 mL cyclohexane are added. After phase separation the aqueous layer is extracted with cyclohexane (4 * 10 mL cyclohexane). The combined organic extracts are washed with H_2O (2 * 5 mL) and brine (1 * 5 mL) and dried over Na_2SO_4. After removal of solvents under reduced pressure, the crude product is purified by flash chromatography (Al_2O_3, Eluent: CH : MTBE = 98 : 2) to yield 389 mg **1p** (97%) as a light yellow oil.

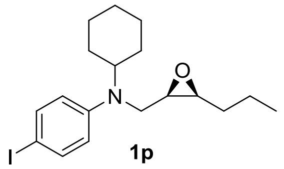

1p
$C_{18}H_{26}INO$
399,32 g/mol

R_f= 0.4 (10% MTBE in CH); **^{1}H NMR (400.1 MHz, C_6D_6, RT) δ [ppm]**= 0.83-0.97 (m, 4H), 1.01-1.51 (m, 9H), 1.54-1.64 (m, 3H), 1.73 (dtt, *J*= 12.3 Hz, *J*= 3.4 Hz, *J*= 1.7 Hz, 1H), 2.65 (ddd, *J*= 6.6 Hz, *J*= 5.2 Hz, *J*= 4.1 Hz, 1H), 2.73 (ddd, *J*= 5.2 Hz, *J*= 4.1 Hz, *J*= 2.8 Hz, 1H), 2.99 (dd, *J*= 16.2 Hz, *J*= 5.2 Hz, 1H), 3.23-3.34 (m, 2H), 6.50-6.56 (m, 2H), 7.48-7.55 (m, 2H); **^{13}C NMR (101 MHz, C_6D_6, RT) δ [ppm]**= 14.1, 20.5, 26.3, 26.2, 26.4, 30.6, 30.7, 31.3, 44.2, 56.4, 56.6, 57.5, 78.0, 116.1, 138.4, 148.8; **IR (KBr) ν_{max} (neat)[cm^{-1}]**= 767, 801, 1007, 1148, 1173, 1234, 1282, 1348, 1361, 1450, 1491, 1584, 2854, 2928; **HRMS (ESI) m/z** calculated for $[M+H]^+$ 400.1132 u found 400.1128 u; **α_D^{20}(CHCl$_3$)=** -18.6°.

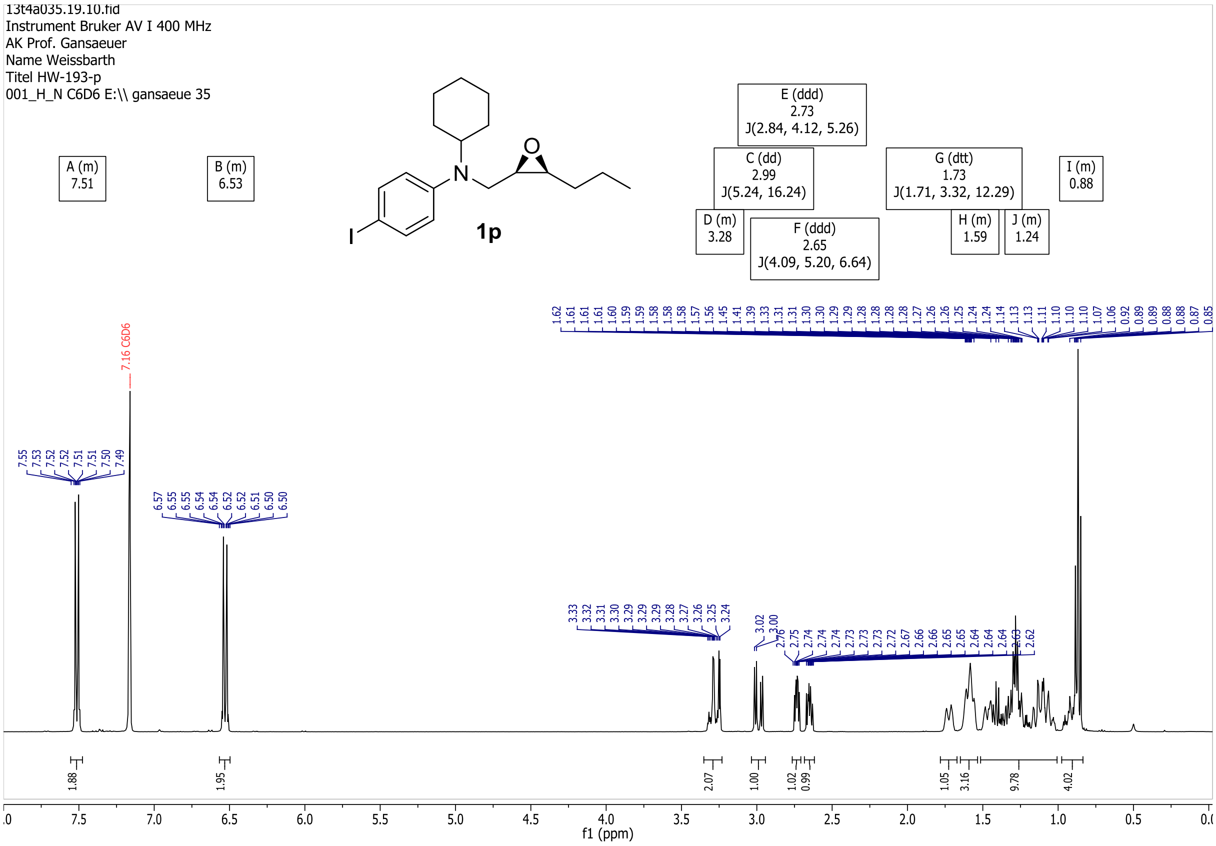
13t4a035.19.10.fid
Instrument Bruker AV I 400 MHz
AK Prof. Gansaeuer
Name Weissbarth
Titel HW-193-p
001_H_N C6D6 E:\\ gansaeue 35
1p
A (m) 7.51
B (m) 6.53
C (dd) 2.99 J(5.24, 16.24)
D (m) 3.28
E (ddd) 2.73 J(2.84, 4.12, 5.26)
F (ddd) 2.65 J(4.09, 5.20, 6.64)
G (dtt) 1.73 J(1.71, 3.32, 12.29)
H (m) 1.59
I (m) 0.88
J (m) 1.24
7.16 C6D6
1.88
1.95
2.07
1.00
1.02
0.99
1.05
3.16
9.78
4.02
f1 (ppm)

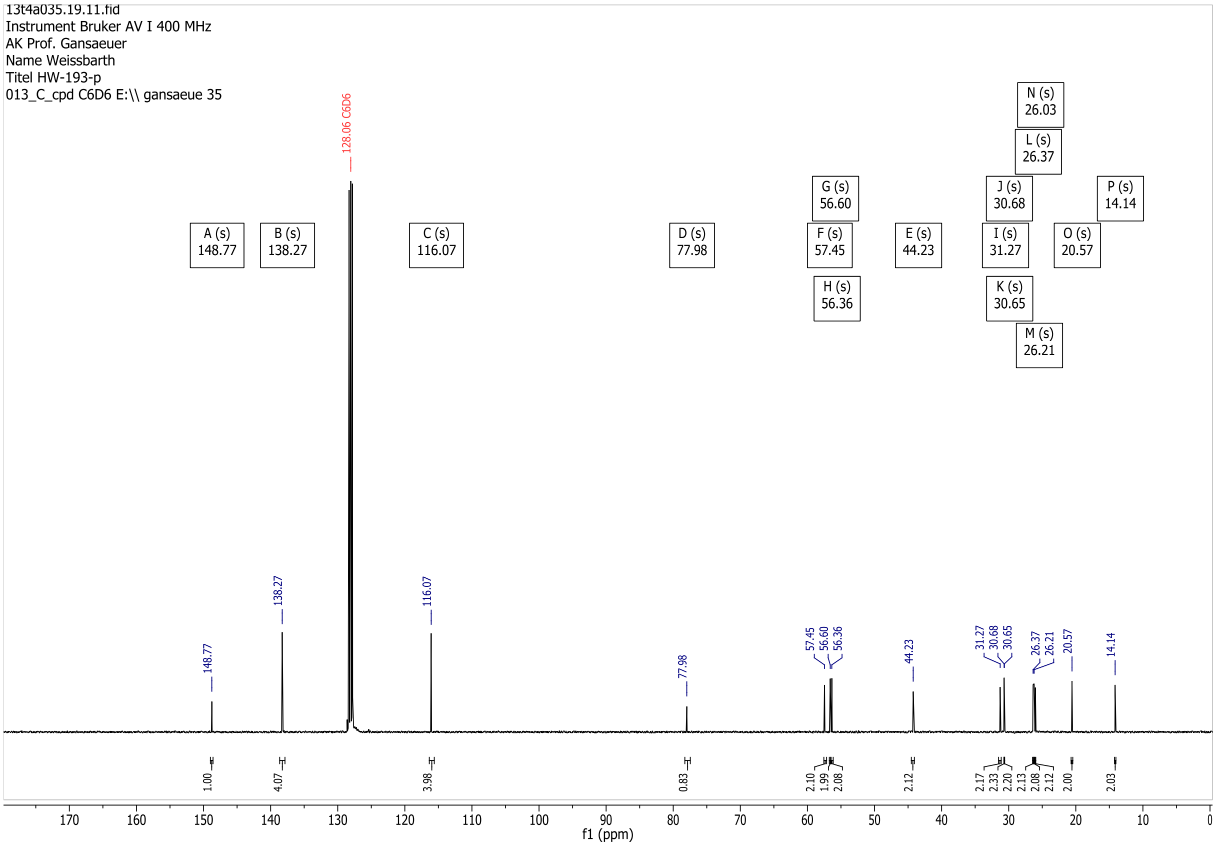
13t4a035.19.11.fid
Instrument Bruker AV I 400 MHz
AK Prof. Gansaeuer
Name Weissbarth
Titel HW-193-p
013_C_cpd C6D6 E:\\ gansaeue 35
128.06 C6D6
A (s) 148.77
B (s) 138.27
C (s) 116.07
D (s) 77.98
E (s) 44.23
F (s) 57.45
G (s) 56.60
H (s) 56.36
I (s) 31.27
J (s) 30.68
K (s) 30.65
L (s) 26.37
M (s) 26.21
N (s) 26.03
O (s) 20.57
P (s) 14.14
f1 (ppm)

4. REO-ArS$_R$

4.1 Investigation of different catalysts for the REO-ArS$_R$ with epoxide 1a.

Table 1. Investigation on the performance of different catalysts in the REO-ArS$_R$ of **1a**. Reaction conditions: 10 mol% catalyst, 30 mol% Zn, entries 1,3,4,: 30 mol% Lut·HCl, 0.2 M in THF, 48 h, rt. Regioisomeric ratios (*r.r.*) and diastereoisomeric ratios (*d.r.*) were determined by integration of the signals of the C-OH-group in the ^{13}C-NMR of the crude-mixture. [a]: *d.r.* (**2a**) = ***cis*-2a** : ***trans*-2a**; [b]: *d.r.* (**3a**) = **(*R,S*)-3a** : **(*S,S*)-3a**; signals of the C-OH-group: ***cis*-2a** 67.3 ppm, ***trans*-2a** 67.9 ppm, **(*R,S*)-3a** 72.1 ppm, **(*S,S*)-3a** 73.9 ppm. n.d. = not determined.

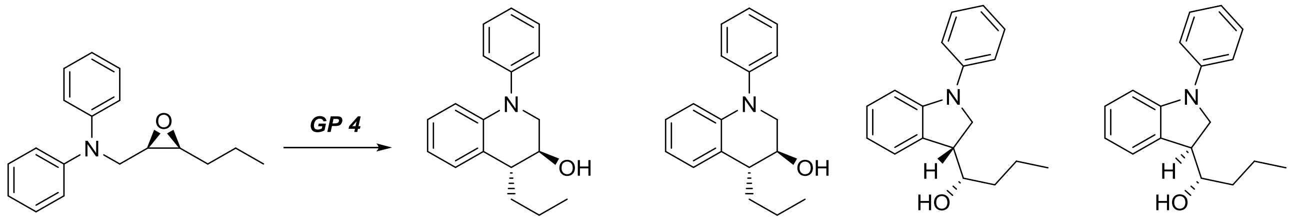

***cis*-2a** ***trans*-2a** **(*R*,*S*)-3a** **(*S*,*S*)-3a**

	catalyst	conversion of **1a** in %	***r.r.*** **2a** : **3a**	*d.r.*$_{2a}$[a]	*yield* **2a** (*d.r.*$_{2a\ isolated}$)	*d.r.*$_{3a}$[b]	*yield* **3a** (*d.r.*$_{3a\ isolated}$)
**1**	**Cp_2Ti-Cl_2**	> 98	78 : 22	31 : 69	-	90 : 10	-
**2**	**Cp_2Ti-$(OTs)_2$**	93	79 : 21	25 : 75	-	90 : 10	-
**3**	***ent*-Kat-Cl_2 (5 mol%)**	> 98	10 : 90	n.d.	-	92 : 8	72% (>98 : <2)
**4**	**Kat-Cl_2**	> 98	93 : 7	33 : 67	72% (15 : 85)	n.d.	-
**5**	**Kat-$(OTs)_2$**	> 98	94 : 6	24 : 76	73% (10 : 90)	n.d.	-

4.2 Structural assignment of the tetrahydroquinoline-scaffold and configurational analysis with tetrahydroquinolines 2a and 2g.

The constitution of the THQ scaffold was elucidated by single crystal X-ray diffraction of purified N-methyl-tetrahydroquinoline **2g** (pure *trans*-isomer: ***trans*-2g**). The X-ray also revealed the *trans*-configuration and the axial orientation of the hydroxy group in the pseuso-chairlike 1,2,3,4-tetrahydro-pyridine ring of the THQ **2g**.

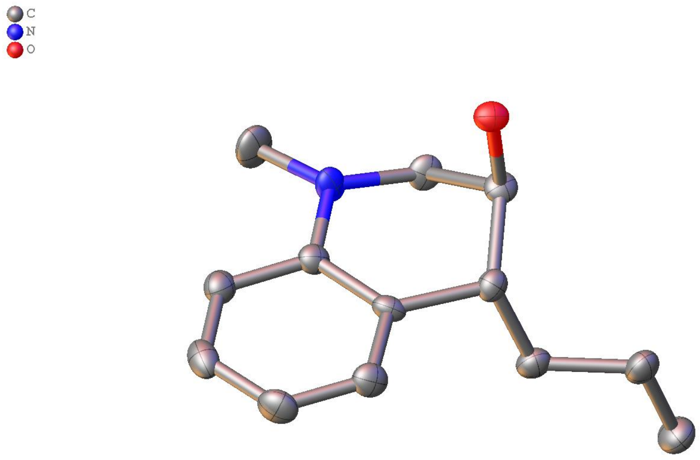

In the ^{1}H-NMR-spectra of ***cis*-2g** and ***trans*-2g**, the signal of the equatorially oriented proton H_a features three types of couplings: the geminal ^2J-coupling to $H_{a'}$, the ^3J-coupling to H_b and the ^4J- or so called 'W-coupling' to H_c The signal of $H_{a'}$ does not feature the 'W-coupling' and is therefore easily identified. In ***cis*-2g** as well as in ***trans*-2g** $H_{a'}$ shows a ^3J-coupling with H_b. In the case of ***cis*-2g**, $H_{a'}$ and H_b are *trans* to each other leading to a large $^3J^{trans}$ of 6.0 Hz. In the case of ***trans*-2g**, $H_{a'}$ and H_b are *cis* to each other leading to a small $^3J^{cis}$ of 2.5 Hz.

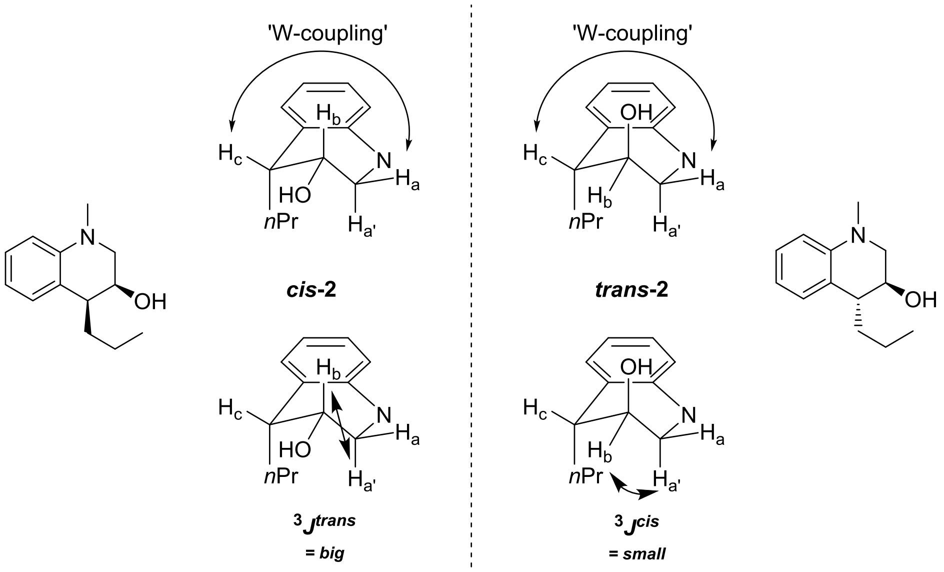

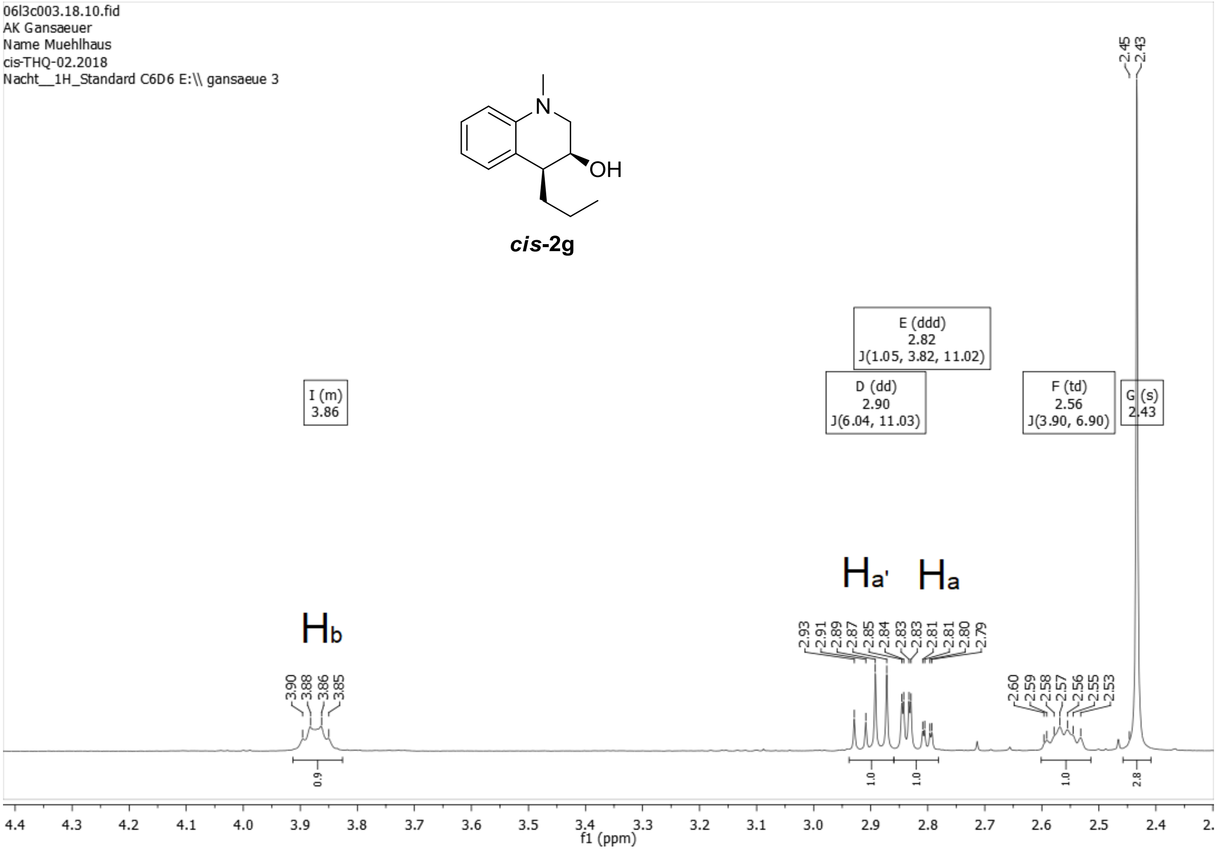
06l3c003.18.10.fid
AK Gansaeuer
Name Muehlhaus
cis-THQ-02.2018
Nacht__1H_Standard C6D6 E:\\ gansaeue 3
N
OH
cis-2g
E (ddd)
2.82
J(1.05, 3.82, 11.02)
I (m)
3.86
D (dd)
2.90
J(6.04, 11.03)
F (td)
2.56
J(3.90, 6.90)
G (s)
2.43
Ha' Ha
Hb
f1 (ppm)

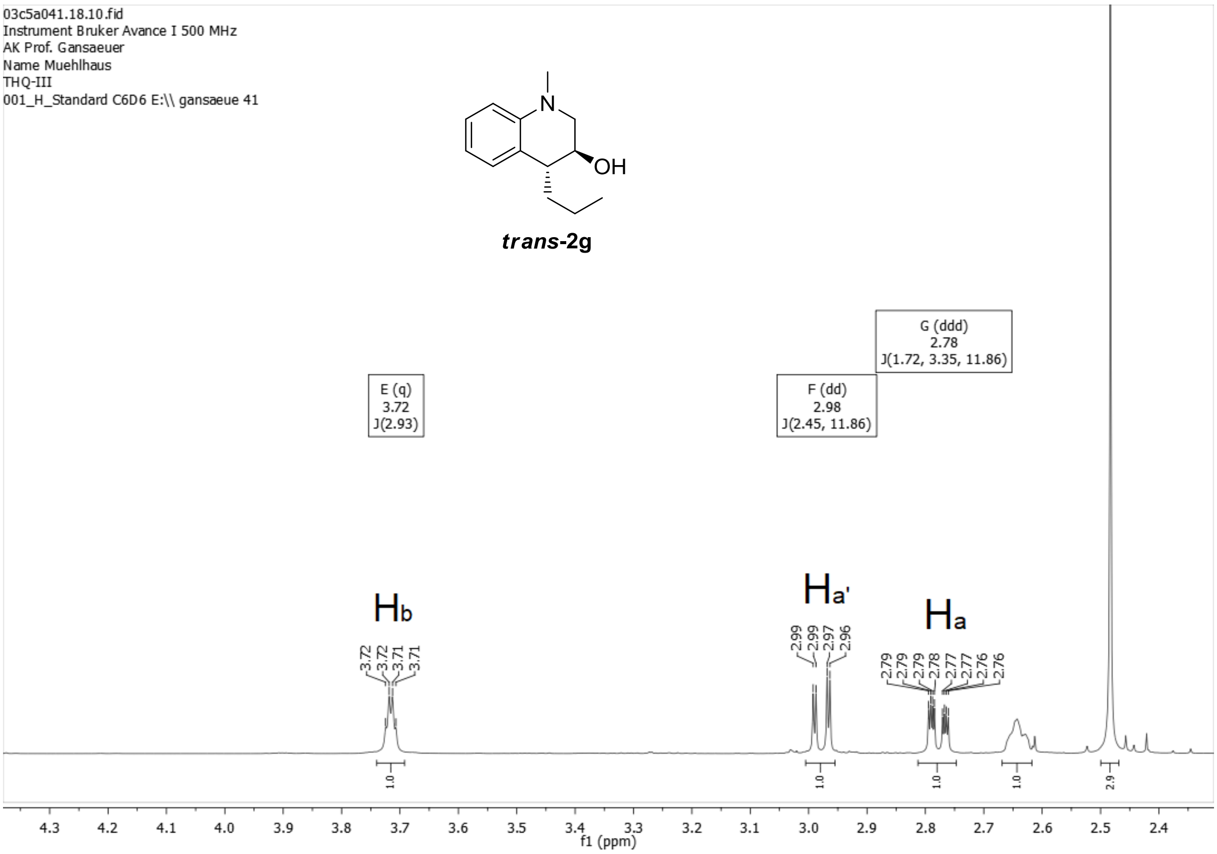
03c5a041.18.10.fid
Instrument Bruker Avance I 500 MHz
AK Prof. Gansaeuer
Name Muehlhaus
THQ-III
001_H_Standard C6D6 E:\\ gansaeue 41
N
OH
trans-2g
G (ddd)
2.78
J(1.72, 3.35, 11.86)
E (q)
3.72
J(2.93)
F (dd)
2.98
J(2.45, 11.86)
Hb
Ha'
Ha
f1 (ppm)

In addition to THQ **2g**, the coupling behavior of *N*-phenyl-substituted tetrahydroquinoline **2a** is very similar. For the mainly occurring isomer ***trans*-2a**, the ^{3}J coupling constant between $H_{a'}$ and H_b is small, while it is big for the side product ***cis*-2a**.

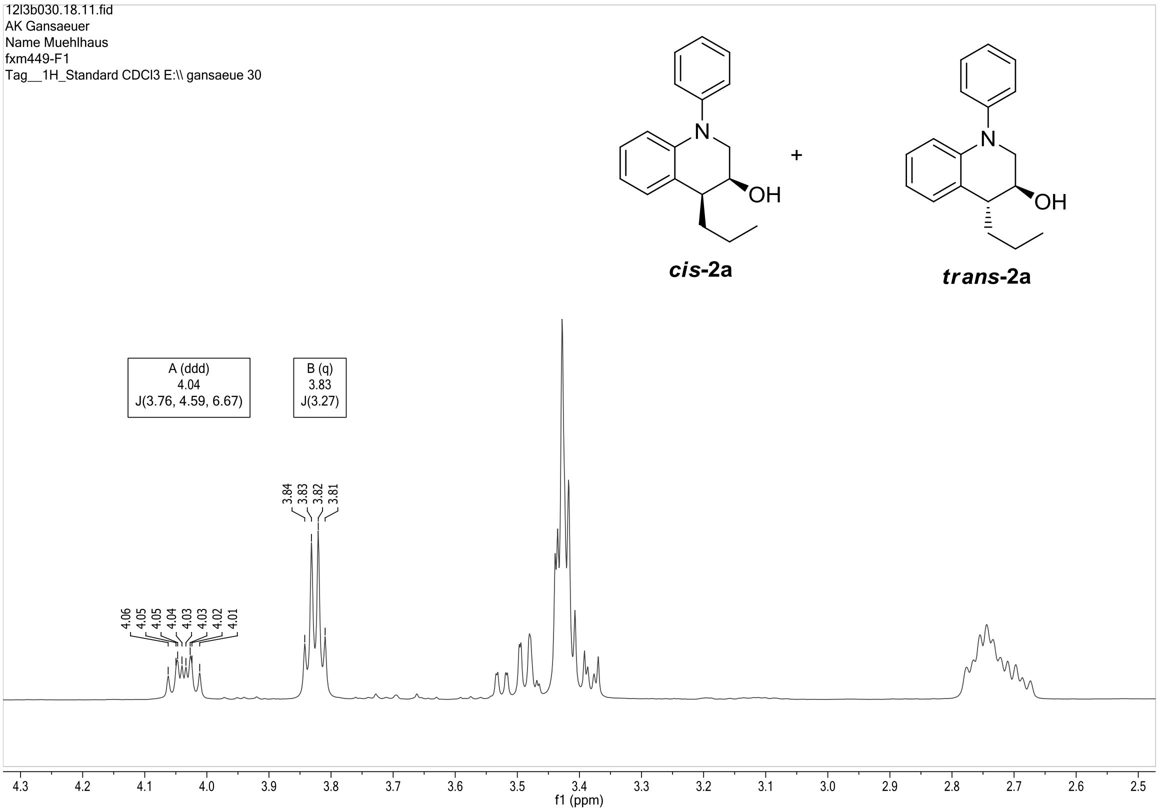

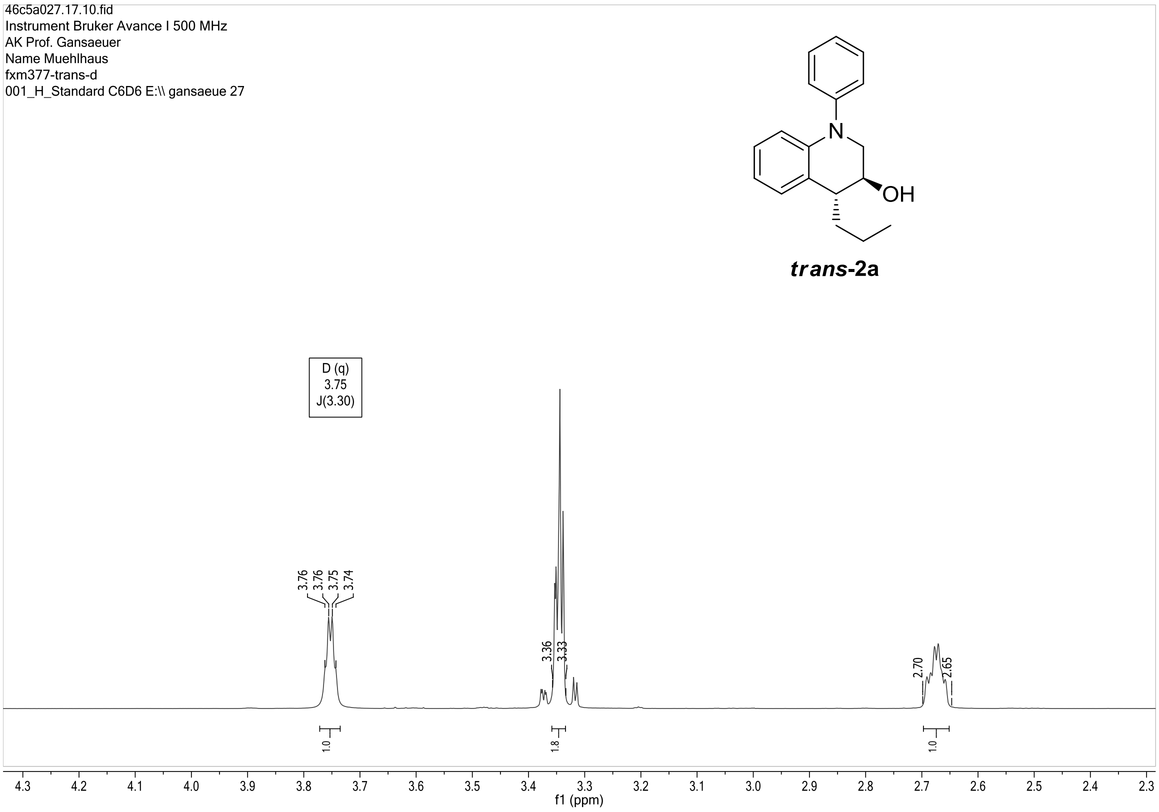

4.3 Synthesis of tetrahydroquinolines by REO-ArS$_R$

4.3.1 Synthesis of (3*S*,4*R*)-1-Phenyl-4-propyl)-1,2,3,4-tetrahydroquinoline-3-ol (2a).

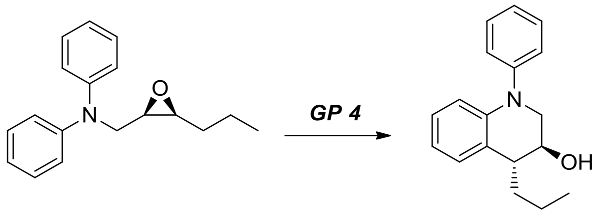

According to GP4: 134 mg of **1a** (1.00 eq., 0.50 mmol), 10 mg zinc (0.30 eq., 0.15 mmol) and 40 mg **cat-(OTs)$_2$** (0.10 eq., 0.05 mmol) are reacted in dry THF for 48 hours at room temperature. Regio- and diastereoisomeric ratio were determined by ^{13}C-NMR of the crude product. Flash chromatography (SiO_2, Eluent: Ch : Mtbe = 87 : 13) yielded 98 mg **2a** (d.r. = 90 : 10, 73%) as a viscous, colorless liquid.

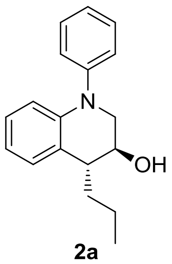

2a
$C_{18}H_{21}NO$
267.37 g/mol

R$_f$ = 0.63 (40% EE in Ch), **^{1}H-NMR (500 MHz, C_6D_6, RT): δ [ppm] =** 0.82 (t, 3H, *J* = 7.1 Hz) ,1.20-1.45 (m, 4H), 1.65-1.75 (s$_{br}$, 1H), 2.65-2.70 (m, 1H), 3.33-3.36 (m, 2H), 3.75 (dd, 1H, *J* = 3.3 Hz, *J* = 3.1 Hz), 6.76 (td, 1H, *J* = 7.3 Hz, *J* = 1.3 Hz), 6.85 (dd, 1H, *J* = 8.3 Hz, *J* = 1.3 Hz), 6.88-6.91 (m, 1H), 6.92-6.96 (m, 1H), 7.01 (dd, 1H, *J* = 7.5 Hz, *J* = 1.6 Hz), 7.07-711 (m, 2H), 7.11-7.15 (m, 2H). **^{13}C-NMR (75 MHz, C_6D_6, RT): δ [ppm] =** 14.4, 20.5, 38.8, 45.3, 53.5, 67.6, 116.0, 119.1, 124.3, 125.3, 125.6, 127.3, 129.8, 131.3, 143.3, 148.3. **IR (Film) $\tilde{\nu}$ [cm^{-1}] =** 461, 499, 559, 610, 698, 745, 957, 1061, 1094, 1127, 1204, 1239, 1299, 1377, 1492, 1574, 1592, 2870, 2927, 2956. **HRMS (ESI):** *m/z* calculated for $C_{18}H_{22}NO^+$: 268.1696 u, found: 268.1699 u. **$[\alpha]_D^{20}$**= -43.2 (*c* 0.25, $CHCl_3$). determination of d.r. (**2a**, isolated) by HPLC: *Knauer Eurospher II 100-2 C18*, H_2O/MeCN (65 : 35), flowrate 0.6 mL/min; t_R = 2.8 min (major, *trans*), t_R = 1.5 min (minor, *cis*), d.r. = 90 : 10. determination of *e.r.* (**2a**, isolated) by HPLC: *DAICEL Chiralpak IC-U*01; *n*Hexane/*i*PrOH (95 : 5); flowrate 0.43 mL/min; t_R = 1.5 min (major, 3*S*,4*R*), t_R = 2.0 min (minor, 3*R*,4*S*), *er* = > 99 : < 1.

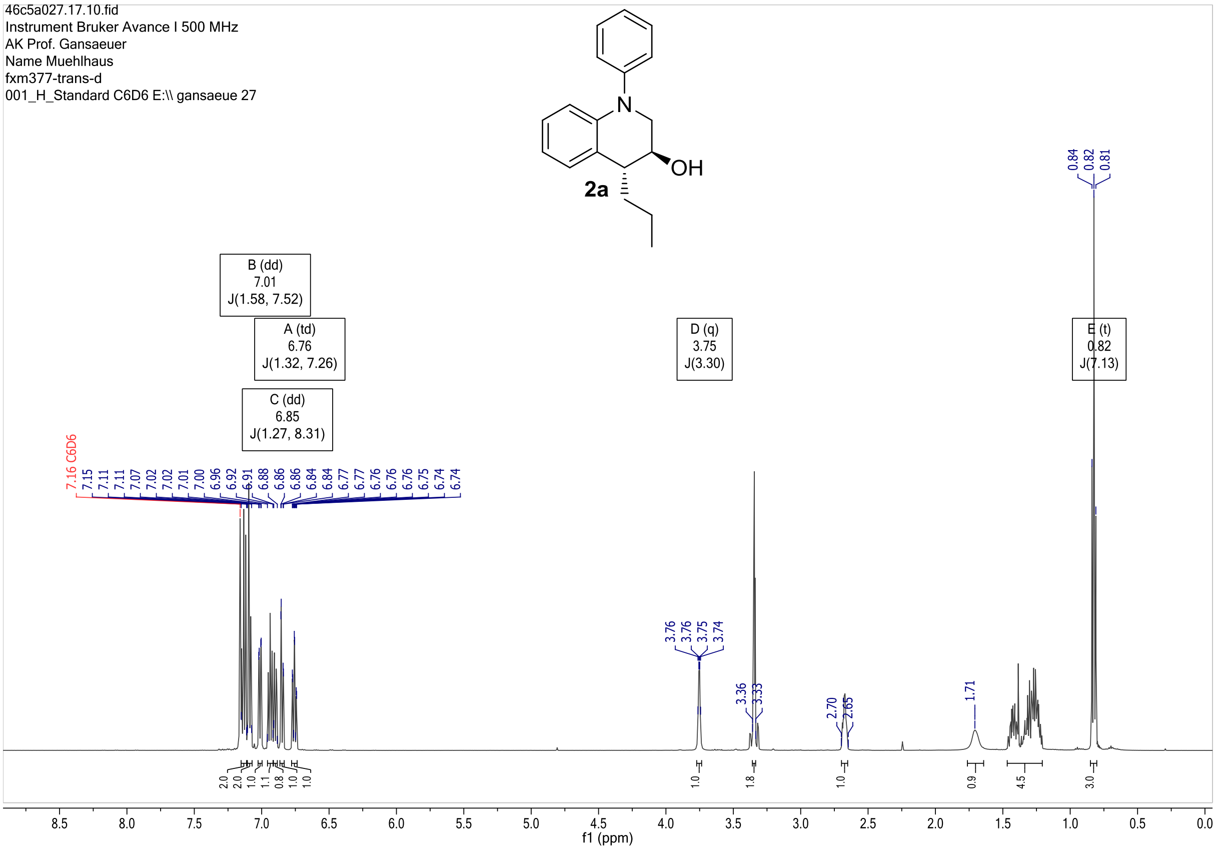
46c5a027.17.10.fid
Instrument Bruker Avance I 500 MHz
AK Prof. Gansaeuer
Name Muehlhaus
fxm377-trans-d
001_H_Standard C6D6 E:\\ gansaeue 27
2a
OH
N
B (dd) 7.01 J(1.58, 7.52)
A (td) 6.76 J(1.32, 7.26)
C (dd) 6.85 J(1.27, 8.31)
D (q) 3.75 J(3.30)
E (t) 0.82 J(7.13)
f1 (ppm)

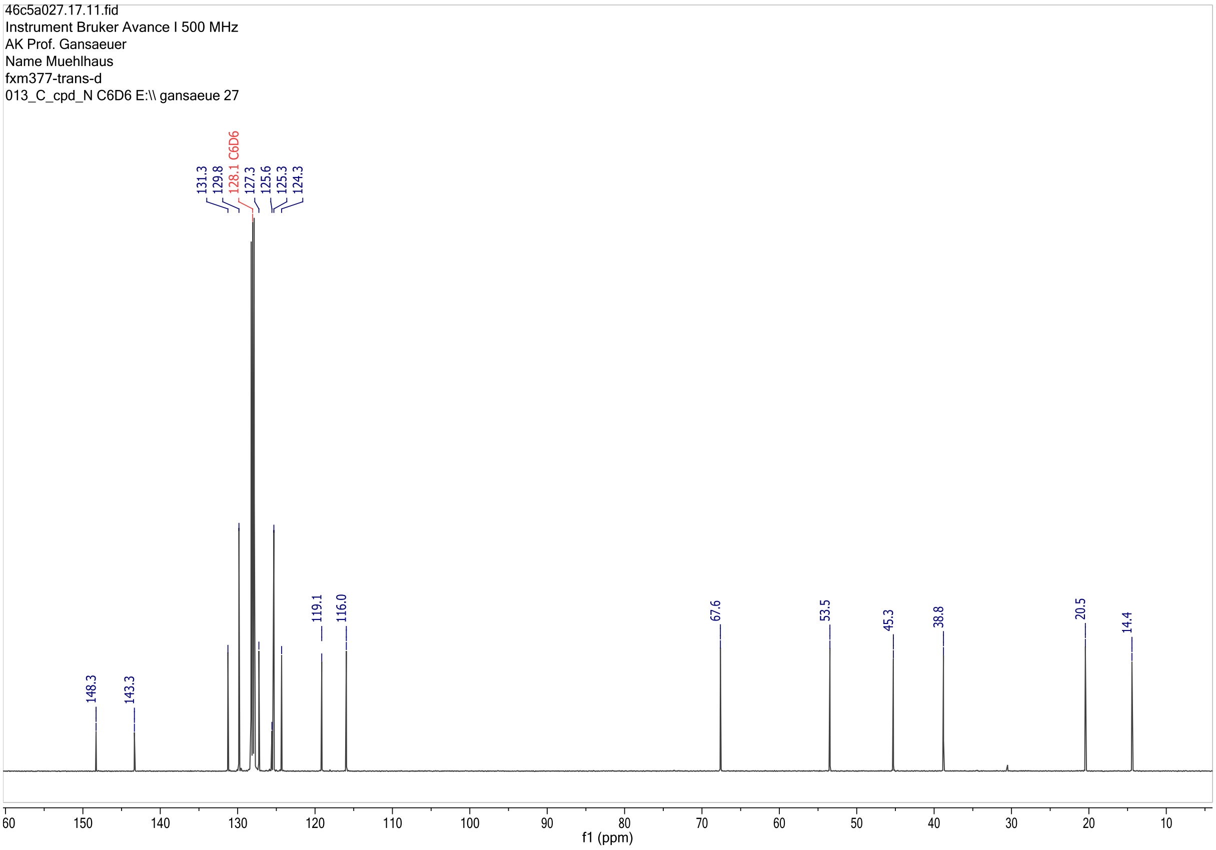
46c5a027.17.11.fid
Instrument Bruker Avance I 500 MHz
AK Prof. Gansaeuer
Name Muehlhaus
fxm377-trans-d
013_C_cpd_N C6D6 E:\\ gansaeue 27
131.3
129.8
128.1 C6D6
127.3
125.6
125.3
124.3
148.3
143.3
119.1
116.0
67.6
53.5
45.3
38.8
20.5
14.4
f1 (ppm)

4.3.2 Synthesis of (3*S*,4*R*)-4-isobutyl-1-phenyl-4-propyl)-1,2,3,4-tetrahydroquinoline-3-ol (2c).

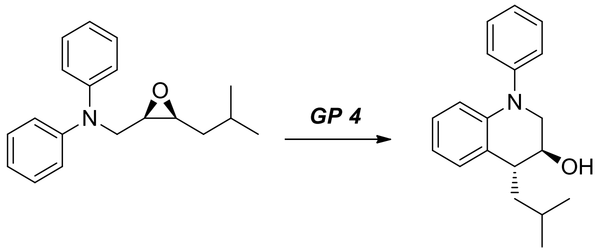

According to GP4: 141 mg of **1c** (1.00 eq., 0.50 mmol), 10 mg zinc (0.30 eq., 0.15 mmol) and 40 mg **cat-(OTs)$_2$** (0.10 eq., 0.05 mmol) are reacted in dry THF for 48 hours at room temperature. Regio- and diastereoisomeric ratio were determined by ^{13}C-NMR of the crude product. Flash chromatography (SiO_2, Eluent: Ch : Mtbe = 87 : 13) yielded 104 mg **2c** (d.r. = 81 : 19, 74%) as a viscous, colorless liquid.

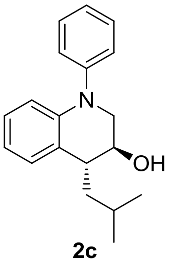

2c
$C_{19}H_{22}NO$
281.39 g/mol

R$_f$ = 0.25 (30% Et_2O in Ch), **^{1}H-NMR (500 MHz, C_6D_6, RT): δ [ppm] =** 0.86 (d, 6H, *J* = 6.5 Hz) ,1.10-1.20 (m, 1H), 1.63 (sept., 1H, *J* = 6.5 Hz), 2.83 (td, 1H, *J* = 7.4 Hz, *J* = 2.8 Hz), 3.30-3.37 (m, 2H), 3.76 (d, 1H, *J* = 4.0 Hz), 6.76 (td, 1H, *J* = 7.3 Hz, *J* = 1.3 Hz), 6.74-6.79 (m, 1H), 6.96-6.84 (m, 3H), 7.02-7.14 (m, 4H). **^{13}C-NMR (75 MHz, C_6D_6, RT): δ [ppm] =** 22.7, 23.1, 25.2, 43.0, 46.7, 53.1, 67.7, 115.9, 119.2, 123.5, 124.4, 125.4, 127.3, 129.9, 131.3, 143.3, 148.3. **IR (Film) ṽ [cm^{-1}] =** 698, 745, 1037, 1063, 1205, 1242, 1274, 1302, 1462, 1493, 1574, 1592, 2953. **HRMS (ESI):** *m/z* calculated for $C_{19}H_{24}NO^+$: 282.1852 u, found: 282.1849 u. **[α]$_D^{20}$=** - 47.0 (*c* 1.00, $CHCl_3$).

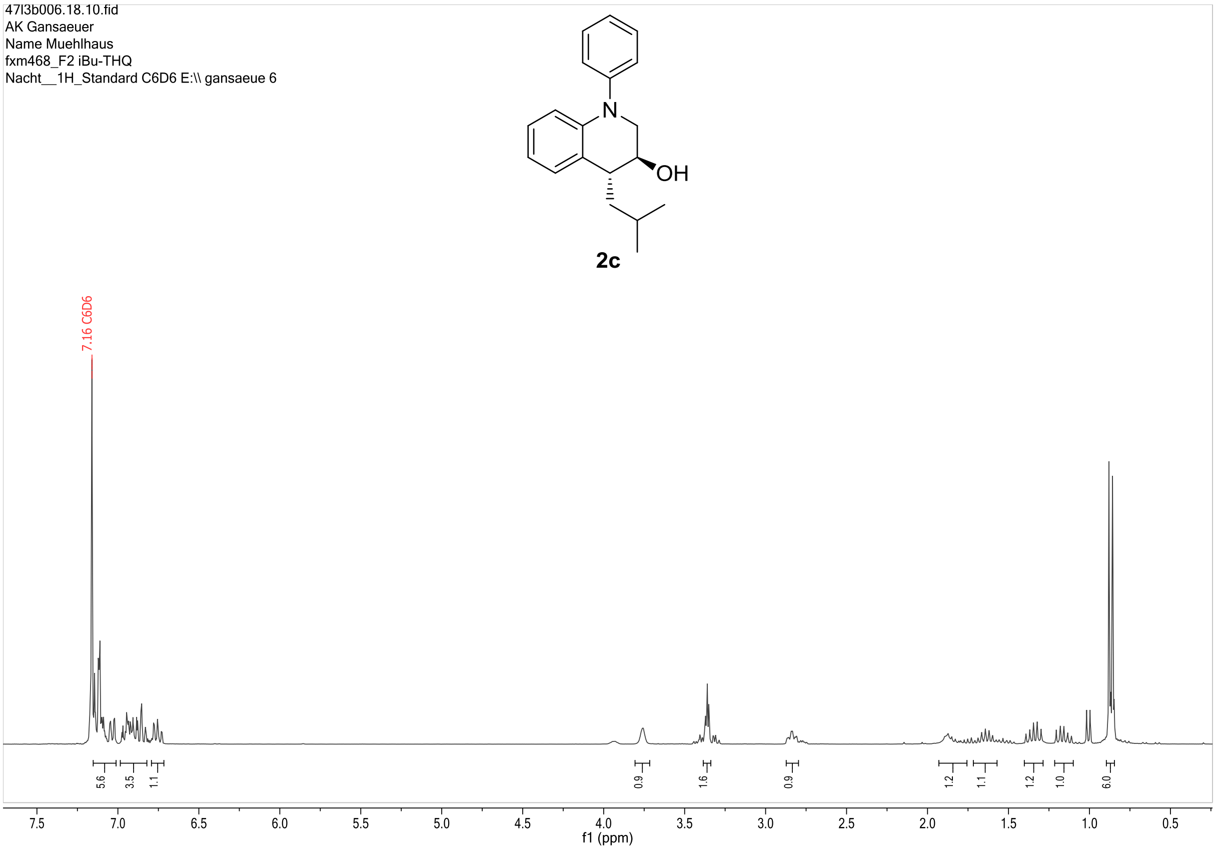
47l3b006.18.10.fid
AK Gansaeuer
Name Muehlhaus
fxm468_F2 iBu-THQ
Nacht__1H_Standard C6D6 E:\\ gansaeue 6
N
OH
2c
7.16 C6D6
5.6
3.5
1.1
0.9
1.6
0.9
1.2
1.1
1.2
1.0
6.0
7.5 7.0 6.5 6.0 5.5 5.0 4.5 4.0 3.5 3.0 2.5 2.0 1.5 1.0 0.5
f1 (ppm)

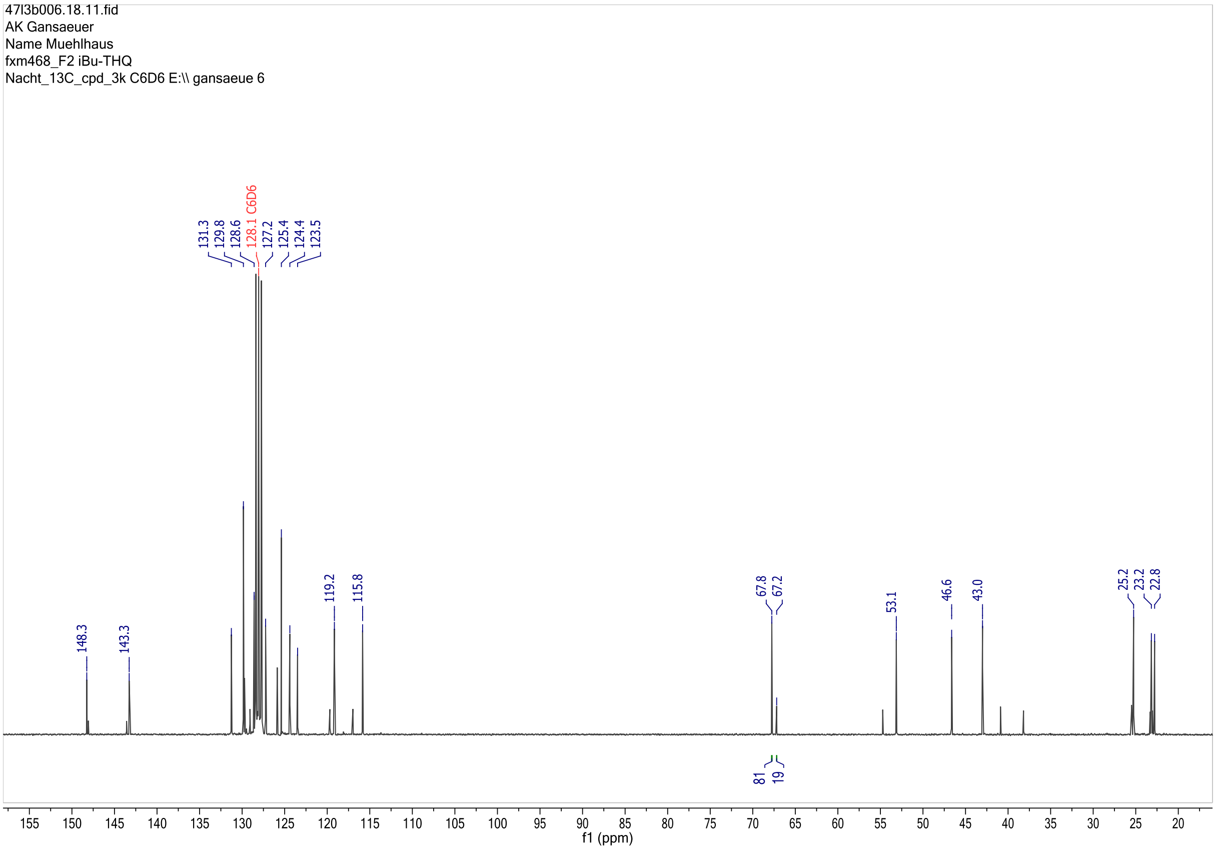
47l3b006.18.11.fid
AK Gansaeuer
Name Muehlhaus
fxm468_F2 iBu-THQ
Nacht_13C_cpd_3k C6D6 E:\\ gansaeue 6
131.3
129.8
128.6
128.1 C6D6
127.2
125.4
124.4
123.5
148.3
143.3
119.2
115.8
67.8
67.2
53.1
46.6
43.0
25.2
23.2
22.8
81
19
155 150 145 140 135 130 125 120 115 110 105 100 95 90 85 80 75 70 65 60 55 50 45 40 35 30 25 20
f1 (ppm)

4.3.3 Synthesis of (3*S*,4*R*)-1-cyclohexyl-4-propyl)-1,2,3,4-tetrahydroquinoline-3-ol (2d).

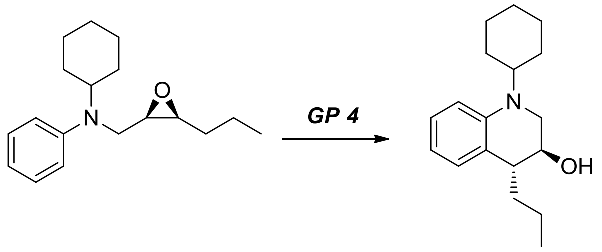

According to GP4: 137 mg of **1d** (1.00 eq., 0.50 mmol), 10 mg zinc (0.30 eq., 0.15 mmol) and 40 mg **cat-(OTs)$_2$** (0.10 eq., 0.05 mmol) are reacted in dry THF for 48 hours at room temperature. Regio- and diastereoisomeric ratio were determined by ^{13}C-NMR of the crude product. Flash chromatography (SiO_2, Eluent: Ch : Mtbe = 87 : 13) yielded 114 mg **2d** (d.r. = 97 : 3, 83%) as a viscous, colorless liquid.

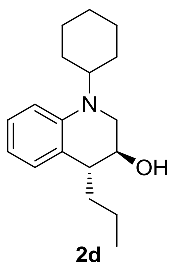

2d
$C_{18}H_{27}NO$
273.41 g/mol

R$_f$ = 0.7 (40% EE in CH); **^{1}H-NMR (300 MHz, C_6D_6, RT): δ [ppm]** = 0.83 (t, 3H, *J* = 7.0 Hz), 1.10-1.44 (m, 10H), 1.51-1.74 (m, 4H), 1.99-2.17 (s_{br}, 1H), 2.62-2.69 (m, 1H), 2.89 (dd, 1H, *J* = 12.1 Hz, *J* = 2.5 Hz), 3.00 (ddd, 1H, *J* = 12.2 Hz, *J* = 3.4 Hz, *J* = 1.6 Hz), 3.46 (tt, 1H, *J* = 8.1 Hz, *J* = 3.3 Hz), 3.75-3.82 (m, 1H), 6.67 (d, 1H, *J* = 8.4 Hz), 6.72 (td, 1H, *J* = 7.3 Hz, *J* = 1.0 Hz); 7.01 (dd, 2H, *J* = 7.4 Hz, *J* = 1.7 Hz), 7.10-7.14 (m, 1H). **^{13}C-NMR (75 MHz, C_6D_6, RT): δ [ppm]** = 14.5, 20.5, 26.3, 26.3, 26.5, 27.3, 29.6, 29.7, 39.2, 44.3, 45.5, 55.7, 66.5, 111.0, 116.4, 123.9, 127.8, 131.7, 144.0. **IR ṽ [cm^{-1}]** = 497, 741, 1049, 1174, 1242, 1303, 1455, 1601, 2856, 2929. **HRMS (ESI):** *m/z* calculated for $C_{18}H_{28}NO^+$: 274.2165 u, found: 274.2167 u. **[α]$_D^{20}$**= -36.0 ° (*c* 0.5, $CHCl_3$). determination of d.r. (**2c**, isolated) by HPLC: *DAICEL Chiralpak IC-U*; *n*Hexane/*i*PrOH (95 : 5); flowrate 1.0 mL/min; t_R = 1.0 min (minor, *3S4S*), t_R = 1.5 min (minor, *3S4R*); d.r. = 97 : 3.

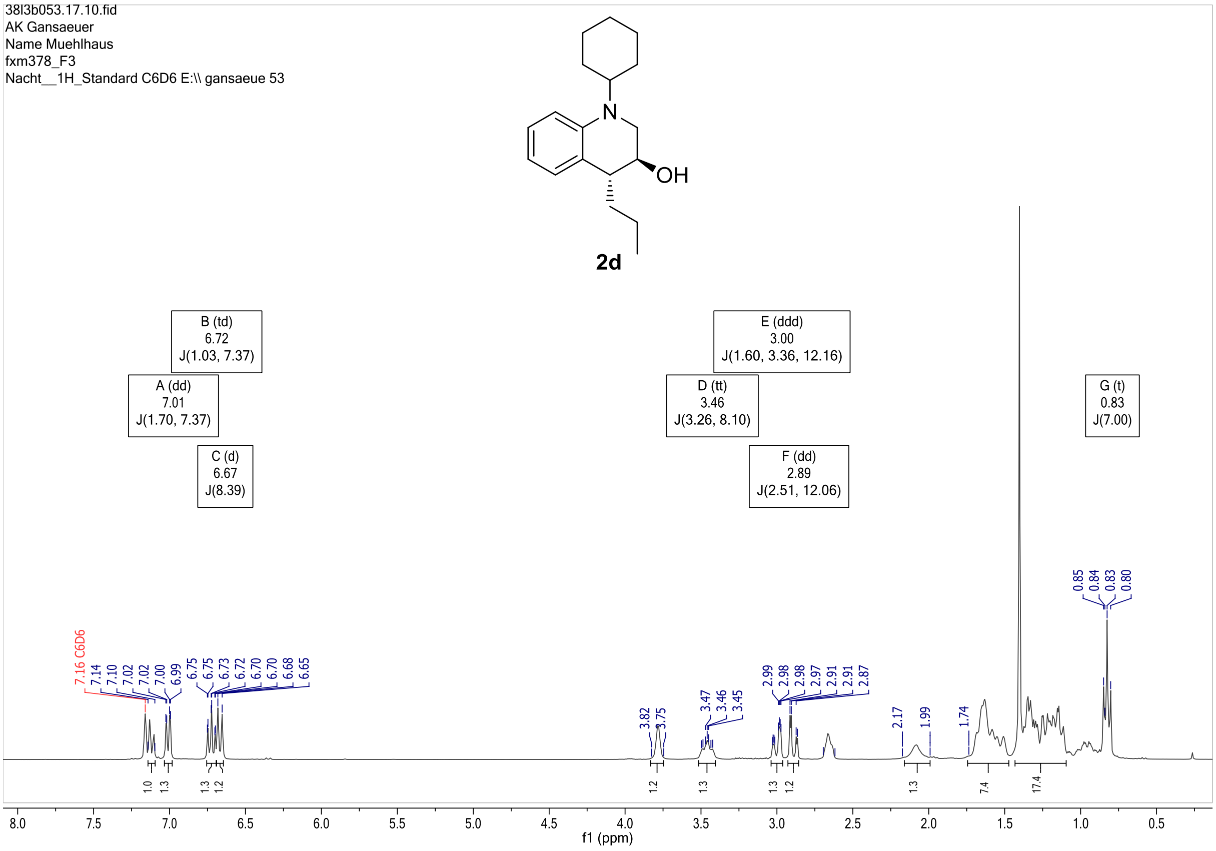
38l3b053.17.10.fid
AK Gansaeuer
Name Muehlhaus
fxm378_F3
Nacht__1H_Standard C6D6 E:\\ gansaeue 53
2d
A (dd) 7.01 J(1.70, 7.37)
B (td) 6.72 J(1.03, 7.37)
C (d) 6.67 J(8.39)
D (tt) 3.46 J(3.26, 8.10)
E (ddd) 3.00 J(1.60, 3.36, 12.16)
F (dd) 2.89 J(2.51, 12.06)
G (t) 0.83 J(7.00)
f1 (ppm)

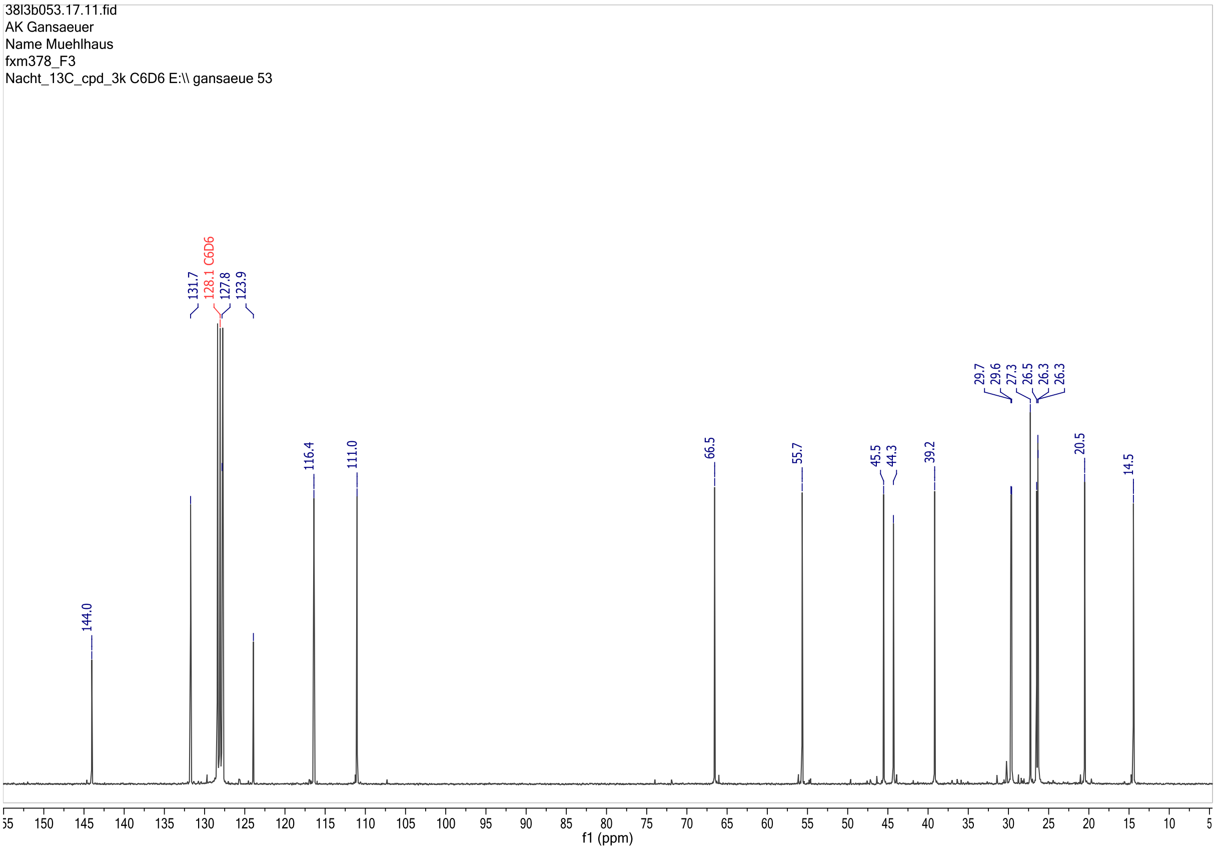
38l3b053.17.11.fid
AK Gansaeuer
Name Muehlhaus
fxm378_F3
Nacht_13C_cpd_3k C6D6 E:\\ gansaeue 53
144.0
131.7
128.1 C6D6
127.8
123.9
116.4
111.0
66.5
55.7
45.5
44.3
39.2
29.7
29.6
27.3
26.5
26.3
26.3
20.5
14.5
f1 (ppm)

4.3.4 Synthesis of (3*S*,4*R*)-1-*iso*propyl-4-propyl)-1,2,3,4-tetrahydroquinoline-3-ol (2e).

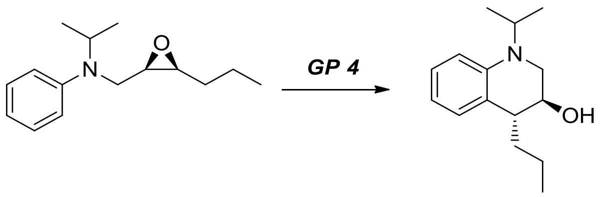

According to GP4: 117 mg of **1e** (1.00 eq., 0.50 mmol), 10 mg zinc (0.30 eq., 0.15 mmol) and 40 mg **cat-(OTs)$_2$** (0.10 eq., 0.05 mmol) are reacted in dry THF for 48 hours at room temperature. Regio- and diastereoisomeric ratio were determined by ^{13}C-NMR of the crude product. Flash chromatography (SiO_2, Eluent: Ch : Mtbe = 87 : 13) yielded 82 mg **2e** (d.r. = 95 : 5, 70%) as a viscous, colorless liquid.

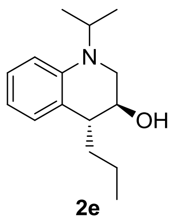

2e
$C_{15}H_{23}NO$
233.35 g/mol

R$_f$ = 0.6 (40% EE in CH), **^{1}H-NMR (300 MHz, C_6D_6, RT): δ [ppm] =** 0.80 (t, 3H, *J* = 7.0 Hz), 0.87 (d, 3H, *J* = 6.6 Hz), 0.91 (d, 3H, *J* = 6.6 Hz), 1.12-1.40 (m, 4H), 1.51-1.74 (m, 4H), 1.66-1.74 (s_{br}, 1H), 2.60-2.68 (m, 1H), 2.78 (dd, 1H, *J* = 12.0 Hz, *J* = 2.4 Hz), 2.91 (ddd, 1H, *J* = 12.1 Hz, *J* = 3.4 Hz, *J* = 1.7 Hz), 3.73-3.88 (m, 2H), 6.60 (dt, 1H, *J* = 8.4 Hz, *J* = 0.9 Hz), 6.75 (td, 1H, *J* = 7.3 Hz, *J* = 1.1 Hz), 7.01 (dd, 2H, *J* = 7.4 Hz, *J* = 1.8 Hz), 7.10-7.18 (m, 1H). **^{13}C-NMR (75 MHz, C_6D_6, RT): δ [ppm] =** 14.4, 18.4, 19.1, 20.5, 39.3, 42.7, 45.6, 46.2, 66.4, 111.2, 116.6, 124.0, 127.9, 131.8, 144.1. **IR (Film) ṽ [cm^{-1}] =** 453, 741, 1051, 1088, 1119, 1168, 1190, 1302, 1363, 1456, 1495, 1601, 2871, 2928, 2957. **HRMS (ESI):** *m/z* calculated for $C_{15}H_{26}NO^+$: 236.2009 u, found: 234.1855 u. **[α]$_D$^{20}**= -50.0 °(*c* 0.1, $CHCl_3$). d.r. = 95 : 5 (**2d**, isolated).

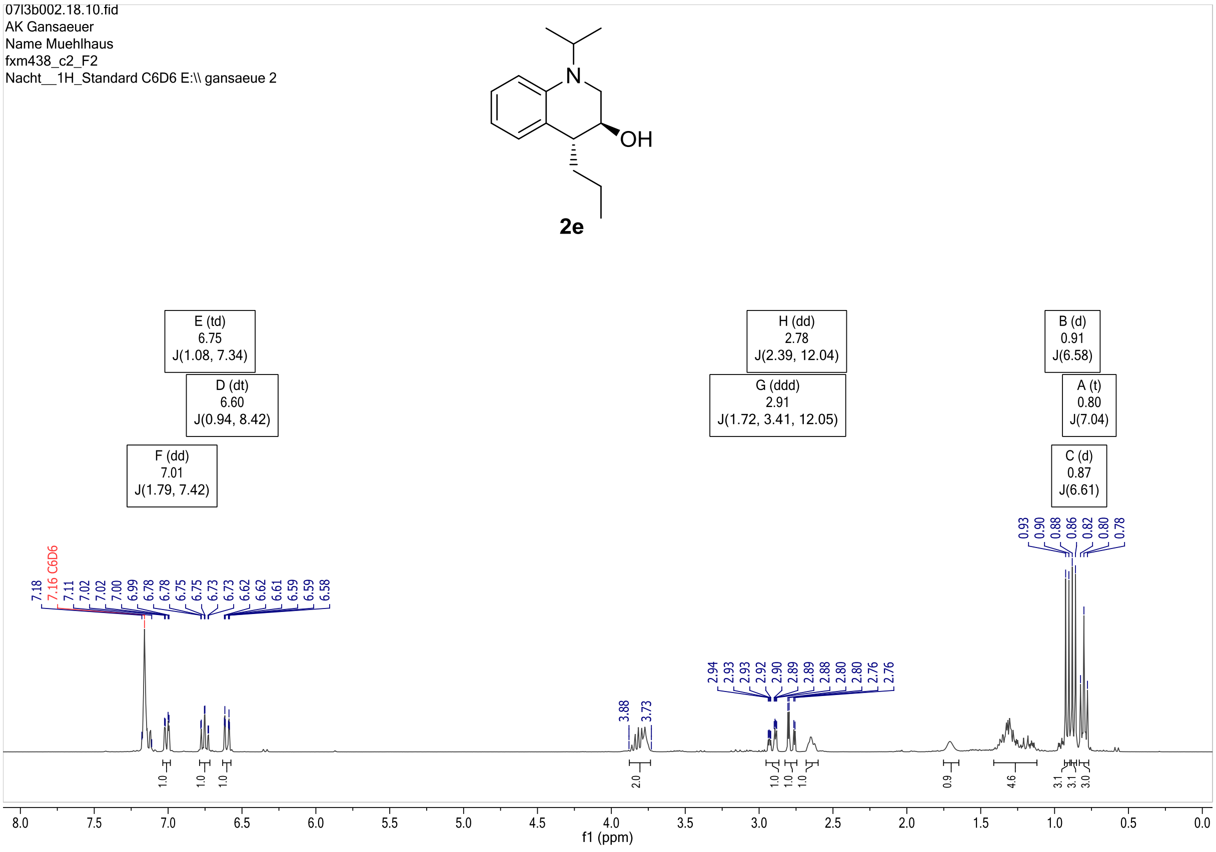
07l3b002.18.10.fid
AK Gansaeuer
Name Muehlhaus
fxm438_c2_F2
Nacht__1H_Standard C6D6 E:\\ gansaeue 2
N
OH
2e
E (td) 6.75 J(1.08, 7.34)
D (dt) 6.60 J(0.94, 8.42)
F (dd) 7.01 J(1.79, 7.42)
H (dd) 2.78 J(2.39, 12.04)
G (ddd) 2.91 J(1.72, 3.41, 12.05)
B (d) 0.91 J(6.58)
A (t) 0.80 J(7.04)
C (d) 0.87 J(6.61)
7.16 C6D6
f1 (ppm)

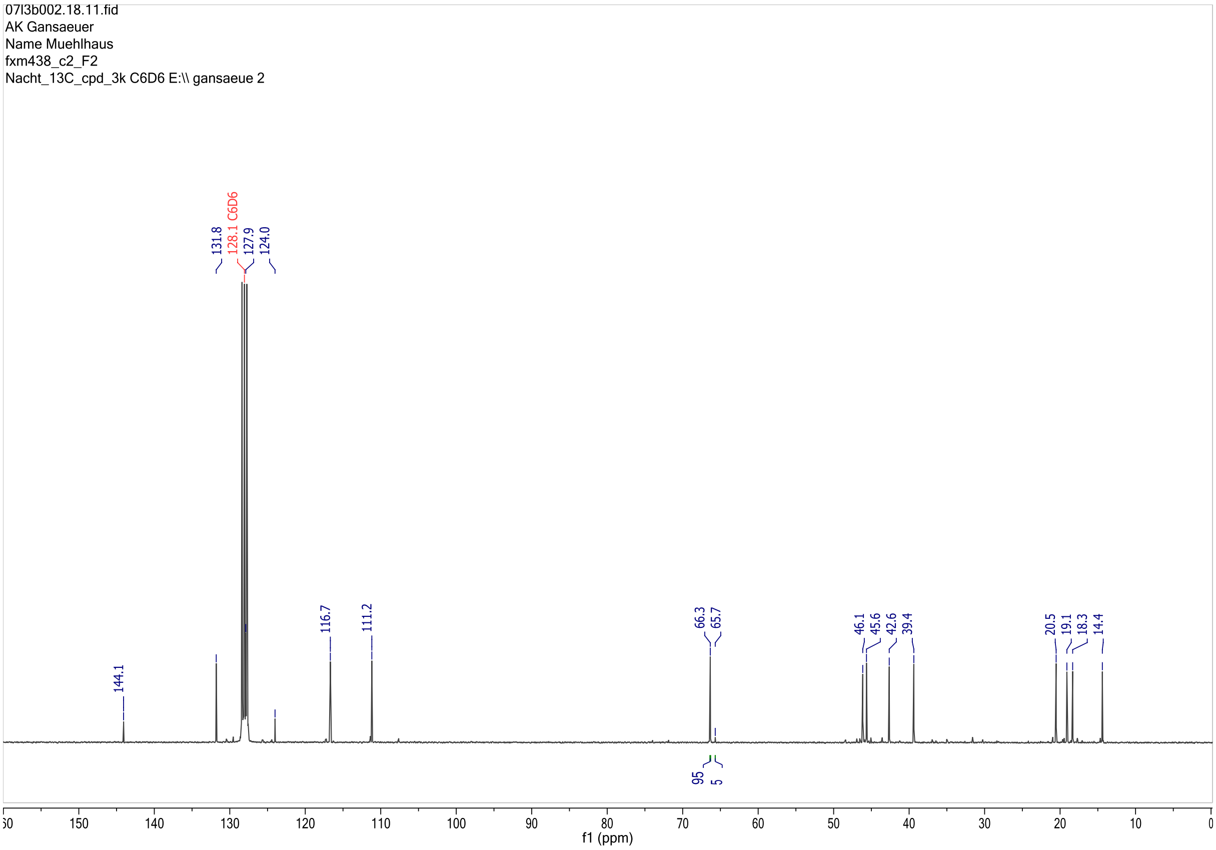
07l3b002.18.11.fid
AK Gansaeuer
Name Muehlhaus
fxm438_c2_F2
Nacht_13C_cpd_3k C6D6 E:\\ gansaeue 2
144.1
131.8
128.1 C6D6
127.9
124.0
116.7
111.2
66.3
65.7
46.1
45.6
42.6
39.4
20.5
19.1
18.3
14.4
f1 (ppm)

4.3.5 Synthesis of (3*S*,4*R*)-1-cyclopropyl-4-propyl-1,2,3,4-tetrahydroquinolin-3-ol (2f).

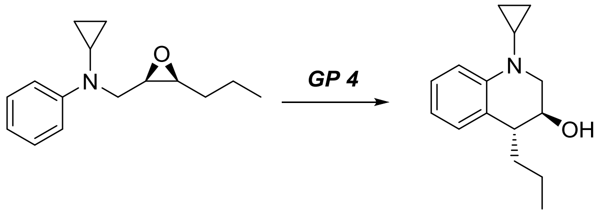

According to GP4: 116 mg of **1f** (1.00 eq., 0.50 mmol), 10 mg zinc (0.30 eq., 0.15 mmol) and 40 mg **cat-(OTs)$_2$** (0.10 eq., 0.05 mmol) are reacted in dry THF for 48 hours at room temperature. Regio- and diastereoisomeric ratio were determined by ^{13}C-NMR of the crude product. Flash chromatography (SiO_2, Eluent: Ch : Mtbe = 90 : 10) yielded 94 mg **2f** (d.r. = 98 : 2, 81%) as a viscous, colorless liquid.

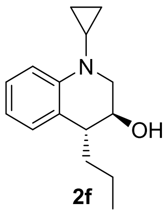

2f

$C_{15}H_{21}NO$
233.35 g/mol

R$_f$= 0.6 (30 % EE in CH);**^{1}H NMR (300.1 MHz, C_6D_6, RT) δ [ppm]**= 0.27-0.53 (m, 4H), 0.80 (t, *J*= 7.0 Hz, 3H), 1.11-1.45 (m, 4H), 1.66-1.88 (m, 1H), 2.03 (tt, *J*= 5.3 Hz, *J*= 4.5 Hz, 1H), 2.65 (td, *J*= 6.6 Hz, *J*= 5.6 Hz, *J*= 2.7 Hz, 1H), 2.98-3.03 (m, 2H), 3.75 (q, *J*= 2.9 Hz, 1H), 6.84 (ddd, *J*= 7.7 Hz, *J*= 4.9 Hz, *J*= 3.6 Hz, 1H), 7.03 (dq, *J*= 7.6 Hz, *J*= 0.8 Hz, 1H), 7.18-7.22 (m, 2H); **^{13}C NMR (75.5 MHz, $CDCl_3$, RT) δ [ppm]**= 8.0, 9.5, 14.4, 20.6, 31.9, 39.7, 45.2, 51.4, 66.9, 113.3, 118.4, 125.1, 127.3, 130.9, 145.5; **IR ν_{max} (neat) [cm^{-1}]=** 491, 527, 745, 778, 939, 1023, 1046, 1181, 1234, 1301, 1362, 1450, 1495, 1601, 2928, 2954**; HRMS (ESI)**: *m/z* calculated for $[M+H]^+$ 232.1696 u found: 232.1702 u.. **$[\alpha]_D^{20}$=** +12.4 °(*c* 1, $CHCl_3$)

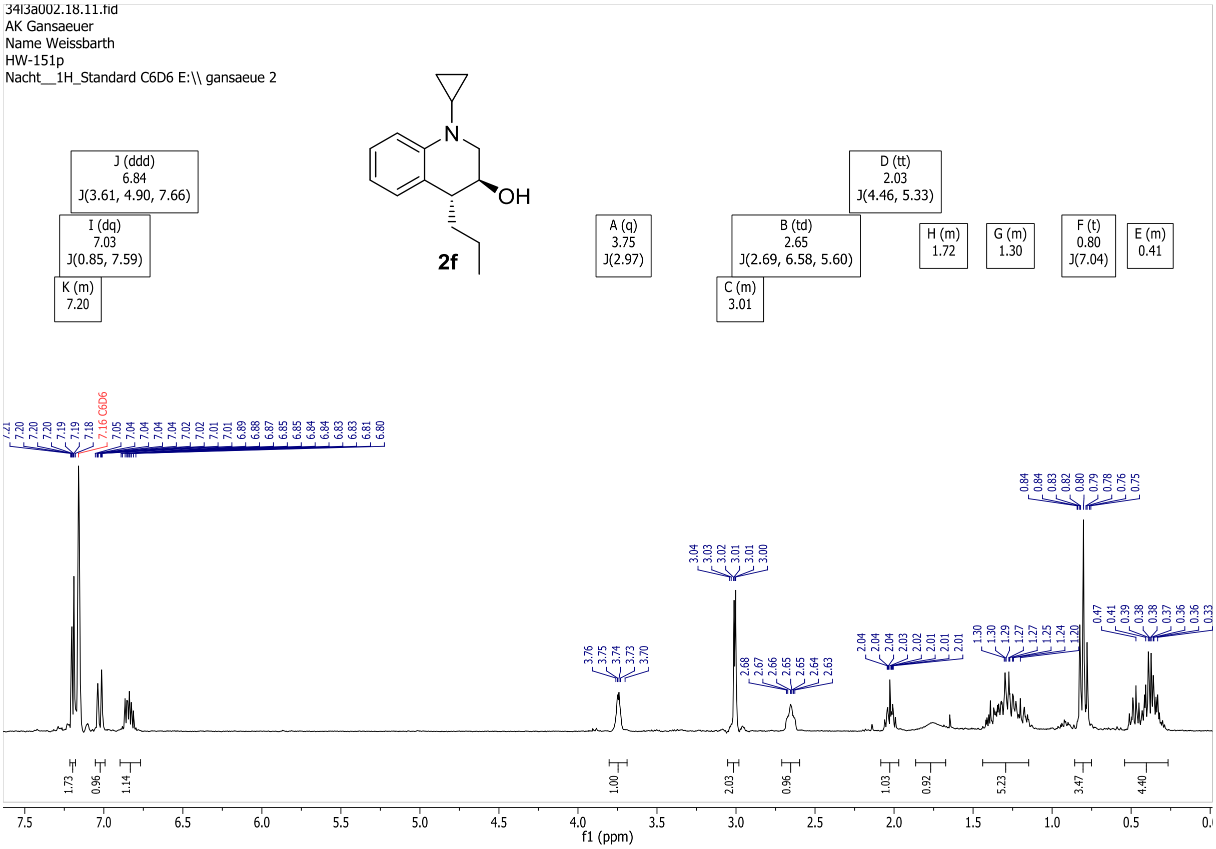
34l3a002.18.11.fid
AK Gansaeuer
Name Weissbarth
HW-151p
Nacht__1H_Standard C6D6 E:\\ gansaeue 2
2f
J (ddd) 6.84 J(3.61, 4.90, 7.66)
I (dq) 7.03 J(0.85, 7.59)
K (m) 7.20
A (q) 3.75 J(2.97)
B (td) 2.65 J(2.69, 6.58, 5.60)
C (m) 3.01
D (tt) 2.03 J(4.46, 5.33)
H (m) 1.72
G (m) 1.30
F (t) 0.80 J(7.04)
E (m) 0.41
7.16 C6D6
f1 (ppm)

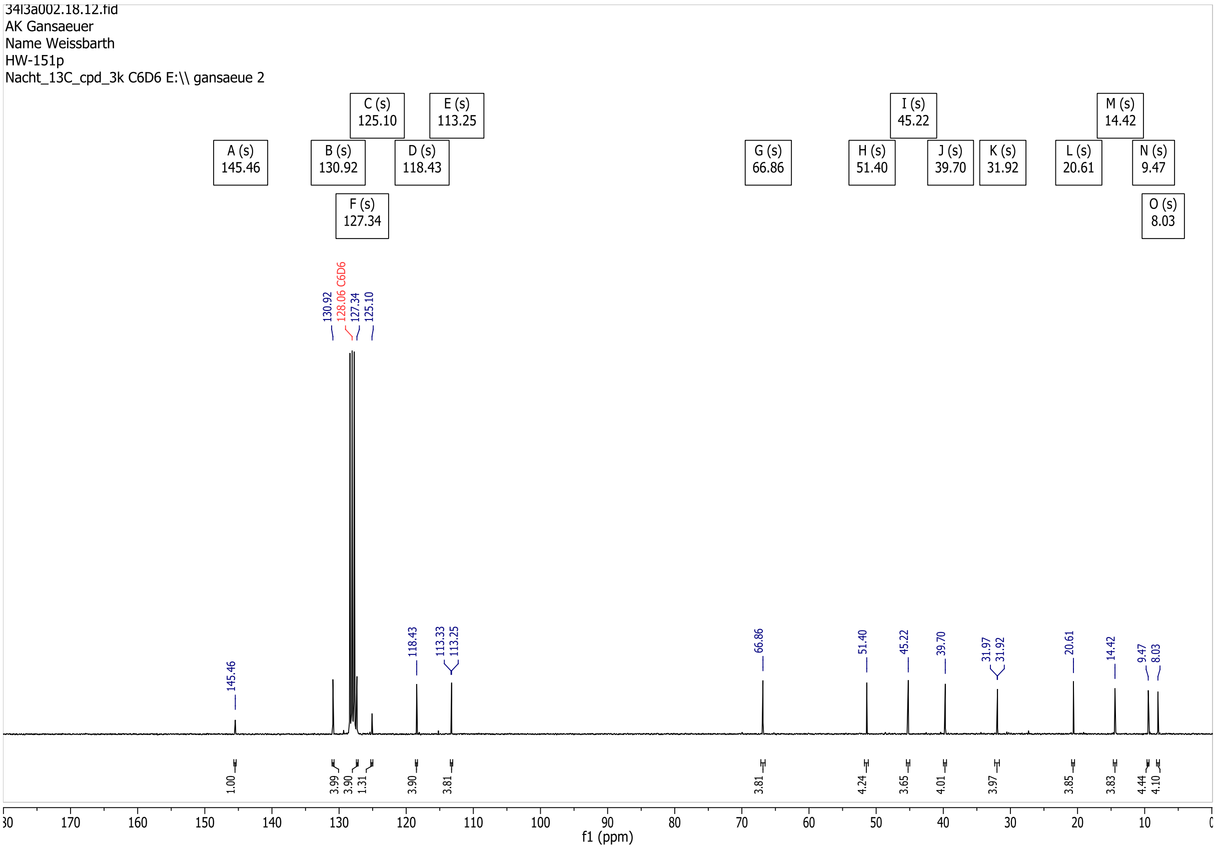
34l3a002.18.12.fid
AK Gansaeuer
Name Weissbarth
HW-151p
Nacht_13C_cpd_3k C6D6 E:\\ gansaeue 2
A (s) 145.46
B (s) 130.92
C (s) 125.10
D (s) 118.43
E (s) 113.25
F (s) 127.34
G (s) 66.86
H (s) 51.40
I (s) 45.22
J (s) 39.70
K (s) 31.92
L (s) 20.61
M (s) 14.42
N (s) 9.47
O (s) 8.03
128.06 C6D6
f1 (ppm)

4.3.6 Synthesis of (3*S*,4*R*)-1-methyl-4-propyl)-1,2,3,4-tetrahydroquinoline-3-ol (2g).

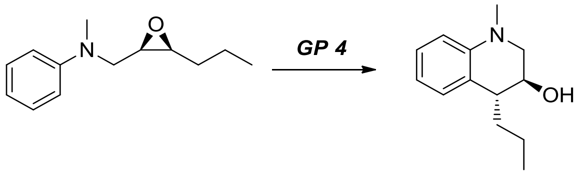

According to GP4: 103 mg of **1g** (1.00 eq., 0.50 mmol), 10 mg zinc (0.30 eq., 0.15 mmol) and 40 mg **cat-(OTs)$_2$** (0.10 eq., 0.05 mmol) are reacted in dry THF for 48 hours at room temperature. Regio- and diastereoisomeric ratio were determined by ^{13}C-NMR of the crude product. Flash chromatography (SiO_2, Eluent: Ch : Mtbe = 87 : 13) was performed rapidly and yielded 91 mg **2g** (d.r. = 96 : 4, 89%) as a transparent-yellow solid (m.p. = 36 °C).

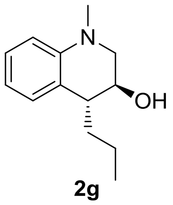

2g
$C_{13}H_{19}NO$
205.30 g/mol

R$_f$ = 0.6 (40% EE in CH), **^{1}H-NMR (500 MHz, C_6D_6, RT): δ [ppm] =** 0.79 (t, 3H, *J* = 7.1 Hz), 1.11-1.35 (m, 4H), 1.96-2.08 (s$_{broad}$, 1H), 2.48 (s, 3H), 2.62-2.67 (m, 1H), 2.78 (ddd, 1H, *J* = 11.9 Hz, *J* = 3.4 Hz, *J* = 1.7 Hz), 2.98 (dd, 1H, *J* = 11.9 Hz, *J* = 2.4 Hz), 3.72 (dd, 1H, *J* = 2.9 Hz, *J* = 2.9 Hz), 6.50 (d, 1H, *J* = 8.3 Hz), 6.75 (dd, 1H, *J* = 7.4 Hz, *J* = 1.2 Hz), 6.98 (dd, 1H, *J* = 7.4 Hz, *J* = 1.6 Hz), 7.13 (ddd, 1H, *J* = 8.5 Hz, *J* = 7.3 Hz, *J* = 1.7 Hz). **^{13}C-NMR (125 MHz, C_6D_6, RT): δ [ppm] =** 14.4, 20.5, 38.5, 39.5, 45.3, 53.5, 66.9, 111.2, 117.2, 123.9, 127.8, 131.1, 145.1. **IR ṽ [cm^{-1}] =** 452, 499, 744, 1056, 1116, 1211, 1294, 1336, 1454, 1505, 1603, 2871, 2930, 2956. **HRMS (ESI):** *m/z* calculated for $C_{13}H_{20}NO^+$: 206.1539 u, found: 206.1536 u. **$[\alpha]_D^{20}$=** -28.0 (*c* 0.2, $CHCl_3$); determination of d.r. by HPLC: *DAICEL Chiralpak* 1A; *n*-Hexane/iPrOH (95:5); flowrate 1.0 mL/min; t_R = 8.7 min (minor, 3*S*4*R*), t_R = 9.6 min (major, 3*R*4*S*); d.r. = 4 : 96 (*cis* : *trans*, **2g** isolated).

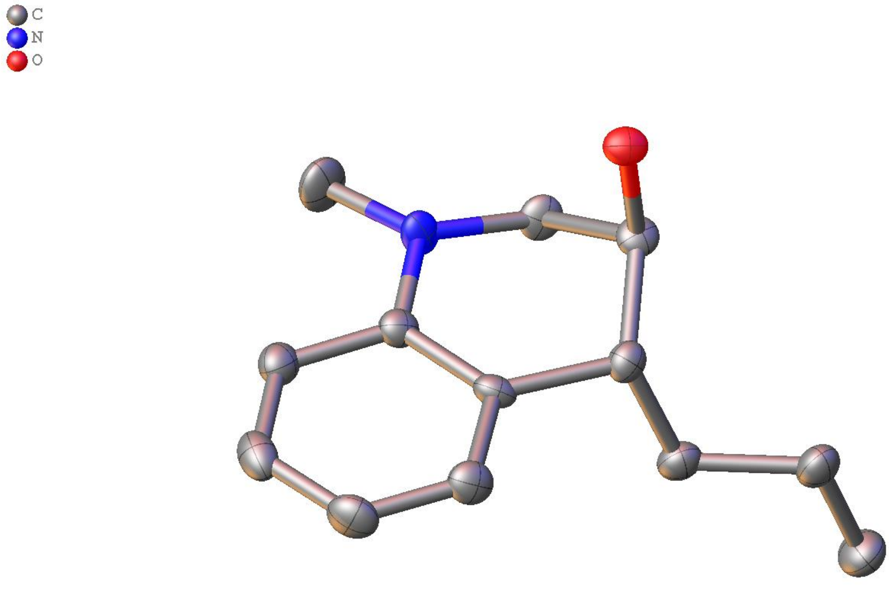

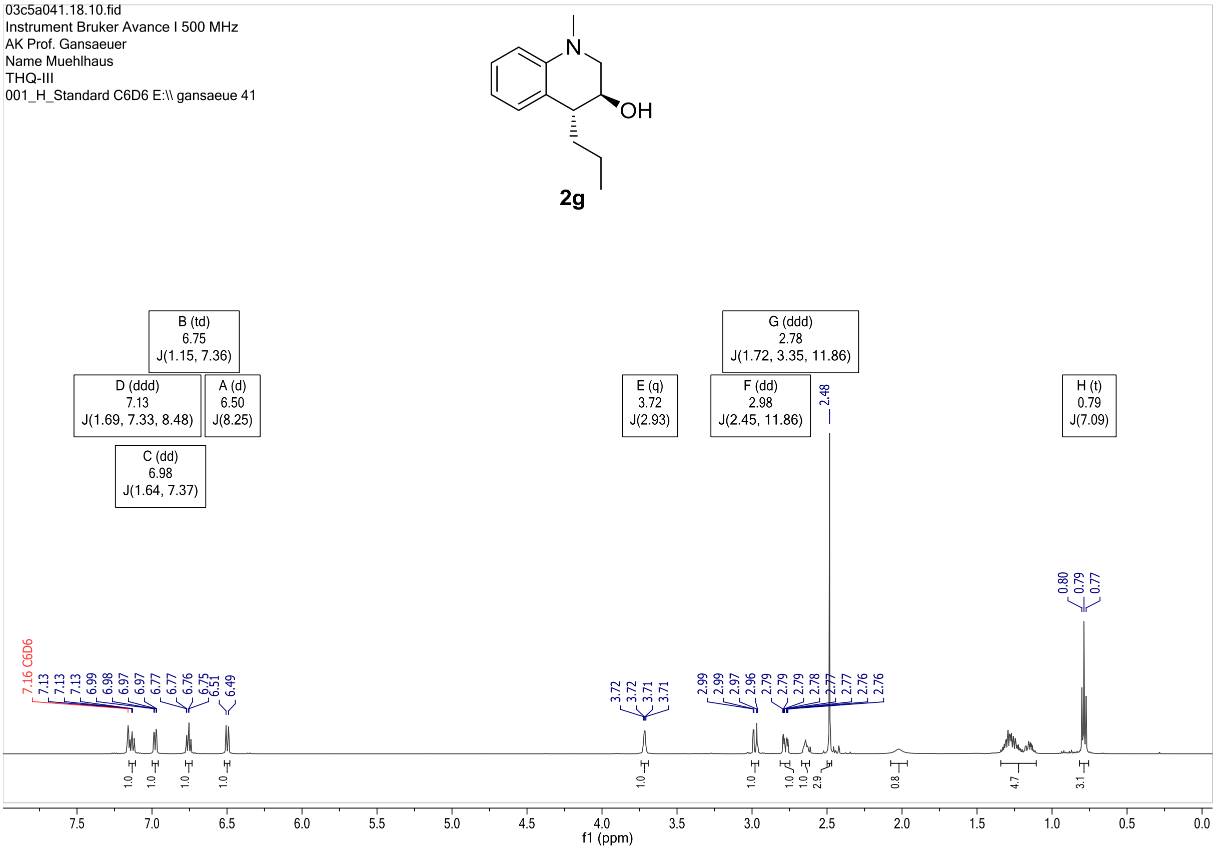
03c5a041.18.10.fid
Instrument Bruker Avance I 500 MHz
AK Prof. Gansaeuer
Name Muehlhaus
THQ-III
001_H_Standard C6D6 E:\\ gansaeue 41
N
OH
2g
B (td) 6.75 J(1.15, 7.36)
D (ddd) 7.13 J(1.69, 7.33, 8.48)
A (d) 6.50 J(8.25)
C (dd) 6.98 J(1.64, 7.37)
E (q) 3.72 J(2.93)
G (ddd) 2.78 J(1.72, 3.35, 11.86)
F (dd) 2.98 J(2.45, 11.86)
H (t) 0.79 J(7.09)
7.16 C6D6
7.13 7.13 7.13 6.99 6.98 6.97 6.97 6.77 6.77 6.76 6.76 6.75 6.51 6.49
3.72 3.72 3.71 3.71
2.99 2.99 2.97 2.96 2.79 2.79 2.79 2.78 2.77 2.77 2.76 2.76
2.48
0.80 0.79 0.77
1.0 1.0 1.0 1.0 1.0 1.0 1.0 1.0 2.9 0.8 4.7 3.1
f1 (ppm)

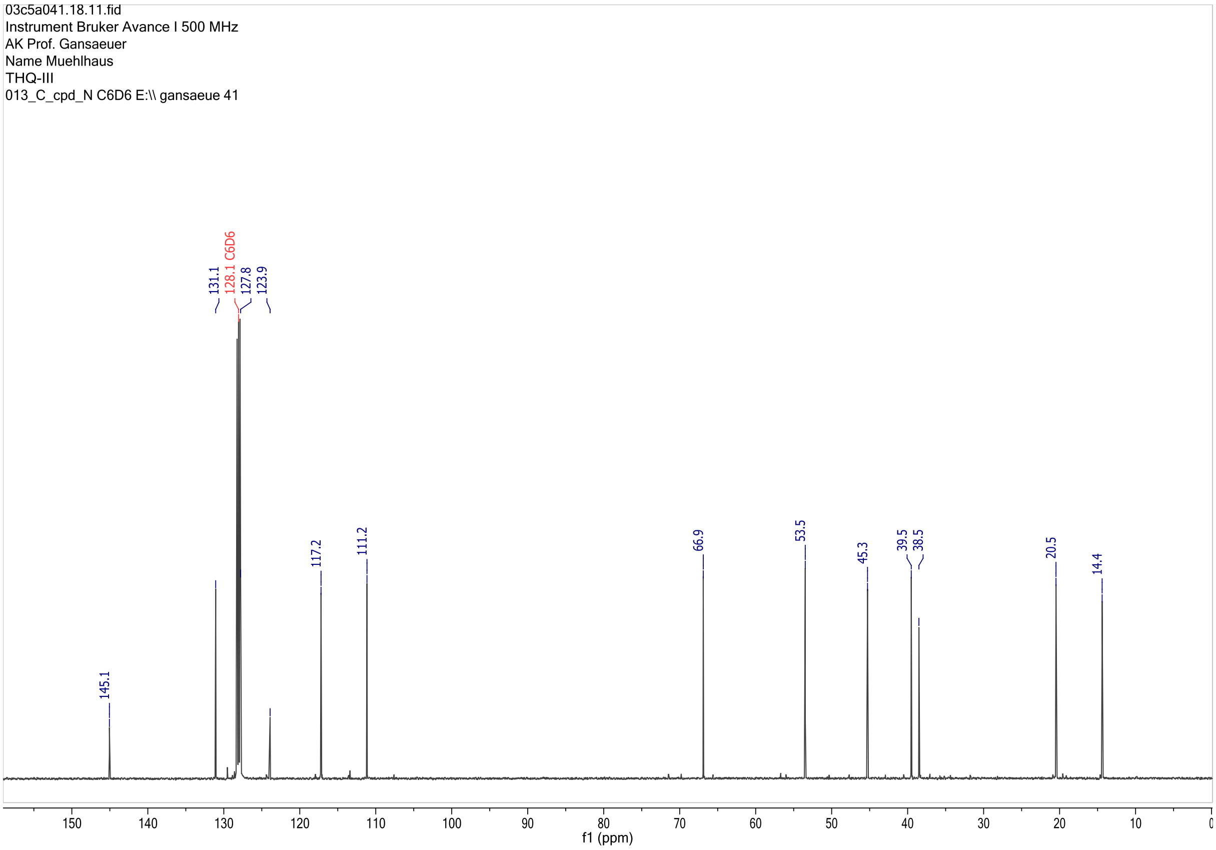
03c5a041.18.11.fid
Instrument Bruker Avance I 500 MHz
AK Prof. Gansaeuer
Name Muehlhaus
THQ-III
013_C_cpd_N C6D6 E:\\ gansaeue 41
145.1
131.1
128.1 C6D6
127.8
123.9
117.2
111.2
66.9
53.5
45.3
39.5
38.5
20.5
14.4
f1 (ppm)

4.3.7 Synthesis of (3*S*,4*R*)-6-chloro-1-cyclohexyl-4-propyl-1,2,3,4-tetrahydroquinolin-3-ol (2h).

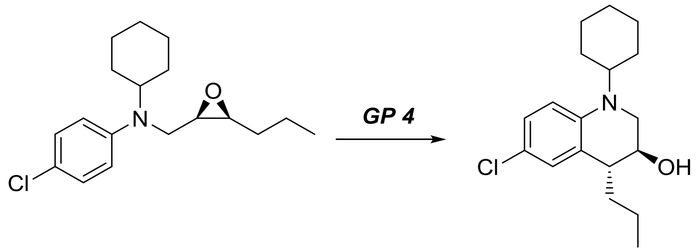

According to GP4: 154 mg of **1h** (1.00 eq., 0.50 mmol), 10 mg zinc (0.30 eq., 0.15 mmol) and 40 mg **cat-(OTs)$_2$** (0.10 eq., 0.05 mmol) are reacted in dry THF for 48 hours at room temperature. Regio- and diastereoisomeric ratio were determined by ^{13}C-NMR of the crude product. Flash chromatography (SiO_2, Eluent: Ch : Mtbe = 90 : 10) yielded 119 mg **2h** (d.r. = >98 : <2, 77%) as a viscous, colorless liquid.

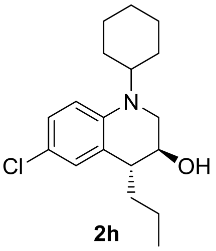

2h

$C_{18}H_{26}ClNO$
307.86 g/mol

R$_f$= 0.4 (20 % EE in CH)**; ^{1}H NMR (500.1 MHz, C_6D_6, RT) δ [ppm]**= 0.75 (t, *J*= 6.9 Hz, 3H), 0.86-0,98 (m, 1H), 1.02-1.27 (m, 8H), 1.45-1.66 (m, 6H), 2.47 (ddt, *J*= 8.6 Hz, *J*= 5.7 Hz, *J*= 2.3, Hz, 1H), 2.90 (ddd, *J*= 12.2 Hz, *J*= 3.2 Hz, *J*= 1.7 Hz, 1H), 3.28 (tq, *J*= 11.2 Hz, *J*= 3.8 Hz, 1H), 3.69 (q, *J*= 2.7 Hz, 1H), 6.42 (d, *J*= 8.9 Hz, 1H), 7.05 (d, *J*= 2.6 Hz, 1H), 7.12 (dt, *J*= 9.0 Hz, *J*= 1.8 Hz, 1H); **^{13}C NMR (125 MHz, $CDCl_3$, RT) δ [ppm]**= 14.1, 20.4, 26.2, 26.3, 29.4, 29.5, 38.9, 44.1, 45.4, 55.8, 66.1, 112.2, 120.9, 125.8, 127.6, 131.2, 142.6; **IR ν_{max} (neat) [cm^{-1}]=** 405, 445, 546, 605, 654, 750, 791, 835, 871, 891, 956, 1062, 1107, 1171, 1241, 1263, 1299, 1343, 1378, 1421, 1451, 1494, 1596, 1659, 2854, 2928, 3348**; HRMS (ESI)**: *m/z* calculated for $[M+H]^+$ 308.1776 u found: 308.1772 u. **$[\alpha]_D^{20}$=** -22.3 °(*c* 1, $CHCl_3$)

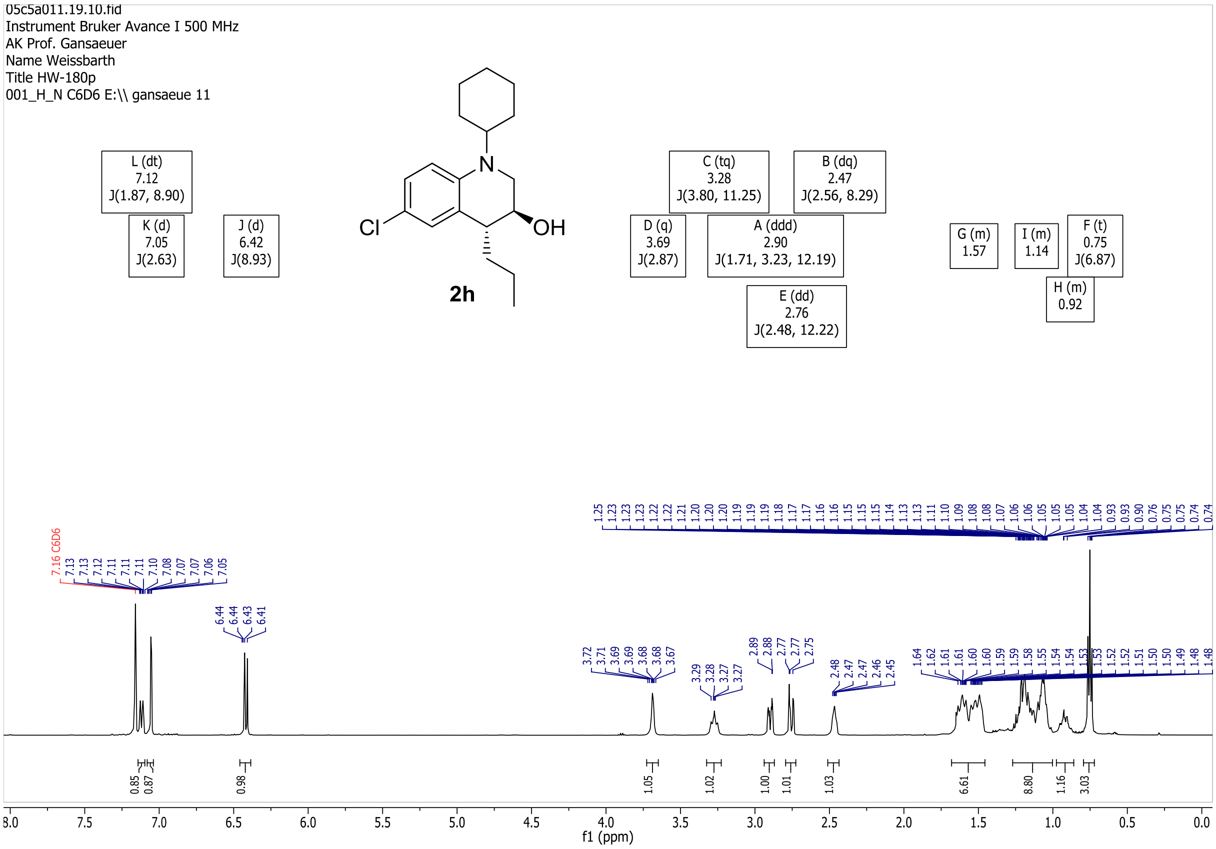
05c5a011.19.10.fid
Instrument Bruker Avance I 500 MHz
AK Prof. Gansaeuer
Name Weissbarth
Title HW-180p
001_H_N C6D6 E:\\ gansaeue 11
Cl
N
OH
2h
L (dt) 7.12 J(1.87, 8.90)
K (d) 7.05 J(2.63)
J (d) 6.42 J(8.93)
C (tq) 3.28 J(3.80, 11.25)
B (dq) 2.47 J(2.56, 8.29)
D (q) 3.69 J(2.87)
A (ddd) 2.90 J(1.71, 3.23, 12.19)
E (dd) 2.76 J(2.48, 12.22)
G (m) 1.57
I (m) 1.14
F (t) 0.75 J(6.87)
H (m) 0.92
7.16 C6D6
f1 (ppm)

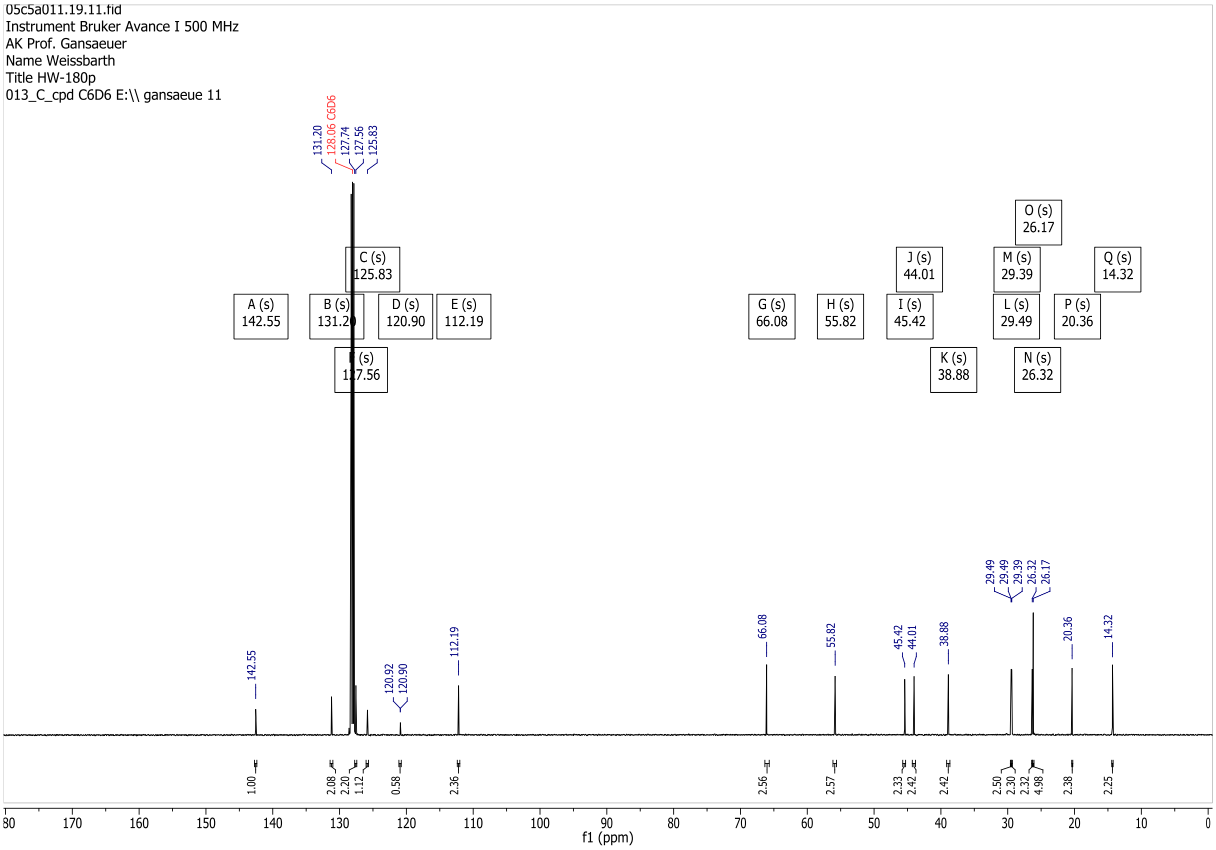
05c5a011.19.11.fid
Instrument Bruker Avance I 500 MHz
AK Prof. Gansaeuer
Name Weissbarth
Title HW-180p
013_C_cpd C6D6 E:\\ gansaeue 11
128.06 C6D6
A (s) 142.55
B (s) 131.20
C (s) 125.83
D (s) 120.90
E (s) 112.19
F (s) 127.56
G (s) 66.08
H (s) 55.82
I (s) 45.42
J (s) 44.01
K (s) 38.88
L (s) 29.49
M (s) 29.39
N (s) 26.32
O (s) 26.17
P (s) 20.36
Q (s) 14.32
f1 (ppm)

4.3.8 Synthesis of (3*S*,4*R*)-6-chloro-1-isopropyl-4-propyl-1,2,3,4-tetrahydroquinolin-3-ol (2j).

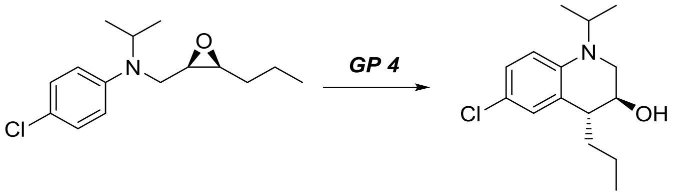

According to GP4: 134 mg of **1j** (1.00 eq., 0.50 mmol), 10 mg zinc (0.30 eq., 0.15 mmol) and 40 mg **cat-(OTs)$_2$** (0.10 eq., 0.05 mmol) are reacted in dry THF for 48 hours at room temperature. Regio- and diastereoisomeric ratio were determined by ^{13}C-NMR of the crude product. Flash chromatography (SiO_2, Eluent: Ch : Mtbe = 90 : 10) yielded 112 mg **2j** (d.r. = >98 : <2, 84%) as a viscous, colorless liquid.

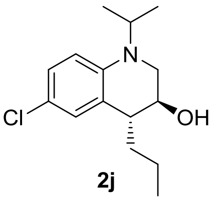

2j

$C_{15}H_{22}ClNO$
267,80 g/mol

R$_f$= 0.3 (30 % MTBE in CH); **^{1}H NMR (300.1 MHz, C_6D_6, RT) δ [ppm]**= 0.70-0.76 (m, 3H), 0.79 (d, *J*= 6.6 Hz, 3H), 0.85 (d, *J*= 6.5 Hz, 3H), 0.91-1.28 (m, 4H), 1.72 (s$_{br}$, 1H), 2.44 (tt, *J*= 6.6 Hz, *J*= 2.2, Hz, 1H), 2.66 (dd, *J*= 12.2 Hz, *J*= 2.5 Hz, 1H), 2.81 (ddd, *J*= 12.2 Hz, *J*= 3.3 Hz, *J*= 1.7 Hz, 1H), 3.60 (h, *J*= 6.6 Hz, 1H), 3.67 (q, *J*= 2.9 Hz, 1H), 6.29 (dd, *J*= 8.8 Hz, *J*= 0.8 Hz, 1H), 7.03 (dd, *J*= 2.6 Hz, *J*= 0.7 Hz, 1H), 7.10 (dd, *J*= 8.9 Hz, *J*= 2.6 Hz, 1H); **^{13}C NMR (75 MHz, $CDCl_3$, RT) δ [ppm]**=14.3, 18.3, 18.9, 20.4, 38.9, 42.4, 45.5, 46.4, 65.9, 112.3, 121.0, 125.9, 127.6, 131.1, 142.6; **IR ν_{max} (neat) [cm^{-1}]=** 538, 629, 785, 796, 874, 1051, 1195, 1264, 1306, 1494; **HRMS (ESI)**: *m/z* calculated for $[M+H]^+$ 268.1463 u found: 268.1460 u. **$[\alpha]_D^{20}$=** -47.8 °(*c* 1, $CHCl_3$)

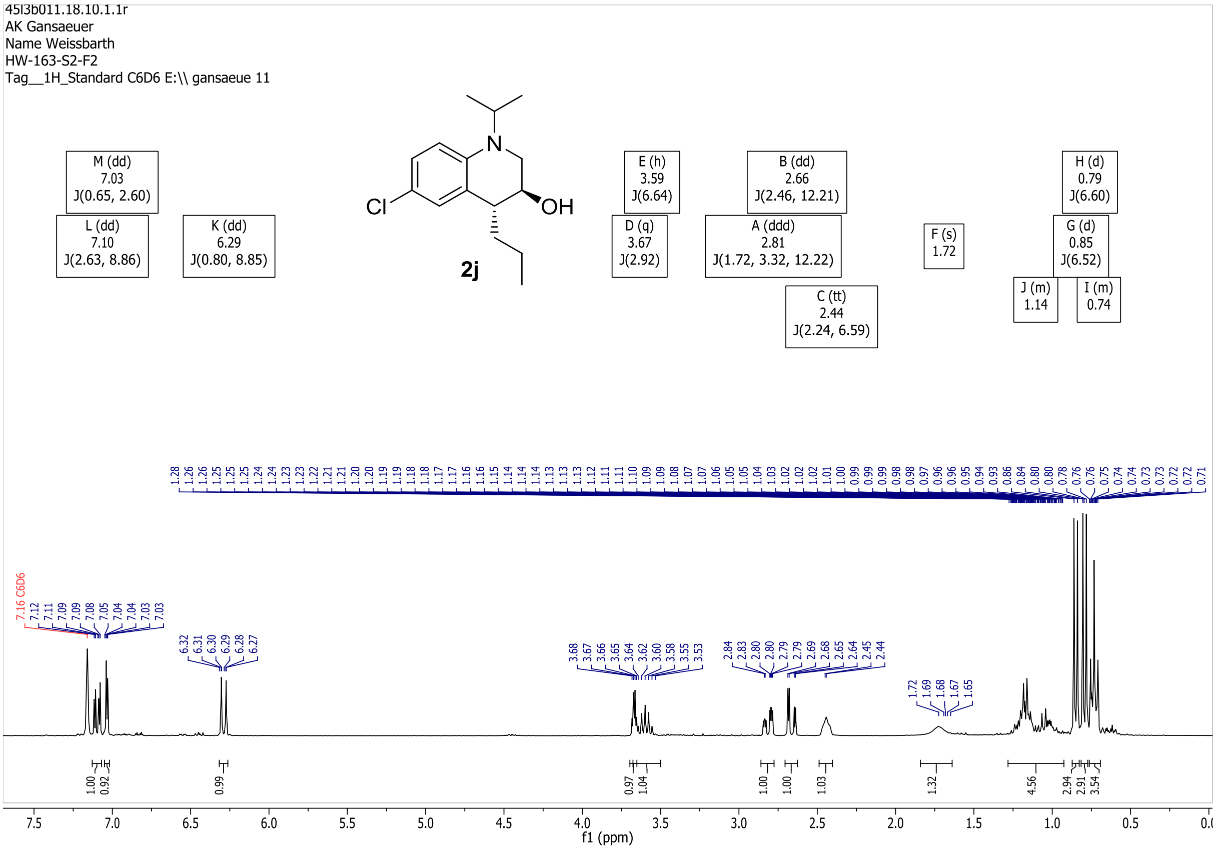
45l3b011.18.10.1.1r
AK Gansaeuer
Name Weissbarth
HW-163-S2-F2
Tag__1H_Standard C6D6 E:\\ gansaeue 11
2j
Cl
N
OH
M (dd) 7.03 J(0.65, 2.60)
L (dd) 7.10 J(2.63, 8.86)
K (dd) 6.29 J(0.80, 8.85)
E (h) 3.59 J(6.64)
D (q) 3.67 J(2.92)
B (dd) 2.66 J(2.46, 12.21)
A (ddd) 2.81 J(1.72, 3.32, 12.22)
C (tt) 2.44 J(2.24, 6.59)
F (s) 1.72
H (d) 0.79 J(6.60)
G (d) 0.85 J(6.52)
J (m) 1.14
I (m) 0.74
7.16 C6D6
f1 (ppm)

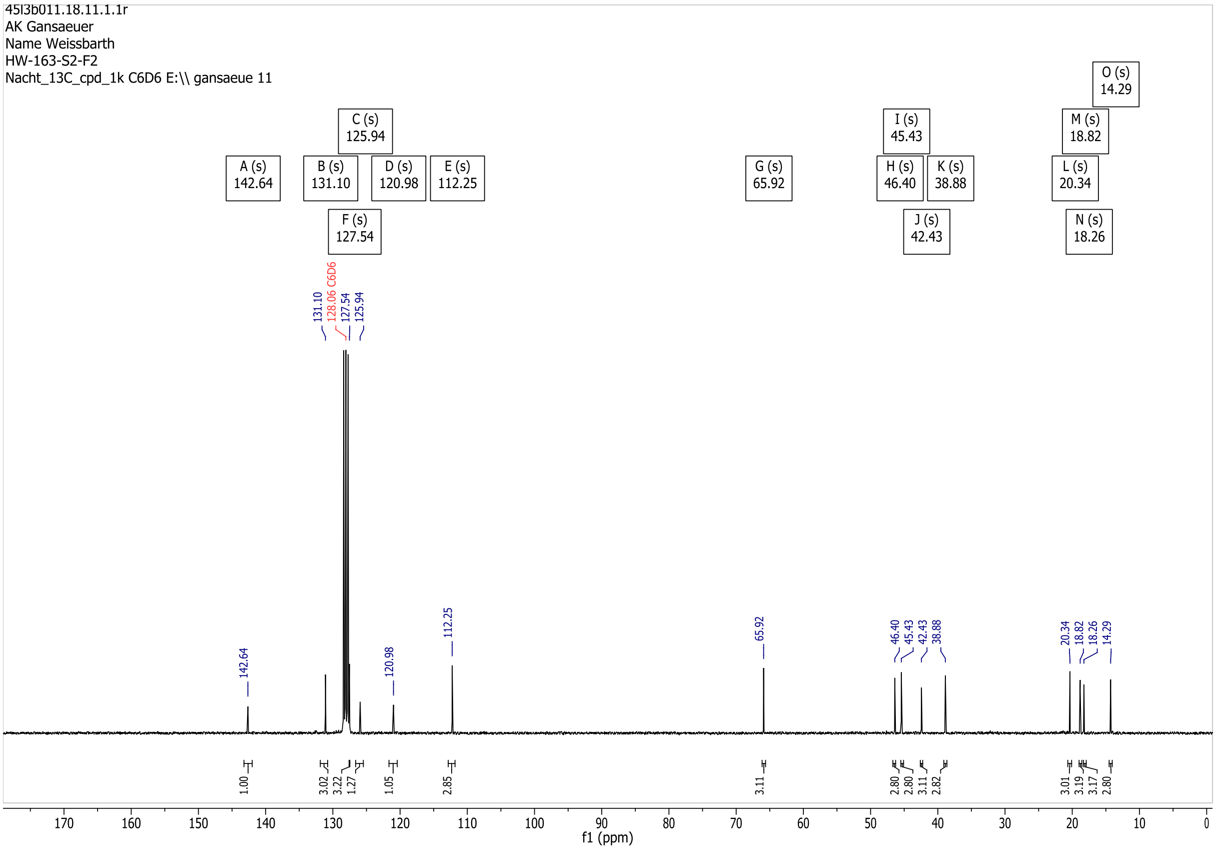
45l3b011.18.11.1.1r
AK Gansaeuer
Name Weissbarth
HW-163-S2-F2
Nacht_13C_cpd_1k C6D6 E:\\ gansaeue 11
A (s) 142.64
B (s) 131.10
C (s) 125.94
D (s) 120.98
E (s) 112.25
F (s) 127.54
G (s) 65.92
H (s) 46.40
I (s) 45.43
J (s) 42.43
K (s) 38.88
L (s) 20.34
M (s) 18.82
N (s) 18.26
O (s) 14.29
128.06 C6D6
f1 (ppm)

4.3.9 (3*S*,4*R*)-1-cyclopropyl-6-fluoro-4-propyl-1,2,3,4-tetrahydroquinolin-3-ol (2l).

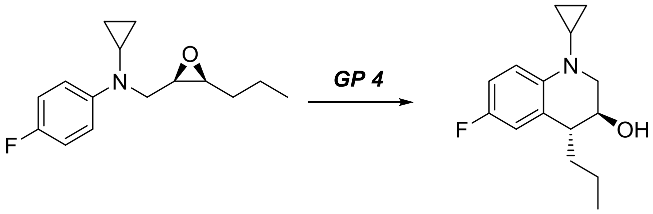

According to GP4: 125 mg of **1l** (1.00 eq., 0.50 mmol), 10 mg zinc (0.30 eq., 0.15 mmol) and 40 mg **cat-(OTs)$_2$** (0.10 eq., 0.05 mmol) are reacted in dry THF for 48 hours at room temperature. Regio- and diastereoisomeric ratio were determined by ^{13}C-NMR of the crude product. Flash chromatography (SiO_2, Eluent: CH : MTBE = 90 : 10) yielded 98 mg **2l** (d.r. = 97 : 3, 79%) as a viscous, colorless liquid.

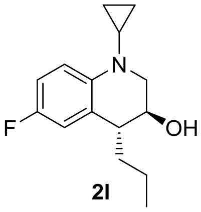

2l

$C_{15}H_{20}FNO$
249,33 g/mol

R$_f$= 0.3 (20 % EE in CH); **^{1}H NMR (500.1 MHz, C_6D_6, RT) δ [ppm]**= 0.26-0.49 (m, 4H), 0.76 (t, *J*= 7.2 Hz, 3H), 1.07-1.34 (m, 4H), 1.92 (tt, *J*= 6.4 Hz, *J*= 3.8 Hz, 2H), 2.48-2.55 (m, 1H), 2.89 (dd, *J*= 11.8 Hz, *J*= 2.4 Hz, 1H), 2.93 (ddd, *J*= 11.8 Hz, *J*= 3.9 Hz, *J*= 1.4 Hz, 1H), 3.67 (q, *J*= 3.0 Hz, 1H), 6.80 (ddd, *J*= 9.2 Hz, *J*= 3.0 Hz, *J*= 0.8 Hz, 1H), 6.87 (td, *J*= 8.7 Hz, *J*= 3.0 Hz, 1H), 6.97 (dd, *J*= 9.0 Hz, *J*= 5.0 Hz, 1H); **^{13}C NMR (125 MHz, $CDCl_3$, RT) δ [ppm]**=7.9, 9.5, 14.4, 20.5, 32.2, 39.4, 45.3 (d, $J_{C,F}$= 1.2 Hz), 51.5, 66.7, 113.5 (d, $J_{C,F}$= 21.6 Hz), 113.9 (d, $J_{C,F}$= 7.3 Hz), 117.0 (d, $J_{C,F}$= 21.7 Hz), 127.2 (d, $J_{C,F}$= 6.3 Hz), 141.9 (d, $J_{C,F}$= 1.8 Hz), 156.8 (d, $J_{C,F}$= 235.7 Hz); **^{19}F NMR (470 MHz, C_6D_6, RT) δ [ppm]**= -127.3; **IR ν_{max} (neat) [cm^{-1}]=** 705, 803, 865, 1025, 1055, 1208, 1363, 1498; **HRMS (ESI)**: *m/z* calculated for $[M+H]^+$ 250.1602 u found: 250.1610 u. **$[\alpha]_D^{20}$=** +15.7 °(*c* 1, $CHCl_3$)

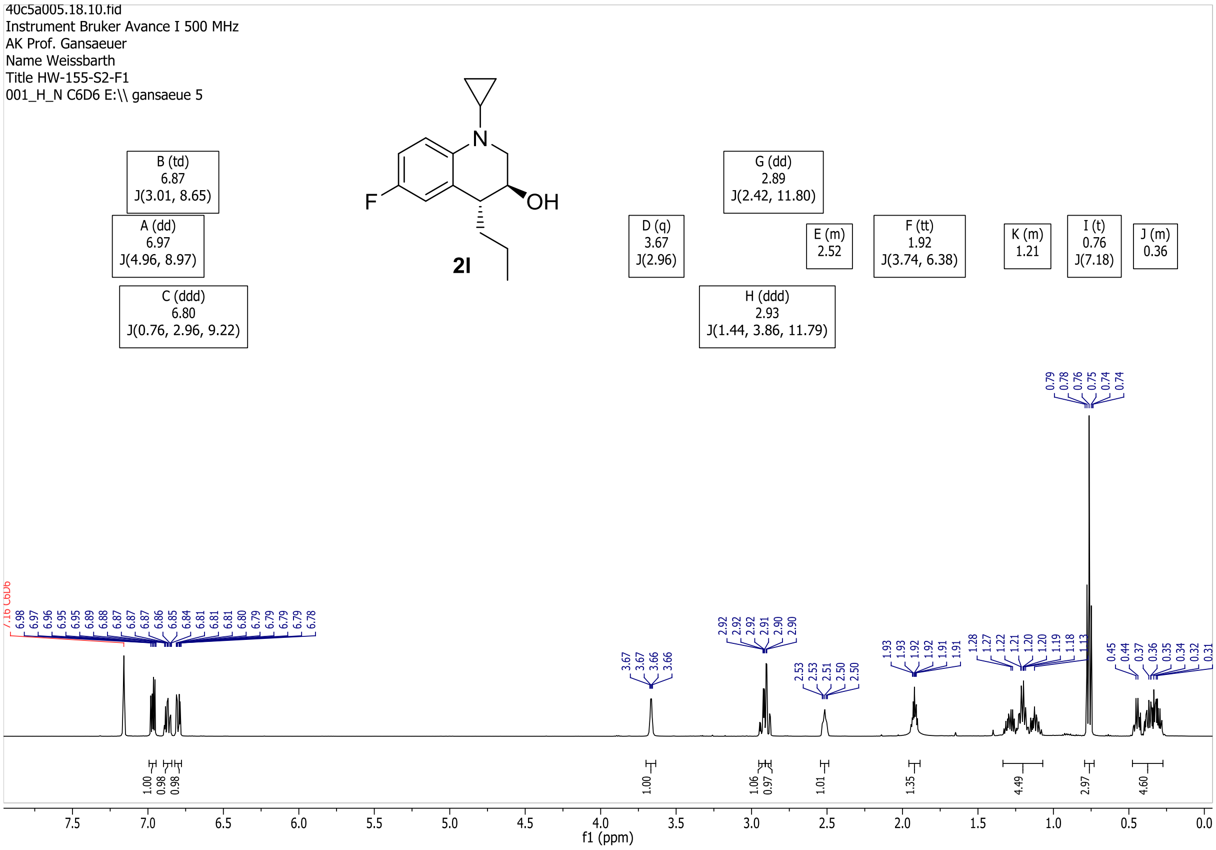
40c5a005.18.10.fid
Instrument Bruker Avance I 500 MHz
AK Prof. Gansaeuer
Name Weissbarth
Title HW-155-S2-F1
001_H_N C6D6 E:\\ gansaeue 5
F
N
OH
2l
B (td) 6.87 J(3.01, 8.65)
A (dd) 6.97 J(4.96, 8.97)
C (ddd) 6.80 J(0.76, 2.96, 9.22)
D (q) 3.67 J(2.96)
G (dd) 2.89 J(2.42, 11.80)
H (ddd) 2.93 J(1.44, 3.86, 11.79)
E (m) 2.52
F (tt) 1.92 J(3.74, 6.38)
K (m) 1.21
I (t) 0.76 J(7.18)
J (m) 0.36
7.16 C6D6
f1 (ppm)

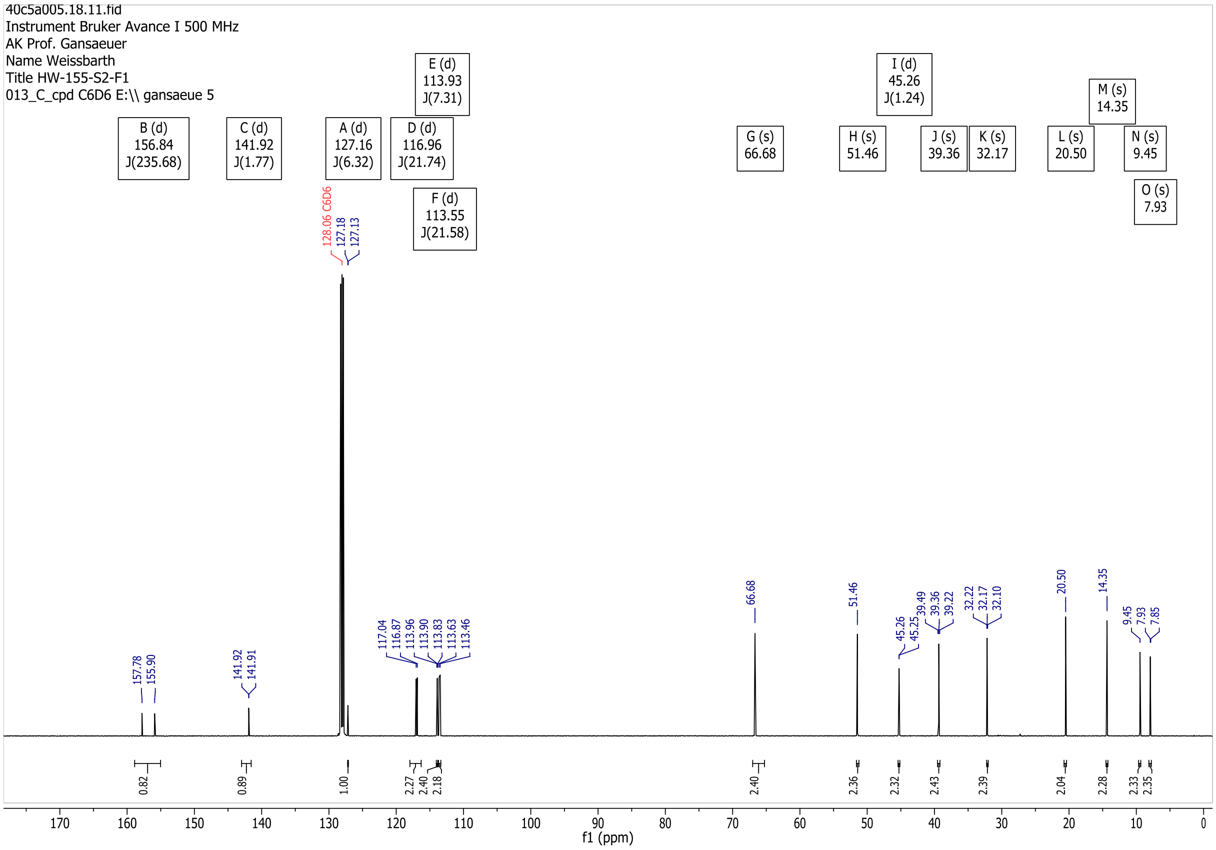
40c5a005.18.11.fid
Instrument Bruker Avance I 500 MHz
AK Prof. Gansaeuer
Name Weissbarth
Title HW-155-S2-F1
013_C_cpd C6D6 E:\\ gansaeue 5
B (d) 156.84 J(235.68)
C (d) 141.92 J(1.77)
A (d) 127.16 J(6.32)
D (d) 116.96 J(21.74)
E (d) 113.93 J(7.31)
F (d) 113.55 J(21.58)
G (s) 66.68
H (s) 51.46
I (d) 45.26 J(1.24)
J (s) 39.36
K (s) 32.17
L (s) 20.50
M (s) 14.35
N (s) 9.45
O (s) 7.93
128.06 C6D6
f1 (ppm)

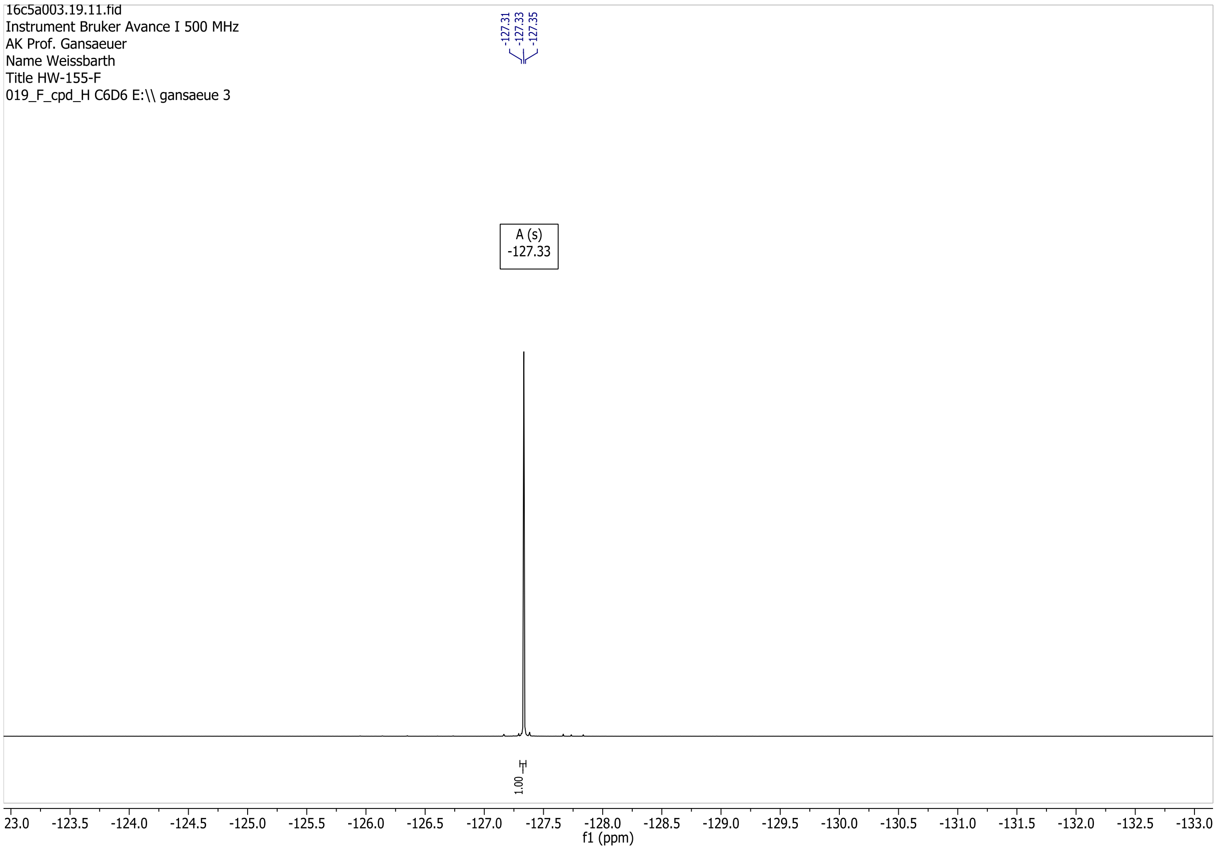
16c5a003.19.11.fid
Instrument Bruker Avance I 500 MHz
AK Prof. Gansaeuer
Name Weissbarth
Title HW-155-F
019_F_cpd_H C6D6 E:\\ gansaeue 3
-127.31
-127.33
-127.35
A (s)
-127.33
1.00
f1 (ppm)

4.3.10 Synthesis of (3*S*,4*R*)-6-chloro-1-methyl-4-propyl)-1,2,3,4-tetrahydroquinoline-3-ol (2m).

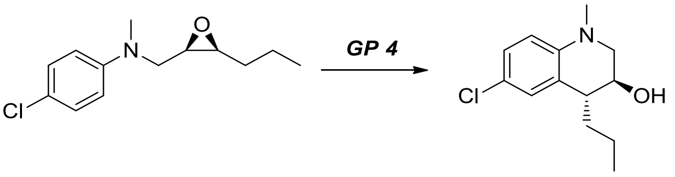

According to GP4: 120 mg of **1m** (1.00 eq., 0.50 mmol), 10 mg zinc (0.30 eq., 0.15 mmol) and 40 mg **cat-(OTs)$_2$** (0.10 eq., 0.05 mmol) are reacted in dry THF for 48 hours at room temperature. Regio- and diastereoisomeric ratio were determined by ^{13}C-NMR of the crude product. Flash chromatography (SiO_2, Eluent: Ch : Mtbe = 87 : 13) was performed rapidly and yielded 97 mg **2m** (d.r. = 98 : 2, 81%) as a viscous, yellow liquid.

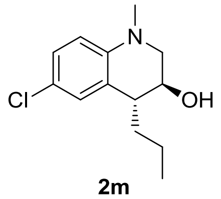

2m
$C_{13}H_{18}ClNO$
239.74 g/mol

R$_f$ = 0.5 (40% EE in CH), **^{1}H-NMR (300 MHz, C_6D_6, RT): δ [ppm]** = 0.73 (t, 3H, *J* = 7.0 Hz), 0.97-1.22 (m, 4H), 2.35 (s, 3H), 2.44 (dtd, 1H, *J* = 8.7 Hz, *J* = 5.9 Hz, *J* = 3.2 Hz), 2.67 (ddd, 1H, *J* = 12.6 Hz, *J* = 3.3 Hz, *J* = 1.7 Hz), 2.86 (dd, 1H, *J* = 12.6 Hz, *J* = 2.4 Hz), 3.60 (q, 1H, *J* = 2.9 Hz), 6.19 (d, 1H, *J* = 8.7 Hz), 7.00 (dd, 1H, *J* = 2.6 Hz, *J* = 0.7 Hz), 7.08 (dd, 1H, *J* = 8.7 Hz, *J* = 2.6 Hz). **^{13}C-NMR (75 MHz, C_6D_6, RT): δ [ppm]** = 14.3, 20.3, 38.4, 39.1, 45.2, 53.1, 66.5, 112.2, 121.7, 125.7, 127.5, 130.6, 143.5. IR ṽ [cm^{-1}] = 507, 629, 764, 808, 957, 1097, 1120 1190, 1243, 1368, 1455, 1596, 2960. **HRMS (ESI):** *m/z* calculated for $C_{13}H_{19}ClNO^+$: 240.1150 u; found: 240.1148 u. **[α]$_D$ ^{20}**=-35.4° (*c* 0.5, $CHCl_3$). d.r. = 2 : 98 (*cis* : *trans*, **2i** isolated).

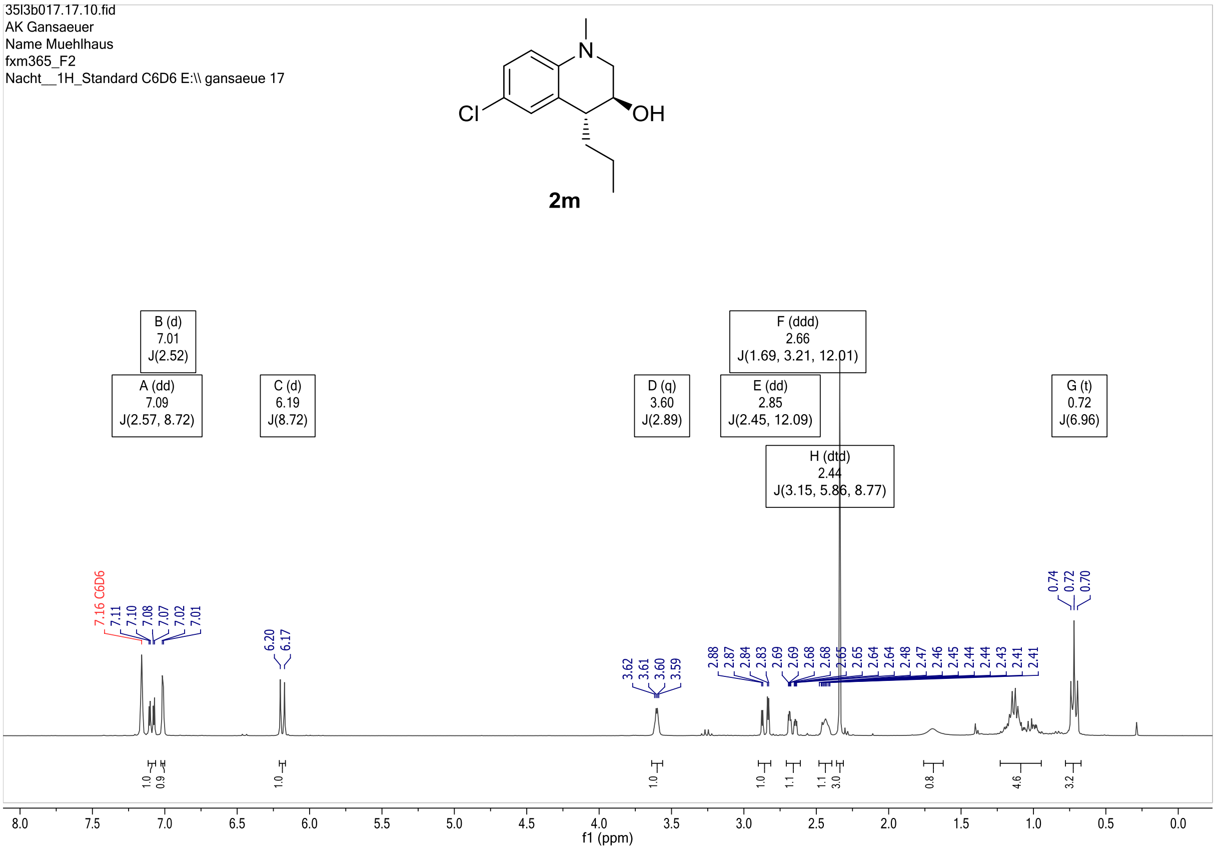
35l3b017.17.10.fid
AK Gansaeuer
Name Muehlhaus
fxm365_F2
Nacht__1H_Standard C6D6 E:\\ gansaeue 17
2m
A (dd) 7.09 J(2.57, 8.72)
B (d) 7.01 J(2.52)
C (d) 6.19 J(8.72)
D (q) 3.60 J(2.89)
E (dd) 2.85 J(2.45, 12.09)
F (ddd) 2.66 J(1.69, 3.21, 12.01)
G (t) 0.72 J(6.96)
H (dtd) 2.44 J(3.15, 5.86, 8.77)
f1 (ppm)

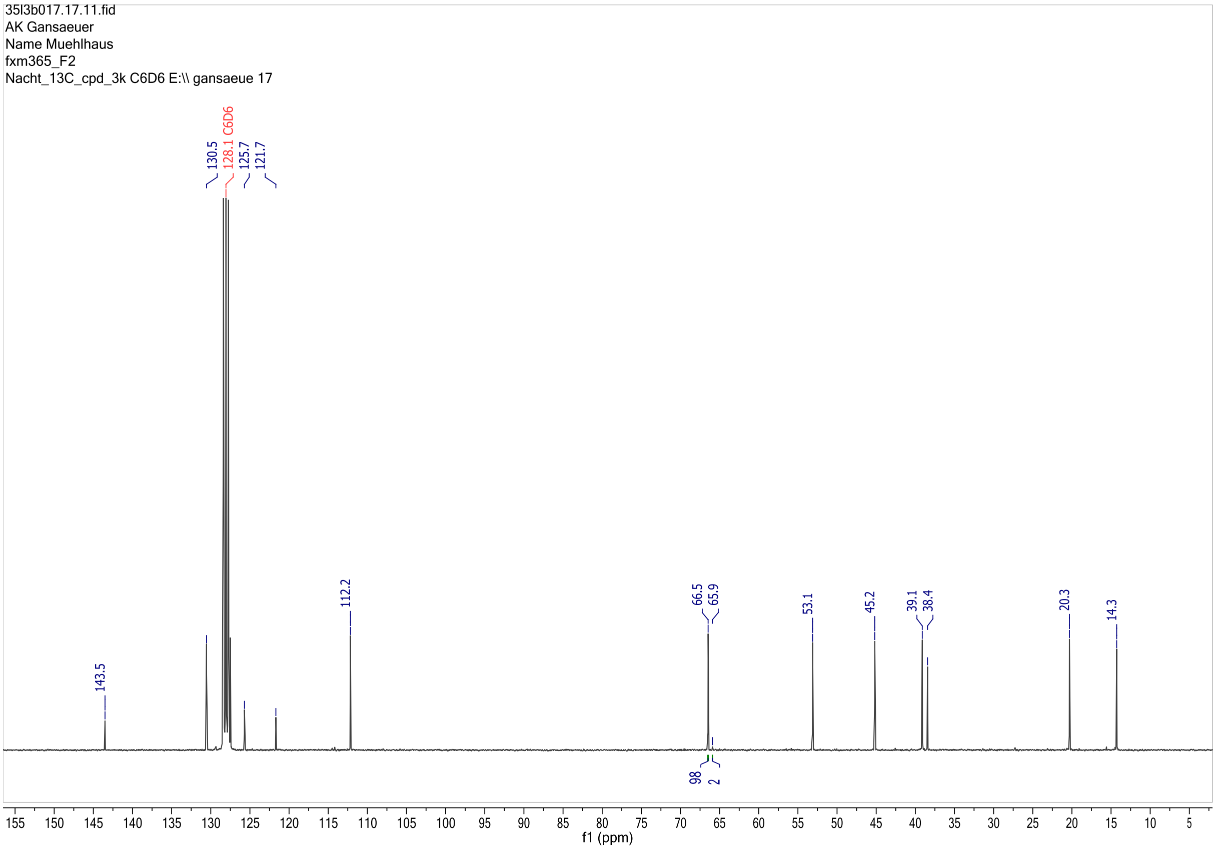
35l3b017.17.11.fid
AK Gansaeuer
Name Muehlhaus
fxm365_F2
Nacht_13C_cpd_3k C6D6 E:\\ gansaeue 17
143.5
130.5
128.1 C6D6
125.7
121.7
112.2
66.5
65.9
53.1
45.2
39.1
38.4
20.3
14.3
f1 (ppm)

4.3.11 Synthesis of Methyl-*(3S,4R)*-3-hydroxy-1-methyl-4-propyl-1,2,3,4-tetrahydroquinoline-6-carboxylate (2n).

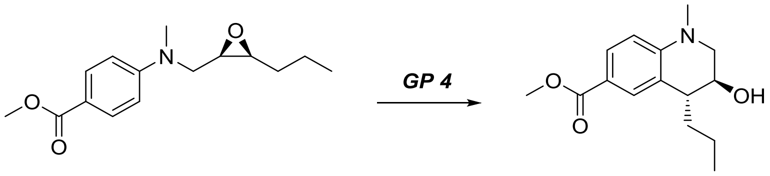

According to GP4: 132 mg of **1n** (1.00 eq., 0.50 mmol), 10 mg zinc (0.30 eq., 0.15 mmol) and 40 mg **cat-(OTs)$_2$** (0.10 eq., 0.05 mmol) are reacted in dry THF for 48 hours at room temperature. Regio- and diastereoisomeric ratio were determined by ^{13}C-NMR of the crude product. Flash chromatography (SiO_2, Eluent: CH : MTBE = 90 : 10) yielded 81 mg **2n** (d.r. = 98 : 2, 61%) as a viscous, yellow liquid.

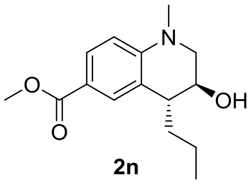

2n
$C_{15}H_{21}NO_3$
263,34 g/mol

R$_f$ = 0.4 (40% EE in CH);**^{1}H-NMR (500 MHz, C_6D_6, RT): δ [ppm]** = 0.75 (t, *J*= 7.1 Hz, 3H), 1.01-1.33 (m, 4H), 2.43 (s, 3H), 2.53-2.64 (m, 2H), 2.80 (ddd, *J*= 12.7 Hz, *J*= 2.7 Hz, *J*= 1.8 Hz, 1H), 3.01 (dd, *J*= 12.6 Hz, *J*= 2.7 Hz, 1H), 3.62 (s, 3H), 3.66 (q, *J*= 3.0 Hz, 1H), 6.32 (d, *J*= 8.7 Hz, 1H), 7.97 (d, *J*= 2.1 Hz, 1H), 8.08 (dd, *J*= 8.7 Hz, *J*= 2.1 Hz, 1H). **^{13}C-NMR (125 MHz, C_6D_6, RT): δ [ppm]** =14.3, 20.3, 38.2, 38.6, 45.0, 51.2, 53.1, 66.3, 109.6, 117.6, 122.6, 128.6, 130.3, 132.7, 148.4, 167.5. **IR ṽ [cm^{-1}]** =797, 1064, 1114, 1152, 1190, 1203, 1223, 1235, 1269, 1288, 1402, 1528, 1603, 1674, 2890, 3439. **HRMS (ESI):** *m/z* calculated for $[M+H]^+$: 264.1594 u; found: 264.1591 u. **$[\alpha]_D^{20}$** = -2.3° (*c* 1, $CHCl_3$).

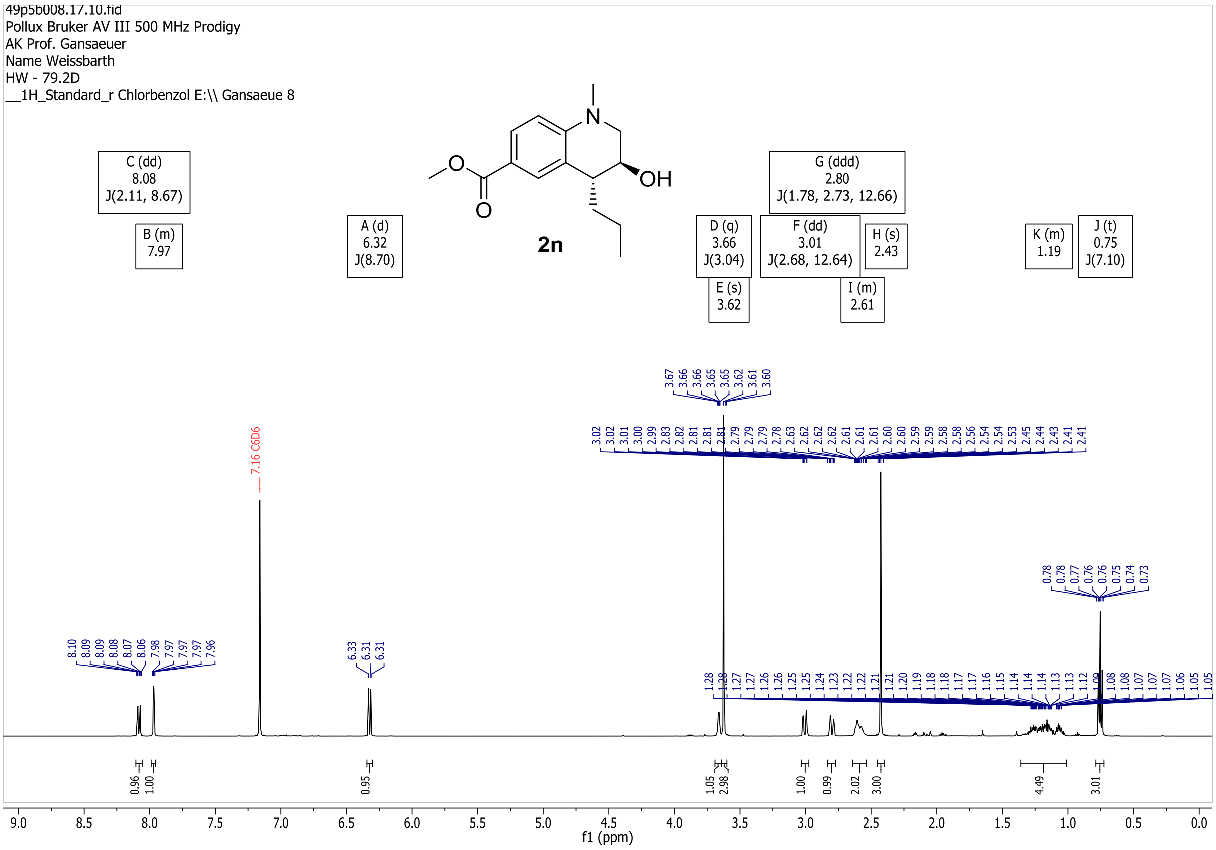
49p5b008.17.10.fid
Pollux Bruker AV III 500 MHz Prodigy
AK Prof. Gansaeuer
Name Weissbarth
HW - 79.2D
__1H_Standard_r Chlorbenzol E:\\ Gansaeue 8
2n
C (dd) 8.08 J(2.11, 8.67)
B (m) 7.97
A (d) 6.32 J(8.70)
G (ddd) 2.80 J(1.78, 2.73, 12.66)
D (q) 3.66 J(3.04)
F (dd) 3.01 J(2.68, 12.64)
H (s) 2.43
E (s) 3.62
I (m) 2.61
K (m) 1.19
J (t) 0.75 J(7.10)
7.16 C6D6
f1 (ppm)

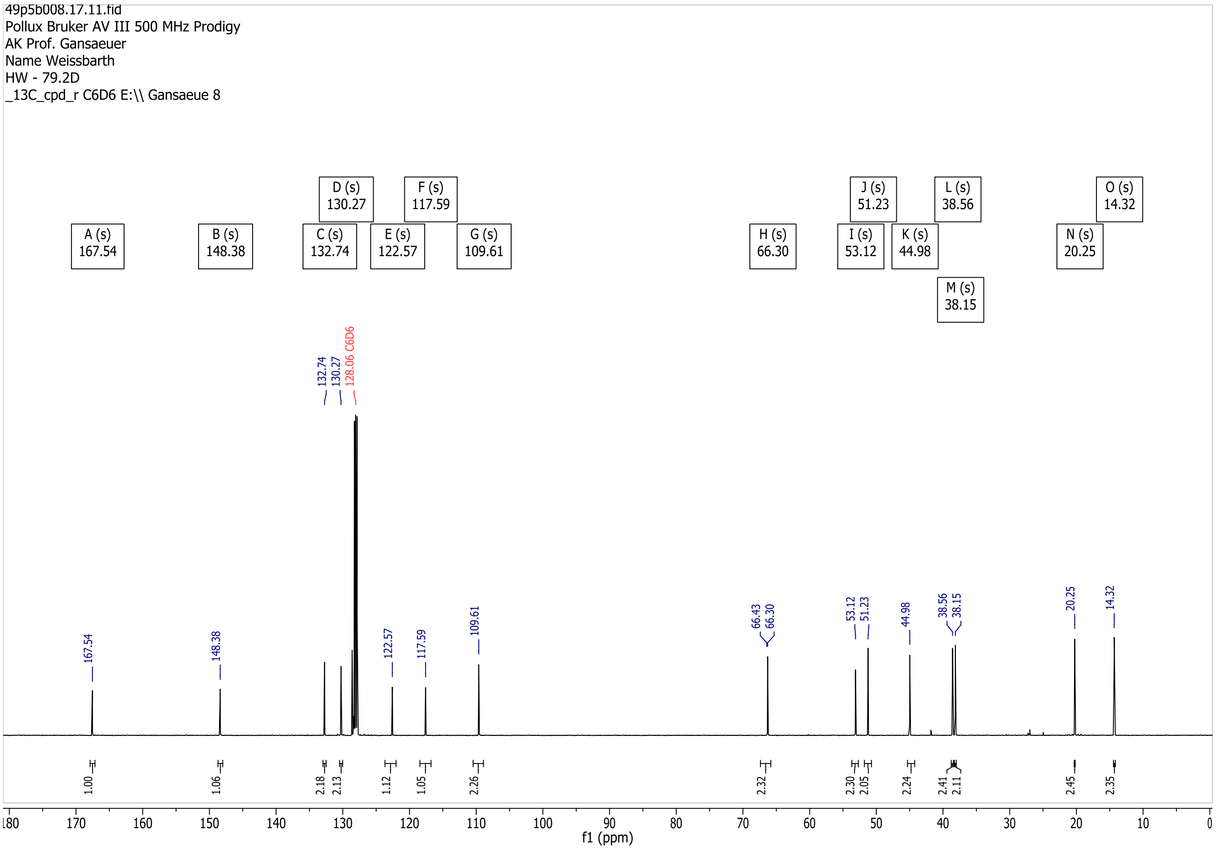
49p5b008.17.11.fid
Pollux Bruker AV III 500 MHz Prodigy
AK Prof. Gansaeuer
Name Weissbarth
HW - 79.2D
_13C_cpd_r C6D6 E:\\ Gansaeue 8
A (s) 167.54
B (s) 148.38
C (s) 132.74
D (s) 130.27
E (s) 122.57
F (s) 117.59
G (s) 109.61
H (s) 66.30
I (s) 53.12
J (s) 51.23
K (s) 44.98
L (s) 38.56
M (s) 38.15
N (s) 20.25
O (s) 14.32
128.06 C6D6
f1 (ppm)

4.3.12 Synthesis of (3*S*,4*R*)-6-iodo-1-methyl-4-propyl)-1,2,3,4-tetrahydroquinoline-3-ol (2o).

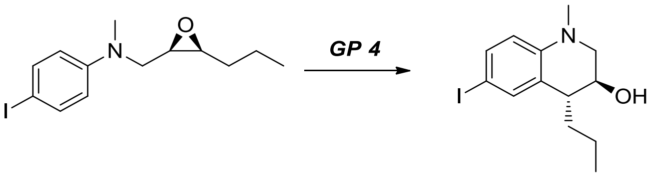

According to GP4: 156 mg of **1o** (1.00 eq., 0.50 mmol), 10 mg zinc (0.30 eq., 0.15 mmol) and 40 mg **cat-(OTs)$_2$** (0.10 eq., 0.05 mmol) are reacted in dry THF for 48 hours at room temperature. Regio- and diastereoisomeric ratio were determined by ^{13}C-NMR of the crude product. Flash chromatography (SiO_2, Eluent: CH : MTBE = 87 : 13) was performed rapidly and yielded 96 mg **2o** (d.r. = > 98 : < 2, 58%) as a viscous, yellow liquid.

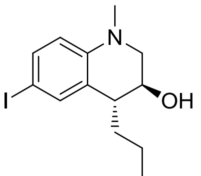

2o
$C_{13}H_{18}INO$
331.19 g/mol

R$_f$ = 0.45 (40% EE in CH);**^{1}H-NMR (300 MHz, C_6D_6, RT): δ [ppm]** = 0.71 (t, 3H, *J* = 7.0 Hz), 0.95-1.22 (m, 4H), 1.75-1.91 (s_{br}, 1H), 2.34 (s, 3H), 2.37-2.44 (m, 1H), 2.67 (ddd, 1H, *J* = 12.1 Hz, *J* = 3.1 Hz, *J* = 1.7 Hz), 2.87 (dd, 1H, *J* = 12.2 Hz, *J* = 2.5 Hz), 3.58 (q, 1H, *J* = 2.9 Hz), 6.04 (d, 1H, *J* = 8.6 Hz), 7.31 (d, 1H, *J* = 2.2 Hz), 7.37 (dd, 1H, *J* = 8.6 Hz, *J* = 2.2 Hz). **^{13}C-NMR (75 MHz, C_6D_6, RT): δ [ppm]** = 14.3, 20.3, 38.3, 39.1, 44.5, 53.0, 66.3, 77.6, 113.2, 126.7, 136.4, 139.0, 144.4. **IR ṽ [cm^{-1}] =** 524, 669, 793, 879, 1051, 1104, 1213, 1328, 1498, 2342, 2362. **HRMS (ESI):** *m/z* calculated for $NaC_{13}H_{18}INO^+$: 354.0325 u; found: 354.0332 u. **$[\alpha]_D^{20}$** = -13.4° (*c* 5.0, $CHCl_3$). d.r. = < 1 : > 99 (*cis* : *trans*, **2o** isolated).

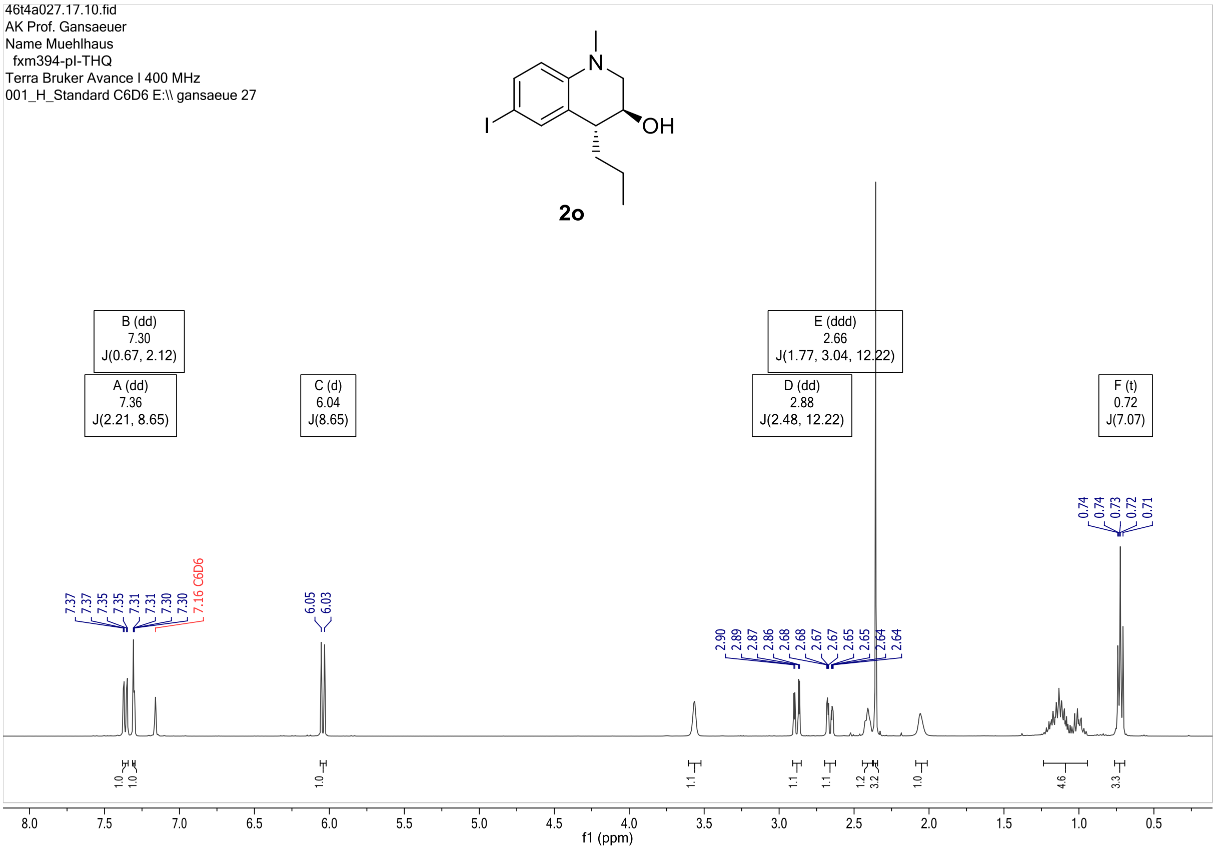
46t4a027.17.10.fid
AK Prof. Gansaeuer
Name Muehlhaus
fxm394-pI-THQ
Terra Bruker Avance I 400 MHz
001_H_Standard C6D6 E:\\ gansaeue 27
N
I
OH
2o
B (dd) 7.30 J(0.67, 2.12)
A (dd) 7.36 J(2.21, 8.65)
C (d) 6.04 J(8.65)
E (ddd) 2.66 J(1.77, 3.04, 12.22)
D (dd) 2.88 J(2.48, 12.22)
F (t) 0.72 J(7.07)
7.37 7.37 7.35 7.35 7.31 7.31 7.30 7.30 7.16 C6D6
6.05 6.03
2.90 2.89 2.87 2.86 2.68 2.68 2.67 2.67 2.65 2.65 2.64 2.64
0.74 0.74 0.73 0.72 0.71
1.0 1.0 1.0 1.1 1.1 1.1 1.2 3.2 1.0 4.6 3.3
f1 (ppm)

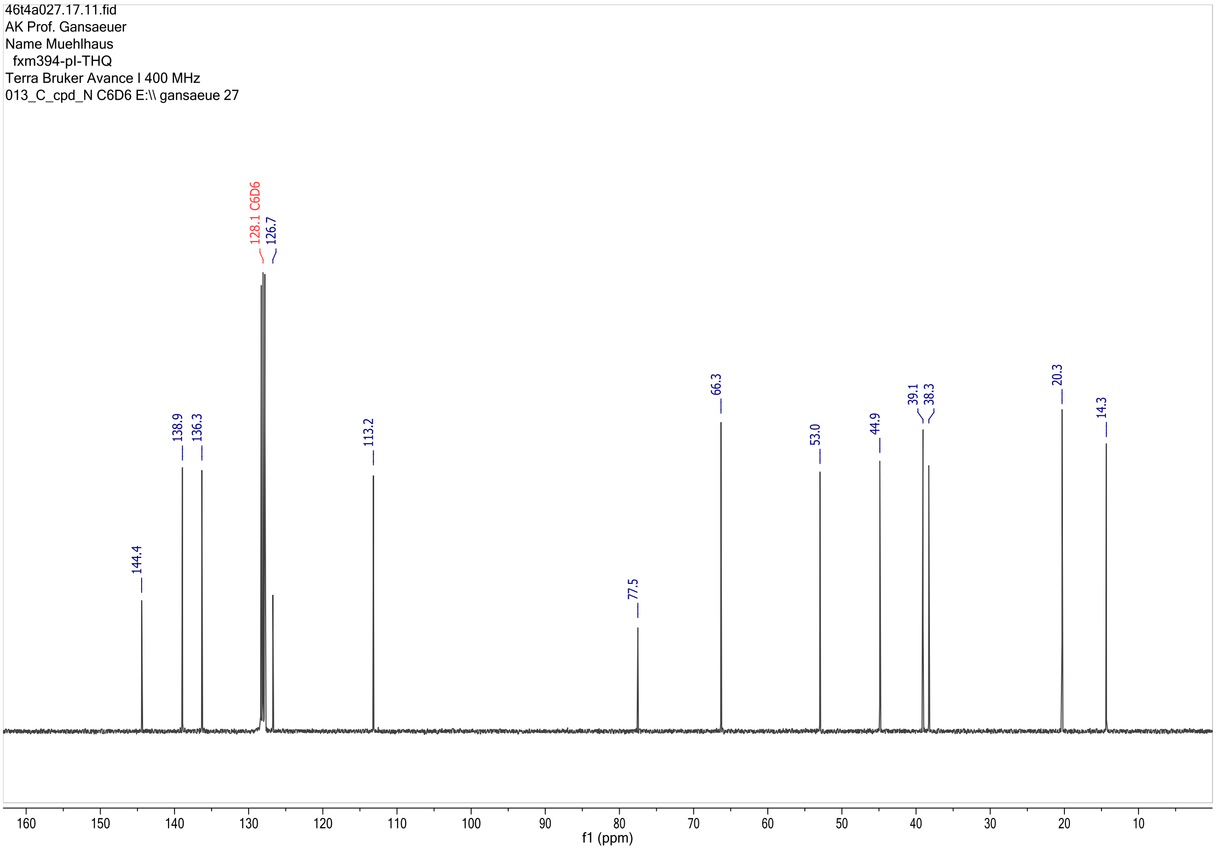
46t4a027.17.11.fid
AK Prof. Gansaeuer
Name Muehlhaus
fxm394-pI-THQ
Terra Bruker Avance I 400 MHz
013_C_cpd_N C6D6 E:\\ gansaeue 27
144.4
138.9
136.3
128.1 C6D6
126.7
113.2
77.5
66.3
53.0
44.9
39.1
38.3
20.3
14.3
f1 (ppm)

4.4. Synthesis of indolines 3 by REO-ArS$_R$

4.4.1 (*S*)-1-((*R*)-1-phenylindolin-3-yl)-butan-1-ol (3a).

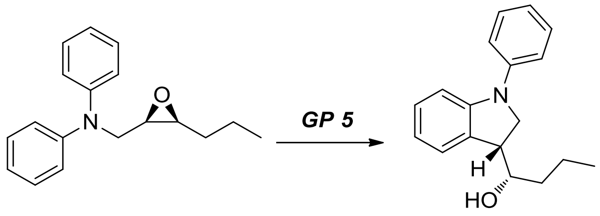

According to GP5: 134 mg of **1a** (1.00 eq., 0.50 mmol), 22 mg lutidinium chloride (0.30 eq., 0.15 mmol), 10 mg zinc (0.30 eq., 0.15 mmol) and 18 mg ***ent*-cat-Cl$_2$** (0.07 eq., 0.035 mmol) are reacted in dry THF for 48 hours at room temperature. Regio- and diastereoisomeric ratio were determined by ^{13}C-NMR of the crude product. Flash chromatography (SiO_2, Eluent: Ch : Mtbe = 87 : 13) yielded 96 mg **3a** (d.r. = > 98 : < 2, 72%) as colorless, crystalline solid (m.p. = 109 °C).

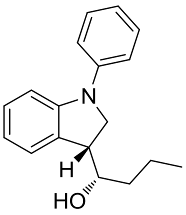

3a
$C_{18}H_{21}NO$
267.37 g/mol

R_f = 0.64 (40% EE in CH), **^{1}H-NMR (300 MHz, C_6D_6, RT): δ [ppm] =** 0.84 (t, 3H, *J* = 7.2 Hz), 1.13-1.38 (m, 4H), 1.38-1.45 (m, 1H), 3.00 (dt, 1H, *J* = 10.1 Hz, *J* = 5.2 Hz), 3.57 (t, 1H, *J* = 9.5 Hz), 3.60-3.64 (m, 1H), 3.85 (dd, 1H, *J* = 9.4 Hz, *J* = 6.1 Hz), 6.70-6.76 (m, 1H), 6.86-6.91 (m, 1H), 7.02 (t, 1H, *J* = 7.2 Hz), 7.11-7.17 (m, 3H), 7.20 (dd, 1H, *J* = 8.7 Hz, *J* = 7.0 Hz). **^{13}C-NMR (75 MHz, C_6D_6, RT): δ [ppm] =** 14.3, 19.5, 36.6, 46.7, 53.2, 71.9, 108.9, 118.1, 119.1, 121.3, 125.2, 129.5, 131.8, 144.5, 148.2. **IR ṽ [cm^{-1}] =** 491, 497, 554, 616, 691, 735, 742, 961, 1014, 1065, 1079, 1120, 1247, 1288, 1333, 1383, 1460, 1882, 1501, 1589. **HRMS (ESI):** *m/z* calculated for $C_{18}H_{22}NO^+$: 268.1696 u, found 267.1700 u. **$[\alpha]_D^{20}$=** – 69.6 (*c* 0.25, $CHCl_3$). determination of e.r. (**3a**, isolated) by HPLC: *DAICEL Chiralpak IC-U* 01; *n*-Hexane/iPrOH (95 : 5); flowrate 0.43 mL/min; t_R = 2.5 min (major), t_R = 2.8 min (minor); e.r. = > 99 : < 1.

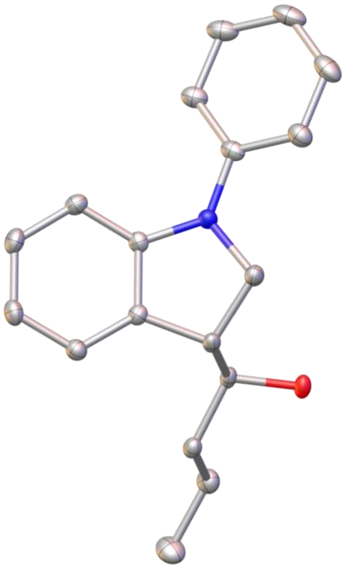

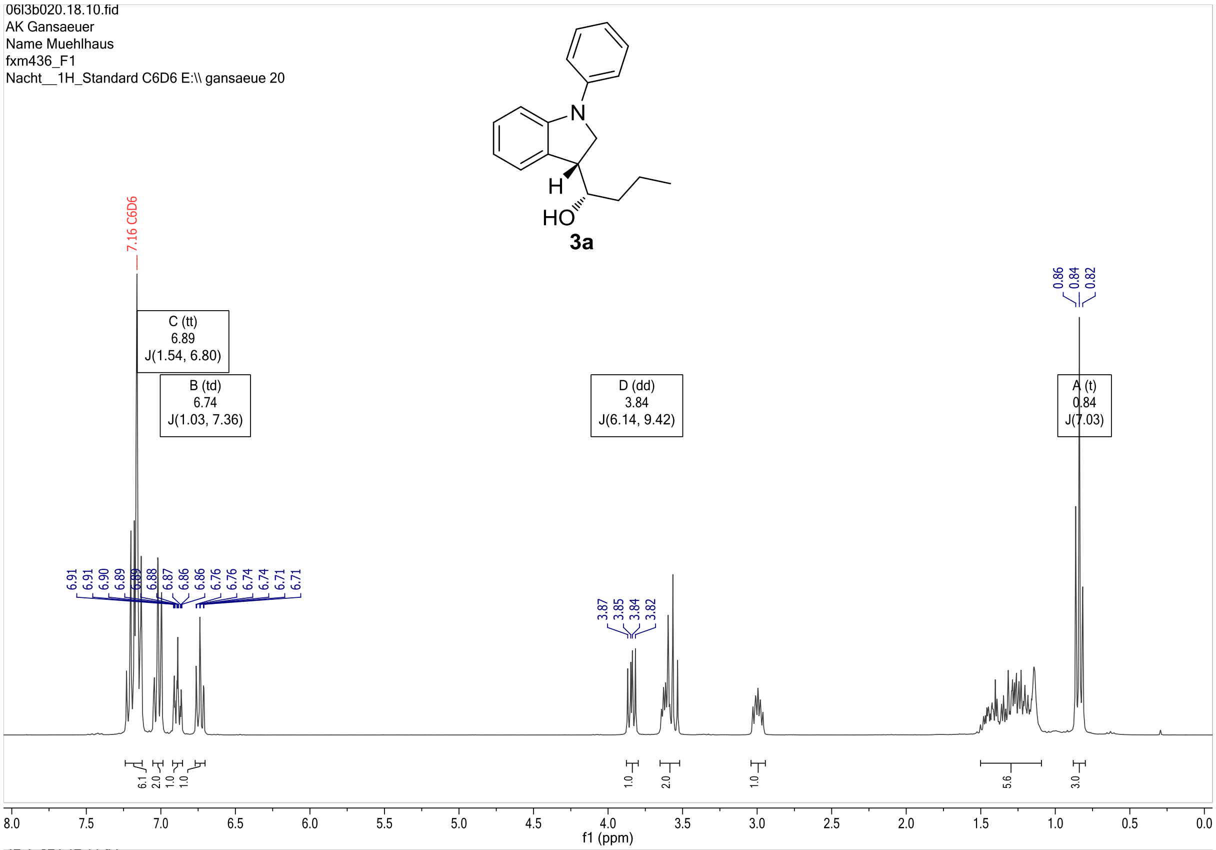
06l3b020.18.10.fid
AK Gansaeuer
Name Muehlhaus
fxm436_F1
Nacht__1H_Standard C6D6 E:\\ gansaeue 20
3a
N
H
HO
7.16 C6D6
C (tt)
6.89
J(1.54, 6.80)
B (td)
6.74
J(1.03, 7.36)
D (dd)
3.84
J(6.14, 9.42)
A (t)
0.84
J(7.03)
0.86
0.84
0.82
6.91 6.91 6.90 6.89 6.89 6.88 6.87 6.86 6.86 6.76 6.76 6.74 6.74 6.71 6.71
3.87 3.85 3.84 3.82
6.1 2.0 1.0 1.0 1.0 2.0 1.0 5.6 3.0
f1 (ppm)

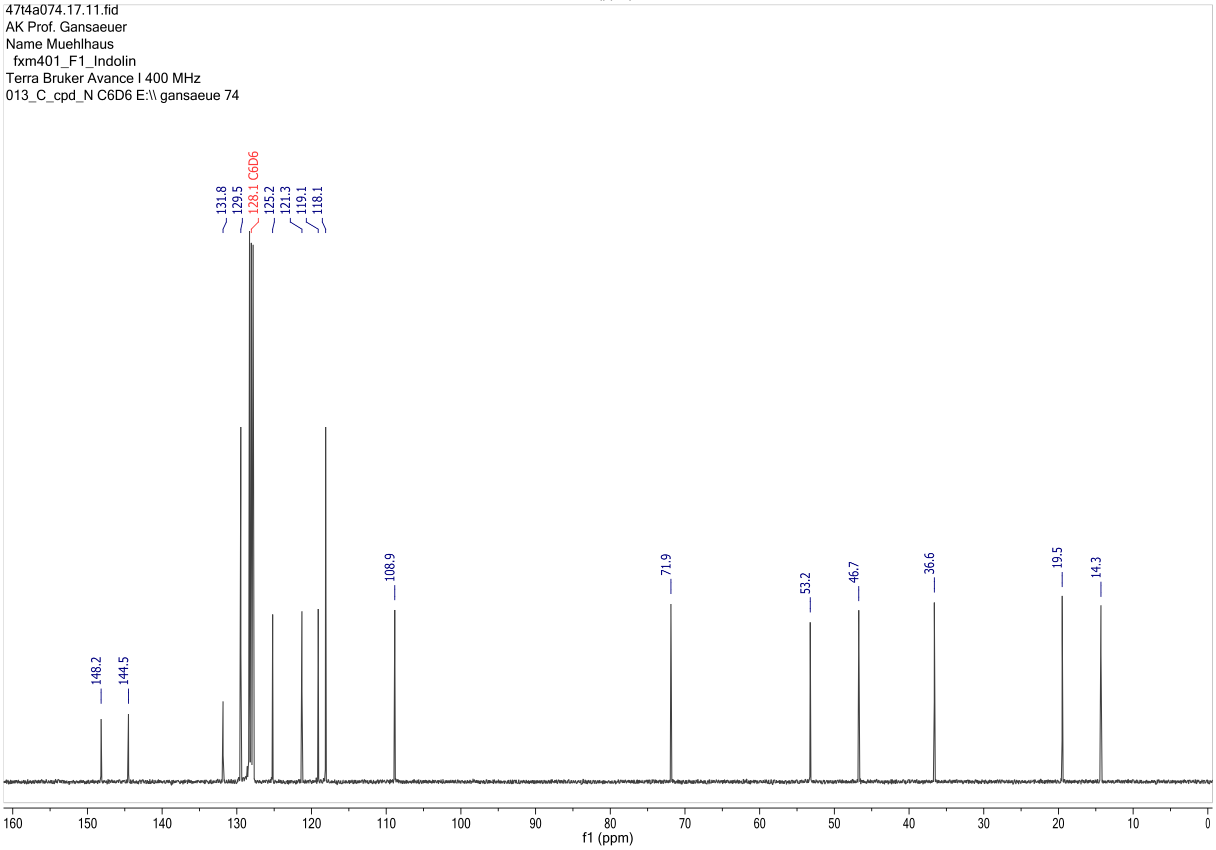
47t4a074.17.11.fid
AK Prof. Gansaeuer
Name Muehlhaus
fxm401_F1_Indolin
Terra Bruker Avance I 400 MHz
013_C_cpd_N C6D6 E:\\ gansaeue 74
131.8
129.5
128.1 C6D6
125.2
121.3
119.1
118.1
148.2
144.5
108.9
71.9
53.2
46.7
36.6
19.5
14.3
f1 (ppm)

4.4.2 Synthesis of (*S*)-1-((*R*)-1-phenylindolin-3-yl)-hexan-1-ol (3b).

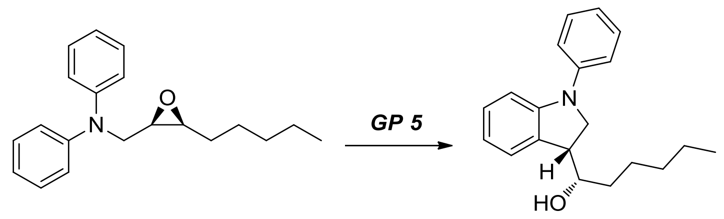

According to GP5: 148 mg of **1b** (1.00 eq., 0.50 mmol), 22 mg lutidinium chloride (0.30 eq., 0.15 mmol), 10 mg zinc (0.30 eq., 0.15 mmol) and 18 mg ***ent*-cat-Cl$_2$** (0.07 eq., 0.035 mmol) are reacted in dry THF for 48 hours at room temperature. Regio- and diastereoisomeric ratio were determined by ^{13}C-NMR of the crude product. Flash chromatography (SiO_2, Eluent: Ch : Mtbe = 87 : 13) yielded 111 mg **3b** (d.r. = > 98 : < 2, 75%) as colorless, crystalline solid (m.p. = 115 °C).

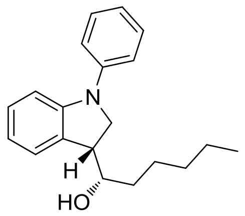

3b
$C_{20}H_{25}NO$
295.42 g/mol

R$_f$ = 0.7 (40% EE in CH), **^{1}H-NMR (300 MHz, C$_6$D$_6$, RT): δ [ppm] =** 0.90 (t, 3H, *J* = 6.9 Hz), 1.12 (d, 1H, *J* = 3.9 Hz), 1.14-1.50 (m, 8H), 2.98 (m, 1H), 3.55-3.08 (m, 2H), 3.87 (dd, 1H, *J* = 9.4 Hz, *J* = 6.1 Hz), 6.70-6.76 (m, 1H), 6.86-6.91 (m, 1H), 7.02 (t, 1H, *J* = 7.2 Hz), 7.11-7.17 (m, 3H), 7.20 (dd, 1H, *J* = 8.7 Hz, *J* = 7.0 Hz).**^{13}C-NMR (75 MHz, C$_6$D$_6$, RT): δ [ppm] =** 14.4, 23.1, 26.1, 32.3, 33.6, 46.8, 53.3, 72.2, 108.9, 118.2, 119.2, 121.3, 125.2, 129.5, 131.9, 144.6, 148.2. **IR ṽ [cm^{-1}] =** 618, 694, 728, 736, 744, 752, 1332, 1382, 1460, 1483, 1501, 1591, 2360, 2913. **HRMS (ESI):** *m/z* calculated for $C_{20}H_{26}NO^+$: 296.2009 u, found: 296.2012 u. **[α]$_D^{20}$=** – 68.0 (*c* 0.25, $CHCl_3$).

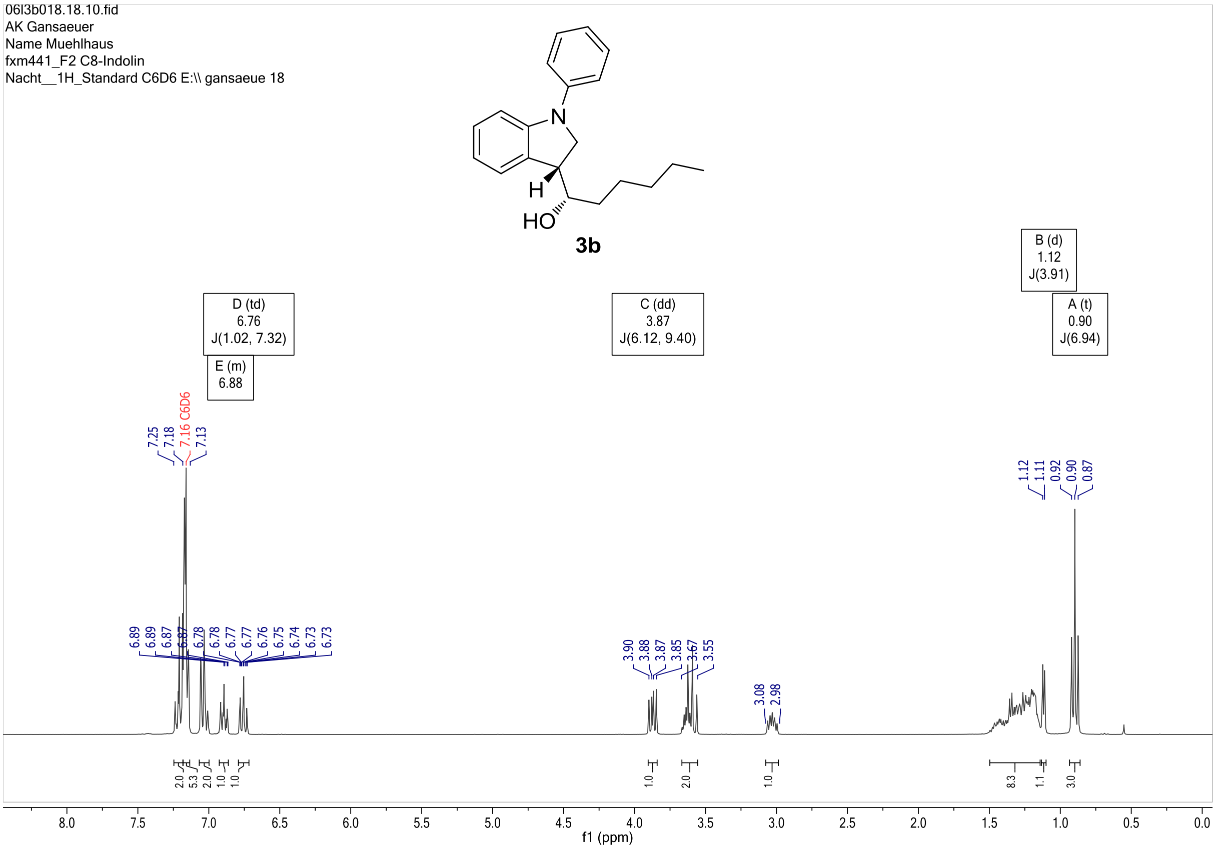
06l3b018.18.10.fid
AK Gansaeuer
Name Muehlhaus
fxm441_F2 C8-Indolin
Nacht__1H_Standard C6D6 E:\\ gansaeue 18
3b
D (td) 6.76 J(1.02, 7.32)
E (m) 6.88
C (dd) 3.87 J(6.12, 9.40)
B (d) 1.12 J(3.91)
A (t) 0.90 J(6.94)
7.16 C6D6
f1 (ppm)

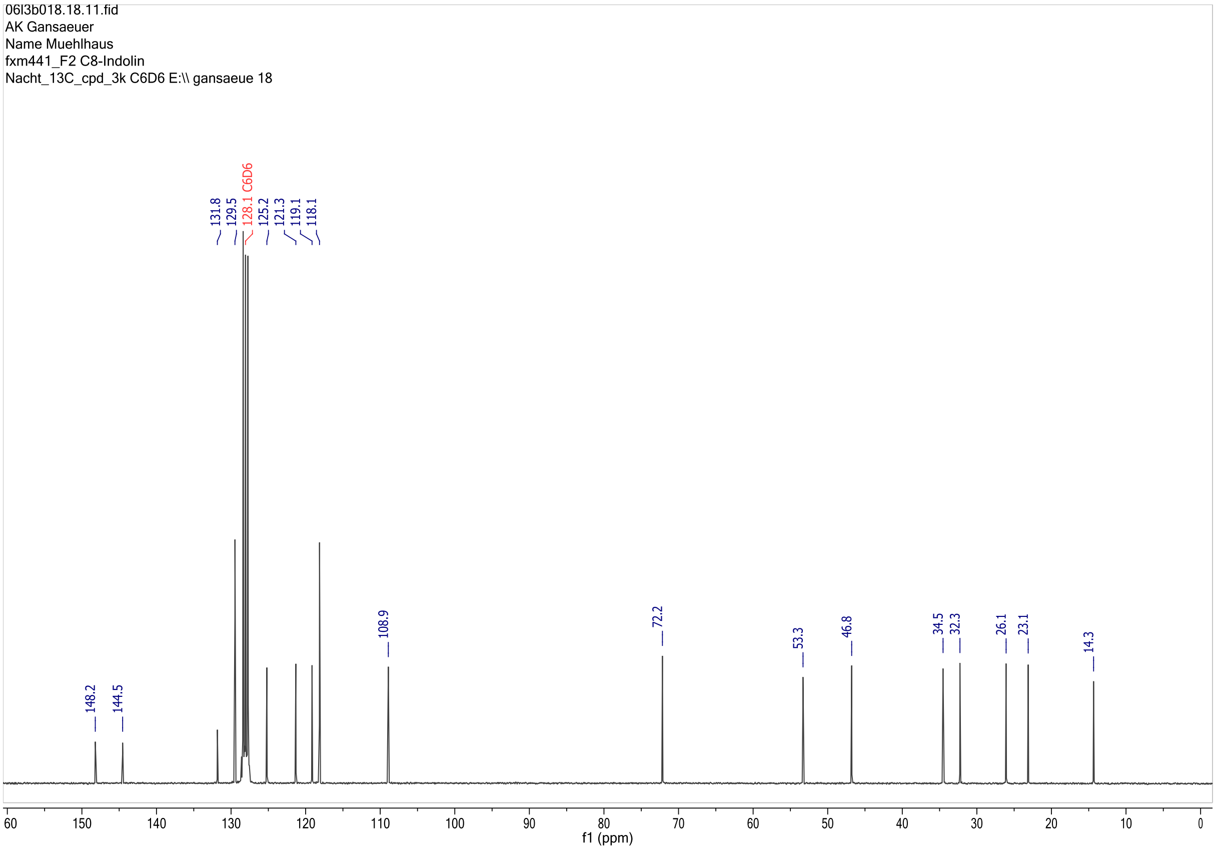
06l3b018.18.11.fid
AK Gansaeuer
Name Muehlhaus
fxm441_F2 C8-Indolin
Nacht_13C_cpd_3k C6D6 E:\\ gansaeue 18
148.2
144.5
131.8
129.5
128.1 C6D6
125.2
121.3
119.1
118.1
108.9
72.2
53.3
46.8
34.5
32.3
26.1
23.1
14.3
f1 (ppm)

4.4.3 Synthesis of (*S*)-3-methyl-1-((*R*)-1-phenylindolin-3-yl)-butan-1-ol (3c).

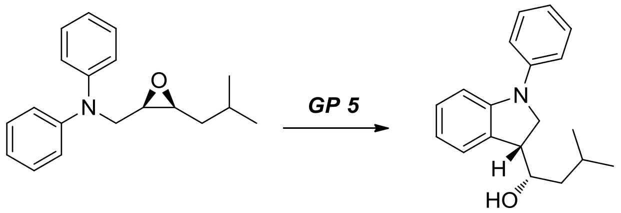

According to GP5: 141 mg of **1c** (1.00 eq., 0.50 mmol), 22 mg lutidinium chloride (0.30 eq., 0.15 mmol), 10 mg zinc (0.30 eq., 0.15 mmol) and 18 mg ***ent*-cat-Cl_2** (0.07 eq., 0.035 mmol) are reacted in dry THF for 48 hours at room temperature. Regio- and diastereoisomeric ratio were determined by ^{13}C-NMR of the crude product. Flash chromatography (SiO_2, Eluent: Ch : Mtbe = 87 : 13) yielded 85 mg **3c** (d.r. = > 98 : < 2, 60%) as colorless, crystalline solid (m.p. = 66 °C).

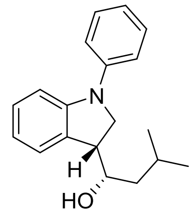

3c
$C_{19}H_{23}NO$
281.39 g/mol

R_f = 0.2 (30% Et_2O in CH), **^{1}H-NMR (300 MHz, C_6D_6, RT): δ [ppm] =** 0.82 (d, 3H, *J* = 6.6 Hz), 0.89 (d, 3H, *J* = 6.6 Hz), 1.04-1.13 (m, 1H), 1.31-1.40 (m, 1H), 1.75 (sept, 1H, *J* = 6.5 Hz), 3.00 (ddd, 1H, *J* = 10.1 Hz, *J* = 6.3 Hz, *J* = 3.9 Hz), 3.56 (t, 1H, *J* = 9.5 Hz), 3.80 (dd, 1H, *J* = 8.5 Hz, *J* = 5.1 Hz), 6.70 (dd, 1H. *J* = 8.7 Hz, *J* = 5.7 Hz), 6.88 (td, 1H, *J* = 7.0 Hz, *J* = 1.6 Hz), 7.11-7.22 (m, 5H). **^{13}C-NMR (75 MHz, C_6D_6, RT): δ [ppm] =** 22.0, 23.9, 24.9, 43.5, 47.1, 53.0, 70.2, 108.9, 118.2, 119.1, 121.3, 125.1, 128.4, 129.5, 131.8, 144.5, 148.2. **IR ṽ [cm^{-1}] =** 496, 531, 583, 692, 743, 755, 967, 1020, 1168, 1081, 1139, 1206, 1247, 1289, 1312, 1334, 1382, 1457, 1499, 1587. **HRMS (ESI):** *m/z* calculated for $C_{19}H_{24}NO^+$: 282.1852 u, found 282.1848 u. **$[\alpha]_D^{20}$=** – 62.0 (*c* 1.0, $CHCl_3$).

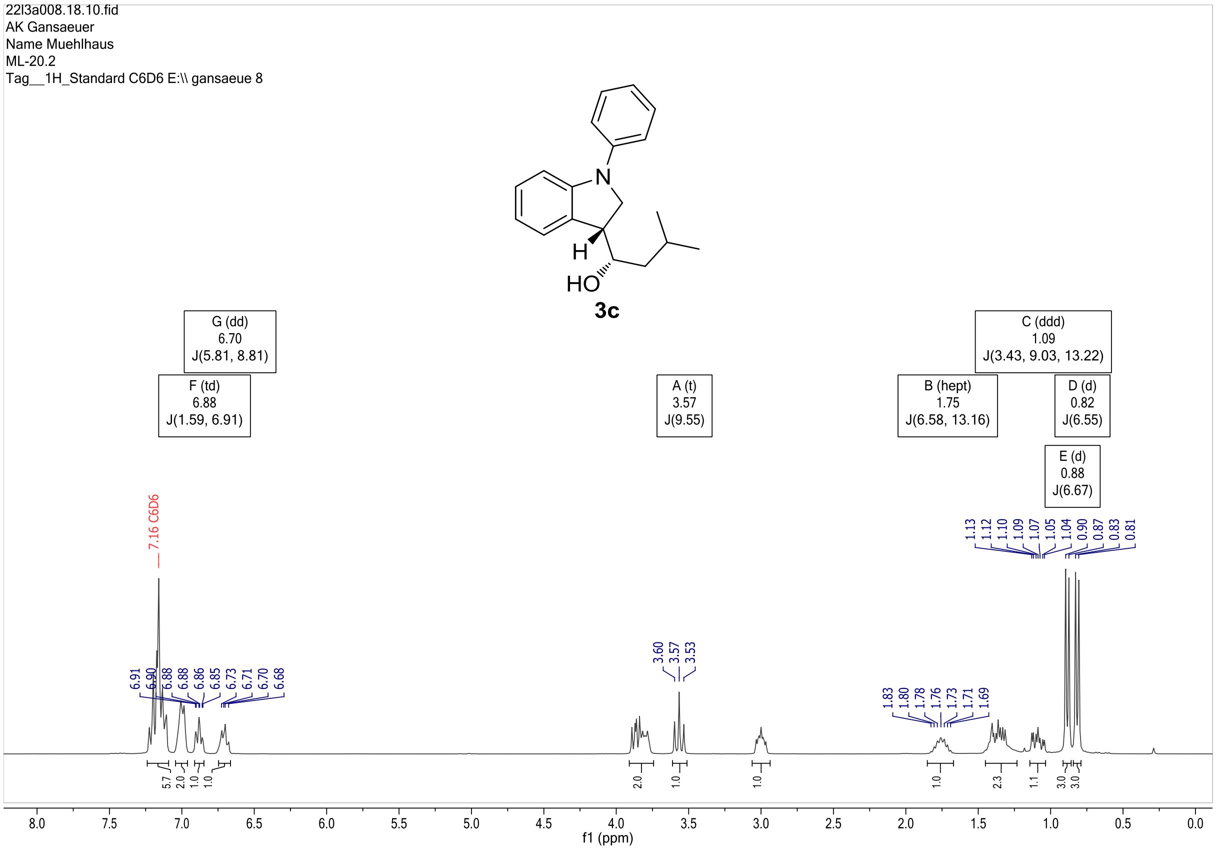
22l3a008.18.10.fid
AK Gansaeuer
Name Muehlhaus
ML-20.2
Tag__1H_Standard C6D6 E:\\ gansaeue 8
N
H
HO
3c
G (dd) 6.70 J(5.81, 8.81)
F (td) 6.88 J(1.59, 6.91)
A (t) 3.57 J(9.55)
C (ddd) 1.09 J(3.43, 9.03, 13.22)
B (hept) 1.75 J(6.58, 13.16)
D (d) 0.82 J(6.55)
E (d) 0.88 J(6.67)
7.16 C6D6
6.91 6.90 6.88 6.88 6.86 6.85 6.73 6.71 6.70 6.68
3.60 3.57 3.53
1.83 1.80 1.78 1.76 1.73 1.71 1.69
1.13 1.12 1.10 1.09 1.07 1.05 1.04 0.90 0.87 0.83 0.81
5.7 2.0 1.0 1.0 2.0 1.0 1.0 1.0 2.3 1.1 3.0 3.0
f1 (ppm)

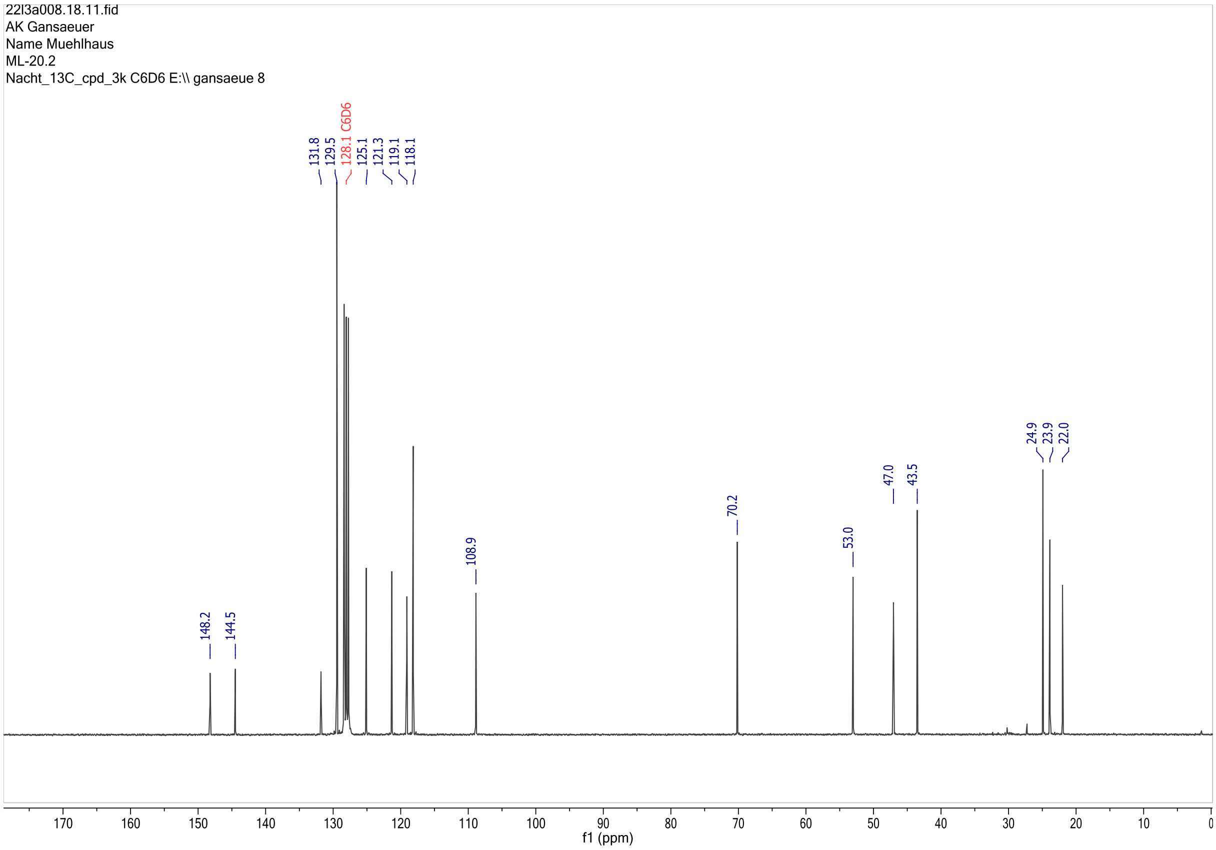
22l3a008.18.11.fid
AK Gansaeuer
Name Muehlhaus
ML-20.2
Nacht_13C_cpd_3k C6D6 E:\\ gansaeue 8
131.8 129.5 128.1 C6D6 125.1 121.3 119.1 118.1
148.2 144.5 108.9 70.2 53.0 47.0 43.5 24.9 23.9 22.0
f1 (ppm)

4.4.4 Synthesis of (*S*)-1-((*R*)-1-cyclohexylindolin-3-yl)-butan-1-ol (3d).

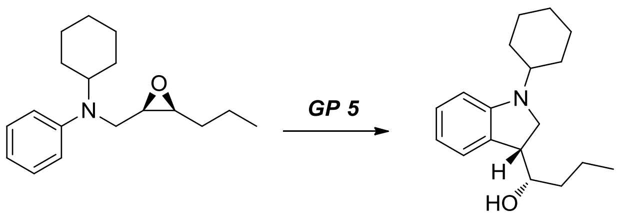

According to GP5: 144 mg of **1d** (1.00 eq., 0.50 mmol), 22 mg lutidinium chloride (0.30 eq., 0.15 mmol), 10 mg zinc (0.30 eq., 0.15 mmol) and 18 mg ***ent*-cat-Cl_2** (0.07 eq., 0.035 mmol) are reacted in dry THF for 48 hours at room temperature. Regio- and diastereoisomeric ratio were determined by ^{13}C-NMR of the crude product. Flash chromatography (SiO_2, Eluent: Ch : Mtbe = 87 : 13) yielded 81 mg **3d** (d.r. = > 98 : < 2, 56%) as colorless, crystalline solid.

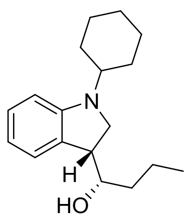

3d
$C_{18}H_{27}NO$
273.41 g/mol

R_f = 0.50 (20% Et_2O in CH); **^{1}H-NMR (300 MHz, C_6D_6, RT): δ [ppm] =** 0.89 (t, 3H, *J* = 7.1 Hz), 1.05-1.57 (m, 10H), 1.59-1.81 (m, 4H), 3.07 (ddd, 1H, *J* = 9.5 Hz, *J* = 7.1 Hz, *J* = 3.8 Hz), 3.16-3.28 (m, 2H), 3.45 (dd, 1H, *J* = 8.6 Hz, *J* = 7.1 Hz), 3.79 (dt, 1H, *J* = 8.2 Hz, *J* = 3.8 Hz), 3.85 (dd, 1H, *J* = 9.4 Hz, *J* = 6.1 Hz), 6.37 (d, 1H, *J* = 7.9 Hz), 6.70 (ddd, 1H, *J* = 7.3 Hz, *J* = 7.3 Hz, *J* = 1.0 Hz), 7.00 (dt, 1H, *J* = 7.4 Hz, *J* = 1.2 Hz), 7.10-7.14 (m, 1H). **^{13}C-NMR (75 MHz, C_6D_6, RT): δ [ppm] =** 14.4, 19.7, 26.2, 26.3, 28.3, 29.5, 36.9, 47.1, 47.5, 54.8, 71.9, 107.4, 117.0, 124.5, 128.6, 130.3, 152.5. **IR ṽ [cm^{-1}] =** 456, 676, 734, 1008, 1028, 1072, 1160, 1242, 1272, 1393, 1457, 1489, 1603, 2854, 2927. **HRMS (ESI):** *m/z* calculated for $C_{18}H_{28}NO^+$: 274.2165 u, found: 274.2168 u. **$[\alpha]_D^{20}$=** – 51.8 (*c* 0.25, $CHCl_3$).

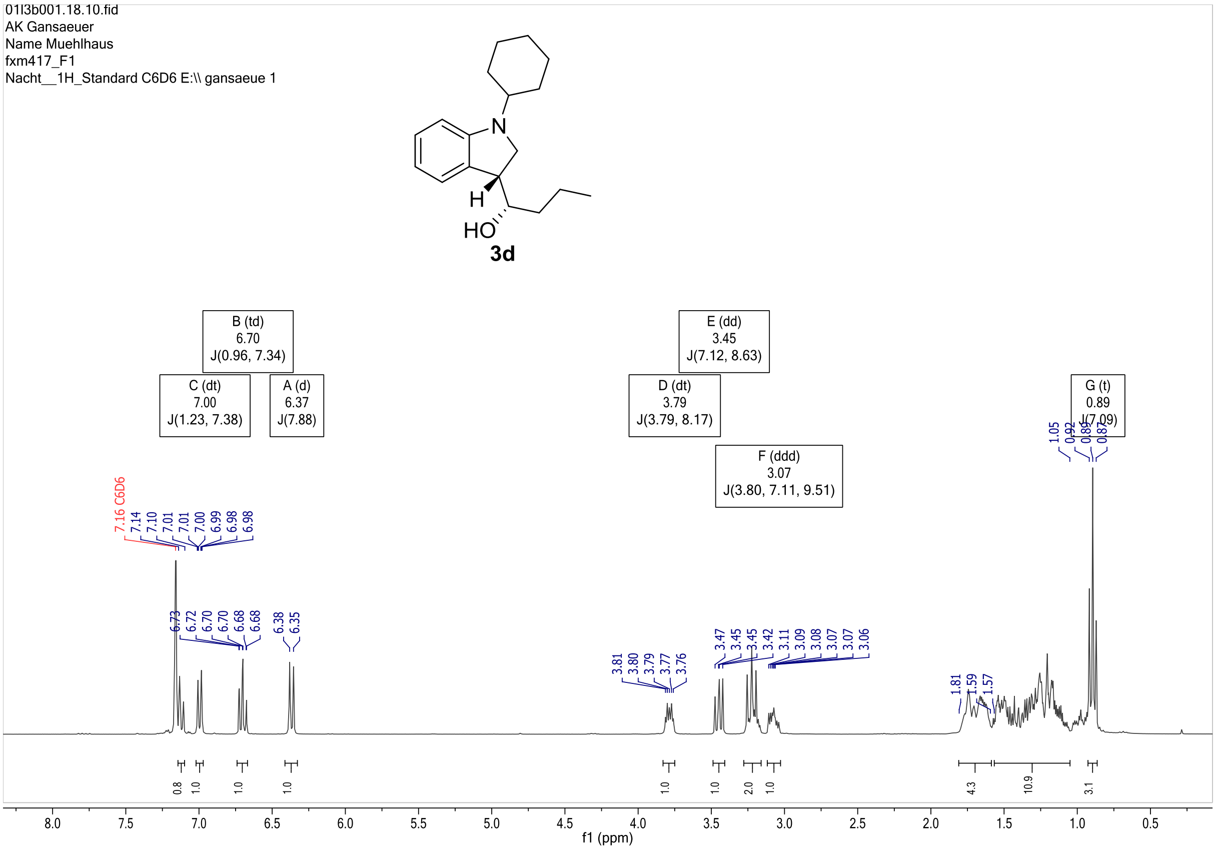
01l3b001.18.10.fid
AK Gansaeuer
Name Muehlhaus
fxm417_F1
Nacht__1H_Standard C6D6 E:\\ gansaeue 1
N
H
HO
3d
B (td) 6.70 J(0.96, 7.34)
C (dt) 7.00 J(1.23, 7.38)
A (d) 6.37 J(7.88)
E (dd) 3.45 J(7.12, 8.63)
D (dt) 3.79 J(3.79, 8.17)
F (ddd) 3.07 J(3.80, 7.11, 9.51)
G (t) 0.89 J(7.09)
f1 (ppm)

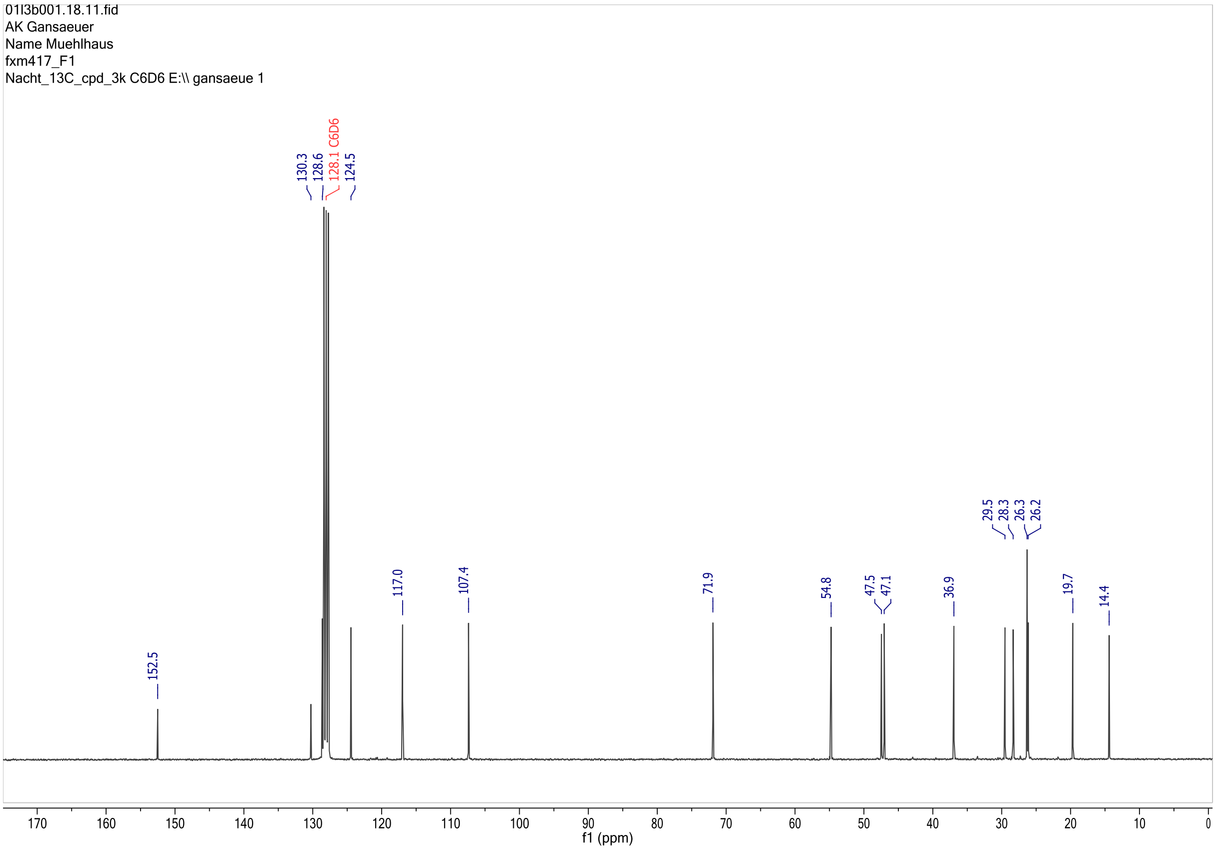
01l3b001.18.11.fid
AK Gansaeuer
Name Muehlhaus
fxm417_F1
Nacht_13C_cpd_3k C6D6 E:\\ gansaeue 1
152.5
130.3
128.6
128.1 C6D6
124.5
117.0
107.4
71.9
54.8
47.5
47.1
36.9
29.5
28.3
26.3
26.2
19.7
14.4
f1 (ppm)

4.4.5 Synthesis of (*S*)-1-((*R*)-1-isopropylindolin-3-yl)-butan-1-ol (3e).

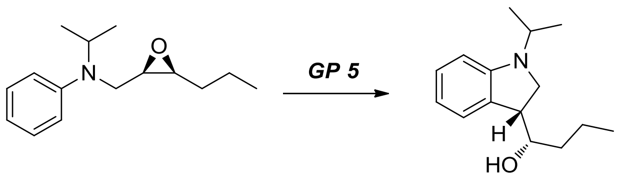

According to GP5: 117 mg of **1e** (1.00 eq., 0.50 mmol), 22 mg lutidinium chloride (0.30 eq., 0.15 mmol), 10 mg zinc (0.30 eq., 0.15 mmol) and 18 mg ***ent*-cat-Cl_2** (0.07 eq., 0.035 mmol) are reacted in dry THF for 48 hours at room temperature. Regio- and diastereoisomeric ratio were determined by ^{13}C-NMR of the crude product. Flash chromatography (SiO_2, Eluent: Ch : Mtbe = 87 : 13) yielded 63 mg **3e** (d.r. = > 98 : < 2, 54%) as colorless, crystalline solid.

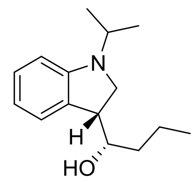

3e
$C_{15}H_{23}NO$
233.35 g/mol

R_f = 0.33 (20% Et_2O in CH), **^{1}H-NMR (300 MHz, C_6D_6, RT): δ [ppm] =** 0.88 (t, 3H, *J* = 7.1 Hz), 0.96 (dd, 6H, *J* = 6.7 Hz, *J* = 1.9 Hz), 1.17-1.61 (m, 4H), 3.05 (ddd, 1H, *J* = 9.3 Hz, *J* = 7.0 Hz, *J* = 3.8 Hz), 3.15 (dd, 1H, *J* = 9.5 Hz, *J* = 8.4 Hz), 3.39 (dd, 1H, *J* = 8.4 Hz, *J* = 6.9 Hz), 3.57 (hept, 1H, *J* = 6.7 Hz), 3.77 (dt, 1H, *J* = 7.9 Hz, *J* = 3.7 Hz), 6.34 (d, 1H, *J* = 7.9 Hz), 6.71 (ddd, 1H, *J* = 7.4 Hz, *J* = 7.4 Hz, *J* = 1.0 Hz), 6.98 (ddd, 1H, *J* = 7.3 Hz, *J* = 1.2 Hz), 7.12 (dd, 1H, *J* = 7.8 Hz, *J* = 1.9 Hz). **^{13}C-NMR (75 MHz, C_6D_6, RT): δ [ppm] =** 14.4, 17.6, 18.6, 19.7, 36.9, 46.0, 46.2, 46.9, 71.9, 107.6, 117.2, 124.4, 128.6, 130.4, 152.6. **IR ṽ [cm^{-1}]** = 427, 453, 525, 741, 995, 1026, 1070, 1120, 1195, 1240, 1266, 1363, 1392, 1458, 1488, 1604, 2870, 2929, 2960. **HRMS (ESI):** *m/z* calculated for $C_{15}H_{24}NO^+$: 234.1852 u, found: 234.1850 u. **$[\alpha]_D^{20}$**= – 36.0 (*c* 0. 5, $CHCl_3$).

17l3d007.18.10.fid
AK Gansaeuer
Name Muehlhaus
fxm461_F1 N-iPr-Indolin
Nacht__1H_Standard C6D6 E:\\ gansaeue 7

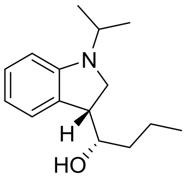

3e

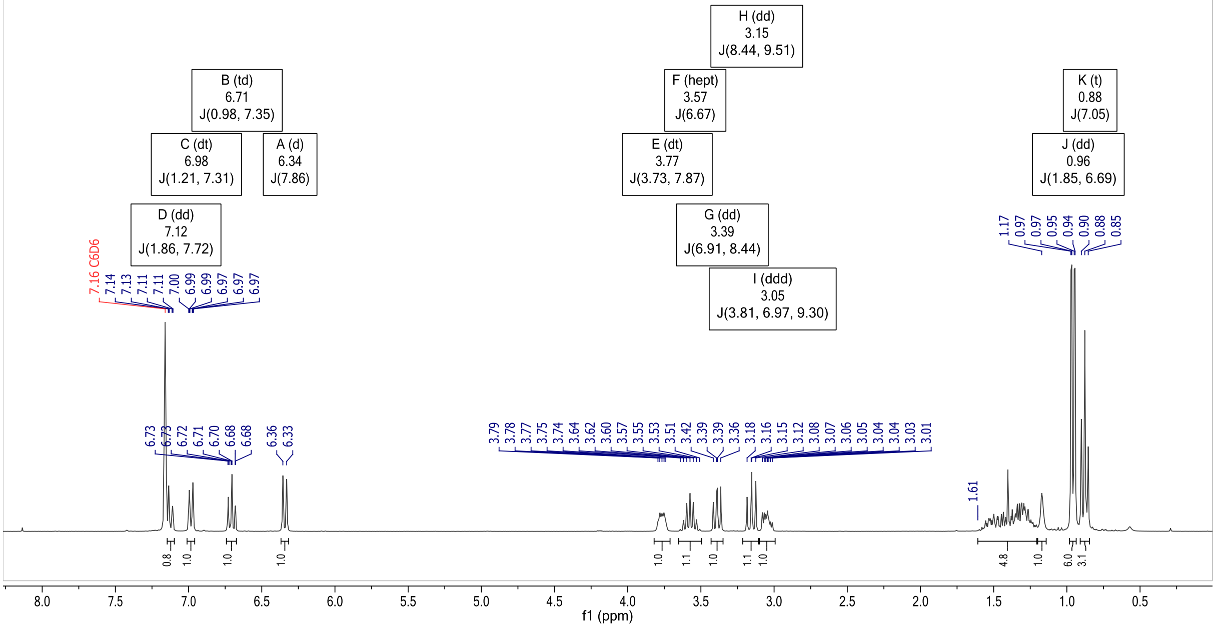

17l3d007.18.11.fid
AK Gansaeuer
Name Muehlhaus
fxm461_F1 N-iPr-Indolin
Nacht_13C_cpd_3k C6D6 E:\\ gansaeue 7

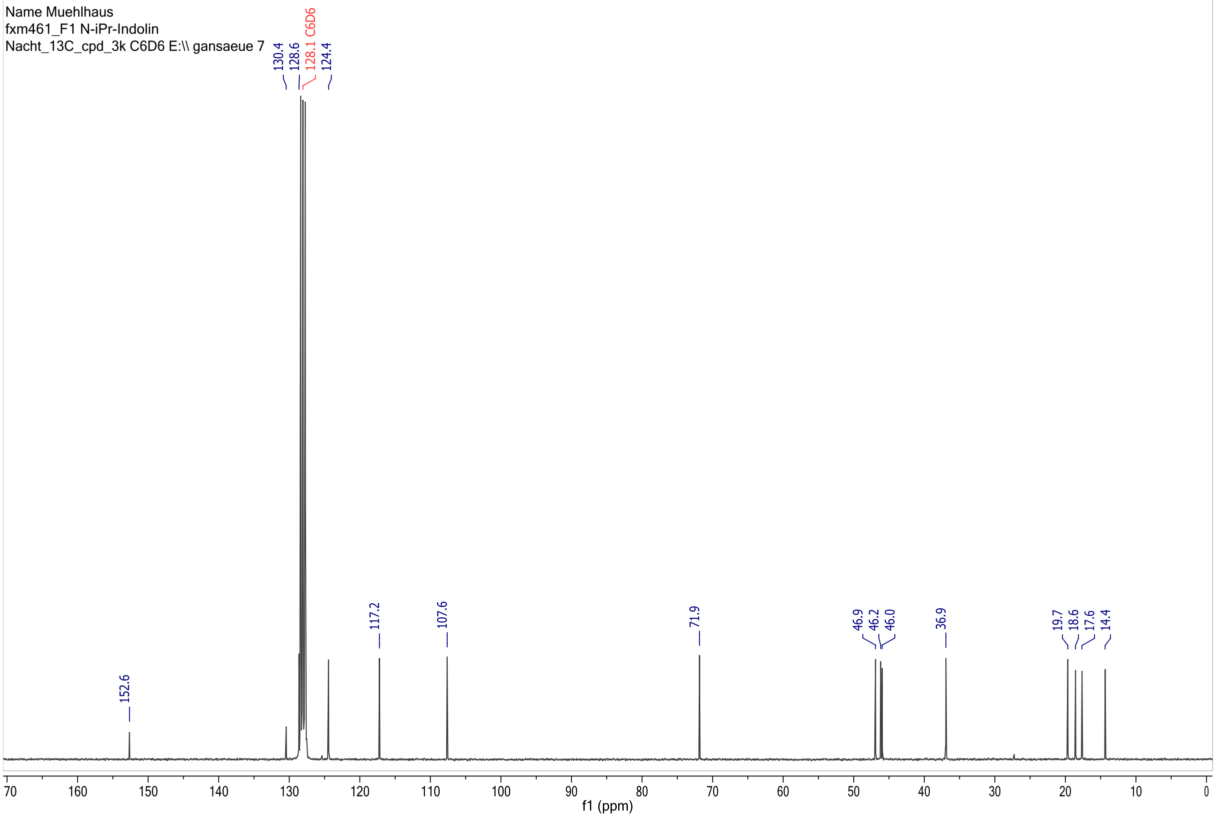

4.4.6 Synthesis of (*S*)-1-((*R*)-5-chloro-1-cyclohexylindolin-3-yl)butan-1-ol (3h).

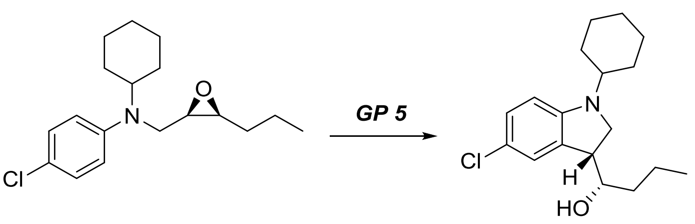

According to GP5: 154 mg of **1h** (1.00 eq., 0.50 mmol), 22 mg lutidinium chloride (0.30 eq., 0.15 mmol), 10 mg zinc (0.30 eq., 0.15 mmol) and 18 mg ***ent*-cat-Cl_2** (0.07 eq., 0.035 mmol) are reacted in dry THF for 48 hours at room temperature. Regio- and diastereoisomeric ratio were determined by ^{13}C-NMR of the crude product. Flash chromatography (SiO_2, Eluent: CH : MTBE = 90 : 10) yielded 88 mg **3h** (d.r. = > 98 : < 2, 57%) as colorless, viscous oil.

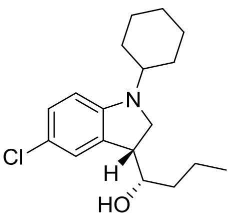

3h
$C_{18}H_{26}ClNO$
307.86 g/mol

R_f = 0.6 (20% EE in CH), **^{1}H-NMR (400 MHz, C_6D_6, RT): δ [ppm]** = 0.85 (t, *J*= 7.1 Hz, 3H), 0.89-1.70 (m, 15H), 2.90 (ddd, *J*= 10.3 Hz, *J*= 7.5 Hz, *J*= 3.6 Hz, 1H), 3.04 (tt, *J*= 10.9 Hz, *J*= 3.3 Hz, 1H), 3.13 (dd, *J*= 9.8 Hz, *J*= 8.8 Hz, 1H), 3.36 (dd, *J*= 8.7 Hz, *J*= 7.3 Hz, 1H), 3.56 (dt, *J*= 8.9 Hz, *J*= 3.6 Hz, 1H) 6.04 (d, *J*= 8.4 Hz, 1H), 7.00 (dd, *J*= 2.2 Hz, *J*= 1.1 Hz, 1H), 7.08 (dd, *J*= 8.4 Hz, *J*= 2.2 Hz, 1H). **^{13}C-NMR (125.5 MHz, C_6D_6, RT): δ [ppm]** = 14.3, 19.6, 26.0, 26.2, 28.1, 29.5, 36.8, 46.7, 47.5, 54.8, 71.6, 107.7, 121.2, 124.8, 128.2, 132.4, 151.0. **IR ṽ [cm^{-1}]** = 444, 475, 525, 551, 576, 603, 682, 715, 738, 795, 841, 885, 959, 1008, 1028, 1073, 1110, 1125, 1144, 1166, 1243, 1269, 1304, 1344, 1385, 1422, 1450, 1488, 1599, 2853, 2926, 3279; **HRMS (ESI)**: *m/z* calculated for $[M+H]^+$ 308.1776 u found: 308.1773 u; . **$[\alpha]_D^{20}$=** – 2.4 (*c* 1, $CHCl_3$).

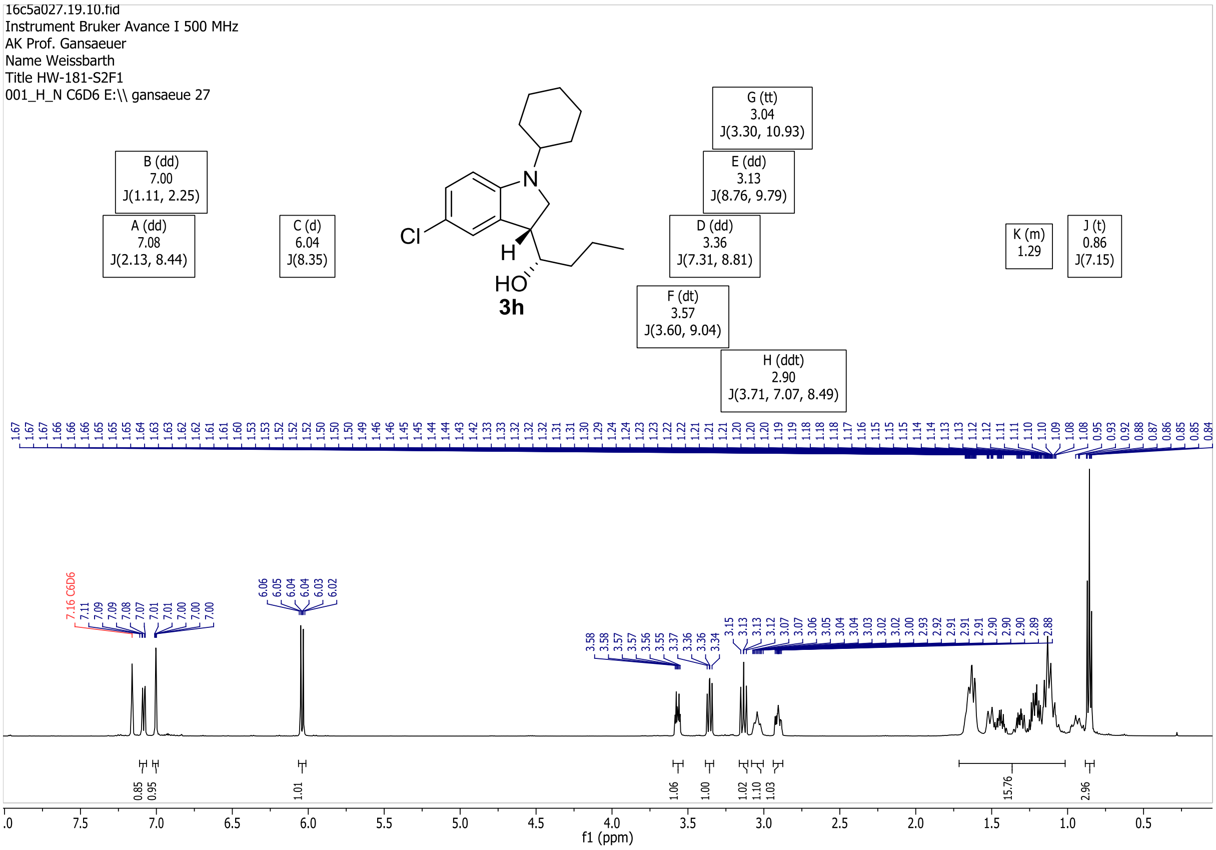
16c5a027.19.10.fid
Instrument Bruker Avance I 500 MHz
AK Prof. Gansaeuer
Name Weissbarth
Title HW-181-S2F1
001_H_N C6D6 E:\\ gansaeue 27
Cl
N
H
HO
3h
A (dd) 7.08 J(2.13, 8.44)
B (dd) 7.00 J(1.11, 2.25)
C (d) 6.04 J(8.35)
D (dd) 3.36 J(7.31, 8.81)
E (dd) 3.13 J(8.76, 9.79)
F (dt) 3.57 J(3.60, 9.04)
G (tt) 3.04 J(3.30, 10.93)
H (ddt) 2.90 J(3.71, 7.07, 8.49)
J (t) 0.86 J(7.15)
K (m) 1.29
7.16 C6D6
0.85
0.95
1.01
1.06
1.00
1.02
1.10
1.03
15.76
2.96
f1 (ppm)

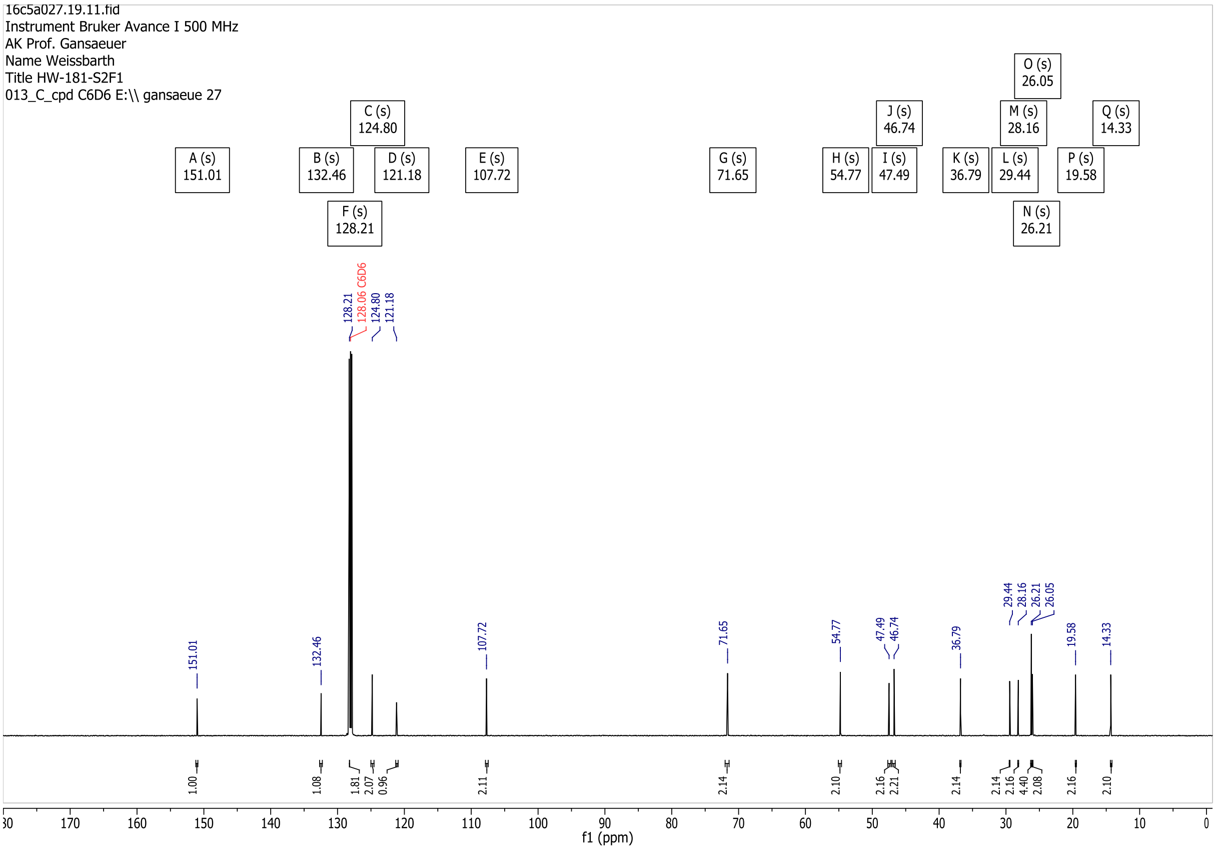
16c5a027.19.11.fid
Instrument Bruker Avance I 500 MHz
AK Prof. Gansaeuer
Name Weissbarth
Title HW-181-S2F1
013_C_cpd C6D6 E:\\ gansaeue 27
A (s) 151.01
B (s) 132.46
C (s) 124.80
D (s) 121.18
E (s) 107.72
F (s) 128.21
G (s) 71.65
H (s) 54.77
I (s) 47.49
J (s) 46.74
K (s) 36.79
L (s) 29.44
M (s) 28.16
N (s) 26.21
O (s) 26.05
P (s) 19.58
Q (s) 14.33
128.06 C6D6
f1 (ppm)

4.4.7 Synthesis of (*S*)-1-((*R*)-5-chloro-1-cyclopentylindolin-3-yl)butan-1-ol (3i).

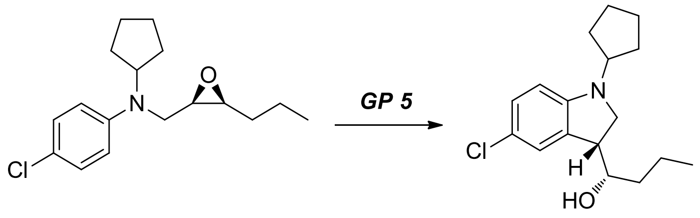

According to GP5: 147 mg of **1i** (1.00 eq., 0.50 mmol), 22 mg lutidinium chloride (0.30 eq., 0.15 mmol), 10 mg zinc (0.30 eq., 0.15 mmol) and 18 mg ***ent*-cat-Cl₂** (0.07 eq., 0.035 mmol) are reacted in dry THF for 48 hours at room temperature. Regio- and diastereoisomeric ratio were determined by ^{13}C-NMR of the crude product. Flash chromatography (SiO_2, Eluent: CH : MTBE = 90 : 10) yielded 85 mg **3i** (d.r. = > 98 : < 2, 58%) as colorless, viscous oil.

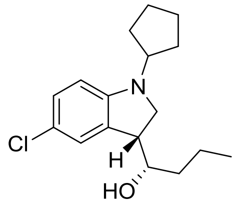

3i
$C_{17}H_{24}ClNO$
293.83 g/mol

$\mathbf{R_f}$ = 0.5 (20% EE in CH), **^{1}H-NMR (400 MHz, C_6D_6, RT): δ [ppm]** = 0.84 (t, *J*= 7.1 Hz, 3H), 1.05 (s_{br}, 1H) 1.14-1.53 (m, 10H), 1.54-1.66 (m, 2H) 2.84-2.92 (m, 1H), 3.11 (t, *J*= 9.1 Hz, 1H) 3.35 (dd, *J*= 8.7 Hz, *J*= 7.3 Hz, 1H), 3.55 (td, *J*= 7.4 Hz, *J*= 3.7 Hz, 2H), 6.11 (d, *J*= 8.4 Hz, 1H), 6.99 (dd, *J*= 2.2 Hz, *J*= 1.1 Hz, 1H), 7.09 (dd, *J*= 8.4 Hz, *J*= 2.2 Hz, 1H). **^{13}C-NMR (125.5 MHz, C_6D_6, RT): δ [ppm]** = 14.3, 19.6, 24.4, 24.5, 27.8, 28.6, 36.9, 46.8, 48.5, 57.9, 71.4, 108.2, 121.6, 124.6, 128.3, 132.7, 152.1. **IR ṽ [cm^{-1}]=** 430, 527, 594, 678, 711, 739, 799, 847, 875, 960, 1004, 1073, 1125, 1195, 1260, 1354, 1393, 1421, 1464, 1487, 1599, 2869, 2955, 3398; **HRMS (ESI)**: *m/z* calculated for $[M+H]^+$: 294.1619 u found: 294.1616 u; $[\alpha]_D^{20}$ = -32.4°(*c* 1, $CHCl_3$).

03t4b055.19.10.fid
Instrument Bruker AV I 400 MHz
AK Prof. Gansaeuer
Name Weissbarth
Titel HW-179p
001_H_N C6D6 E:\\ gansaeue 55

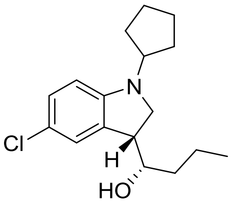

3i

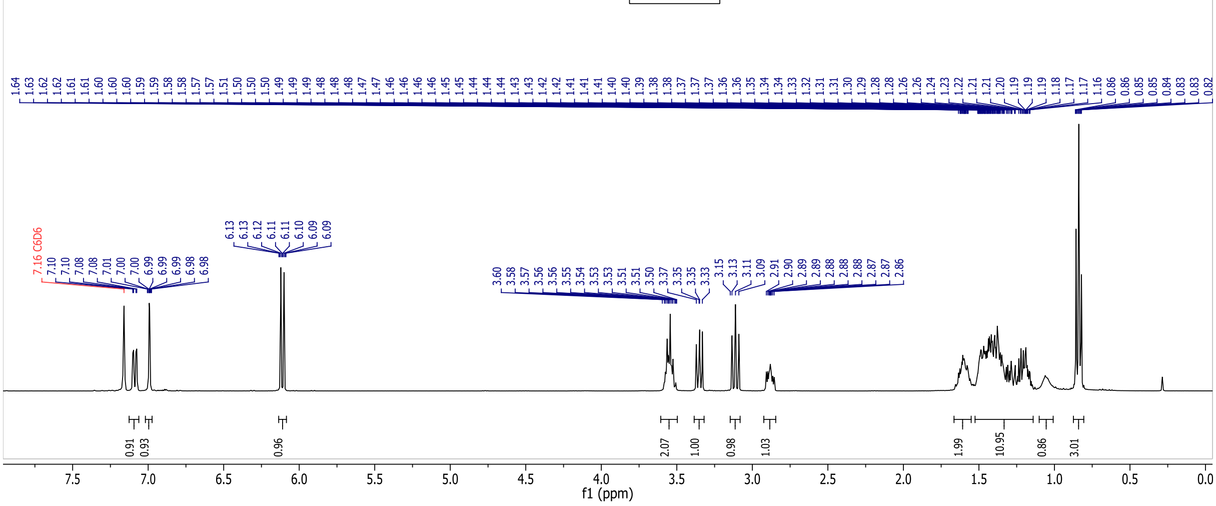

03t4b055.19.11.fid
Instrument Bruker AV I 400 MHz
AK Prof. Gansaeuer
Name Weissbarth
Titel HW-179p
013_C_cpd C6D6 E:\\ gansaeue 55

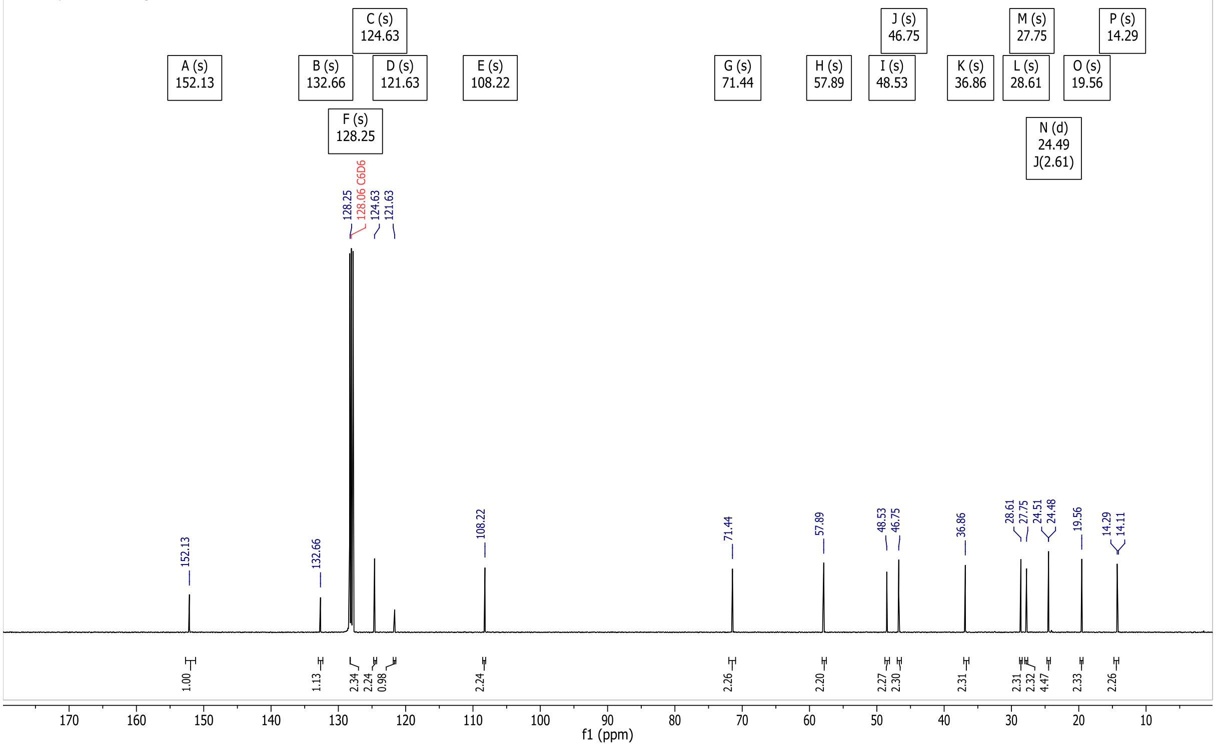

4.4.8 Synthesis of (*S*)-1-((*R*)-5-chloro-1-isopropylindolin-3-yl)butan-1-ol (3j).

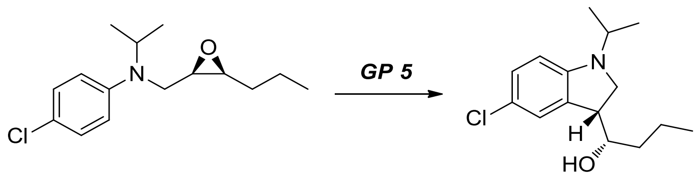

According to GP5: 134 mg of **1j** (1.00 eq., 0.50 mmol), 22 mg lutidinium chloride (0.30 eq., 0.15 mmol), 10 mg zinc (0.30 eq., 0.15 mmol) and 18 mg ***ent*-cat-Cl$_2$** (0.07 eq., 0.035 mmol) are reacted in dry THF for 48 hours at room temperature. Regio- and diastereoisomeric ratio were determined by ^{13}C-NMR of the crude product. Flash chromatography (SiO_2, Eluent: CH : MTBE = 90 : 10) yielded 91 mg **3j** (d.r. = > 98 : < 2, 68%) as colorless, viscous oil.

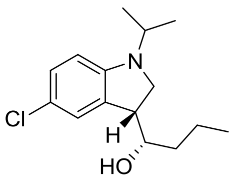

3j
$C_{15}H_{22}ClNO$
267.79 g/mol

R$_f$ = 0.5 (30% MTBE in CH), **^{1}H-NMR (300 MHz, C$_6$D$_6$, RT): δ [ppm]** = 0.81-0.91 (m, 9H), 1.04 (s$_{br}$, 1H) 1.11-1.49 (m, 4H), 2.87 (dddt, *J*= 9.5 Hz, *J*= 7.3 Hz, *J*= 3.7 Hz, *J*= 1.0 Hz, 1H), 3.06 (dd, *J*= 9.7 Hz, *J*= 9.8.7 Hz, 1H) 3.30 (dd, *J*= 8.7 Hz, *J*= 7.3 Hz, 1H), 3.40 (dq, *J*= 13.3 Hz, *J*= 6.7 Hz, 1H), 3.54 (dt, *J*= 8.8 Hz, *J*= 3.6 Hz 1H), 6.00 (d, *J*= 8.4 Hz, 1H), 6.99 (dd, *J*= 2.2 Hz, *J*= 1.1 Hz, 1H), 7.08 (ddd, *J*= 8.3 Hz, *J*= 2.2 Hz, *J*= 0.7 Hz, 1H). **^{13}C-NMR (75 MHz, C$_6$D$_6$, RT): δ [ppm]** = 14.3, 17.4, 18.5, 19.6, 36.8, 46.0, 46.2, 46.6, 71.5, 108.0, 121.5, 124.8, 128.2, 132.6, 151.2. **IR ṽ [cm^{-1}]**= 711, 800, 809, 1124, 1189, 1263, 1471, 1487; **HRMS (ESI)**: *m/z* calculated for $[M+H]^+$ 268.1463 u found: 268.1461 u; **[α]$_D$ ^{20}**= -43.6°(*c* 1, $CHCl_3$).

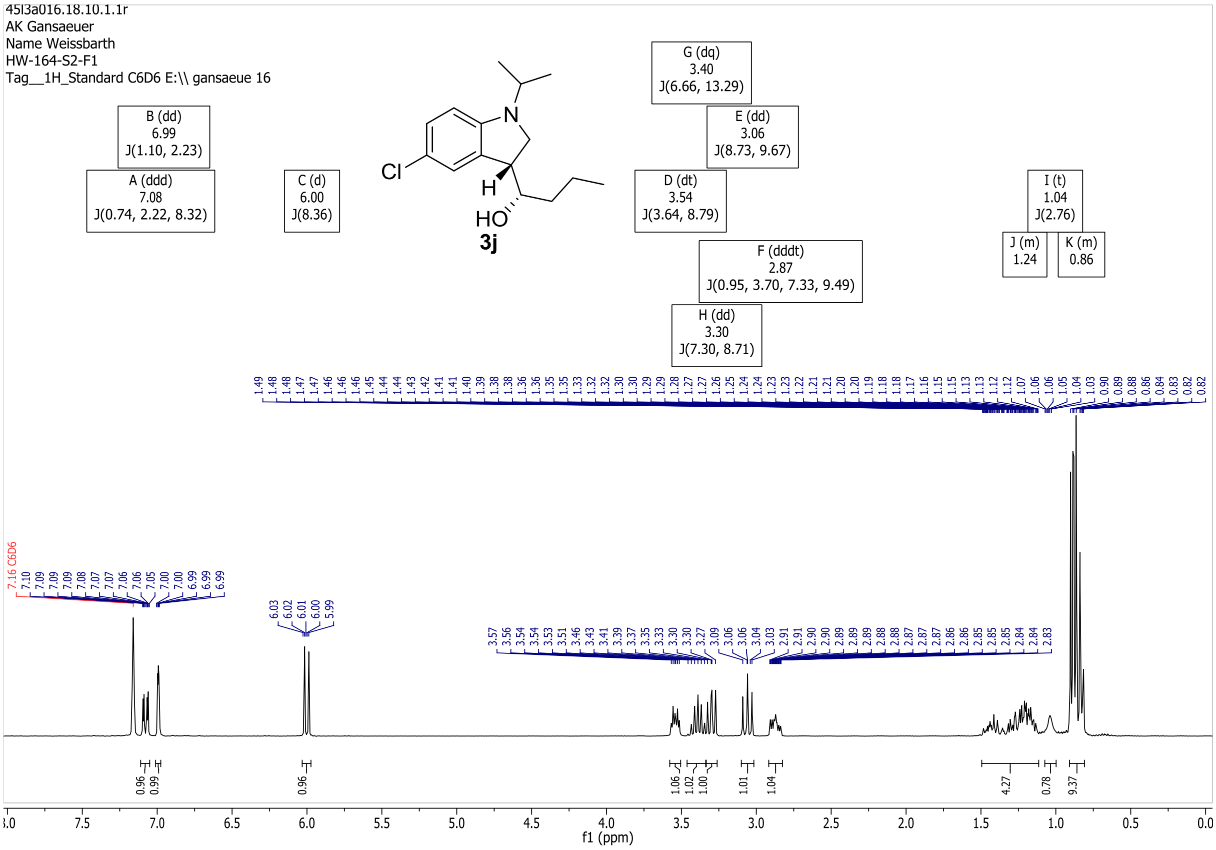
45l3a016.18.10.1.1r
AK Gansaeuer
Name Weissbarth
HW-164-S2-F1
Tag__1H_Standard C6D6 E:\\ gansaeue 16
3j
A (ddd) 7.08 J(0.74, 2.22, 8.32)
B (dd) 6.99 J(1.10, 2.23)
C (d) 6.00 J(8.36)
D (dt) 3.54 J(3.64, 8.79)
E (dd) 3.06 J(8.73, 9.67)
F (dddt) 2.87 J(0.95, 3.70, 7.33, 9.49)
G (dq) 3.40 J(6.66, 13.29)
H (dd) 3.30 J(7.30, 8.71)
I (t) 1.04 J(2.76)
J (m) 1.24
K (m) 0.86
7.16 C6D6
f1 (ppm)

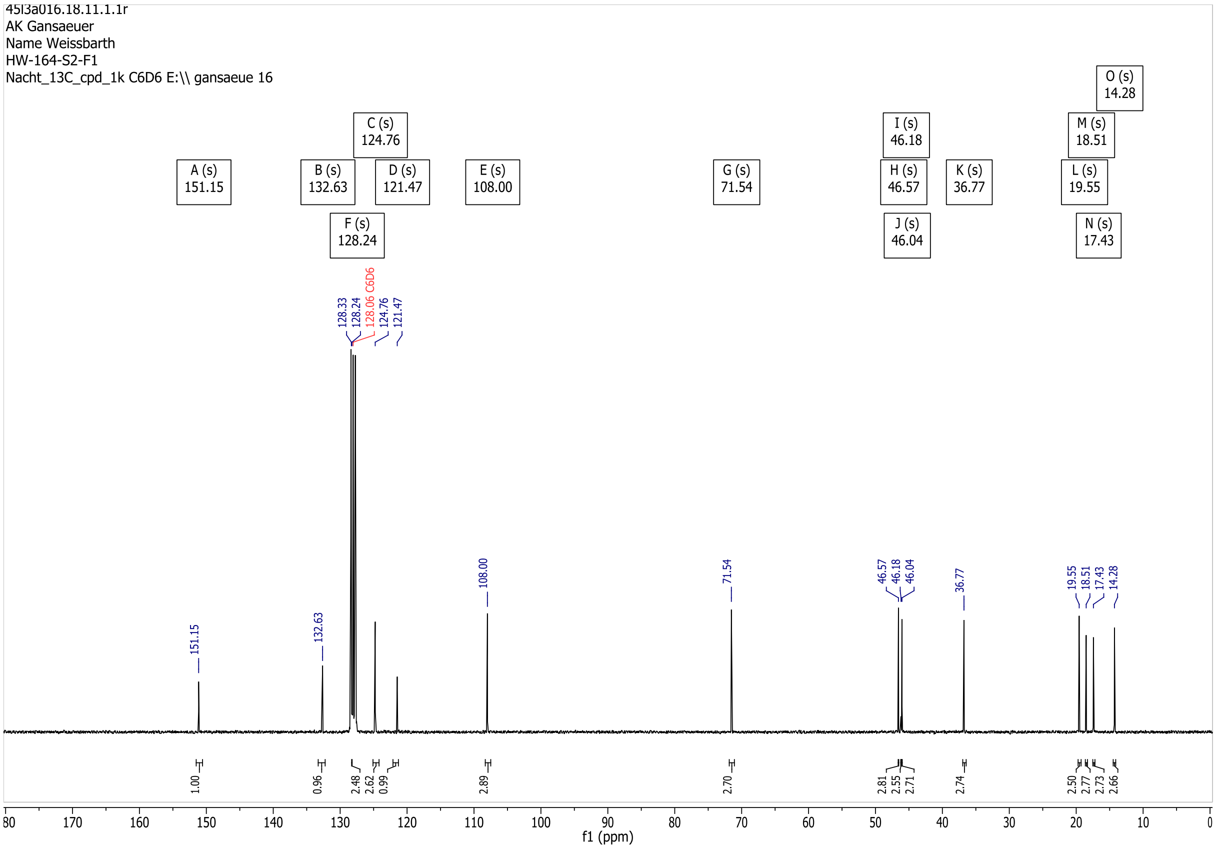
45l3a016.18.11.1.1r
AK Gansaeuer
Name Weissbarth
HW-164-S2-F1
Nacht_13C_cpd_1k C6D6 E:\\ gansaeue 16
A (s) 151.15
B (s) 132.63
C (s) 124.76
D (s) 121.47
E (s) 108.00
F (s) 128.24
G (s) 71.54
H (s) 46.57
I (s) 46.18
J (s) 46.04
K (s) 36.77
L (s) 19.55
M (s) 18.51
N (s) 17.43
O (s) 14.28
128.06 C6D6
f1 (ppm)

4.4.9 Synthesis of (*S*)-1-((*R*)-5-fluoro-1-isopropylindolin-3-yl)butan-1-ol (3k).

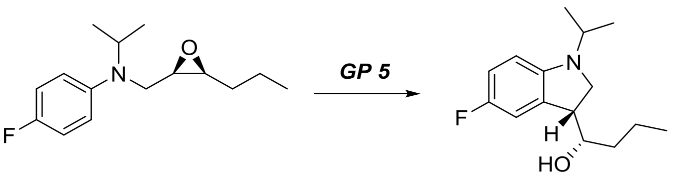

According to GP5: 126 mg of **1k** (1.00 eq., 0.50 mmol), 22 mg lutidinium chloride (0.30 eq., 0.15 mmol), 10 mg zinc (0.30 eq., 0.15 mmol) and 18 mg ***ent*-cat-Cl₂** (0.07 eq., 0.035 mmol) are reacted in dry THF for 48 hours at room temperature. Regio- and diastereoisomeric ratio were determined by ^{13}C-NMR of the crude product. Flash chromatography (SiO_2, Eluent: CH : MTBE = 90 : 10) yielded 58 mg **3k** (d.r. = > 98 : < 2, 46%) as colorless, viscous oil.

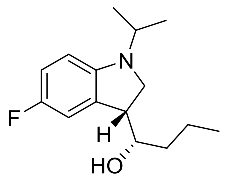

3k
$C_{15}H_{22}FNO$
251,35 g/mol

R_f = 0.5 (20% EE in CH), **^{1}H-NMR (300 MHz, C_6D_6, RT): δ [ppm]** = 0.85 (t, 3H, *J*= 7.1 Hz), 0.91 (d, *J*= 6.6 Hz, 3H), 0.92 (d, *J*= 6.6 Hz, 3H), 1.13 (s_{br}, 1H) 1.16-1.52 (m, 4H), 2.85-2.95 (m, 1H), 3.09 (t, *J*= 9.1 Hz, 1H) 3.30 (dd, *J*= 8.7 Hz, *J*= 7.2 Hz, 1H), 3.43 (hept, *J*= 6.6 Hz, 1H), 3.59 (dt, *J*= 8.1 Hz, *J*= 3.7 Hz 1H), 6.03 (dd, *J*= 8.5 Hz, *J*= 4.2 Hz, 1H), 6.73-6.84 (m, 2H). **^{13}C-NMR (75 MHz, C_6D_6, RT): δ [ppm]** = 14.3, 17.4, 18.3, 19.6, 36.8, 46.3, 46.6, 46.8 (d, *J*=1.8 Hz), 71.6, 107.4 (d, *J*=7.9 Hz), 112.2 (d, *J*=23.7 Hz), 114.2 (d, *J*=22.7 Hz), 132.2 (d, *J*=7.2 Hz), 148.8, 156.5 (d, *J*=233.4 Hz); **^{19}F-NMR (470 MHz, C_6D_6, RT): δ [ppm]** = -128.5; **IR ṽ [cm^{-1}]=** 413, 450, 488, 515, 536, 550, 582, 648, 748, 778, 803, 842, 870, 885, 962, 1002, 1030, 1066, 1082, 1095, 1126, 1192, 1210, 1263, 1364, 1388, 1437, 1470, 4187, 2871, 2908, 2953, 3333; **HRMS (ESI)**: *m/z* calculated for $[M+H]^+$ 252.1758 u found: 252.1755 u; **$[\alpha]_D^{20}$**= -57.6° (*c* 1, $CHCl_3$).

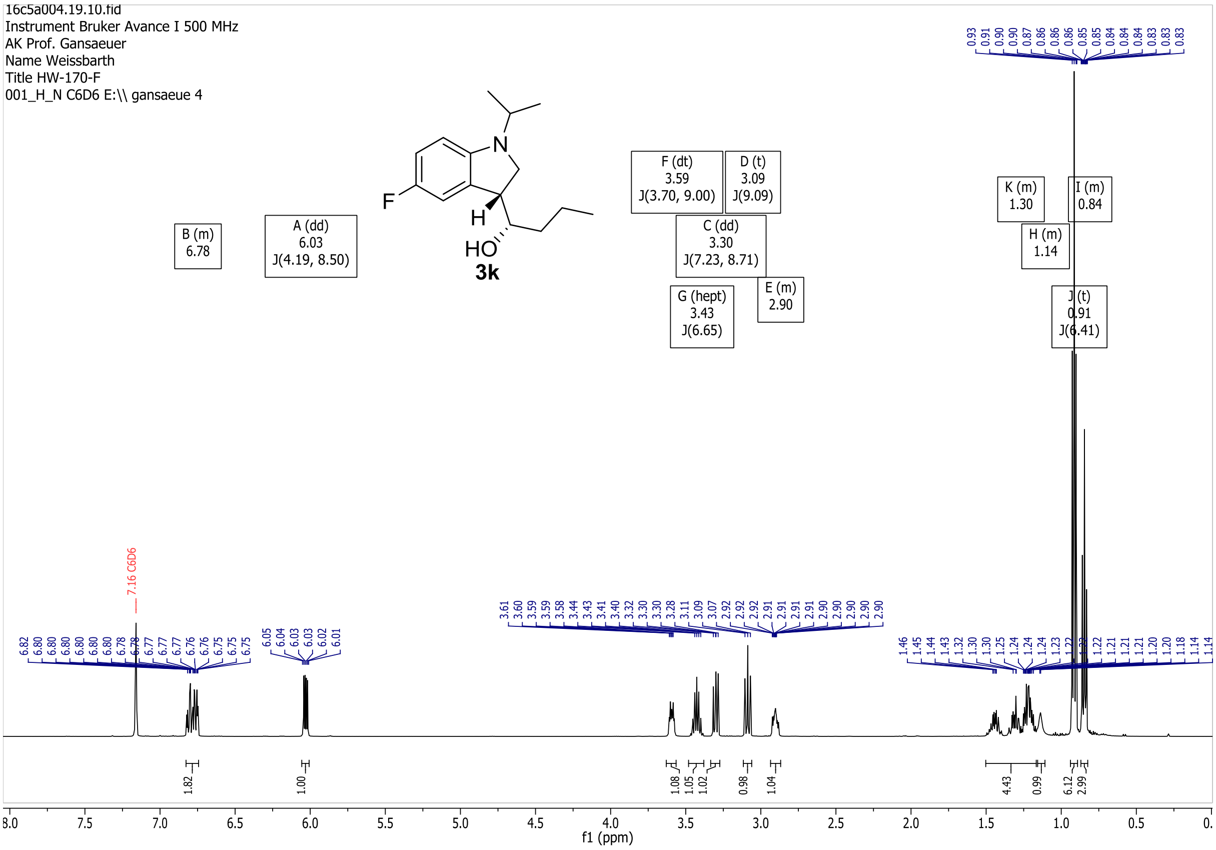
16c5a004.19.10.fid
Instrument Bruker Avance I 500 MHz
AK Prof. Gansaeuer
Name Weissbarth
Title HW-170-F
001_H_N C6D6 E:\\ gansaeue 4
3k
f1 (ppm)

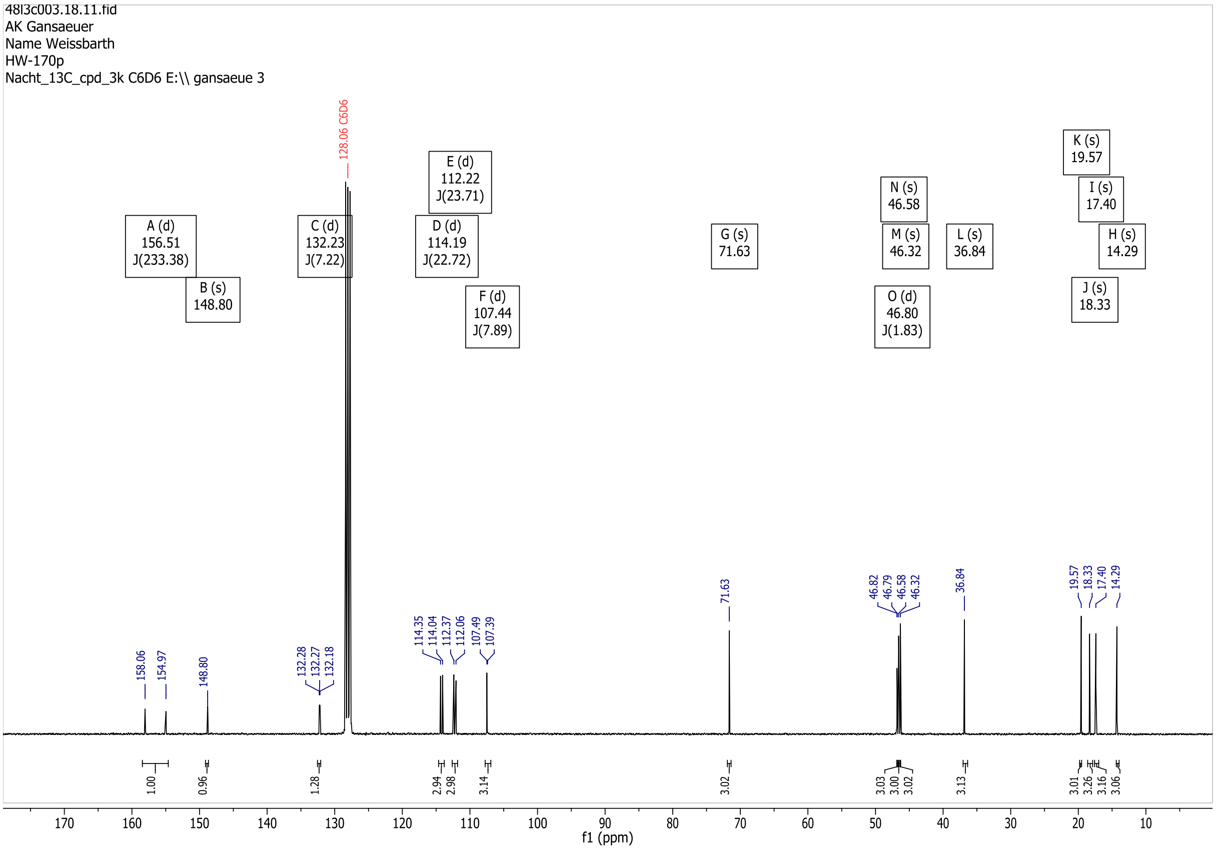
48l3c003.18.11.fid
AK Gansaeuer
Name Weissbarth
HW-170p
Nacht_13C_cpd_3k C6D6 E:\\ gansaeue 3
f1 (ppm)

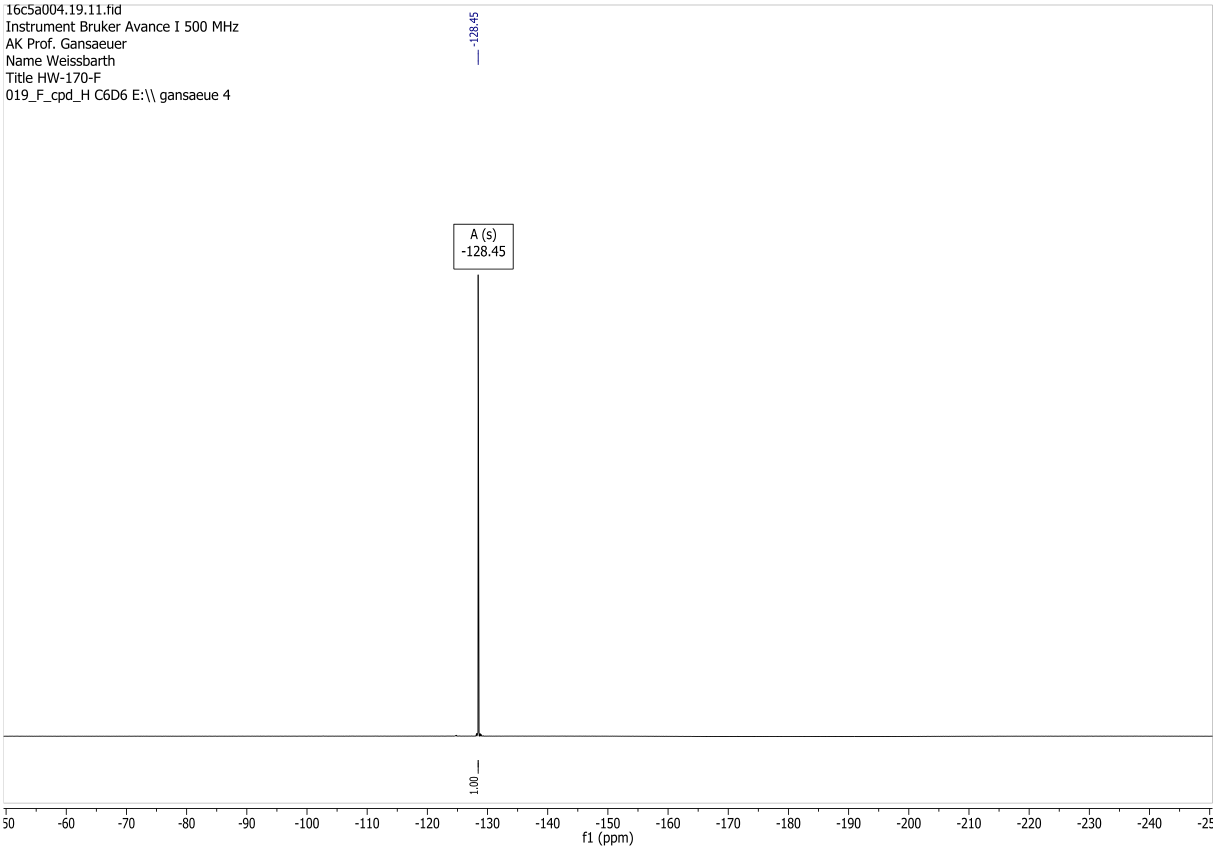
16c5a004.19.11.fid
Instrument Bruker Avance I 500 MHz
AK Prof. Gansaeuer
Name Weissbarth
Title HW-170-F
019_F_cpd_H C6D6 E:\\ gansaeue 4
-128.45
A (s)
-128.45
1.00
-60
-70
-80
-90
-100
-110
-120
-130
-140
-150
-160
-170
-180
-190
-200
-210
-220
-230
-240
f1 (ppm)

4.4.10 Synthesis of (*S*)-1-((*R*)-1-cyclohexyl-5-iodoindolin-3-yl)butan-1-ol (3p).

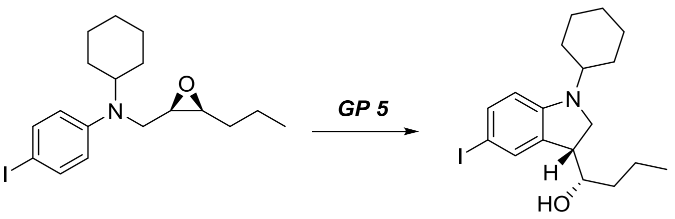

According to GP5: 200 mg of **1p** (1.00 eq., 0.50 mmol), 22 mg lutidinium chloride (0.30 eq., 0.15 mmol), 10 mg zinc (0.30 eq., 0.15 mmol) and 18 mg ***ent*-cat-Cl$_2$** (0.07 eq., 0.035 mmol) are reacted in dry THF for 48 hours at room temperature. Regio- and diastereoisomeric ratio were determined by ^{13}C-NMR of the crude product. Flash chromatography (SiO_2, Eluent: CH : MTBE = 90 : 10) yielded 117 mg **3p** (d.r. = > 98 : < 2, 59%) as colorless, viscous oil. The product has to be stored under argon to prevent rapid decomposition.

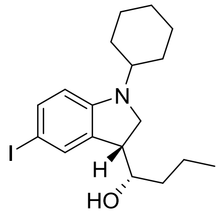

3p
$C_{18}H_{26}INO$
399,32 g/mol

R$_f$ = 0.6 (20% EE in CH), **^{1}H-NMR (500 MHz, C$_6$D$_6$, RT): δ [ppm]** = 0.85 (t, 3H, *J*= 7.2 Hz), 0.88-0.98 (m, 2H), 1.00-1.52 (m, 10H), 1.55-1.67 (m, 3H), 2.88 (ddd, *J*= 9.6 Hz, *J*= 7.4 Hz, *J*= 3.5 Hz, 1H), 3.02 (tq, *J*= 11.2 Hz, *J*= 3.4 Hz, 1H), 3.10 (dd, *J*= 9.9 Hz, *J*= 8.8 Hz, 1H), 3.35 (dd, *J*= 8.8 Hz, *J*= 7.4 Hz, 1H), 3.52 (dt, *J*= 9.0 Hz, *J*= 3.6 Hz 1H), 5.93 (d, *J*= 8.3 Hz, 1H), 7.29 (t, *J*= 1.5 Hz, 1H), 7.40 (dd, *J*= 8.3 Hz, *J*= 1.8 Hz 1H). **^{13}C-NMR (125 MHz, C$_6$D$_6$, RT): δ [ppm]** =14.3, 19.6, 26.0, 26.2, 28.1, 29.5, 36.8, 46.6, 47.2, 54.6, 71.6, 76.5, 109.2, 133.0, 133.6, 137.1, 152.0. **IR ṽ [cm^{-1}]=** 434, 449, 459, 471, 504, 551, 592, 653, 708, 807, 844, 876, 892, 958, 998, 1008, 1083, 1124, 1157, 1227, 1243, 1378, 1424, 1463, 1589, 2886, 2931, 3389 ; **HRMS (ESI)**: *m/z* calculated for $[M+H]^+$ 400.1132 u found: 400.1126 u; **$[\alpha]_D^{20}$**= -6.6° (*c* 1, $CHCl_3$).

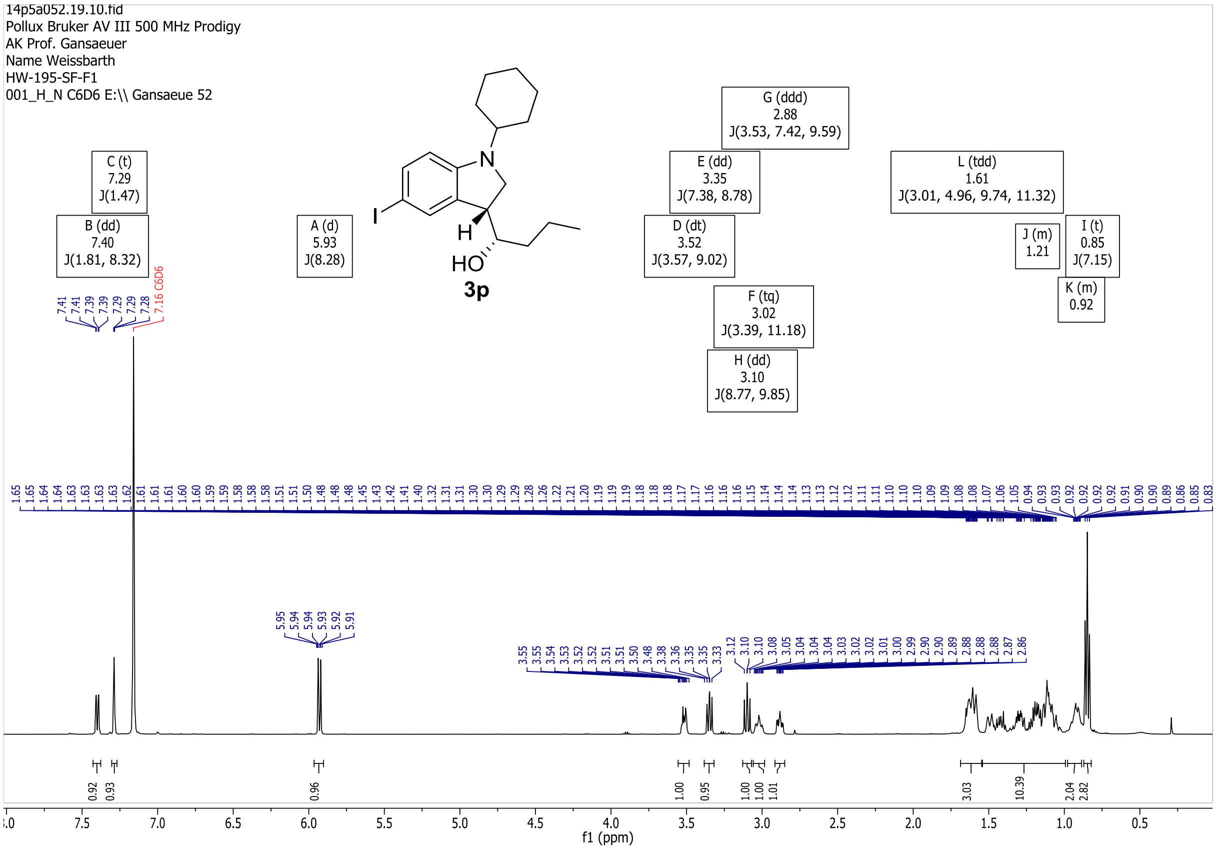
14p5a052.19.10.fid
Pollux Bruker AV III 500 MHz Prodigy
AK Prof. Gansaeuer
Name Weissbarth
HW-195-SF-F1
001_H_N C6D6 E:\\ Gansaeue 52
3p
C (t) 7.29 J(1.47)
B (dd) 7.40 J(1.81, 8.32)
A (d) 5.93 J(8.28)
G (ddd) 2.88 J(3.53, 7.42, 9.59)
E (dd) 3.35 J(7.38, 8.78)
D (dt) 3.52 J(3.57, 9.02)
F (tq) 3.02 J(3.39, 11.18)
H (dd) 3.10 J(8.77, 9.85)
L (tdd) 1.61 J(3.01, 4.96, 9.74, 11.32)
J (m) 1.21
I (t) 0.85 J(7.15)
K (m) 0.92
7.16 C6D6
f1 (ppm)

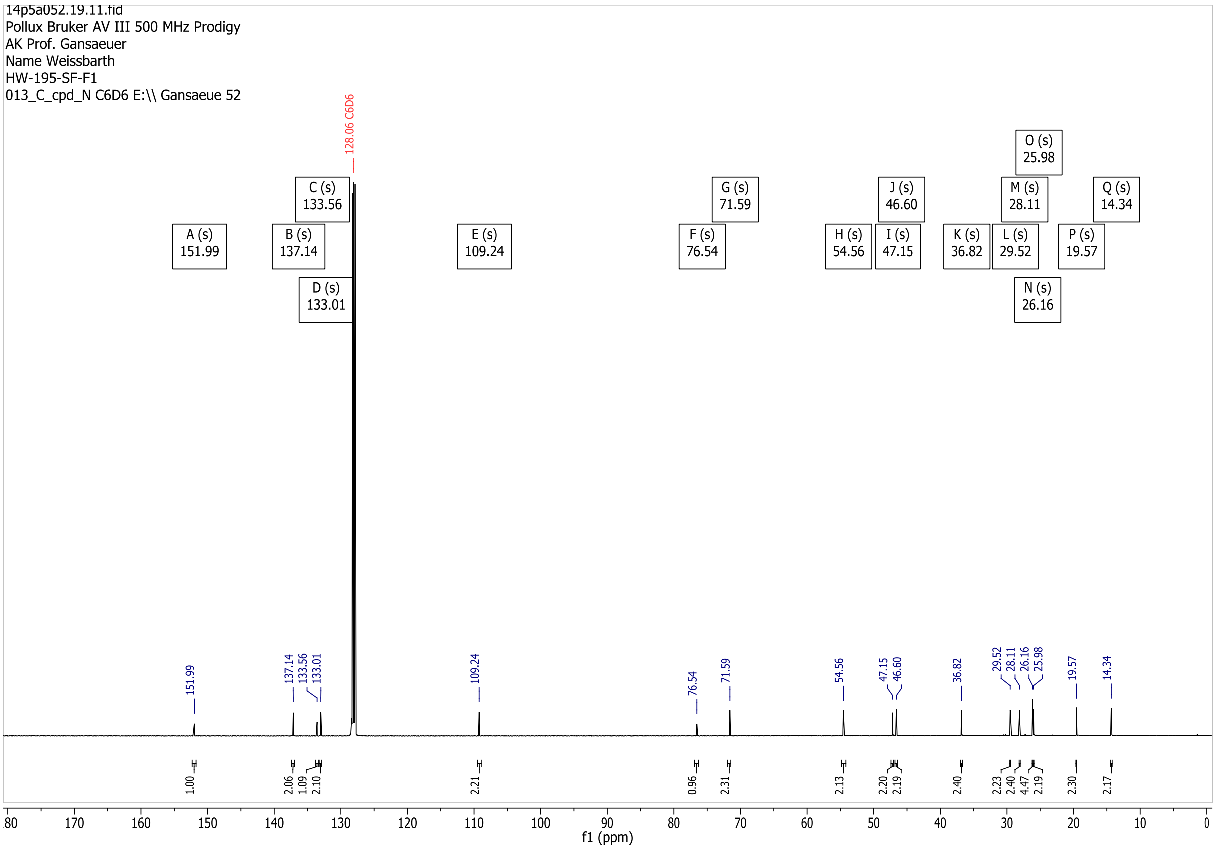
14p5a052.19.11.fid
Pollux Bruker AV III 500 MHz Prodigy
AK Prof. Gansaeuer
Name Weissbarth
HW-195-SF-F1
013_C_cpd_N C6D6 E:\\ Gansaeue 52
128.06 C6D6
A (s) 151.99
B (s) 137.14
C (s) 133.56
D (s) 133.01
E (s) 109.24
F (s) 76.54
G (s) 71.59
H (s) 54.56
I (s) 47.15
J (s) 46.60
K (s) 36.82
L (s) 29.52
M (s) 28.11
N (s) 26.16
O (s) 25.98
P (s) 19.57
Q (s) 14.34
f1 (ppm)

5. Synthesis of catalysts for the REO-ArS$_R$

The Synthesis of **cat-Cl$_2$** and ***ent*-cat-Cl$_2$** were performed according to the literature.[7]

5.1 Synthesis of cat-(OTs)$_2$

1.35 g of **cat-Cl$_2$** (1.00 eq., 2.4 mmol) are dissolved in 20 mL DMF and cooled to 0 °C. Over the course of 10 minutes 3.8 mL (2.5 eq., 6.1 mmol) of 1.6M MeLi-solution are added dropwised. After stirring for 30 minutes, 50 mL of 6 w% NH_4Cl-solution are added, phases are separated and the organic layer is washed with H_2O and brine. The crude product is reacted without further purification.

The solution of **cat-Me$_2$** in Et_2O is transferred to a Schlenk flask and cooled to 0°C. 0.92 g *p*-Toluenesulfonic acid (2.00 eq., 4.8 mmol) are added. After stirring for 30 minutes at 0°C the ice bath is removed and the reaction mixture is stirred for 90 minutes at room temperature. Half of the solvent is removed under reduced pressure and *n*-pentane is added. The precipitated solid is washed with n-pentane and Et_2O and dried *in vacuo* for 16h. Cat-(OTs)2 is obtained as an orange solid (1.35 g, 1.7 mmol, 70%).

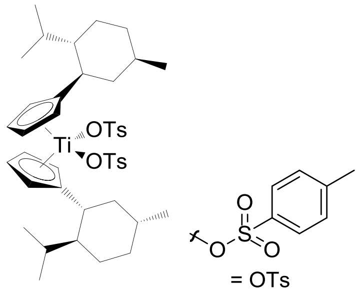

cat-(OTs)$_2$
$C_{44}H_{60}O_6S_2Ti$
796.94 g/mol

^{1}H-NMR (500 MHz, C_6D_6, RT): δ [ppm] = 0.57 (d, 6H, *J* = 6.9 Hz), 0.63 (d, 6H, *J* = 6.9 Hz), 0.75-0.88 (m, 8H), 1.01 (d, 6H, *J* = 5.6 Hz), 1.08-1.16 (m, 2H), 1.29 (dhept, 2H, *J* = 6.7 Hz, *J* = 2.0 Hz), 1.46-1.52 (m, 2H), 1.63 (dd, 2H, *J* = 11.6 Hz, *J* = 3.3 Hz, *J* = 2.1 Hz), 1.73-1.79 (m, 2H), 1.97 (s, 6H), 2.20-2.28 (m, 2H), 6.09 (dd, 2H, *J* = 2.8 Hz, *J* = 2.8 Hz), 6.66 (dd, 2H, *J* = 2.4 Hz, *J* = 2.4 Hz), 6.82 (s, 2H), 6.97 (d, 4H, *J* = 8.0 Hz); 7.22 (s, 2H), 8.32 (d, 4H, *J* = 8.1 Hz). **^{13}C-NMR (125 MHz, C_6D_6, RT): δ [ppm] =** 15.3, 21.1, 21.6, 22.9, 25.0, 27.6, 32.6, 35.2, 40.2, 41.5, 50.6, 110.3, 122.1, 127.4, 129.4, 140.9, 141.8, 150.4.

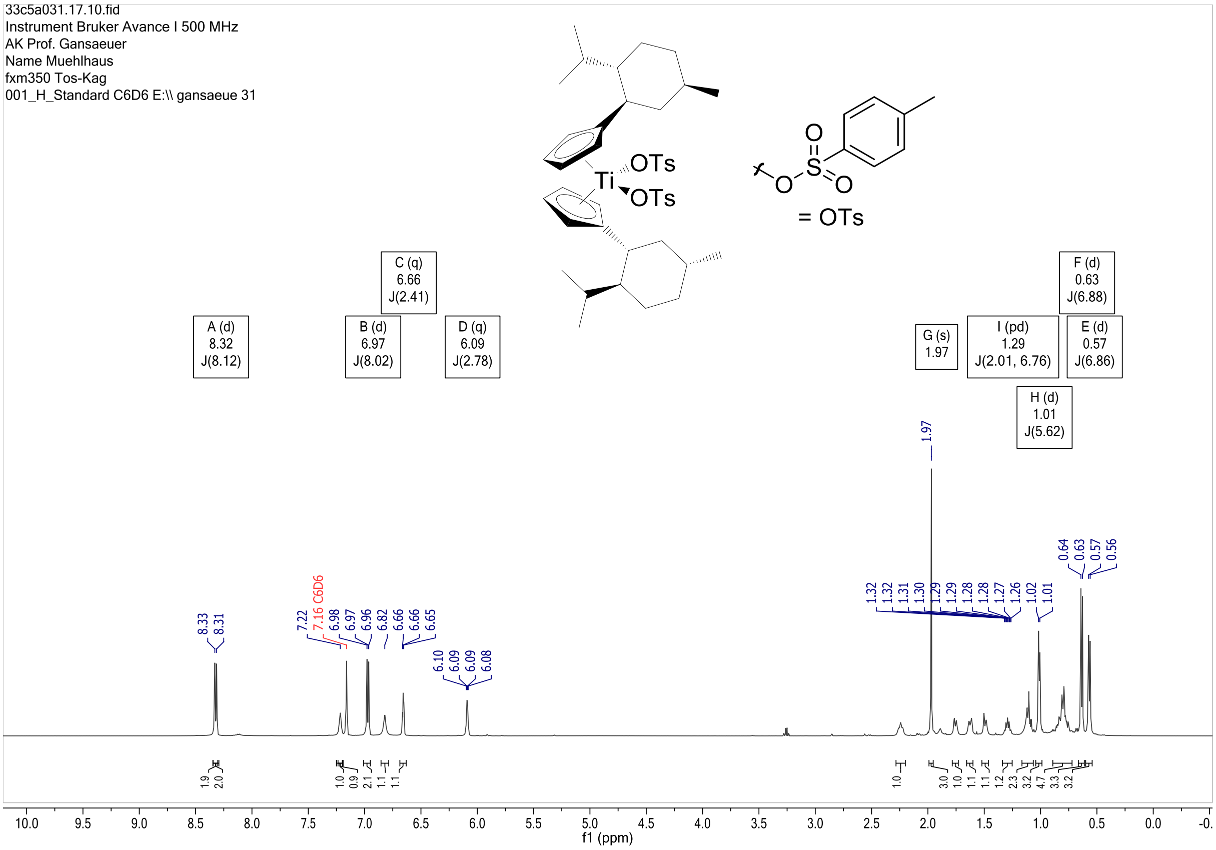
33c5a031.17.10.fid
Instrument Bruker Avance I 500 MHz
AK Prof. Gansaeuer
Name Muehlhaus
fxm350 Tos-Kag
001_H_Standard C6D6 E:\\ gansaeue 31
OTs
Ti
OTs
= OTs
A (d) 8.32 J(8.12)
B (d) 6.97 J(8.02)
C (q) 6.66 J(2.41)
D (q) 6.09 J(2.78)
G (s) 1.97
I (pd) 1.29 J(2.01, 6.76)
H (d) 1.01 J(5.62)
F (d) 0.63 J(6.88)
E (d) 0.57 J(6.86)
7.16 C6D6
f1 (ppm)

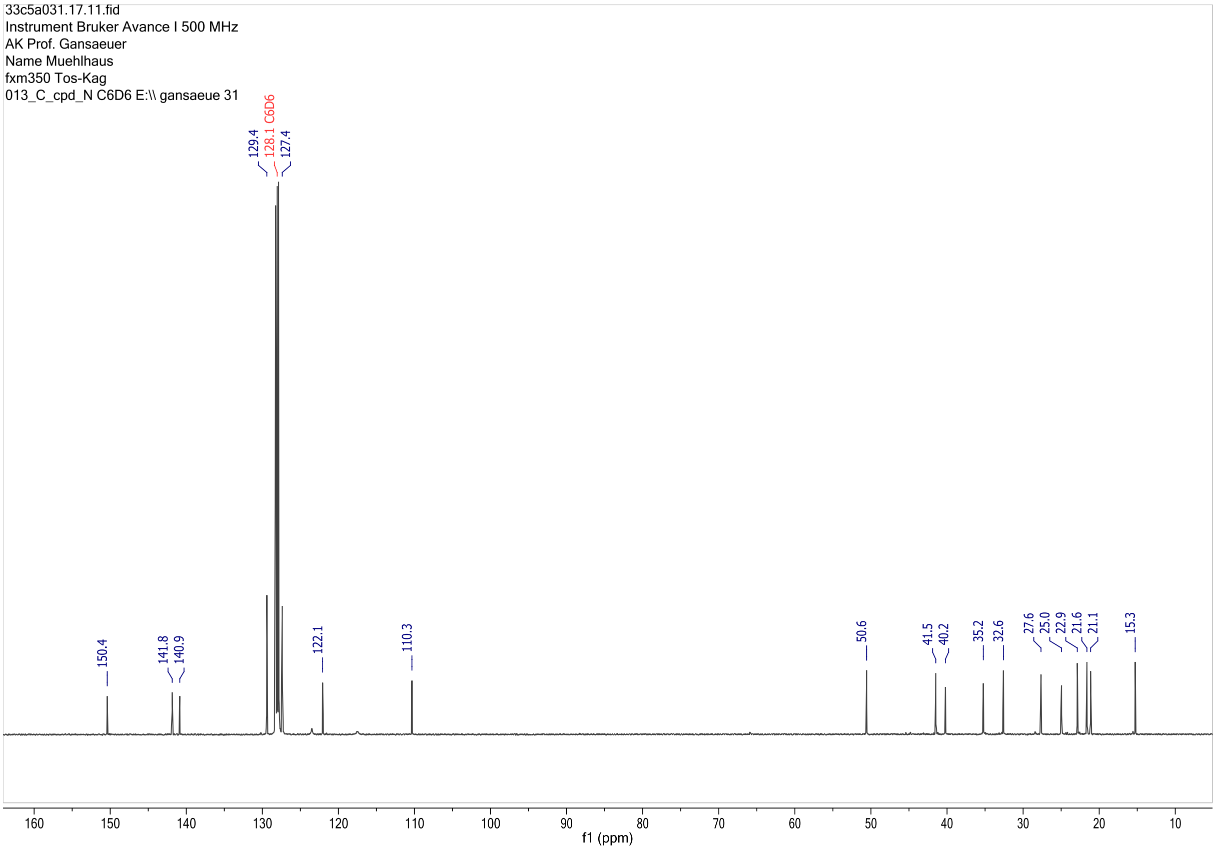
33c5a031.17.11.fid
Instrument Bruker Avance I 500 MHz
AK Prof. Gansaeuer
Name Muehlhaus
fxm350 Tos-Kag
013_C_cpd_N C6D6 E:\\ gansaeue 31
150.4
141.8
140.9
129.4
128.1 C6D6
127.4
122.1
110.3
50.6
41.5
40.2
35.2
32.6
27.6
25.0
22.9
21.6
21.1
15.3
f1 (ppm)

6. References

[1] D. A. L. Otte, D. E. Borchmann, C. Lin, M. Weck, K. A. Woerpel, *Org. Lett.* **2014**, *16*, 1566-1569.

[2] a) G. M. Sheldrick, *Acta Cryst.* **2015***, A71***,** 3-8; b) G. M. Sheldrick, *Acta Cryst.* **2015***, C71***,** 3-8.

[2] D. X. Hu, G. M. Shibaya, N.Z. Burns, *J. Am. Chem. Soc.* **2013**, *135*, 12960-12963.

[3] C. Meister, H.-D. Scharf, *Synthesis* **1981**, *9*, 733-736.

[4] a) M. Tokunaga, J. F. Larrow, K. Kakuichi, E. N. Jacobsen, *Science* **1997**, *277*, 936-938; b) S. E. Schauss, B. D. Brandes, J. F. Larrow, M. Tokunaga, K. B. Hansen, A. E. Gould, M. E. Furrow, E. N. Jacobsen, *J. Am. Chem. Soc.* **2002**, *124*, 1307-1315.

[5] a) L. E. Overman, L. A. Flippin, *Tetrahedron Lett.* **1981**, *22*, 195-196; b) L. E. Overman, M. Kakimoto, M. E. Okazaki, G. P. Meier, *J. Am. Chem. Soc.* **1983**, *105*, 6622-6629.

[6] A. Gansäuer, C. Kube, K. Daasbjerg, R. Sure, S. Grimme, G. D. Fianu, D. V. Sadasivam, R. A. Flowers II, *J. Am. Chem. Soc.* **2014**, *136*, 1663-1671.

[7] A. Gansäuer, S. Narayan, N. Schiffer-Ndene, H. Bluhm, J. E. Oltra, J. M. Cuerva, A. Rosales, M. Nieger, *J. Organomet. Chem.* **2006**, *691*, 2327-2331.
